# Supplementary material for: Study designs for clinical trials applied to personalised medicine: a scoping review
Source: BMJ Open. 2022 May 6;12(5):e052926. doi: 10.1136/bmjopen-2021-052926 (PMC9083424; doi:10.1136/bmjopen-2021-052926)
Supplement: Author's manuscript [file bmjopen-2021-052926.draft_revisions.pdf]

BMJ Open is committed to open peer review. As part of this commitment we make the peer review history of every article we publish publicly available.

When an article is published we post the peer reviewers' comments and the authors' responses online. We also post the versions of the paper that were used during peer review. These are the versions that the peer review comments apply to.

The versions of the paper that follow are the versions that were submitted during the peer review process. They are not the versions of record or the final published versions. They should not be cited or distributed as the published version of this manuscript.

BMJ Open is an open access journal and the full, final, typeset and author-corrected version of record of the manuscript is available on our site with no access controls, subscription charges or pay-per-view fees (http://bmjopen.bmj.com).

If you have any questions on BMJ Open's open peer review process please email info.bmjopen@bmj.com

BMJ Open

Study designs for clinical trials applied to personalised medicine: a scoping review

Journal:	*BMJ Open*
Manuscript ID	bmjopen-2021-052926
Article Type:	Original research
Date Submitted by the Author:	28-Apr-2021

Complete List of Authors:	Superchi, Cecilia; Centre de Recherche Épidémiologie et StatistiqueS Université de Paris (CRESS-UMR1153)<br>Brion Bouvier, Florie; Centre de Recherche Épidémiologie et StatistiqueS Université de Paris (CRESS-UMR1153)<br>Gerardi, Chiara; Istituto di Ricerche Farmacologiche Mario Negri, Center for Health Regulatory Policies<br>Carmona, Montserrat; Instituto de Salud Carlos III, Agencia de Evaluación de Tecnologias Sanitarias; Red de Investigación en Servicios de Salud en Enfermedades Crónicas (REDISSEC)<br>San Miguel, Lorena; Belgian Health Care Knowledge Centre (KCE)<br>Sanchez-Gomez, Luis; Instituto de Salud Carlos III, Agencia de Evaluación de Tecnologias Sanitarias; Red de Investigación en Servicios de Salud en Enfermedades Crónicas (REDISSEC)<br>Imaz-Iglesia, Iñaki; Instituto de Salud Carlos III, Agencia de Evaluación de Tecnologias Sanitarias; Red de Investigación en Servicios de Salud en Enfermedades Crónicas (REDISSEC)<br>Garcia, Paula; European Clinical Research Infrastructure Network (ECRIN)<br>Demotes, Jacques; European Clinical Research Infrastructure Network (ECRIN)<br>Banzi, Rita; Istituto di Ricerche Farmacologiche Mario Negri, Center for Health Regulatory Policies<br>Porcher, Raphaël; Centre de Recherche Épidémiologie et StatistiqueS Université de Paris (CRESS-UMR1153)

Keywords:	EPIDEMIOLOGY, STATISTICS & RESEARCH METHODS, PUBLIC HEALTH

SCHOLARONE™
Manuscripts

I, the Submitting Author has the right to grant and does grant on behalf of all authors of the Work (as defined in the below author licence), an exclusive licence and/or a non-exclusive licence for contributions from authors who are: i) UK Crown employees; ii) where BMJ has agreed a CC-BY licence shall apply, and/or iii) in accordance with the terms applicable for US Federal Government officers or employees acting as part of their official duties; on a worldwide, perpetual, irrevocable, royalty-free basis to BMJ Publishing Group Ltd ("BMJ") its licensees and where the relevant Journal is co-owned by BMJ to the co-owners of the Journal, to publish the Work in this journal and any other BMJ products and to exploit all rights, as set out in our licence.

The Submitting Author accepts and understands that any supply made under these terms is made by BMJ to the Submitting Author unless you are acting as an employee on behalf of your employer or a postgraduate student of an affiliated institution which is paying any applicable article publishing charge ("APC") for Open Access articles. Where the Submitting Author wishes to make the Work available on an Open Access basis (and intends to pay the relevant APC), the terms of reuse of such Open Access shall be governed by a Creative Commons licence – details of these licences and which Creative Commons licence will apply to this Work are set out in our licence referred to above.

Other than as permitted in any relevant BMJ Author's Self Archiving Policies, I confirm this Work has not been accepted for publication elsewhere, is not being considered for publication elsewhere and does not duplicate material already published. I confirm all authors consent to publication of this Work and authorise the granting of this licence.

For peer review only

Title

Study designs for clinical trials applied to personalised medicine: a scoping review

Authors

Cecilia Superchi[1], Florie Brion Bouvier[1], Chiara Gerardi[2], Montserrat Carmona[3,4], Lorena San Miguel[5], Luis María Sánchez-Gómez[3,4], Iñaki Imaz-Iglesia[3,4], Paula Garcia[6], Jacques Demotes-Mainard[6], Rita Banzi[2], Raphaël Porcher[1], the PERMIT Group

Affiliations

1. Centre of Research in Epidemiology and Statistics, Université de Paris (CRESS-UMR1153), Paris, France
2. Center for Health Regulatory Policies, Istituto di Ricerche Farmacologiche Mario Negri IRCCS, Milan, Italy
3. Agencia de Evaluación de Tecnologias Sanitarias (AETS), Instituto de Salud Carlos III, Madrid, Spain
4. Red de Investigación en Servicios de Salud en Enfermedades Crónicas (REDISSEC). Madrid, Spain
5. Belgian Health Care Knowledge Centre (KCE), Brussel, Belgium
6. European Clinical Research Infrastructure Network (ECRIN), Paris, France

Corresponding author:

Cecilia Superchi

Centre de Recherche Épidémiologie et StatistiqueS Université de Paris (CRESS-UMR1153)

Hôpital Hôtel-Dieu, 1 Place du Parvis Notre-Dame, 75004 Paris, France

cecilia.superchi@parisdescartes.fr | cecilia.superchi@gmail.com

ORCID IDs

Cecilia Superchi: https://orcid.org/0000-0002-5375-6018

Florie Brion Bouvier

Chiara Gerardi https://orcid.org/0000-0002-2459-4769

Montserrat Carmona Rodriguez https://orcid.org/0000-0002-4680-7745

Lorena San Miguel https://orcid.org/0000-0002-7155-7303

Luis María Sánchez-Gómez https://orcid.org/0000-0003-4358-6032

Iñaki Imaz-Iglesia https://orcid.org/0000-0002-7864-419

Paula Garcia

Jacques Demotes-Mainard: https://orcid.org/0000-0002-0807-0746

Rita Banzi https://orcid.org/0000-0002-2211-3300

Raphaël Porcher: https://orcid.org/0000-0002-5277-4679

Word count: 300 (abstract, word limit: 300), 4111 (main text, word limit: 4000)

For peer review only

Abstract

Objective: Personalised medicine allows treating patients based on their individual demographic, genomic or biological characteristics for tailoring the *'right treatment for the right person at the right time'*. Robust methodology is required for personalised medicine clinical trials, to correctly identify groups of participants and treatments. As an initial step for the development of new recommendations on trial designs for personalised medicine, we aimed to present an overview of the study designs that have been used in this field.

Design: Scoping review

Methods: We searched (April 2020) PubMed, EMBASE and the Cochrane Library for all reports in English, French, German, Italian and Spanish, describing study designs for clinical trials applied to personalised medicine. Study selection and data extraction were performed in duplicate resolving disagreements by consensus or by involving a third expert reviewer. We extracted information on the characteristics of trial designs and examples of current applications of these approaches. The extracted information was used to generate a new classification of trial designs for personalised medicine.

Results: We identified 23 trial designs, 9 subtypes, and 30 variations of trial designs applied to personalised medicine, which we classified into four core categories (namely, Master protocol, Randomise-all, Biomarker strategy and Enrichment). We found 132 clinical trials using these designs, of which the great majority were master protocols (85/132, 64.4%). Most of the trials were phase II studies (76/132, 57.6%) in the field of oncology (114/132, 86.4%). We identified 29 main features of trial designs regarding different aspects (e.g., framework, control group, randomisation). The four core categories and 29 features were merged into a double-entry table to create a new classification of trial designs for personalised medicine.

Conclusions: A variety of trial designs exists applied to personalised medicine. More research is needed to identify and report on the pros and cons of each approach.

Keywords

Precision medicine, Clinical trial, Study design, Scoping review

Article Summary

- This is the first overview of all trial designs applied to personalised medicine.
- The screening process and data extraction were performed in duplicate.
- A new classification of trial designs for personalised medicine has been proposed.
- Although we systematically searched for trial designs applied to personalised medicine, we cannot exclude that we missed some relevant designs since we restricted the search to the last 15 years.

For peer review only

Introduction

Personalised medicine is an evolving field, which allows treating patients by providing them a specific therapy according to their individual demographic, genomic or biological characteristics (1). Patient stratification is therefore, a prerequisite for testing treatment options targeted to the characteristics of an identified cluster of patients.

Over the last years, many complex innovative designs have been proposed to evaluate targeted treatments in patients' groups (2). According to the Clinical Trials Facilitation and Coordination Group, a clinical trial is considered using a complex design "if it has separate parts that could constitute individual clinical trials and/or is characterised by extensive prospective adaptations such as planned additions of new Investigational Medicinal Products (IMPs) or new target populations" (3). Examples of complex designs are the so-called basket, umbrella, and platform trials, which are frequently applied in the field of oncology (4). Basket trials refer to designs in which patients with heterogeneous diagnoses but with similar disease mechanisms are tested using the same targeted therapy. Contrary, umbrella trials evaluate multiple treatment options in patient groups, which present the same disease, but with different genetic mutations. Finally, platform trials permit testing multiple targeted therapies in patients with the same disease in a perpetual manner, using interim evaluations and allowing therapies to enter or leave the trial (5).

Numerous methodological challenges exist due to the complexity of these three types of trial designs (6), which often require independent statistical analyses for each sub-protocol, including interim analyses driving prospective adaptation with the addition of new interventions or populations, and/or termination of sub-protocols based on futility or safety issues.

The application of robust methodologies is especially important for clinical trials applied to personalised medicine to correctly select participants and treatments to be tested. As a starting point for the development of new recommendations on the use of trial designs applied to personalised medicine, we aimed to present an overview of the existing study designs for clinical trials applied to this medical field.

Our specific objectives were to answer to the following five research questions:

1. What are the available designs for clinical trials applied to personalised medicine?
2. What are the examples of current applications of these approaches?
3. What are the pros and cons of the different approaches?
4. How is a personalised medicine strategy vs. non-personalised strategy evaluated?
5. What are the gaps in the current research on personalised medicine clinical trials?

This scoping review is part of the PERMIT project (PERsonalised MedIcine Trials) aimed at mapping the methods for personalised medicine research and building recommendations on robustness and reproducibility of different stages of the development programmes. Although several categorization may be proposed, the PERMIT project considers four main building blocks of the personalised medicine research pipeline: 1) design, building and management of stratification and validation cohorts; 2) application of machine learning methods for patient stratification; 3) use of preclinical methods for translational development, including the use of preclinical models used to assign treatments to patient clusters; 4) evaluation of treatments in randomised clinical trials. This scoping review covers the fourth building block in this framework.

For peer review only

Methods

We conducted a scoping review following the methodological framework suggested by the Joanna Briggs Institute (7). The framework consists of six stages: 1) identifying the research questions, 2) identifying relevant studies, 3) selecting the studies, 4) charting the data, 5) collating, summarising and reporting results and 6) pursuing a consultation.

A study protocol was published in the Zenodo before conducting the review (8). Due to the iterative nature of scoping reviews, deviations from the protocol were expected and duly reported when occurred. We used the PRISMA-ScR (Preferred Reporting Items for Systematic reviews and Meta-Analyses extension for Scoping Reviews) checklist to report our results (9).

Study identification

Relevant studies and documents were identified balancing feasibility with breadth and comprehensiveness of searches. We searched PubMed, EMBASE and the Cochrane Library (search date: April 7-8, 2020) for reports describing a study design for clinical trials applied to personalised medicine. Online supplementary file 1 reports the search strategies applied. Because many systematic and narrative reviews on trial designs applied to personalised medicine have already been published over the last years, we limited our search from 2005 to April 2020. We restricted inclusion to English, French, German Italian, and Spanish languages. We searched for the grey literature on websites of existing projects about innovative clinical trials (e.g., EU-PEARL) and by consulting partners of the PERMIT project.

Eligibility criteria and deviation from the protocol

We included all reports describing a trial design applied to personalised medicine. The operational definition of personalised medicine used in the present study is reported in Box 1. Because of the extensive volume of literature related to trial designs in personalised medicine, we restricted the inclusion criteria to trial designs for Phase II, III and IV. We excluded single-arm trials, which are not part of a master protocol, non-adaptive enrichment design and N-of-1 trials. We also excluded publications such as prefaces to a special issue and speaker, symposium and panel abstracts, posters and letters to the editor due to the limited information usually provided. These exclusion criteria were not specified in the protocol, but they were agreed among the authors before starting the screening process. The research question "*What are the pros and cons of the different approaches?*" is not reported in the present scoping review, and will be subject to a specific study.

Study selection

We exported the references retrieved from the searches into the Rayyan online tool (10). Duplicates were removed automatically using the reference manager Endnote X9 (Clarivate Analytics, Philadelphia, United States) and manually by one author (CS). Five reviewers independently screened the titles and abstract: one reviewer (CS) screened all the records and four reviewers (II, LMSG, LSM, PJ) screened 25% of references each. Due to the involvement of many reviewers, we conducted a pilot screening using 56 articles (2.5%), corresponding to the articles published from January 1, 2020 to search date (April 7-8, 2020), for verifying whether all reviewers used the same inclusion and exclusion criteria. We retrieved full-text copies of potentially eligible reports for further assessment. Six reviewers independently confirmed the eligibility: one reviewer (CS) examined all full-text copies and five reviewers (IB, II, LMSG, MMPS, SLM)

For peer review only

assessed 20% of references each. Disagreements were solved by consensus or by involving a third expert reviewer (RP).

Charting the data

We designed a data extraction form using Google® Forms (Online supplementary file 2). General study characteristics extracted were as follows: first author name, title of article, contact detail of corresponding author, year of publication and type of publication. In addition, for each trial design referred to in the paper, we collected information on its definition, methodology, statistical considerations, advantages, disadvantages, utility, gaps and examples of actual trials, which adopted the design. A list of trial designs, which were retrieved from two previously conducted systematic reviews (11,12), was included in the data extraction form to harmonise the names used to report the same trial design. This initial list of trial designs was used as starting point to classify the identified trial designs and then modified and expanded on based on the results obtained in the present scoping review.

Two reviewers (CS, FBB) piloted and refined the data extraction form using three reviews (4%). Since many narrative reviews were already published about trial designs applied to personalised medicine, the data extraction was conducted in two phases. Firstly, two reviewers (CS, FBB) independently extracted data from the identified systematic and narrative reviews. Secondly, three reviewers (CS, FBB, MC) working independently extracted data for all the remaining selected records only if they provided new information, which was not extracted in the previous phase. One reviewer (FBB) extracted data for all records and two reviewers (CS, MC) extracted 60% and 40% of articles, respectively. Disagreements were solved by consensus or by involving a third expert reviewer (RP).

It was not within the remit of this scoping review to assess the methodological quality of individual studies included in the analysis.

Collating, summarising and reporting results

We summarised the extracted data in tables and figures. Information on the definition, methodology, statistical considerations, advantages, disadvantages, utility and gaps of trial designs was extracted verbatim. Data on the examples of clinical trials adopting the different approaches were summarised using frequencies and percentages.

A researcher (CS) listed all study designs and identified the main features for each of them, which were grouped into feature domains. The initial list was reviewed by a senior statistician with expertise in designing clinical trials (RP). A final list was created and agreed on with members of the PERMIT steering committee and co-authors of the present study.

New classification of trial designs in personalised medicine

Based on the identified trial designs and features, we proposed a new classification of trial designs for personalised medicine. Other attempts in classifying trial designs applied to personalised medicine have been proposed in the literature. However, they were limited to classifying the designs into categories (2,4,13) or identifying the design based on a specific feature (e.g., adaptive or non-adaptive trials) (11,12). This new classification goes a step further, proposing a new approach in classifying the trial designs considering two variables, which are core designs and design features, into a double-entry table.

For peer review only

Consultation exercise

The members of the PERMIT consortium, associated partners, and the PERMIT project Scientific Advisory Board discussed the preliminary findings of the scoping review in a 2-hour online workshop. A first version of the classification of the trial designs in personalised medicine was presented and discussed.

Patient and public involvement

The European Patients' Forum is a member of PERMIT project. Although not directly involved in the conduction of the scoping review, they received the draft review protocol for collecting comments and feedback.

Results

Study selection and general characteristics of reports

We retrieved 2350 citations from the electronic search and after removing the duplicates, 2301 remained. We excluded 1841 records based on titles and abstracts. After full-text assessment, 290 publications were excluded, and 167 met the inclusion criteria (see flow chart in Figure 1 and online supplementary file 3). From these 167 publications, we identified 6 systematic reviews, 69 narrative reviews, 8 original research articles, 26 methodological studies, 4 study protocols, 37 conference abstracts, 4 commentaries, 2 discussion papers, 3 reports, 1 book chapter, 1 editorial, 1 guidance document, and 5 links about trial registration (e.g., clinicaltrials.gov).

Trial designs and core designs in personalised medicine

We identified 23 trial designs, 9 sub-types, and 30 variations of trial designs applied to personalised medicine (Table 1). Information on the definition, methodology, and statistical considerations of identified trial designs are reported on the online supplementary file 4.

We classified the trial designs into four core categories named as *Master protocols*, *Randomise-all, Biomarker-strategy,* and *Enrichment*. Building on the definitions provided by Tajik et al. (13) and Park et al. (4), we defined the four core categories as:

- *Master protocols*: trial design, which includes multiple parallel sub studies under a common infrastructure.
- *Randomise-all*: trial design where eligible patients, irrespective of their biomarker status, are randomised to either an experimental or control treatment. This category also includes those hybrid designs, which first use a *Randomise-all* design, and then only a specific biomarker defined subgroup is randomised to either an experimental or control treatment.
- *Biomarker-strategy*: trial design where eligible patients are randomised to either a maker-based treatment strategy or non-marker-based treatment strategy.
- *Enrichment*: trial design where eligibility is determined according to the biomarker status and patients are then randomised to either an experimental or control treatment. A specific biomarker defined subgroup (usually biomarker positives) is believed to benefit more from a treatment compared to the other subgroup (usually biomarker negatives).

An example of a study design for each core category, including its definition and methodology used, is shown in Box 2. Overall, we identified 5 trial designs, 6 sub-types and 7 variations for

For peer review only

Master protocols, and 11 trial designs, 3 sub-types and 20 variations for *Randomise-all*, 5 trial designs for *Biomarker-strategy* and 2 trial designs and 3 variations for *Enrichment.*

From the identified designs, we found 29 main features of trial designs in personalised medicine, which were clustered into 10 features domains (Table 2). The features concern different aspects of a study design such as framework, model, control group, randomisation, biomarker assessment and adaptive aspects. A new classification of the trials designs for personalised medicine has been proposed and is reported in Table 3. The classification is presented in a double entry table, which includes the main trial features on the y-axis and core categories of the trial designs on the x-axis.

General characteristics of clinical trials in personalised medicine

We found 132 clinical trials, which used the identified designs (Online supplementary file 5). Table 4 presents the general characteristics of the identified trials.

Most trials used a basket (35/132, 26.5%), umbrella (29/132, 22.0%), platform (18/132, 13.6%) or marker stratified (15/132, 11.4%) design. The great majority of the trials were in the field of oncology (114/132, 86.4%). At the time of writing (March 2021), the recruitment status was on going for 47.7% (63/132) of the trials. A trial (0.8%) was not registered and seven (5.3%) presented an unknown status (meaning that the trial status has not been verified within the past two years on the clinicaltrials.gov website). Out of 132, 76 (57.6%) trials were phase II studies. For five trial designs, we did not find any examples of current applications.

Trial designs for assessing personalised versus non-personalised strategy

We identified 16 trials (16/132, 12.1%) evaluating a personalised vs. a non-personalised medicine strategy, which used nine different study designs (see online supplementary file 6).

Three trials used a biomarker design with a biomarker assessment in the control group (11,14,15). This study design consists of first testing the marker status of the entire study population and then randomises the patients either to a biomarker-based strategy arm or a non-biomarker strategy arm (11). In the GILT docetaxel trial (NCT00174629), patients with advanced non-small-cell lung cancer (NSCLC) were randomly assigned to either the control arm receiving a standard therapy of docetaxel plus cisplatin or the genotypic arm in which patients with low ERCC1 levels received docetaxel plus cisplatin and those with high levels received docetaxel plus gemcitabine. In the LIFT trial (NCT02498977), liver transplant recipients were randomised to either non-biomarker-based immunosuppression (IS) weaning or a biomarker-based IS weaning. ERCC1 gene expression was assessed in patients with NSCLC, which were then randomised to either to platinum therapy or non-platinum therapy in the ERCC1 trial (NCT00801736).

Four trials used a biomarker strategy design without biomarker assessment in the control arm (11,16–18). This design only evaluates the biomarker status in patients who are assigned to the biomarker-based strategy (11). Patients were randomised to either the NT-pro-BNP-guided therapy or usual care in the GUIDE-IT trial (NCT01685840) and either an algorithm driven individualized hemodynamic goal-directed therapy or standard care in the iPEGASUS trial (NCT03021525). Patients with mild head injury were randomly assigned to computed tomography or observation in the hospital in the OCTOPUS trial (ISRCTN81464462) and children with a doctor's diagnosis of asthma were randomised to a personalised medicine genotype-guided treatment arm or to usual care, nongenotype-guided, control arm in the PUFFIN trial (NCT03654508).

A modified strategy design, which differs from the previous strategy designs in including multiple targeted molecular profiles (19), was used in two trials (19–22). Patients with refractory cancer in the SHIVA trial (NCT01771458) were randomised to receive a molecularly targeted therapy based on metastasis molecular profiling or a conventional chemotherapy. In the NCI-MPACT trial

For peer review only

(NCT01827384), patients with an actionable mutation of interest (aMOI) were assigned to a targeted therapy based on mutation status or a therapy, chosen from the four regimes, not targeting the aMOI. We found that these two trials were also labelled as basket trials (23–25) as well as platform trial in the case of the SHIVA trial (26).

A trial used an adaptive strategy design for biomarkers with measurement error (22). This design is used when a second cheaper biomarker exists and may be concordant with a more expensive one, which is considered the gold standard. This design was used with some modifications in the OPTIMA trial (ISRCTN42400492). Oestrogen receptor-positive, HER-2 negative breast cancer patients were randomised to be either in the control arm receiving the standard care (i.e., chemotherapy and endocrine therapy) or in the treatment arm receiving the marker-guided therapy (i.e., endocrine therapy). Patients in the treatment arm, which obtained a high-risk test, also received chemotherapy.

The Siyaphambili Study (NCT03500172) used a sequential multiple assignment randomised (SMART) design to compare an individualised intervention (i.e., peer-led, individualised case management) to standard care (i.e., nurse-led mobile decentralised treatment programs) in women living with HIV (27). The SMART design allows comparing adaptive treatment strategies (ATSs), which consist of a series of tailored therapies during the course of a treatment (28).

ProBio (NCT03903835) used an outcome-randomization adaptive design to investigate whether a treatment based on molecular biomarker signature is more effective than standard care in men with metastatic castrate-resistant prostate cancer.

Finally, we found four trials, which evaluated a personalised versus a non-personalised strategy using a master protocol design (29–32). IMPACT II (NCT02152254) used a basket design and UPSTREAM (NCT03088059), SAFIR02_Breast (NCT02299999) and SAFIR02_Lung (NCT02117167) an umbrella design.

Gaps in the current research on clinical trials applied to personalised medicine

The results of this scoping review also allowed us to identify some gaps in the current research on clinical trials in personalised medicine. We identified three main gaps, which concern 1) the terminology used in labelling trial designs applied to personalised medicine, 2) the applications of complex innovative trial designs to fields outside of oncology and 3) the implementation of trials for evaluating personalised medicine strategy vs. non-personalised strategy.

We found that trial designs are often labelled in different ways or mislabelled. An example is the *Marker stratified design*, which was named using 18 different labels (see Table 1). We also found that a study design adopted in a clinical trial was defined differently across the literature. For instance, the I-SPY 2 trial (NCT01042379) has been labelled as outcome-based adaptive randomisation (12), platform (33) or umbrella design (34). The I-SPY 2 is an on-going platform trial, which studies multiple therapies in the context of breast cancer in a perpetual manner with arms being added or dropped based on current knowledge and collected data. Moreover, the study design adopted in the I-SPY 2 trial includes Bayesian adaptation algorithms in order to make decisions on estimated posterior probabilities, which are calculated at frequent interim-analysis points and response-adaptive randomisation (5). According to the new proposed classification, I-SPY 2 trial would be classified as *Master protocol* because it includes multiple sub studies under the same framework, with common/shared control group, early stopping, interim analysis and outcome-based adaptive randomisation as main design features.

Moreover, another gap in the current research on personalised medicine is the lack of application of novel complex study designs to fields outside of oncology. We found that 94% (80/85) of the clinical trials which used a master protocol design were in the field of oncology.

For peer review only

Finally, a strong need exists for clinical trials evaluating the effectiveness of a personalised medicine strategy vs. non-personalised strategy. This constitutes the third gap that we identified by mapping the evidence on clinical trials applied to personalised medicine. We found only 16 trials using nine different trial designs, which compared the two strategies.

Discussion

The present study provides a broad overview and proposes a new classification of the trial designs applied to personalised medicine.

The scoping review approach was considered to be the most suitable to respond to the broad scope of the field. Compared to systematic reviews that aim to answer specific questions, scoping reviews are used to present a broad overview of the evidence pertaining to a topic and they are useful to examine areas that are emerging, to clarify key concepts and identify gaps (35,36).

To our knowledge, this is the first study, which systematically reviews all trial designs, including complex innovative designs (i.e., basket, umbrella and platform), applied to personalised medicine. Other systematic reviews have been performed on specific trial designs such as biomarker-guided adaptive trial designs (12), biomarker-guided non-adaptive trials designs (11) and master protocols (4) or without considering master protocols in the search strategy (13).

We identified 23 trial designs, 9 sub-types, and 30 variations of trial designs applied to personalised medicine, which have been classified into four core categories: *Master protocols*, *Randomise-all*, *Biomarker strategy* and *Enrichment*. *Randomise-all* encompasses the largest number of trial designs (i.e., 11 trial designs, 3 sub-types and 20 variations) and *Master protocols* includes those study designs which are more frequently used in clinical trials (85/132, 64.4%).

From the different approaches applied to personalised medicine, we identified 29 main features, which were combined with the four core categories in a double entry table. The proposed table constitutes a novel manner to classify trial designs applied to personalised medicine.

Due to the variety and diversity of trial designs currently available in personalised medicine, the proposed classification permits to classify a trial design considering its corresponding core category and main features (e.g., central or accessory adaptive aspects). Also, it permits to consider all the relevant features associated with a trial design reducing confusion in reporting and labelling. We believe that this classification is more accurate and appropriate for describing a trial design applied to personalised medicine in its complexity. Moreover, it could help researchers and clinicians in using a harmonised terminology for labelling a trial.

Based on the results obtained, we identified three main gaps in the current research on clinical trials applied to personalised medicine. We found that more research is needed to evaluate the efficiency of personalised medicine approach vs. non-personalised standard of care and apply trial designs to fields outside of oncology. This last result was consistent with what was found in a recent systematic review of master protocols (4). The review showed that the great majority of basket, umbrella and platform studies (76/83, 91.6%) were conducted in the field of oncology. In particular, no umbrella trials were found outside of oncology. Finally, in line with two previous systematic reviews (2,13), we found that a harmonised terminology was required because it would permit increase clarity among the variety of trial designs applied to personalised medicine.

The present study has strengths but also limitations. This is the first scoping review, which presents an overview of all trial designs applied to personalised medicine. We followed a systematic approach to map the evidence and described the process using the PRISMA-ScR guideline. However, we restricted the search strategy to the last 15 years proving a comprehensive overview rather than an exhaustive list of trial designs used in personalised medicine. Although we

For peer review only

conducted a pilot screening for verifying the use of the same inclusion and exclusion criteria among reviewers, we cannot exclude that we did not identify some relevant publications. The information on the definition, methodology, statistical considerations, advantages, disadvantages, utility and gaps of trial designs was extracted verbatim from the included records. However, the selection of this information could be affected by the perception of the three reviewers who conducted the data extraction. Also, even if we built on existing reviews (11,12) and carefully developed a comprehensive classification, all attempts at categorisation are reductive in nature, and different classification schemes could be proposed. Nonetheless, our proposal allows separating between core design features that characterise the main objective of the trial and the patient flow, important aspects of the trial, and more accessory design features. It may form the basis of the evaluation of which design, and which features would be best suited for a given situation.

This review is the first step for the development of new recommendations on the use of trial designs applied to personalised medicine and on trials assessing personalised versus non-personalised medicine strategy. These recommendations are strongly needed to conduct new studies within the context of personalised medicine and, consequently, have new direct high-quality evidence in the evaluation of co-dependent personalised medicine technologies (37).

The information extracted on the pros and cons of each approach will be subject of further analysis and will be publish in a separate study due to considerable volume of information collected. We will also explore the pros and cons of each approach in more detail, together with experts from academia and regulatory agencies, when preparing the recommendations on the use of trial designs applied to personalised medicine.

Conclusions

The findings of this scoping review show that several trial designs exist applied to personalised medicine, which have been grouped into four core categories. A new classification has been proposed that allows describing trial designs taking into account their corresponding core category and main features.

For peer review only

Acknowledgments

The authors thank Vanna Pistotti for her assistance with search strategy development and conduction and Ines Bouajila for collaborating to the screening process.

List of Figures

Figure 1: Study selection flow diagram

References

1. Heckman-Stoddard BM, Smith JJ. Precision Medicine Clinical Trials: Defining New Treatment Strategies. Semin Oncol Nurs. 2014 May;30(2):109–16.

2. Janiaud P, Serghiou S, Ioannidis JPA. New clinical trial designs in the era of precision medicine: An overview of definitions, strengths, weaknesses, and current use in oncology. Cancer Treat Rev. 2019 Feb;73:20–30.

3. Clinical Trials Facilitation and Coordination Group. Recommendation Paper on the Initiation and Conduct of Complex Clincial Trials. 2019.

4. Park JJH, Siden E, Zoratti MJ, Dron L, Harari O, Singer J, et al. Systematic review of basket trials, umbrella trials, and platform trials: a landscape analysis of master protocols. Trials. 2019 Dec;20(1):572.

5. Woodcock J, LaVange LM. Master Protocols to Study Multiple Therapies, Multiple Diseases, or Both. Drazen JM, Harrington DP, McMurray JJV, Ware JH, Woodcock J, editors. N Engl J Med. 2017 Jul 6;377(1):62–70.

6. Medical Research Council. The MRC Framework for the Development, Design and Analysis of Stratified Medicine Research. Swindon,UK; 2018. Report No.: ISBN: 978-0-903730-28-0.

7. Joanna Briggs Institute. The Joanna Briggs Institute reviewers' manual 2015. Methodol JBI Scoping Rev Joanna Briggs Inst JBI; 2015.

8. Banzi R, Gerardi C, Fratelli M, Garcia P, Torres T, Haro Abad JM, et al. Methodological approaches for personalised medicine: protocol for a series of scoping reviews. 2020 Apr; 10.5281/zenodo.3770937

9. Tricco AC, Lillie E, Zarin W, O'Brien KK, Colquhoun H, Levac D, et al. PRISMA Extension for Scoping Reviews (PRISMA-ScR): Checklist and Explanation. Ann Intern Med. 2018 Oct 2;169(7):467.

10. Ouzzani M, Hammady H, Fedorowicz Z, Elmagarmid A. Rayyan—a web and mobile app for systematic reviews. Syst Rev. 2016 Dec;5(1):210.

11. Antoniou M, Kolamunnage-Dona R, Jorgensen A. Biomarker-Guided Non-Adaptive Trial Designs in Phase II and Phase III: A Methodological Review. J Pers Med. 2017 Jan 25;7(1):1.

12. Antoniou M, Jorgensen AL, Kolamunnage-Dona R. Biomarker-Guided Adaptive Trial Designs in Phase II and Phase III: A Methodological Review. Soyer HP, editor. PLOS ONE. 2016 Feb 24;11(2):e0149803.

13. Tajik P, Zwinderman AH, Mol BW, Bossuyt PM. Trial Designs for Personalizing Cancer Care: A Systematic Review and Classification. Clin Cancer Res. 2013 Sep 1;19(17):4578–88.

14. Clinicaltrials.gov. Liver Immunosuppression Free Trial (LIFT) [Internet]. Available from: https://clinicaltrials.gov/ct2/show/NCT02498977

15. Freidlin B, McShane LM, Korn EL. Randomized Clinical Trials With Biomarkers: Design Issues. JNCI J Natl Cancer Inst. 2010 Feb 3;102(3):152–60.

16. Ahmad T, O'Connor CM. Therapeutic Implications of Biomarkers in Chronic Heart Failure. Clin Pharmacol Ther. 2013 Oct;94(4):468–79.

17. Funcke S, Saugel B, Koch C, Schulte D, Zajonz T, Sander M, et al. Individualized, perioperative, hemodynamic goal-directed therapy in major abdominal surgery (iPEGASUS trial): study protocol for a randomized controlled trial. Trials. 2018 Dec;19(1):273.

18. Vijverberg SJ, Pijnenburg MW, Hövels AM, Koppelman GH, Maitland-van der Zee A-H. The need for precision medicine clinical trials in childhood asthma: rationale and design of the PUFFIN trial. Pharmacogenomics. 2017 Mar;18(4):393–401.

19. Renfro LA, An M-W, Mandrekar SJ. Precision oncology: A new era of cancer clinical trials. Cancer Lett. 2017 Feb;387:121–6.

20. Dienstmann R, Rodon J, Tabernero J. Optimal design of trials to demonstrate the utility of genomically-guided therapy: Putting Precision Cancer Medicine to the test. Mol Oncol. 2015 May;9(5):940–50.

21. Paoletti X, Asselain B, Kamal M, Servant N, Huppé P, Bieche I, et al. Design and statistical principles of the SHIVA trial. Chin Clin Oncol. 2015;4(3):1–10.

22. Renfro LA, Mallick H, An M-W, Sargent DJ, Mandrekar SJ. Clinical trial designs incorporating predictive biomarkers. Cancer Treat Rev. 2016 Feb;43:74–82.

23. Coyne GO, Takebe N, Chen AP. Defining precision: The precision medicine initiative trials NCI-MPACT and NCI-MATCH. Curr Probl Cancer. 2017 May;41(3):182–93.

24. Gómez-López G, Dopazo J, Cigudosa JC, Valencia A, Al-Shahrour F. Precision medicine needs pioneering clinical bioinformaticians. Brief. Bioinformatics. 2017:1–15.

25. Simon R. New designs for basket clinical trials in oncology. J Biopharm Stat. 2018 Mar 4;28(2):245–55.

26. Leonetti A, Boyd L, Giuliani J, Giovannetti E, Garajová I. Light and shadow on innovative clinical trial designs: reflections from the EORTC-PAMM course on 'preclinical and early-phase clinical pharmacology.' Expert Rev Clin Pharmacol. 2019 Nov 2;12(11):1033–6.

27. Clinicaltrials.gov. HIV Treatment Retention Interventions for Women Living With HIV (Siyaphambili Study) [Internet]. Available from: https://clinicaltrials.gov/ct2/show/NCT03500172

28. Kidwell KM, Wahed AS. Weighted log-rank statistic to compare shared-path adaptive treatment strategies. Biostatistics. 2013 Apr 1;14(2):299–312.

29. Lindberg J, De Laere B, Crippa A, Eklund M, Grönberg H. ProBio: An outcome-adaptive, multi-arm, open-label, multiple assignment randomised controlled biomarker-driven trial in patients with metastatic castration-resistant prostate cancer. Ann Oncol. 2019 Oct;30:v354

30. Galot R, Le Tourneau C, Saada-Bouzid E, Daste A, Even C, Debruyne PR, et al. A phase II study of monalizumab in patients with recurrent/metastatic (RM) squamous cell carcinoma of the head and neck (SCCHN): Results of the I1 cohort of the EORTC-HNCG-1559 trial (UPSTREAM). Ann Oncol. 2019 Oct;30:v449–50.

31. Ruiz-Garcia E, Astudillo-de la Vega H, editors. Translational Research and Onco-Omics

For peer review only

Applications in the Era of Cancer Personal Genomics [Internet]. Cham: Springer International Publishing; 2019 [cited 2021 Mar 8]. (Advances in Experimental Medicine and Biology; vol. 1168). Available from: http://link.springer.com/10.1007/978-3-030-24100-1

32. Park JJH, Hsu G, Siden EG, Thorlund K, Mills EJ. An overview of precision oncology basket and umbrella trials for clinicians. CA Cancer J Clin. 2020 Mar;70(2):125–37.

33. Talisa VB, Yende S, Seymour CW, Angus DC. Arguing for Adaptive Clinical Trials in Sepsis. Front Immunol. 2018 Jun 28;9:1502.

34. Tao JJ, Schram AM, Hyman DM. Basket Studies: Redefining Clinical Trials in the Era of Genome-Driven Oncology. Annu Rev Med. 2018 Jan 29;69(1):319–31.

35. Pham MT, Rajić A, Greig JD, Sargeant JM, Papadopoulos A, McEwen SA. A scoping review of scoping reviews: advancing the approach and enhancing the consistency. Res Synth Methods. 2014 Dec;5(4):371–85.

36. Munn Z, Peters MDJ, Stern C, Tufanaru C, McArthur A, Aromataris E. Systematic review or scoping review? Guidance for authors when choosing between a systematic or scoping review approach. BMC Med Res Methodol. 2018 Dec;18(1):143.

37. European Network for Health Technology Assessment. Personalised medicine and co-dependent technologies, with a special focus on issues of study design [Internet]. 2015. Available from: https://eunethta.eu/wp-content/uploads/2018/03/Personalized_Medicine_2016-03-07_reflection_paper_pm_2nd_draft.pdf

38. Council of the European Union. Notices from European Union Institutions, bodies, offices and agencies. OJEU. 2015;C 421/2-C 421/5.

39. Simon R. Critical Review of Umbrella, Basket, and Platform Designs for Oncology Clinical Trials: Review of umbrella, basket, and platform trial designs. Clin Pharmacol Ther. 2017 Dec;102(6):934–41.

For peer review only

Authors' contributions

Study conception and design: CG, CS, II, JDM, LSM, LMSG, PG, RB and RP

Methodology: CG, CS, RB

Data collection and analysis: CS, FBB, MCR, II, LSM and LMSG.

Trial design classification: CS and RP

Original draft preparation: CS

Review and editing: CG, II, LSM, LMSG, MCR, PG, RB and RP.

All authors read and approved the final version of the manuscript.

The members of the PERMIT group were involved in the preparation or revision of the joint protocol of the four scoping reviews of the PERMIT series, attended the joint workshop (consultation exercise) or contributed to one of the other scoping reviews of the PERMIT series.

PG and JDM coordinate the PERMIT project. JDM obtained funding.

Collaborators

PERMIT Group: Antonio L Andreu, Florence Bietrix, Maria del Mar Polo-de Santos, Jacques Demotes-Mainard, Maddalena Fratelli, Vibeke Fosse, Paula Garcia, Enrico Glaab, Rainer Girgenrath, Alexander Grundmann, Josep Maria Haro, Frank Hulstaert, Iñaki Imaz-Iglesia, Pascale Jonckheer, Setefilla Luengo Matos, Emmet McCormack, Anna Monistrol Mula, Albert Sanchez Niubo, Emanuela Oldoni, Teresa Torres.

Funding statement

This project has received funding from the European Union's Horizon 2020 research and innovation programme under grant agreement No. 874825.

Competing interests statement

None declared

Patient consent

Not required

Data sharing statement

The datasets supporting the conclusions of the present study will be available in the Zenodo repository.

For peer review only

Table 1. Trial designs applied to personalised medicine

Trial designs	Sub-type of trial designs	Variations	Core designs
**Marker stratified design**<br>1) Marker-stratified design<br>2) Biomarker-stratified design<br>3) Stratified-Randomised design<br>4) Stratification design<br>5) Stratified design<br>6) Stratified Analysis design<br>7) Marker by treatment – interaction design<br>8) Marker-by-treatment interaction design<br>9) Treatment by marker interaction design<br>10) Treatment-by-marker interaction design<br>11) Marker x treatment interaction design<br>12) Treatment-marker interaction design<br>13) Biomarker-by-treatment interaction design<br>14) Non-targeted RCT (stratified by marker) design<br>15) Genomic Signature stratified designs<br>16) Signature-Stratified design<br>17) Randomisation or analysis stratified by biomarker status design<br>18) Marker-interaction design	**Subgroup specific design**	**Sequential-subgroup specific design**<br>1) Sequential design<br>2) Sequential testing<br>3) Fixed-sequence 2 design<br>4) Hierarchical fixed sequence testing procedure	Randomise-all
		**Parallel-subgroup specific design**<br>1) Phase III biomarker-stratified design	Randomise-all
	**Biomarker-positive and overall strategies**	**Biomarker-positive and overall strategies with parallel assessment**<br>1) Overall/biomarker-positive design with parallel assessment<br>2) Prospective subset design<br>3) Hybrid design[1]	Randomise-all
		**Biomarker-positive and overall strategies with sequential assessment**<br>1) Overall/biomarker-positive design with sequential assessment<br>2) Sequential design<br>3) Fixed-sequence 2 design<br>4) Hierarchical fixed sequence testing procedure	Randomise-all
		**Biomarker-positive and overall strategies with fall-back analysis**<br>1) Biomarker-stratified design with fall-back analysis<br>2) Fall-back design<br>3) Prospective subset design<br>4) Sequential design<br>5) Other analysis plan design<br>6) Fallback design	Randomise-all
		**Marker sequential test design**<br>1) MaST design<br>2) Hybrid design[1]	Randomise-all
		**Auxiliary variable–enriched biomarker-stratified design (AEBSD)**	Randomise-all
**Hybrid design**<br>1) Mixture design<br>2) Combination of trial designs<br>3) Hybrid biomarker design			Randomise-all

For peer review only

**Biomarker strategy design with biomarker assessment in the control arm**<br>1) Marker strategy design<br>2) Biomarker-strategy design<br>3) Strategy design<br>4) Marker-based strategy design<br>5) Marker-based design<br>6) Random disclosure design<br>7) Customized strategy design<br>8) Parallel controlled pharmacogenetic study design<br>9) Marker-based strategy design I<br>10) Biomarker-guided design<br>11) Biomarker-based assignment of specific drug therapy design<br>12) Marker-based strategy I design<br>13) Biomarker-strategy design with a standard control<br>14) Marker strategy design for prognostic biomarkers			Biomarker-strategy
**Biomarker strategy design without biomarker assessment in the control arm**<br><br>1) Biomarker-strategy design with standard control<br>2) Direct-predictive biomarker-based<br>3) RCT of testing<br>4) Test-treatment<br>5) Parallel controlled pharmacogenetic diagnostic study<br>6) Marker strategy<br>7) Marker-based with no randomisation in the non-marker-based arm<br>8) Classical<br>9) Marker-based strategy<br>10) Marker strategy design for prognostic biomarkers			Biomarker-strategy
**Biomarker strategy design with treatment randomisation in the control arm**<br><br>1) Biomarker-strategy design with a randomised control<br>2) Modified marker-based strategy design (for predictive biomarkers)<br>3) Biomarker-strategy design with randomised control<br>4) Marker-based design with randomisation in the non-marker-based arm<br>5) Marker-based strategy design II<br>6) Marker-strategy design<br>7) Augmented strategy design<br>8) Trial design allowing the evaluation of both the treatment and the marker effect			Biomarker-strategy

For peer review only

**Reverse marker based strategy**			Biomarker-strategy
**Modified biomarker strategy design**<br><br>1) Modified marker based strategy design			Biomarker-strategy
**Sequential Multiple Assignment Randomised Trial (SMART) design**			Randomise-all
**Adaptive biomarker design**<br><br>1) Biomarker adaptive design			Randomise-all
**Adaptive strategy for biomarker with measurement error**			Randomise-all
**Adaptive signature design**<br><br>1) Two-stage adaptive signature design<br>2) Adaptive two-stage design<br>3) Biomarker adaptive signature design		**Adaptive threshold design**<br><br>1) Biomarker adaptive threshold design	Randomise-all
		**Molecular signature design**	Randomise-all
		**Cross-validated adaptive signature design**	Randomise-all
		**Generalized adaptive signature design**	Randomise-all
		**Adaptive signature design with subgroup plots**	Randomise-all
**Outcome-based adaptive randomisation design**<br><br>1) Adaptive randomisation Bayesian adaptive<br>2) Bayesian adaptive randomisation<br>3) Combined dynamic multi-arm<br>4) Outcome-adaptive randomisation<br>5) Outcome-based Bayesian adaptive randomisation		**Bayesian covariate adjusted response-adaptive randomisation**	Randomise-all
		**Bayesian hierarchical model for response-adaptive randomised design**	Randomise-all
**Adaptive threshold sample-enrichment design**<br><br>1) Threshold sample-enrichment approach<br>2) Two-stage sample enrichment<br>3) Two stage sample-enrichment design strategy<br>4) Two-stages adaptive threshold enrichment design			Enrichment
**Adaptive patient enrichment design**<br><br>1) Adaptive accrual<br>2) Adaptive accrual based on interim analysis design<br>3) Adaptive enrichment<br>4) Adaptive modification of target population<br>5) Adaptive population enrichment<br>6) Two-stage adaptive design<br>7) Two stage adaptive accrual		**Modified Bayesian version of the two-stage design**<br><br>1) Two-Stage Bayesian design<br>2) Bayesian adaptive enrichment design	Enrichment
		**Multistage adaptive biomarker-directed targeted (MAT) design**	Enrichment

For peer review only

		**Run- in phase design**	Enrichment
**Adaptive parallel Simon two-stage design**<br><br>1) Biomarker-adaptive parallel two-stage<br>2) Adaptive parallel<br>3) Two-parallel Simon<br>4) Two-stage design		**Parashar design**	Randomise-all
**Multi-arm multi-stage design**<br><br>1) Adaptive biomarker-driven design<br>2) Adaptive analysis<br>3) Adaptive multi-stage designs<br>4) Multi-stage	**Seamless design**	**Two-stage adaptive seamless design**<br><br>1) Seamless Phase II/III designs<br>2) Adaptive Seamless<br>3) Phase II/III Adaptive design<br>4) Two-stage Adaptive Seamless design<br>5) Adaptive Seamless Phase II/III design	Randomise-all
		**Adaptive design for population selection using correlated time to event endpoints**	Randomise-all
		**Bayesian adaptive patient enrolment restriction (BAPER) approach**	Randomise-all
		**Bayesian subgroup based adaptive design (SUBA)**	Randomise-all
		**Group sequential design**	Randomise-all
**Stratified adaptive design**<br><br>1) Adaptive stratified design			Randomise-all
**Tandem two stage design**<br><br>1) Tandem two-step phase II trial<br>2) Tandem-two step trial (phase II)<br>3) Tandem two-step phase 2 trial design<br>4) Tandem two-step			Randomise-all
**Platform design**	**Open adaptive platform**	**Randomised, embedded multifactorial adaptive platform (REMAP)**	Master protocols
		**Bayesian Adaptive Platform Trial**	Master protocols
	**Closed platform**		Master protocols
**Basket design**	**Randomised basket design**		Master protocols

For peer review only

	**Non randomised basket design**		Master protocols
		**Bayesian basket design**	Master protocols
		**Sequential basket trial design with Bayesian monitoring rules**	Master protocols
		**Bayesian latent subgroup trial (BLAST) design**	Master protocols
		**Bayesian hierarchical adaptive design**	Master protocols
**Basket of basket design**			Master protocols
**Umbrella design**	**Randomised umbrella design**		Master protocols
	**Non randomised umbrella design**		Master protocols
		**Bayesian adaptive umbrella design**	Master protocols
**Umbrella-basket hybrid**			Master protocols

[1] "Marker sequential test design" and "Biomarker-positive and overall strategies with parallel assessment" are also named as "Hybrid design" in the literature, although they present a different trial design compared to what we meant as "Hybrid design"

For peer review only

Box. 1 Personalised medicine definition

What is Personalised Medicine?

According to the European Council Conclusion on personalised medicine for patients personalised medicine is 'a medical model using characterisation of individuals' phenotypes and genotypes (e.g. molecular profiling, medical imaging, lifestyle data) for tailoring the right therapeutic strategy for the right person at the right time, and/or to determine the predisposition to disease and/or to deliver timely and targeted prevention (38).

In the context of the Permit project, we applied the following common operational definition of personalised medicine research: a set of comprehensive methods, (methodological, statistical, validation or technologies) to be applied in the different phases of the development of a personalised approach to treatment, diagnosis, prognosis, or risk prediction. Ideally, robust and reproducible methods should cover all the steps between the generation of the hypothesis (e.g., a given stratum of patients could better respond to a treatment), its validation and pre-clinical development, and up to the definition of its value in a clinical setting (8).

For peer review only

Box 2. Examples of core categories

Core category	Study design example	Study design definition	Study design methodology
**Master protocols**	Platform	"A platform trial is a single histology randomized phase II clinical trial involving multiple biomarkers and multiple drugs. Rather than assuming that we know which drug is appropriate for which biomarker stratum, randomization among drugs is used in the platform trial." (39)	"Initially the treatments are randomized with equal weights to the patients of a stratum. As data accumulates, the randomization weights change to favour assignment of drugs with higher within-stratum response rates. The endpoint used must be observed early enough to enable adaption of randomization weights." (39)
**Randomise-all**	Biomarker-positive and overall strategies with fall-back analysis	"It evaluates both the treatment effect in the overall study population and in the biomarker-positive subgroup sequentially." (11)	"In the fall-back design, we first test the overall population using the reduced significance level α_1 and if the test is significant, we consider that the novel treatment is effective in the overall population; however, if the result is not significant then we test the treatment effect in the biomarker-positive subgroup using the level of significance $\alpha_2 = \alpha - \alpha_1$,where α is the overall significance level (Type I error rate). The significance levels α can be considered as one-sided or two-sided significance levels."(11)
**Biomarker strategy**	Biomarker-strategy design with treatment randomization in the control arm	"The biomarker-strategy design with treatment randomization in the control treatment is able to inform us about whether the biomarker-based strategy is better than not only the standard treatment but also better than the experimental treatment in the overall population." (11)	"Patients are first randomly assigned to either the biomarker-based strategy arm or to the non-biomarker-based strategy arm. Next, patients who are allocated to the non-biomarker-based strategy are again randomized either to the experimental treatment arm or to the standard treatment arm irrespective of their biomarker status. Patients who are allocated to the biomarker-based strategy and who are biomarker-positive are given the experimental treatment and patients who are biomarker-negative are given the control treatment." (11)
**Enrichment**	Adaptive threshold sample-enrichment design	"It is a two-stage design in a Phase III setting […] to adaptively modify accrual in order to broaden the targeted patient population." (12)	"At the interim analysis stage, the treatment effect of a sample of patients (n_1) from the biomarker-positive subset is estimated. If an improvement is seen in the experimental treatment arm which is greater than a pre-specified threshold value (i.e. the estimated treatment difference between the novel treatment arm and the control treatment arm for this subpopulation is greater than a threshold value c divided by the square root of the aforementioned sample size n_1) the trial continues with accrual of patients from the entire biomarker-positive subgroup and additional patients are also accrued from the biomarker-negative subpopulation; otherwise the trial is stopped for futility. At the end of the trial, the treatment effect is estimated for all subpopulations. Researchers should choose the sample size $n_1$so that a persuasive result can be reached when the first stage of the trial is completed." (12)

For peer review only

Table 2. Main features of trial designs applied to personalised medicine

Feature domains	Features
**Inference framework**	Bayesian
	Frequentist
**Model**[1]	Disease progression
	Longitudinal
	Hierarchical
**Control group**	Common/Shared[2]
	Contemporaneous[3]
	Historical[4]
**Randomisation**	With treatment randomisation
	Without treatment randomisation
**Randomisation in the non-biomarker based strategy arm**	With treatment randomisation
	Without treatment randomisation[5]
	Reverse[6]
**Subgroup specific**	Sequential subgroup specific[7]
	Parallel subgroup specific[8]
**Biomarker positive and overall strategies**[9]	With parallel assessment
	With sequential assessment
	With fall-back analysis
	Marker sequential test
**Biomarker assessment**	With biomarker assessment in the control arm
	Without biomarker assessment in the control arm
**Central adaptive aspects**	Adaptive enrichment
	Adaptive signature
	Seamless
**Accessory adaptive aspects**	Early stopping [10]
	Interim analysis [11]
	Outcome-based adaptive randomisation
	Sample size reassessment
	Threshold [13]

[1] Model used for analysis. A disease progression model takes into account the patient disease state and other patient baseline characteristics for charactering patient clinical outcome(s). Longitudinal model permits including in the analysis the partial information of patients who have not yet reached their final outcome at an interim analysis.

[2] A common/shared control group can be used in a trial design in which multiple treatments are being tested, instead of each treatment having its own control arm.

[3] If patients in the common/shared control group receive a 'Standard of care' that may change over time or the profile of the patients enrolled on the trial may change over time, a trial design can use a contemporaneous control group meaning that the comparison of treatment's effects may be restricted to those patients who were enrolled/randomised in the same period as those patients who were allocated to the treatment.

[4] If a comparison group is not available in the existing trial or sub-study or at the same time but in a different setting, a trial design can use a historical control consisted of a group of individuals treated in the past.

[5] Patients, which are randomly assigned to the non-biomarker-based strategy arm, receive the control treatment.

[6] Patients which are randomly assigned to reverse-based strategy receive the control treatment if they are biomarker-positive and the experimental treatment if they are biomarker-negative.

[7] Study designs testing the treatment effect first in the biomarker-positive subpopulation and if the result is positive in the biomarker-negative subgroup.

[8] Study designs testing the treatment effect in both biomarker-positive and biomarker negative subgroups simultaneously.

[9] Study designs testing the treatment effect in the entire study population and in the biomarker-positive subgroup separately.

[10] A trial arm or clinical trial is stopped early due to pre-specified rules related to treatment efficacy and safety risk.

[11] Interim analyses are pre-planned analyses, which use accumulating data in order to make an early decision or adaptation.

[12] A threshold is used to divide the population into 'biomarker positive' and 'biomarker negative'.

For peer review only

Table 3. Trial designs classification

Design features \ Core designs		Biomarker strategy	Enrichment	Master protocols	Randomise-all
**Framework**	Bayesian				
	Frequentist				
**Model**	Disease progression				
	Longitudinal				
	Hierarchical				
**Control group**	Common/shared				
	Contemporaneous				
	Historical				
**Randomisation**	With treatment randomisation				
	Without treatment randomisation				
**Randomisation in the non-biomarker based strategy arm**	With treatment randomisation				
	Without treatment randomisation				
	Reverse				
**Subgroup specific**	Sequential subgroup specific				
	Parallel subgroup specific				
**Biomarker positive and overall strategies**	With parallel assessment				

For peer review only

	With sequential assessment				
	With fall-back analysis				
	Marker sequential test				
**Biomarker assessment**	With biomarker assessment in the control arm				
	Without biomarker assessment in the control arm				
**Central adaptive aspects**	Adaptive enrichment				
	Adaptive signature				
	Seamless				
**Accessory adaptive aspects**	Early stopping				
	Interim analysis				
	Outcome-based adaptive randomisation				
	Sample size reassessment				
	Threshold				

For peer review only

Table 4. General characteristics of clinical trials in personalised medicine

Trial design	Clinical trial[1]	Recruitment status of clinical trial as for March 2021				Disease area		Phases					
		Ongoing	Completed	nf[2]	Unknown[3]	Cancer	No cancer	II	II/III	III	IV	n/a[4]	nf[2]
	n=132 (%)	n=63 (%)	n=61 (%)	n=1 (%)	n=7 (%)	n=114 (%)	n=18 (%)	n=76 (%)	n=13 (%)	n=28 (%)	n=2 (%)	n=12 (%)	n=1 (%)
**Adaptive biomarker design**	2 (1.5)	1 (1.6)	1 (1.6)	0 (0)	0 (0)	2 (1.8)	0 (0)	2 (2.6)	0 (0)	0 (0)	0 (0)	0 (0)	0 (0)
**Adaptive parallel Simon two-stage design**	1 (0.8)	0 (0)	1 (1.6)	0 (0)	0 (0)	1 (0.9)	0 (0)	1 (1.3)	0 (0)	0 (0)	0 (0)	0 (0)	0 (0)
**Adaptive patient enrichment design**	4 (3.0)	0 (0)	4 (6.6)	0 (0)	0 (0)	0 (0)	4 (22.2)	0 (0)	0 (0)	4 (14.3)	0 (0)	0 (0)	0 (0)
**Adaptive signature design**	0 (0)	0 (0)	0 (0)	0 (0)	0 (0)	0 (0)	0 (0)	0 (0)	0 (0)	0 (0)	0 (0)	0 (0)	0 (0)
**Adaptive strategy for biomarker with measurement error**	1 (0.8)	1 (1.6)	0 (0)	0 (0)	0 (0)	1 (0.9)	0 (0)	0 (0)	0 (0)	0 (0)	0 (0)	1 (8.3)	0 (0)
**Adaptive stratified design**	0 (0)	0 (0)	0 (0)	0 (0)	0 (0)	0 (0)	0 (0)	0 (0)	0 (0)	0 (0)	0 (0)	0 (0)	0 (0)
**Adaptive threshold sample-enrichment design**	0 (0)	0 (0)	0 (0)	0 (0)	0 (0)	0 (0)	0 (0)	0 (0)	0 (0)	0 (0)	0 (0)	0 (0)	0 (0)
**Basket**	35 (26.5)	19 (30.2)	13 (21.3)	0 (0)	3 (42.9)	34 (29.8)	1 (5.6)	32 (42.1)	0 (0)	2 (7.1)	0 (0)	1 (8.3)	0 (0)
**Basket of basket design**	1 (0.8)	1 (1.6)	0 (0)	0 (0)	0 (0)	1 (0.9)	0 (0)	1 (1.3)	0 (0)	0 (0)	0 (0)	0 (0)	0 (0)
**Biomarker strategy design with biomarker assessment in the control arm**	3 (2.3)	0 (0)	3 (4.9)	0 (0)	0 (0)	2 (1.8)	1 (5.6)	0 (0)	0 (0)	2 (7.1)	1 (50.0)	0 (0)	0 (0)
**Biomarker strategy design with treatment randomisation in the control arm**	0 (0)	0 (0)	0 (0)	0 (0)	0 (0)	0 (0)	0 (0)	0 (0)	0 (0)	0 (0)	0 (0)	0 (0)	0 (0)
**Biomarker strategy design without biomarker assessment in the control arm**	4 (3.0)	2 (3.2)	2 (3.3)	0 (0)	0 (0)	0 (0)	4 (22.2)	0 (0)	0 (0)	0 (0)	0 (0)	4 (33.3)	0 (0)
**Hybrid design**	1 (0.8)	0 (0)	1 (1.6)	0 (0)	0 (0)	1 (0.9)	0 (0)	0 (0)	0 (0)	1 (3.6)	0 (0)	0 (0)	0 (0)
**Marker stratified design**	15 (11.4)	0 (0)	14 (23.0)	1 (100)	0 (0)	15 (13.2)	0 (0)	0 (0)	0 (0)	14 (50.0)	0 (0)	0 (0)	1 (100.0)
**Modified biomarker strategy design**	3 (2.3)	0 (0)	2 (3.3)	0 (0)	1 (14.3)	3 (2.6)	0 (0)	2 (2.6)	0 (0)	1 (3.6)	0 (0)	0 (0)	0 (0)

For peer review only

**Multi-arm multi-stage design**	7 (5.3)	3 (4.8)	3 (4.9)	0 (0)	1 (14.3)	5 (4.4)	2 (11.1)	4 (5.3)	2 (15.4)	1 (3.6)	0 (0)	0 (0)	0 (0)
**Outcome-based adaptive randomisation design**	4 (3.0)	2 (3.2)	2 (3.3)	0 (0)	0 (0)	3 (2.6)	1 (5.6)	2 (2.6)	1 (7.7)	1 (3.6)	0 (0)	0 (0)	0 (0)
**Platform**	18 (13.6)	13 (20.6)	4 (6.6)	0 (0)	1 (14.3)	14 (12.3)	4 (22.2)	11 (14.5)	4 (30.8)	1 (3.6)	1 (50.0)	1 (8.3)	
**Reverse marker biased strategy**	0 (0)	0 (0)	0 (0)	0 (0)	0 (0)	0 (0)	0 (0)	0 (0)	0 (0)	0 (0)	0 (0)	0 (0)	0 (0)
**Sequential Multiple Assignment Randomised trial**	1 (0.8)	0 (0)	1 (1.6)	0 (0)	0 (0)	0 (0)	1 (5.6)	0 (0)	0 (0)	0 (0)	0 (0)	1 (8.3)	0 (0)
**Tandem two stage design**	1 (0.8)	0 (0)	1 (1.6)	0 (0)	0 (0)	1 (0.9)	0 (0)	1 (1.3)	0 (0)	0 (0)	0 (0)	0 (0)	0 (0)
**Umbrella**	29 (22.0)	19 (30.2)	9 (14.8)	0 (0)	1 (14.3)	29 (25.4)	0 (0)	18 (23.7)	6 (46.2)	1 (3.6)	0 (0)	4 (33.3)	0 (0)
**Umbrella-basket hybrid**	2 (1.5)	2 (3.2)	0 (0)	0 (0)	0 (0)	2 (1.8)	0 (0)	2 (2.6)	0 (0)	0 (0)	0 (0)	0 (0)	0 (0)

[1] If the same clinical trial was labelled differently across articles, we considered the trial as example of the design reported in the paper. For instance, I-SPY 2 has been labelled as outcome-based adaptive randomisation (12), platform (33) or umbrella design (34) and it was considered as an example for each of those trial designs.

[2] Not found.

[3] Unknown is used to indicate a trial status that has not been verified within the past two years on the Clinicaltrials.gov website.

[4] Not applicable is used on the Clinicaltrials.gov website to describe trials without FDA-defined phases including trials of devices or behavioural interventions.

For peer review only

PRISMA 2009 Flow Diagram

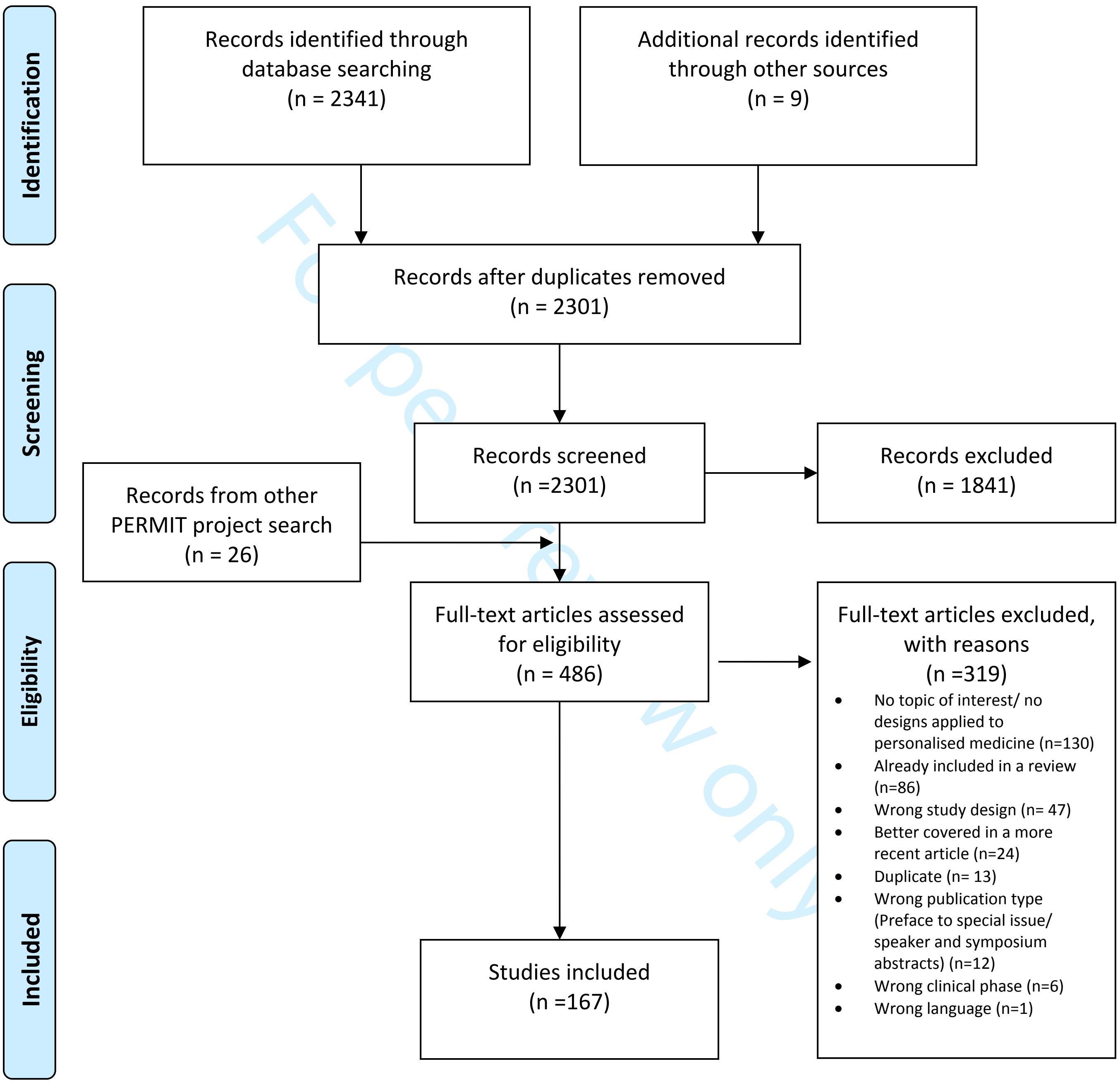

From: Moher D, Liberati A, Tetzlaff J, Altman DG, The PRISMA Group (2009). *P*referred *R*eporting *I*tems for *S*ystematic Reviews and *M*eta-*A*nalyses: The PRISMA Statement. PLoS Med 6(7): e1000097. doi:10.1371/journal.pmed1000097

For more information, visit www.prisma-statement.org.

For peer review only - http://bmjopen.bmj.com/site/about/guidelines.xhtml

Supplementary file I. Search strategies

Pubmed 7/4/2020

No.	Query	Results
#37	Search: #27 OR #30 Filters: English, French, German, Italian, Spanish Sort by: Publication Date	1221
#32	Search: #27 OR #30 Filters: from 2005 - 2020 Sort by: Publication Date	1232
#31	Search: #27 OR #30 Sort by: Publication Date	1277
#30	Search: #28 AND #29 Sort by: Publication Date	375
#29	Search: ("2019/09/01"[Date - Entry] : "3000"[Date - Entry]) Sort by: Publication Date	752605
#28	Search: #2 AND #25 AND (“clinical trial“ [tiab] OR “clinical trials“ [tiab]) Sort by: Publication Date	5359
#27	Search: #1 AND #2 AND #25 Sort by: Publication Date	918
#25	Search: design*[tiab] OR methods[ti] OR method[tiab] OR Research design[Majr] Sort by: Publication Date	3787147
#2	Search: “stratified medicine”[tiab] OR biomarker*[tiab] OR “precision medicine”[tiab] OR “personalized medicine”[tiab] OR “personalised medicine”[tiab] OR “individualized Medicine“[tiab] OR “individualised Medicine“[tiab] OR “individualized therapy“[tiab] OR “individualised therapy“[tiab] OR "Biomarkers"[Majr] OR "Precision Medicine"[Majr]	486778
#1	Search: “umbrella study“[tiab] OR “umbrella studies”[tiab] OR “umbrella trial“[tiab] OR “umbrella trials*“[tiab] OR “adaptive study“[tiab] OR “adaptive studies“[tiab] OR “adaptive trial“[tiab] OR “adaptive trials“[tiab] OR “ basket trial“[tiab] OR “basket trials“[tiab] OR “basket studies“[tiab] OR “basket study“[tiab] OR “multi arm“[tiab] OR “multi arms“[tiab] OR “master protocol“[tiab] OR “master protocols“[tiab]OR “platform study“[tiab] OR “platform studies“[tiab] OR “platform trial“[tiab] OR “platform trials“[tiab] OR"Clinical Trials as Topic"[Majr]	55630

For peer review only

Embase 7/4/202

No.	Query	Results
#14	#11 AND #12 AND ([english]/lim OR [french]/lim OR [german]/lim OR [italian]/lim OR [spanish]/lim)	927
#13	#11 AND #12	929
#12	[embase]/lim NOT [medline]/lim	9610086
#11	#7 OR #10	1221
#10	#4 AND #5 AND #8 AND [2020-2020]/py	202
#9	#4 AND #5 AND #8	7669
#8	'clinical trial*':ti,ab	514125
#7	#3 AND #4 AND #5 AND [2005-2020]/py	1026
#6	#3 AND #4 AND #5	1033
#5	design*:ti,ab OR methods:ti OR method:ti,ab	4793126
#4	'biological marker'/exp/mj OR 'personalized medicine'/exp/mj OR 'stratified medicine':ti,ab OR biomarker*:ti,ab OR 'precision medicine':ti,ab OR 'personalized medicine':ti,ab OR 'personalised medicine':ti,ab OR 'individualized medicine':ti,ab OR 'individualised medicine':ti,ab OR 'individualized therapy':ti,ab OR 'individualised therapy':ti,ab	431819
#3	#1 OR #2	52941
#2	'clinical trial'/exp/mj	50652
#1	'basket trial*':ti,ab OR 'basket stud*':ti,ab OR 'multi arm*':ti,ab OR 'master protocol*':ti,ab OR 'platform stud*':ti,ab OR 'platform trial*':ti,ab OR 'umbrella trial*':ti,ab OR 'adaptive stud*':ti,ab OR 'adaptive trial*':ti,ab OR 'umbrella stud*':ti,ab	2402

Cochrane Library 8/4/2020

No.	Query	Results
#1	'basket trial*':ti,ab OR 'basket stud*':ti,ab OR 'multi arm*':ti,ab OR 'master protocol*':ti,ab OR 'platform stud*':ti,ab OR 'platform trial*':ti,ab OR 'umbrella trial*':ti,ab OR 'adaptive stud*':ti,ab OR 'adaptive trial*':ti,ab OR 'umbrella stud*':ti,ab	22497

#2	'stratified medicine':ti,ab OR biomarker*:ti,ab OR 'precision medicine':ti,ab OR 'personalized medicine':ti,ab OR 'personalised medicine':ti,ab OR 'individualized medicine':ti,ab OR 'individualised medicine':ti,ab OR 'individualized therapy':ti,ab OR 'individualised therapy':ti,ab	29297
#3	design*:ti,ab OR methods:ti OR method:ti,ab	355698
#4	#1 and #2 and #3 with Publication Year from 2005 to 2020, in Trials	560
#5	"accession number" near pubmed	662135
#6	"accession number" near embase	536983
#7	#5 or #6	998271
#8	#4 not #7	193

For peer review only

Supplementary file II. Data extraction form

No	
First author:	
Title of article:	
Contact details of author:	
Publication year:	
Type of paper:	○ Original research article reporting a clinical trial<br>○ Study protocol<br>○ Methodological study<br>○ Methodological review<br>○ Systematic review<br>○ Conference abstract<br>○ Commentary<br>○ Letter to the editor<br>○ Clinicaltrial.gov link<br>○ Guidance document<br>○ Please specify the regulatory or health technologies assessment agency, which issued the report<br>○ Other (please specify): ____________________
Study design type:	○ Umbrella design<br>○ Basket design<br>○ Bayesian basket design<br>○ Basket of baskets design<br>○ Marker stratified design (part of randomize-all design. Marker stratified design includes 1) Marker sequential test design, 2) Biomarker-positive and overall strategies with fall-back analysis, 3) Biomarker-positive and overall strategies with sequential assessment, 4) Biomarker-positive and overall strategies with parallel assessment)<br>○ Hybrid design (part of randomize-all design)<br>○ Biomarker-strategy design with biomarker assessment in the control arm (part of biomarker-based strategy design)<br>○ Biomarker-strategy design without biomarker assessment in the control arm (part of biomarker-based strategy design)<br>○ Biomarker-strategy design with treatment randomization in the control arm (part of biomarker-based strategy design)<br>○ Reverse marker-based strategy design (part of biomarker-based strategy design)<br>○ Two-stage adaptive seamless design<br>○ Multi-arm multi-stage design (MAMS) (also called Platform design. It is an extension of 2-stage adaptive seamless design)<br>○ Adaptive signature design (also called Two-stage

For peer review only

	adaptive signature design, adaptive two-stage design, Biomarker-adaptive signature design)<br><br>○ Outcome-based adaptive randomization design (also called Adaptive randomization Bayesian adaptive, Bayesian adaptive randomization, Combined dynamic multi-arm, Outcome-Adaptive randomization, Outcome-based Bayesian adaptive randomization)<br><br>○ Adaptive threshold sample-enrichment design (also called Threshold sample-enrichment approach, two-stage sample enrichment, two-stage sample-enrichment design strategy)<br><br>○ Adaptive patient enrichment design (also called adaptive accrual, adaptive accrual based on interim analysis, adaptive enrichment, adaptive modification of target population, adaptive population enrichment, two-stage adaptive design, two stage adaptive accrual)<br><br>○ Adaptive parallel Simon two-stage design (also called pick-the-winner, biomarker-adaptive parallel two stage, adaptive parallel, two-parallel Simon, two-stage design)<br><br>○ Stratified adaptive design<br><br>○ Tandem two stage design (also called Tandem two-step phase II trial, tandem-two step trial (phase II), Tandem two-step phase 2 trial design, Tandem two-step)<br><br>○ Other (please specify): ____________________
**Definition** of the trial design referred to in the paper (if reported):	Please copy and paste the exact text.<br>E.g., The design begins with a comparison between the experimental treatment and the standard treatment in the entire study population at a pre-specified level of significance. In case that the overall result is positive, it is considered that the treatment is beneficial and the trial is closed. If the comparison in the overall population is not promising, then the entire population is divided in order to develop and validate a biomarker, using a split sample strategy. More precisely, a portion of patients is used to detect a biomarker signature that best distinguishes subjects for which the novel treatment is better than the standard treatment. Hence, this approach (i) identifies patients who are more susceptible to a specific treatment during the initial stage of the study (at the interim analysis); (ii) it assesses the global treatment effect of the entire randomized study population through a powered test, and (iii) finally, it assesses the treatment effect for the biomarker-positive subgroup identified during the initial stages of the study but only with patients randomized in the remainder of the trial, the so-called ‘validation test’.

For peer review only

**Methodology** of the trial design referred to in the paper (if reported):	Analysis	Please copy and paste the exact text.<br>E.g., The analysis is undertaken as follows: At the interim analysis stage, if the overall treatment effect is not significant at a reduced level a1 (< 0.05), the full set of P patients in the clinical trial is partitioned into a training set Tr and a validation set V. A pre-specified algorithmic analysis plan is applied to the training set to generate a classifier Cl(x;Tr) where x is a biomarker vector.
	Other (please specify):<br>____________________	Please copy and paste the exact text.
**Statistical considerations** of the trial design referred to in the paper (if reported):		Please copy and paste the exact text.<br>E.g., Although the adaptive signature design allows for approval of the novel treatment in a quick and efficient way, the main statistical challenges to be taken into account include the potential increase in the number of patients and the limited power to assess the treatment effect in the biomarker-defined subgroup. However, this approach avoids introduction of bias since the adaptations do not involve modifications in allocation ratio and eligibility criteria. Further, it prevents the inflation Type I error rate as the design does not use the study population which was employed to develop the predictive signature for the assessment of the treatment effect.
**Utility** of the trial design referred to in the paper (if reported):		Please list the reasons why it is recommended to use the study design by coping and pasting the exact text. Each point corresponds to a reason.<br>E.g., 1) In cases where we want to know whether the biomarker is not only prognostic but also predictive, this design is preferable.<br>○ ____________________<br>○ ____________________<br>○ ____________________<br>○ ____________________<br>○ ____________________<br>○ ____________________
**Advantages** of the trial design referred to in the paper (if reported):		Please list the advantages by coping and pasting the exact text. Each point corresponds to strength of the study design.<br>E.g., 1) Identification of optimal group of patients which benefit the most from a specific treatment; 2) Identification and validation of candidate biomarker in a single trial, etc.<br>○ ____________________<br>○ ____________________<br>○ ____________________<br>○ ____________________<br>○ ____________________<br>○ ____________________
**Disadvantages** of the trial design referred to in the paper (if reported):		Please list the disadvantages by coping and pasting the exact text. Each point corresponds to a limitation of the study design.<br>○ ____________________<br>○ ____________________<br>○ ____________________

For peer review only

	○ ______________________<br>○ ______________________<br>○ ______________________<br>○ ______________________
**Gaps** in the study design methodology to be addressed in future research (if reported):	Please list the gaps by coping and pasting the exact text. Each point corresponds to a gap of the study design.<br>○ ______________________<br>○ ______________________<br>○ ______________________<br>○ ______________________<br>○ ______________________<br>○ ______________________
Example of actual trial(s), which have adopted the design mentioned.	Please report the exact name of the trial (e.g., NCI-MATCH trial)
Current status of the trial(s):	○ Ongoing trial<br>○ Completed trial
Trial registration number:	Please report the number
Clinical field:	○ Cancer<br>▪ (please specify):______________________<br>○ No cancer<br>▪ (please specify):______________________
Type of intervention:	○ Pharmaceutical<br>○ Non pharmaceutical
Clinical trial phase	○ Phase II<br>○ Phase III
Eligibility criteria:	○ _______<br>○ _______
Patient subgroups:	○ _______<br>○ _______
Intervention(s):	○ _______<br>○ _______
Control group:	○ _______<br>○ _______
Primary outcome measure(s):	○ _______<br>○ _______
External validity:	○ _______<br>○ _______
Did the study assess a personalised vs. non-personalised strategy?	○ Yes<br>○ No
Other considerations related to the study design:	

For peer review only

Supplementary file III. Included studies

1	Aanur P, Gutierrez M, Kelly RJ, Ajani JA, Ku GY, Denlinger CS, et al. FRACTION (Fast Real-time Assessment of Combination Therapies in Immuno-Oncology)-gastric cancer (GC): A randomized, open-label, adaptive, phase 2 study of nivolumab in combination with other immuno-oncology (IO) agents in patients with advanced GC. J Clin Oncol. 2017;35:TPS4137	Conference abstract
2	Abrams J, Conley B, Mooney M, Zwiebel J, Chen A, Welch JJ, et al. National Cancer Institute's Precision Medicine Initiatives for the New National Clinical Trials Network. Am Soc Clin Oncol Educ Book. 2014 May;(34):71–6.	Narrative review
3	Ahmad T, O'Connor CM. Therapeutic Implications of Biomarkers in Chronic Heart Failure. Clin Pharmacol Ther. 2013 Oct;94(4):468–79.	Narrative review
4	Alexander BM, Ba S, Berger MS, Berry DA, Cavenee WK, Chang SM, et al. Adaptive Global Innovative Learning Environment for Glioblastoma: GBM AGILE. Clin Cancer Res. 2018 Feb 15;24(4):737–43.	Narrative review
5	Alexander BM, Lorenzo T. Bayesian baskets: A novel approach to biomarker-based clinical trial design. J Clin Oncol. 2016;34: e14057	Conference abstract
6	Alexander BM, Trippa L, Gaffey S, Arrillaga-Romany IC, Lee EQ, Rinne ML, et al. Individualized Screening Trial of Innovative Glioblastoma Therapy (INSIGhT): A Bayesian Adaptive Platform Trial to Develop Precision Medicines for Patients With Glioblastoma. JCO Precis Oncol. 2019 Dec;(3):1–13.	Original research article reporting a clinical trial
7	Antoniou M, Jorgensen AL, Kolamunnage-Dona R. Biomarker-Guided Adaptive Trial Designs in Phase II and Phase III: A Methodological Review. Soyer HP, editor. PLOS ONE. 2016 Feb 24;11(2):e0149803.	Systematic review
8	Antoniou M, Kolamunnage-Dona R, Jorgensen A. Biomarker-Guided Non-Adaptive Trial Designs in Phase II and Phase III: A Methodological Review. J Pers Med. 2017 Jan 25;7(1):1.	Systematic review
9	Antoniou M, Kolamunnage-Dona R, Wason J, Bathia R, Billingham C, Bliss JM, et al. Biomarker-guided trials: Challenges in practice. Contemp Clin Trials Commun. 2019 Dec;16:100493.	Discussion paper
10	Bang Y-J, Kaufman B, Geva R, Stemmer SM, Hong S-H, Lee J-S, et al. An open-label, phase II basket study of olaparib and durvalumab (MEDIOLA): Results in patients with relapsed gastric cancer. J Clin Oncol. 2019;37:140	Conference abstract
11	Barroilhet L, Matulonis U. The NCI-MATCH trial and precision medicine in gynecologic cancers. Gynecol Oncol. 2018 Mar;148(3):585–90.	Narrative review
12	Barry WT, Perou CM, Marcom PK, Carey LA, Ibrahim JG. The Use of Bayesian Hierarchical Models for Adaptive Randomization in Biomarker-Driven Phase II Studies. J Biopharm Stat. 2015 Jan 2;25(1):66–88.	Methodological study
13	Bateman RJ, Benzinger TL, Berry S, Clifford DB, Duggan C, Fagan AM, et al. The DIAN-TU Next Generation Alzheimer's prevention trial: Adaptive design and disease progression model. Alzheimers Dement. 2017 Jan;13(1):8–19.	Original research article reporting a clinical trial
14	Beckman R, Antonijevic Z, Kalamegham R, Chen C. Adaptive Design for a Confirmatory Basket Trial in Multiple Tumor Types Based on a Putative Predictive Biomarker. Clin Pharmacol Ther. 2016 Dec;100(6):617–25.	Methodological study
15	Bell S, Copel J, Smith A. The pros and cons of an "umbrella" trial design for a rare disease from a trial management and data management perspective. Trials 2017; 18(Suppl 1): 200	Conference abstract
16	Berry DA. The Brave New World of clinical cancer research: Adaptive biomarker-driven trials integrating clinical practice with clinical research. Mol Oncol. 2015 May;9(5):951–9.	Narrative review
17	Berry SM, Broglio KR, Groshen S, Berry DA. Bayesian hierarchical modeling of patient subpopulations: Efficient designs of Phase II oncology clinical trials. Clin Trials J Soc Clin Trials. 2013 Oct;10(5):720–34.	Methodological study
18	Blagden SP, Billingham L, Brown LC, Buckland SW, Cooper AM, Ellis S, et al. Effective delivery of Complex Innovative Design (CID) cancer trials—A consensus statement. Br J Cancer. 2020 Feb 18;122(4):473–82.	Guidance document
19	Bothwell LE, Avorn J, Khan NF, Kesselheim AS. Adaptive design clinical trials: a review of the literature and ClinicalTrials.gov. BMJ Open. 2018 Feb;8(2):e018320.	Systematic review

20	Bradbury P, Hilton J, Seymour L. Early-phase oncology clinical trial design in the era of molecularly targeted therapy: pitfalls and progress. Clin Investig. 2011 Jan;1(1):33–44.	Narrative review
21	Brana I, Massard C, Baird RD, Opdam F, Schlenk RF, De Petris L, et al. Basket of baskets (BoB): A modular, open label, phase II, multicenter study to evaluate targeted agents in molecularly selected populations with advanced solid tumors. J Clin Oncol. 2019; 37: TPS3151	Conference abstract
22	Buch MH, Pavitt S, Parmar M, Emery P. Creative trial design in RA: optimizing patient outcomes. Nat Rev Rheumatol. 2013 Mar;9(3):183–94.	Narrative review
23	Cabarrou B, Sfumato P, Leconte E, Boher JM, Filleron T. Designing phase II clinical trials to target subgroup of interest in a heterogeneous population: A case study using an R package. Comput Biol Med. 2018 Sep;100:239–46.	Methodological study
24	Cafferkey C, Chau I, Thistlethwaite F, Petty RD, Starling N, WatkinsSheela Rao D, et al. PLATFORM: Planning treatment of oesophago-gastric (OG) cancer randomised maintenance therapy trial. J Clin Oncol. 2016; 34: TPS187	Conference abstract
25	Cecchini M, Rubin EH, Blumenthal GM, Ayalew K, Burris HA, Russell-Einhorn M, et al. Challenges with Novel Clinical Trial Designs: Master Protocols. Clin Cancer Res. 2019 Apr 1;25(7):2049–57.	Discussion paper
26	Cerqueira FP, Jesus AMC, Cotrim MD. Adaptive Design: A Review of the Technical, Statistical, and Regulatory Aspects of Implementation in a Clinical Trial. Ther Innov Regul Sci. 2019 Feb 27;216847901983124.	Narrative review
27	Chen C, Li X (Nicole), Yuan S, Antonijevic Z, Kalamegham R, Beckman RA. Statistical Design and Considerations of a Phase 3 Basket Trial for Simultaneous Investigation of Multiple Tumor Types in One Study. Stat Biopharm Res. 2016 Jul 2;8(3):248–57.	Methodological study
28	Cheng A-L. Combining Adaptive Design and Omics for Future HCC Trials. Liver Cancer 2015. 4: 1-257	Conference abstract
29	Clinicaltrials.gov. HIV Treatment Retention Interventions for Women Living With HIV (Siyaphambili Study) [Internet]. Available from: https://clinicaltrials.gov/ct2/show/NCT03500172	Link
30	Clinicaltrials.gov. Liver Immunosuppression Free Trial (LIFT) [Internet]. Available from: https://clinicaltrials.gov/ct2/show/NCT02498977	Link
31	Clinicaltrials.gov. ProBio: A Biomarker Driven Study in Patients With Metastatic Castrate Resistant Prostate Cancer (ProBio) [Internet]. Available from: https://clinicaltrials.gov/ct2/show/NCT03903835	Link
32	Cochrane Library. Trial for the optimisation of risk assessment and therapy success predicition in patients with early breast cancer by the use of biomarkers in advance to therapy decission-making to personalize therapies [Internet]. Available from: https://www.cochranelibrary.com/central/doi/10.1002/central/CN-01873376/full	Link
33	Conter HJ, MacDonald LD, Fiset S, Bramhecha YM, Chaney M, Rosu GN. Safety and efficacy results of the combination of DPX-Survivac, pembrolizumab and intermittent low dose cyclophosphamide (CPA) in subjects with advanced and metastatic solid tumours: Preliminary results from the hepatocellular carcinoma (HCC), NSCLC, bladder cancer, & MSI-H cohorts. Ann Oncol. 2019 Oct;30:v494.	Conference abstract
34	Coyne GO, Takebe N, Chen AP. Defining precision: The precision medicine initiative trials NCI-MPACT and NCI-MATCH. Curr Probl Cancer. 2017 May;41(3):182–93.	Narrative review
35	D'Angelo S, Blay J, Chow W, Demetri G, Thistlethwaite FC, Wagner M, et al. Autologous T cells with NY-ESO-1-specific T-cell receptor (GSK3377794) in HLA-A*02+previously-treated and -untreated advanced metastatic/unresectable synovial sarcoma: A master protocol study design. Journal for Immunotherapy of Cancer. 2019;7:282	Conference abstract
36	De Mattos-Arruda L, Rodon J. Pilot Studies for Personalized Cancer Medicine: Focusing on the Patient for Treatment Selection. The Oncologist. 2013 Nov;18(11):1180–8.	Narrative review

For peer review only

37	Debily M-A, Kergrohen T, Varlet P, Le Teuff G, Nysom K, Blomgren K, et al. PDTM-36. Whole exome sequencing (WES) of DIPG patients from the BIOMEDE trial reveals new prognostic subgroups with specific oncogenis programmes. Neuro-Oncology 2019;21 (Suppl 6): vi195.	Conference abstract
38	Diao G, Dong J, Zeng D, Ke C, Rong A, Ibrahim JG. Biomarker threshold adaptive designs for survival endpoints. J Biopharm Stat. 2018 Nov 2;28(6):1038–54.	Methodological study
39	Dienstmann R, Rodon J, Tabernero J. Optimal design of trials to demonstrate the utility of genomically-guided therapy: Putting Precision Cancer Medicine to the test. Mol Oncol. 2015 May;9(5):940–50.	Narrative review
40	Do K, Coyne GO, Chen AP. An overview of the NCI precision medicine trials—NCI MATCH and MPACT. Chin Clin Oncol. 2015;4(3):8.	Narrative review
41	Domchek SM, Postel-Vinay S, Im S-A, Hee Park Y, Delord J-P, Italiano A, et al. An open-label, phase II basket study of olaparib and durvalumab (MEDIOLA): Updated results in patients with germline BRCA-mutated (gBRCAm) metastatic breast cancer (MBC). Cancer Res. 2019;79: PD5-04	Conference abstract
42	Doorenbos AZ, Haozous EA, Jang MK, Langford D. Sequential multiple assignment randomization trial designs for nursing research. Res Nurs Health. 2019 Dec;42(6):429–35.	Methodological study
43	Eng KH. Randomized reverse marker strategy design for prospective biomarker validation. Stat Med. 2014 Aug 15;33(18):3089–99.	Methodological study
44	Fadoukhair Z, Zardavas D, Chad MA, Goulioti T, Aftimos P, Piccart M. Evaluation of targeted therapies in advanced breast cancer: the need for large-scale molecular screening and transformative clinical trial designs. Oncogene. 2016 Apr;35(14):1743–9.	Narrative review
45	Fennell D, Hudka M, Darlison L, Lord K, Bzura A, Dzialo J, et al. P2.06-02 Mesothelioma Stratified Therapy (MiST): A Phase IIA Umbrella Trial for Accelerating the Development of Precision Medicines. J Thorac Oncol. 2019 Oct;14(10):S755–6.	Conference abstract
46	Ferrarotto R, Redman MW, Gandara DR, Herbst RS, Papadimitrakopoulou V. Lung-MAP-framework, overview, and design principles. Chin Clin Oncol. 2015;4(3):1–6.	Narrative review
47	Fountzilas E, Tsimberidou AM. Overview of precision oncology trials: challenges and opportunities. Expert Rev Clin Pharmacol. 2018 Aug 3;11(8):797–804.	Narrative review
48	Fracasso PM, Freeman DJ, Simonsen K, Shen Y, Gupta M, Comprelli A, et al. A phase 2, fast real-time assessment of combination therapies in immuno-oncology trial in patients with advanced non-small cell lung cancer (FRACTION-lung). Ann Oncol. 2016 Oct;27:vi451.	Conference abstract
49	Freidlin B, Korn EL, Gray R. Marker Sequential Test (MaST) design. Clin Trials J Soc Clin Trials. 2014 Feb;11(1):19–27.	Methodological study
50	Freidlin B, Korn EL. Biomarker-adaptive clinical trial designs. Pharmacogenomics. 2010 Dec;11(12):1679–82.	Editorial
51	Freidlin B, McShane LM, Korn EL. Randomized Clinical Trials With Biomarkers: Design Issues. JNCI J Natl Cancer Inst. 2010 Feb 3;102(3):152–60.	Commentary
52	Funcke S. Individualized, perioperative, hemodynamic goal-directed therapy in major abdominal surgery (iPEGASUS trial): study protocol for a randomized controlled trial. 2018;10.	Study protocol
53	Galanis E, Wu W, Sarkaria J, Chang SM, Colman H, Sargent D, et al. Incorporation of Biomarker Assessment in Novel Clinical Trial Designs: Personalizing Brain Tumor Treatments. Curr Oncol Rep. 2011 Feb;13(1):42–9.	Narrative review
54	Galot R, Le Tourneau C, Saada-Bouzid E, Daste A, Even C, Debruyne PR, et al. A phase II study of monalizumab in patients with recurrent/metastatic (RM) squamous cell carcinoma of the head and neck (SCCHN): Results of the I1 cohort of the EORTC-HNCG-1559 trial (UPSTREAM). Ann Oncol. 2019 Oct;30:v449–50.	Conference abstract
55	Gandara DR, Hammerman PS, Sos ML, Lara PN, Hirsch FR. Squamous Cell Lung Cancer: From Tumor Genomics to Cancer Therapeutics. Clin Cancer Res. 2015 May 15;21(10):2236–43.	Narrative review

For peer review only

56	Gao Z, Roy A, Tan M. Multistage adaptive biomarker-directed targeted design for randomized clinical trials. Contemp Clin Trials. 2015 May;42:119–31.	Methodological study
57	Garralda E, Dienstmann R, Piris-Giménez A, Braña I, Rodon J, Tabernero J. New clinical trial designs in the era of precision medicine. Mol Oncol. 2019 Mar;13(3):549–57.	Narrative review
58	Gilson C, Chowdhury S, Parmar MKB, Sydes MR. Incorporating Biomarker Stratification into STAMPEDE: an Adaptive Multi-arm, Multi-stage Trial Platform. Clin Oncol. 2017 Dec;29(12):778–86.	Narrative review
59	Gómez-López G, Dopazo J, Cigudosa JC, Valencia A, Al-Shahrour F. Precision medicine needs pioneering clinical bioinformaticians. 2017. :1–15.	Narrative review
60	Grill J, Teuff GL, Nysom K, Blomgren K, Hargrave D, McCowage G, et al. PDCT-01. Biological medicine for diffuse intrinsic pontine gliomas eradication (BIOMEDE): Results of the three-arm biomarker-driven randomized trial in the first 230 patients from Europe and Australia. Neuro-Oncology 2019; 21 (Suppl 6): vi183.	Conference abstract
61	Gronberg H, Eklund M, Lindberg J, Ullén A, Bjartell A, Andren O, et al. ProBio II: An adaptive and randomized multi-arm biomarker driven phase 2 study in men with castrate resistant prostate cancer (CRPC). J Clin Oncol. 2018; 36: TPS397	Conference abstract
62	Grose DB, McKay CJ, Cooke S, Graham JS, Duthie F, Jamieson N, et al. PRIMUS-002: A multicentre, open-label, phase II study examining FOLFOX and nab-paclitaxel (FA) and nab-paclitaxel and gemcitabine (AG) as neoadjuvant therapy for (borderline) resectable pancreatic cancer (PC), focusing on biomarker and liquid biopsy development. J Clin Oncol. 2019; 37: TPS4166	Conference abstract
63	Heckman-Stoddard BM, Smith JJ. Precision Medicine Clinical Trials: Defining New Treatment Strategies. Semin Oncol Nurs. 2014 May;30(2):109–16.	Narrative review
64	Heerspink HJL, List J, Perkovic V. New clinical trial designs for establishing drug efficacy and safety in a precision medicine era. Diabetes Obes Metab. 2018 Oct;20:14–8.	Narrative review
65	Heerspink HJL, Perkovic V. Trial Design Innovations to Accelerate Therapeutic Advances in Chronic Kidney Disease: Moving from Single Trials to an Ongoing Platform. Clin J Am Soc Nephrol. 2018 Jun 7;13(6):946–8.	Narrative review
66	Hernandez-Martinez J-M, Sánchez-Reyes R, De la Garza-Salazar JG, Arrieta O. Onco-omics Approaches and Applications in Clinical Trials for Cancer Patients. In: Translational Research and Onco-Omics Applications in the Era of Cancer Personal Genomics. Springer; 2019. p. 79–90.	Book chapter
67	Hobbs BP, Kane MJ, Hong DS, Landin R. Statistical challenges posed by uncontrolled master protocols: sensitivity analysis of the vemurafenib study. Ann Oncol. 2018 Dec;29(12):2296–301.	Methodological study
68	Hobbs BP, Landin R. Bayesian basket trial design with exchangeability monitoring: Bayesian Basket Trial Design with Exchangeability Monitoring. Stat Med. 2018 Nov 10;37(25):3557–72.	Methodological study
69	Hofmann D, Nitz U, Gluz O, Kates RE, Schinkoethe T, Staib P, et al. WSG ADAPT – adjuvant dynamic marker-adjusted personalized therapy trial optimizing risk assessment and therapy response prediction in early breast cancer: study protocol for a prospective, multi-center, controlled, non-blinded, randomized, investigator initiated phase II/III trial. Trials. 2013;14(1):261.	Study protocol
70	Hollingsworth SJ. Precision medicine in oncology drug development: a pharma perspective. Drug Discov Today. 2015 Dec;20(12):1455–63.	Narrative review
71	Hong F, Simon R. Run-In Phase III Trial Design With Pharmacodynamics Predictive Biomarkers. J Natl Cancer Inst. 2013;105(21):6.	Methodological study
72	Hyman DM, Taylor BS, Baselga J. Implementing Genome-Driven Oncology. Cell. 2017 Feb;168(4):584–99.	Narrative review
73	ISRCTN Registry. Precision Panc: Advancing personalised medicine treatment strategies for pancreatic cancer [Internet]. Available from: https://www.isrctn.com/ISRCTN14879538	Link
74	Janiaud P, Serghiou S, Ioannidis JPA. New clinical trial designs in the era of precision medicine: An overview of definitions, strengths, weaknesses, and current use in oncology. Cancer Treat Rev. 2019 Feb;73:20–30.	Narrative review

For peer review only

75	Johnson DR, Galanis E. Incorporation of Prognostic and Predictive Factors Into Glioma Clinical Trials. Curr Oncol Rep. 2013 Feb;15(1):56–63.	Narrative review
76	Jones CL, Holmgren E. An adaptive Simon Two-Stage Design for Phase 2 studies of targeted therapies. Contemp Clin Trials. 2007 Sep;28(5):654–61.	Methodological study
77	Joshi SS, Maron SB, Lomnicki S, Polite BN, Sharma M, Ibe J, et al. Personalized antibodies for gastroesophageal adenocarcinoma (PANGEA): A phase II precision medicine trial (NCT02213289) .J Clin Oncol. 2018; 36: TPS198	Conference abstract
78	Joshi YB, Light GA. Using EEG-Guided Basket and Umbrella Trials in Psychiatry: A Precision Medicine Approach for Cognitive Impairment in Schizophrenia. Front Psychiatry. 2018 Nov 19;9:554.	Narrative review
79	Kaplan R. The FOCUS4 design for biomarker stratified trials. Chin Clin Oncol. 2015;4(3):1–10.	Narrative review
80	Kesselmeier M, Scherag A. Adaptive clinical trials in sepsis research: pros and cons. Infection. 2019; 47 (Suppl 1): S1-S67.	Conference abstract
81	Kidwell KM, Wahed AS. Weighted log-rank statistic to compare shared-path adaptive treatment strategies. Biostatistics. 2013 Apr 1;14(2):299–312.	Methodological study
82	Kimani PK, Todd S, Renfro LA, Stallard N. Point estimation following two-stage adaptive threshold enrichment clinical trials: Estimators for adaptive threshold enrichment clinical trials. Stat Med. 2018 Sep 30;37(22):3179–96.	Methodological study
83	Korn EL, Freidlin B. Adaptive Clinical Trials: Advantages and Disadvantages of Various Adaptive Design Elements. JNCI J Natl Cancer Inst [Internet]. 2017 Jun [cited 2021 Mar 9];109(6). Available from: https://academic.oup.com/jnci/article-lookup/doi/10.1093/jnci/djx013	Commentary
84	Kourie HR, Aoun F. Why balls are not put inside the basket? A reflection on testicular cancer clinical trial design. Invest New Drugs. 2016 Aug;34(4):513–4.	Commentary
85	Krebs M, Ross K, Kim S, De Jonge M, Barlesi F, Postel-Vinay S, et al. P1.15-004 An Open-Label, Multitumor Phase II Basket Study of Olaparib and Durvalumab (MEDIOLA): Results in Patients with Relapsed SCLC. J Thorac Oncol. 2017 Nov;12(11):S2044–5.	Conference abstract
86	Kummar S, Williams PM, Lih C-J, Polley EC, Chen AP, Rubinstein LV, et al. Application of Molecular Profiling in Clinical Trials for Advanced Metastatic Cancers. JNCI J Natl Cancer Inst. 2015 Feb 6;107(4):djv003–djv003.	Commentary
87	Lam M, Loree JM, Lima AAP, Chun YS, Kopetz S. Accelerating Therapeutic Development through Innovative Trial Design in Colorectal Cancer. Curr Treat Options Oncol. 2018 Feb;19(2):11.	Narrative review
88	Lam VK, Papadimitrakopoulou V. Master protocols in lung cancer: experience from Lung Master Protocol. Curr Opin Oncol. 2018 Mar;30(2):92–7.	Narrative review
89	Le-Rademacher J, Dahlberg S, Lee JJ, Adjei AA, Mandrekar SJ. Biomarker Clinical Trials in Lung Cancer: Design, Logistics, Challenges, and Practical Considerations. J Thorac Oncol. 2018 Nov;13(11):1625–37.	Narrative review
90	Lee J-Y, Yi JY, Kim H-S, Lim J, Kim S, Nam BH, et al. An umbrella study of biomarker-driven targeted therapy in patients with platinum-resistant recurrent ovarian cancer: a Korean Gynecologic Oncology Group study (KGOG 3045), AMBITION. Jpn J Clin Oncol. 2019 Aug 1;49(8):789–92.	Study protocol
91	Lee J, Kim ST, Kim K, Lee H, Kozarewa I, Mortimer PG, et al. Tumor genomic profiling guides metastatic gastric cancer patients to targeted treatment: The VIKTORY Umbrella Trial. Cancer Discov. 2019 Jul 17;CD-19-0442.	Original research article reporting a clinical trial
92	Leonetti A, Boyd L, Giuliani J, Giovannetti E, Garajová I. Light and shadow on innovative clinical trial designs: reflections from the EORTC-PAMM course on ‘preclinical and early-phase clinical pharmacology.’ Expert Rev Clin Pharmacol. 2019 Nov 2;12(11):1033–6.	Narrative review
93	Li BT, Zauderer M, Chaft J, Drilon A, Eng J, Sima C, et al. Ado-trastuzumab emtansine for HER2 amplified or HER2 overexpressed cancers: A phase II “basket” trial. Cancer Res. 2015; 75: CT225	Conference abstract
94	Lin J-A, He P. Reinventing clinical trials: a review of innovative biomarker trial designs in cancer therapies. Br Med Bull. 2015 Jun;114(1):17–27.	Narrative review

For peer review only

95	Lindberg J, De Laere B, Crippa A, Eklund M, Grönberg H. ProBio: An outcome-adaptive, multi-arm, open-label, multiple assignment randomised controlled biomarker-driven trial in patients with metastatic castration-resistant prostate cancer. Ann Oncol. 2019 Oct;30:v354	Conference abstract
96	Liu S, Lee JJ. An overview of the design and conduct of the BATTLE trials. Chin Clin Oncol. 2015;4(3):13.	Narrative review
97	Lopez-Chavez A, Thomas A, Rajan A, Raffeld M, Morrow B, Kelly R, et al. Molecular Profiling and Targeted Therapy for Advanced Thoracic Malignancies: A Biomarker-Derived, Multiarm, Multihistology Phase II Basket Trial. J Clin Oncol. 2015 Mar 20;33(9):1000–7.	Original research article reporting a clinical trial
98	Maitland ML, Schilsky RL. Clinical trials in the era of personalized oncology. CA Cancer J Clin. 2011 Nov;61(6):365–81.	Narrative review
99	Mandrekar SJ, Dahlberg SE, Simon R. Improving Clinical Trial Efficiency: Thinking outside the Box. Am Soc Clin Oncol Educ Book. 2015 May;(35):e141–7.	Narrative review
100	Mandrekar SJ, Sargent DJ. All-Comers versus Enrichment Design Strategy in Phase II Trials. J Thorac Oncol. 2011 Apr;6(4):658–60.	Narrative review
101	Marabelle A, Le DT, Ascierto PA, Di Giacomo AM, De Jesus-Acosta A, Delord J-P, et al. Efficacy of Pembrolizumab in Patients With Noncolorectal High Microsatellite Instability/Mismatch Repair–Deficient Cancer: Results From the Phase II KEYNOTE-158 Study. J Clin Oncol. 2020 Jan 1;38(1):1–10.	Original research article reporting a clinical trial
102	Matsui S, Crowley J. Biomarker-Stratified Phase III Clinical Trials: Enhancement with a Subgroup-Focused Sequential Design. Clin Cancer Res. 2018 Mar 1;24(5):994–1001.	Methodological study
103	Mazzarella L, Morganti S, Marra A, Trapani D, Tini G, Pelicci P, et al. Master protocols in immuno-oncology: do novel drugs deserve novel designs? J Immunother Cancer. 2020 Mar;8(1):e000475.	Narrative review
104	Moore KN, Mannel RS. Is the NCI MATCH trial a match for gynecologic oncology? Gynecol Oncol. 2016 Jan;140(1):161–6.	Narrative review
105	Nakamura Y, Komatsu Y, Kato K, Shinozaki E, Bando H, Kato T, et al. bTMB-High Basket trial: A multicenter phase II trial of nivolumab monotherapy in patients with advanced gastrointestinal cancers with high blood tumor mutational burden (bTMB). J Clin Oncol 2019; 37: TPS179	Conference abstract
106	Nitz U, Gluz O, von Schumann R, Hofmann D, Kates RE, Kuemmel S, et al. ADAPT - Adjuvant Dynamic marker-Adjusted Personalized Therapy trial optimizing risk assessment and therapy response prediction in early breast cancer. Cancer Res. 2015; 75: OT3-2-04	Conference abstract
107	Noda S, Yonemori K, Shirakawa N, Okuma HS, Shimizu T, Hirakawa A, et al. MASTER KEY project: A basket/umbrella trial for rare cancers in Japan.Ann Oncol. 2018 Oct;29:viii148	Conference abstract
108	O'Brien C, Carter L, Cook N, Dean E. Novel Early Phase Clinical Trial Design in Oncology. Pharm Med. 2017 Oct;31(5):297–307.	Narrative review
109	Ocana A, Amir E, Vera-Badillo F, Seruga B, Tannock IF. Phase III Trials of Targeted Anticancer Therapies: Redesigning the Concept. Clin Cancer Res. 2013 Sep 15;19(18):4931–40.	Narrative review
110	Ohwada S, Morita S. Bayesian adaptive patient enrollment restriction to identify a sensitive subpopulation using a continuous biomarker in a randomized phase 2 trial. Pharm Stat. 2016 Sep;15(5):420–9.	Methodological study
111	Ondra T, Dmitrienko A, Friede T, Graf A, Miller F, Stallard N, et al. Methods for identification and confirmation of targeted subgroups in clinical trials: A systematic review. J Biopharm Stat. 2016 Jan 2;26(1):99–119.	Systematic review
112	Ou F-S, An M-W, Ruppert AS, Mandrekar SJ. Discussion of Trial Designs for Biomarker Identification and Validation Through the Use of Case Studies. JCO Precis Oncol. 2019 Dec;(3):1–10.	Narrative review
113	PanACEA consortium, Phillips P, Hoelscher M, Bratton D, Rehal S, Heinrich N, et al. Modifying the multi-arm multi-stage (MAMS) design for use in a phase II tuberculosis trial in sub-Saharan Africa with a time-to-event primary outcome. Trials. 2013 Nov;14(S1):P26, 1745-6215-14-S1-P26.	Conference abstract
114	Paoletti X, Asselain B, Kamal M, Servant N, Huppé P, Bieche I, et al. Design and statistical principles of the SHIVA trial. Chin Clin Oncol. 2015;4(3):1–10.	Narrative review

For peer review only

115	Park JJH, Hsu G, Siden EG, Thorlund K, Mills EJ. An overview of precision oncology basket and umbrella trials for clinicians. CA Cancer J Clin. 2020 Mar;70(2):125–37.	Narrative review
116	Park JJH, Siden E, Zoratti MJ, Dron L, Harari O, Singer J, et al. Systematic review of basket trials, umbrella trials, and platform trials: a landscape analysis of master protocols. Trials. 2019 Dec;20(1):572.	Systematic review
117	Park S, Hur JY, Yoon SE, Lee K, Kim Y, Cho JH, et al. P2.12-05 SUKSES (Small Cell Lung Cancer Umbrella Korea Studies): A Phase II Biomarker-Driven Umbrella Study in Relapsed or Refractory SCLC. J Thorac Oncol. 2018 Oct;13(10):S792.	Conference abstract
118	Park S, Shim J, Jung HA, Sun J-M, Lee S-H, Park W-Y, et al. Biomarker driven phase II umbrella trial study of AZD1775, AZD2014, AZD2811 monotherapy in relapsed small cell lung cancer. J Clin Oncol 2019: 37: 8514	Conference abstract
119	Parke T, Pericàs JM, Posch M, Heimbach N, Spiertz C, Mesenbrink P, et al. D2.1. Report on Terminology, References and Scenarios for Platform Trials and Master Protocols. 2020.	Report
120	Patel SP, Othus M, Chae YK, Giles FJ, Hansel DE, Singh PP, et al. A Phase II Basket Trial of Dual Anti–CTLA-4 and Anti–PD-1 Blockade in Rare Tumors (DART SWOG 1609) in Patients with Nonpancreatic Neuroendocrine Tumors. Clin Cancer Res. 2020 May 15;26(10):2290–6.	Original research article reporting a clinical trial
121	Perco P, Pena M, Heerspink HJL, Mayer G. Multimarker Panels in Diabetic Kidney Disease: The Way to Improved Clinical Trial Design and Clinical Practice? Kidney Int Rep. 2019 Feb;4(2):212–21.	Narrative review
122	Redman MW, Allegra CJ. The Master Protocol Concept. Semin Oncol. 2015 Oct;42(5):724–30.	Narrative review
123	Renfro LA, An M-W, Mandrekar SJ. Precision oncology: A new era of cancer clinical trials. Cancer Lett. 2017 Feb;387:121–6.	Narrative review
124	Renfro LA, Mallick H, An M-W, Sargent DJ, Mandrekar SJ. Clinical trial designs incorporating predictive biomarkers. Cancer Treat Rev. 2016 Feb;43:74–82.	Narrative review
125	Renfro LA, Mandrekar SJ. Definitions and statistical properties of master protocols for personalized medicine in oncology. J Biopharm Stat. 2018 Mar 4;28(2):217–28.	Narrative review
126	Renfro LA, Sargent DJ. Statistical controversies in clinical research: basket trials, umbrella trials, and other master protocols: a review and examples. Ann Oncol. 2017 Jan;28(1):34–43.	Narrative review
127	Riddell CA, Zhao Y, Petkau J. An adaptive clinical trials procedure for a sensitive subgroup examined in the multiple sclerosis context. Stat Methods Med Res. 2016 Aug;25(4):1330–45.	Methodological study
128	Rosenblum M, Hanley DF. Adaptive Enrichment Designs for Stroke Clinical Trials. Stroke. 2017 Jul;48(7):2021–5.	Narrative review
129	Said R, Tsimberidou A-M. Basket Trials and the MD Anderson Precision Medicine Clinical Trials Platform: Cancer J. 2019;25(4):282–6.	Narrative review
130	Saville BR, Berry SM. Efficiencies of platform clinical trials: A vision of the future. Clin Trials J Soc Clin Trials. 2016 Jun;13(3):358–66.	Methodological study
131	Schmoll H-J, Arnold D, de Gramont A, Ducreux M, Grothey A, O'Dwyer PJ, et al. MODUL—a multicenter randomized clinical trial of biomarker-driven maintenance therapy following first-line standard induction treatment of metastatic colorectal cancer: an adaptable signal-seeking approach. J Cancer Res Clin Oncol. 2018 Jun;144(6):1197–204.	Original research article reporting a clinical trial
132	Sebag-Montefiore D, Adams R, Bell S, Berkman L, Gilbert DC, Glynne-Jones R, et al. The Development of an Umbrella Trial (PLATO) to Address Radiation Therapy Dose Questions in the Locoregional Management of Squamous Cell Carcinoma of the Anus. Int J Radiat Oncol. 2016 Oct;96(2):E164–5.	Conference abstract
133	Semler MW, Bernard GR, Aaron SD, Angus DC, Biros MH, Brower RG, et al. Identifying Clinical Research Priorities in Adult Pulmonary and Critical Care. NHLBI Working Group Report. Am J Respir Crit Care Med. 2020 Aug 15;202(4):511–23.	Report

For peer review only

134	Shah SJ. Innovative Clinical Trial Designs for Precision Medicine in Heart Failure with Preserved Ejection Fraction. J Cardiovasc Transl Res. 2017 Jun;10(3):322–36.	Narrative review
135	Simon KC, Tideman S, Hillman L, Lai R, Jathar R, Ji Y, et al. Design and implementation of pragmatic clinical trials using the electronic medical record and an adaptive design. JAMIA Open. 2018 Jul 1;1(1):99–106.	Methodological study
136	Simon R. Clinical trial designs for evaluating the medical utility of prognostic and predictive biomarkers in oncology. Pers Med. 2010 Jan;7(1):33–47.	Narrative review
137	Simon R. Clinical trials for predictive medicine: new challenges and paradigms. Clin Trials J Soc Clin Trials. 2010 Oct;7(5):516–24.	Narrative review
138	Simon R. Critical Review of Umbrella, Basket, and Platform Designs for Oncology Clinical Trials: Review of umbrella, basket, and platform trial designs. Clin Pharmacol Ther. 2017 Dec;102(6):934–41.	Narrative review
139	Simon R. Genomic Alteration–Driven Clinical Trial Designs in Oncology. Ann Intern Med. 2016 Aug 16;165(4):270.	Narrative review
140	Simon R. New designs for basket clinical trials in oncology. J Biopharm Stat. 2018 Mar 4;28(2):245–55.	Narrative review
141	Simonsen KL, Fracasso PM, Bernstein SH, Wind-Rotolo M, Gupta M, Comprelli A, et al. The Fast Real-time Assessment of Combination Therapies in Immuno-ONcology (FRACTION) program: innovative, high-throughput clinical screening of immunotherapies. Eur J Cancer. 2018 Nov;103:259–66.	Original research article reporting a clinical trial
142	Skamene T, Siu LL, Renouf DJ, Laskin JJ, Bedard PL, Jones SJM, et al. Canadian profiling and targeted agent utilization trial (CAPTUR/PM.1): A phase II basket precision medicine trial. J Clin Oncol 2018. 36: TPS12127	Conference abstract
143	Soldatos, Kaduthanam, Jackson. Precision Oncology—The Quest for Evidence. J Pers Med. 2019 Sep 5;9(3):43.	Narrative review
144	Spigel D, Garassino M, Besse B, Sacher A, Barve M, Cousin S, et al. P1.01-110 Novel Regimens Versus Standard-of-Care in NSCLC: A Phase II, Randomized, Open-Label, Platform Trial Using a Master Protocol. J Thorac Oncol. 2019 Oct;14(10):S404–S405.	Conference abstract
145	Tajik P, Zwinderman AH, Mol BW, Bossuyt PM. Trial Designs for Personalizing Cancer Care: A Systematic Review and Classification. Clin Cancer Res. 2013 Sep 1;19(17):4578–88.	Systematic review
146	Talisa VB, Yende S, Seymour CW, Angus DC. Arguing for Adaptive Clinical Trials in Sepsis. Front Immunol. 2018 Jun 28;9:1502.	Narrative review
147	Tao JJ, Schram AM, Hyman DM. Basket Studies: Redefining Clinical Trials in the Era of Genome-Driven Oncology. Annu Rev Med. 2018 Jan 29;69(1):319–31.	Narrative review
148	Thavaneswaran S, Sebastian L, Ballinger M, Best M, Hess D, Lee CK, et al. Cancer Molecular Screening and Therapeutics (MoST): a framework for multiple, parallel signal-seeking studies of targeted therapies for rare and neglected cancers. Med J Aust. 2018 Oct;209(8):354–5.	Study protocol
149	Thavaneswaran S, Sebastian L, Ballinger M, Cowley M, Grady J, Joshua A, et al. The Cancer Molecular Screening and Therapeutics Program (MoST) – A molecular screening platform with multiple, parallel, signal-seeking therapeutic substudies. In Annals of Oncology; 2018. p. viii133–48.	Conference abstract
150	Timmers L, Van Waalwijk Van Doorn S, Pisters A, Van Saase L, Voest E. Ppm1 A Risk Sharing Model For Biomarker-Driven Treatment Of Rare Subgroups Of Cancer Patients. Value Health. 2019 Nov;22:S837.	Conference abstract
151	Trippa L, Alexander BM. Bayesian Baskets: A Novel Design for Biomarker-Based Clinical Trials. J Clin Oncol. 2017 Feb;35(6):JCO.2016.68.286.	Methodological study
152	Tsimberidou AM, Fountzilas E, Nikanjam M, Kurzrock R. Review of precision cancer medicine: Evolution of the treatment paradigm. Cancer Treat Rev. 2020 Jun;86:102019.	Narrative review
153	Uozumi R, Hamada C. Interim decision-making strategies in adaptive designs for population selection using time-to-event endpoints. J Biopharm Stat. 2017 Jan 2;27(1):84–100.	Methodological study
154	Van Norman GA. Phase II Trials in Drug Development and Adaptive Trial Design. JACC Basic Transl Sci. 2019 Jun;4(3):428–37.	Narrative review

155	Verweij J, Hendriks HR, Zwierzina H, Hanauske, Wacheck V, Collignon O, et al. Innovation in oncology clinical trial design. Cancer Treat Rev. 2019 Mar;74:15–20.	Narrative review
156	Vijverberg SJ, Pijnenburg MW, Hövels AM, Koppelman GH, Maitland-van der Zee A-H. The need for precision medicine clinical trials in childhood asthma: rationale and design of the PUFFIN trial. Pharmacogenomics. 2017 Mar;18(4):393–401.	Report
157	Wang S-J, Hung HMJ, O'Neill R. Adaptive design clinical trials and trial logistics models in CNS drug development. Eur Neuropsychopharmacol. 2011 Feb;21(2):159–66.	Narrative review
158	Wang T, Wang X, Zhou H, Cai J, George SL. Auxiliary variable–enriched biomarker-stratified design. Stat Med. 2018 Dec 30;37(30):4610–35.	Methodological study
159	Weber J, Long GV, Haanen JB, Arance A, Dummer R, Nathan P, et al. A randomized, open-label, phase II open platform study evaluating the efficacy and safety of novel spartalizumab (PDR001) combinations in previously treated unresectable or metastatic melanoma (PLATForM). Ann Oncol. 2018;29:viii442-viii466	Conference abstract
160	Xu Y, Trippa L, Müller P, Ji Y. Subgroup-Based Adaptive (SUBA) Designs for Multi-arm Biomarker Trials. Stat Biosci. 2016 Jun;8(1):159–80.	Methodological study
161	Yee LM, McShane LM, Freidlin B, Mooney MM, Korn EL. Biostatistical and Logistical Considerations in the Development of Basket and Umbrella Clinical Trials: Cancer J. 2019;25(4):254–63.	Narrative review
162	Yu H, Goldberg S, Le X, Piotrowska Z, Smith P, Mensi I, et al. P2.01-22 ORCHARD: A Phase II Platform Study in Patients with Advanced NSCLC Who Have Progressed on First-Line Osimertinib Therapy. J Thorac Oncol. 2019 Oct;14(10):S647.	Conference abstract
163	Yuan Y. Invited session 11 - Recent developments in umbrella, basket and platform trial designs. Clinical Trials. 2018; 15(S2);35-192	Conference abstract
164	Zardavas D, Piccart-Gebhart M. Clinical Trials of Precision Medicine through Molecular Profiling: Focus on Breast Cancer. Am Soc Clin Oncol Educ Book. 2015 May;(35):e183–90.	Narrative review
165	Zhang W, Wang J, Menon S. Advancing cancer drug development through precision medicine and innovative designs. J Biopharm Stat. 2018 Mar 4;28(2):229–44.	Narrative review
166	Zhang Z, Chen R, Soon G, Zhang H. Treatment evaluation for a data-driven subgroup in adaptive enrichment designs of clinical trials: Treatment evaluation for a data-driven subgroup in adaptive enrichment designs of clinical trials. Stat Med. 2018 Jan 15;37(1):1–11.	Methodological study
167	Zhou Q, Zhang X-C, Tu H-Y, Gan B, Wang B-C, Xu C-R, et al. Biomarker-integrated study of single agent targeting molecular alterations of PI3KCA, MET, ALK, ROS1, KRAS, NRAS or BRAF in advanced NSCLC: Phase 2 umbrella trial in China (CTONG1505). Ann Oncol. 2018 Nov;29:ix113.	Conference abstract

For peer review only

Supplementary file IV. Definition, methodology, and statistical considerations of identified trial designs

Trial designs	Sub-type of trial designs	Variations	Definition	Methodology	Statistical considerations
**Marker stratified design**			The marker-by-treatment interaction design detects the interaction between biomarker and treatment effect by using biomarker status as stratum (or strata) with the presumption that the entire population can be separated by marker-defined subgroup(s). (Lin2015)	All patients are randomly assigned to treatments, but the results are analyzed according to biomarker status. (Ahmad2013)	Marker-stratified designs can be conducted using two different testing plans; the so-called 1) marker-by-treatment interaction with separate tests and 2) marker-by-treatment interaction with interaction test. Both of these approaches involve conducting two independent clinical trials.<br><br>1) The marker-by-treatment interaction design using separate tests is a testing plan which determines whether the novel treatment is superior to the control treatment separately within each biomarker-defined subgroup. Consequently, the hypothesis to be tested, the calculation of the number of patients required for the trial, the estimation of the statistical power of the design and the randomization procedure of patients to different treatments are independent among the different subgroups. The sample size of the trial should be calculated in such a way so as to yield adequate statistical power when testing whether the experimental treatment is superior to the control treatment separately in the two biomarker-defined subgroups. Hence, this approach is not widely used due to the required large sample size as essentially two separate trials are being conducted. Another limitation of this approach is that when multiple biomarker-defined subsets and treatments are to be investigated, it is difficult to implement in practice.<br><br>2) The marker-by-treatment interaction using interaction test uses a test for interaction between the biomarker status and treatment assignment. A marker stratified design which uses this testing plan is also referred to in the literature as an "interaction design" or "genomic signature stratified design". First, a formal statistical test for interaction between biomarker status and treatment assignment is undertaken. If this interaction is not significant, then the study is continued by testing the different treatments overall at a two-sided significance level of 0.05, otherwise, the treatments are compared within each biomarker-defined subpopulation at a two-sided 0.05 significance level (i.e., the same as in the marker-by-treatment interaction design using separate tests). The sample size for this second testing plan is calculated with reference to the

For peer review only

					treatment effect in the entire study population. Therefore, it might not provide sufficient power for detecting the treatment effect in each biomarker defined-subset individually. More precisely, if the sample size is calculated for the overall analysis and the proportion of the biomarker-defined subpopulation which responds to the novel treatment is very small, the statistical power for the subgroup analysis may be inadequate. In addition, when several biomarker-defined subpopulations and treatments are to be investigated, this strategy is not easy to be implemented. (Antoniou2017)
				Individuals are stratified into biomarker-positive and biomarker-negative subgroups according to the results of the biomarker assessment and then they are randomized either to the experimental or to the control treatment group. The biomarker status in the Marker-Stratified design acts as a stratification factor where stratification is used to ensure balance across treatment groups with regard to biomarkers. Only individuals with valid biomarker results enter the trial. Consequently, we have four treatment groups, i.e., biomarker-positive patients assigned to either the experimental treatment arm or the control treatment arm and biomarker-negative patients assigned to either the experimental treatment arm or the control treatment arm. (Antoniou2017)	***It refers to marker-by-treatment interaction with separate tests***<br><br>The hypothesis to be tested, the sample size calculation and power estimation, and the randomization procedure are independent among subgroups. (Galanis2011)
			[…] a trial randomizing patients to experimental versus control treatments within marker-defined subgroups (Renfro2016_Clinical trial designs incorporating)	***It refers to marker-by-treatment interaction with separate tests***<br><br>[…] all patients with a valid marker result are assigned to a marker-based subgroup, and within each subgroup, patients are randomized between two or more treatment arms. (Galanis2011)	***It refers to marker-by-treatment interaction with interaction test***<br><br>[…] the sample size is calculated to provide adequate power to test for a different treatment effect in the two marker groups (Galan2011)
				In this design, patients are randomized in different treatment groups. Although their biomarker status is prospectively determined, it does not impact on treatment decision. […] A variation on the marker by treatment interaction design allows for its use in trials in which each arm does not need to be individually powered to evaluate the primary hypothesis, but instead the trial as a whole is powered to assess for interaction between treatment effect and biomarker subgroup. (Johnson2013)	The sample size is, however, calculated to provide adequate power to test for a different treatment effect in the different marker groups (Johnson2013)

				The subjects are then randomized to treatment arms within marker defined groups. Statistical modeling including interaction effect or statistical test for dependency between two factors, such as interaction term of treatment by biomarker for continuous end point or X^2 for categorical end point, may then be implemented. (Lin2015)	[…] several null hypotheses are tested to examine the efficacy of the experimental treatment. This leads to Type I error rate inflation and a multiplicity adjustment must be applied to control the familywise error rate (FWER) in the strong sense. (Ondra2016)
				This design includes four arms, where patients are screened for biomarker status and randomization, stratified for the biomarker status, is performed. Biomarker-positive as well as biomarker-negative patients are randomized to the treatment T and control C […]. (Ondra2016)	• Requires excellent assay performance<br>• Requires fast assay turn-around time<br><br>(From Table 1. Renfro2016_Clinical trial designs incorporating)
				In this design, all patients are randomized to experimental versus control treatments; however, patients are first stratified by marker status and then randomized to a treatment arm within their given marker cohort. (Renfro2017_Precision oncology)	
				In this case the RCT comparing the new treatment to control includes both test-positive and test-negative patients, but a prospective primary analysis plan stipulating how the test will be used in the analysis of treatment effect is defined in the protocol. (Simon2010_Clinical trials for predictive)	
	**Subgroup specific design**			In this design, all patients with a particular disease are randomly assigned to experimental therapy versus SOC, but coprimary objectives are defined to test for superior clinical outcome with the experimental therapy in the subgroup of patients positive for the biomarker as well as in either the subgroup of patients negative for the biomarker or in all patients. With co-primary objectives, the significance level (α) is allocated or split between the two objectives to maintain an acceptable overall type I family-wise error rate using a conservative Bonferroni correction or a less conservative correction that considers the correlation between the two tests. In the case with co-primary objectives defined for the biomarker-positive subgroup and all patients, the design can be subgroup focused or all-population focused. (Ou2019)	

		**Sequential-subgroup specific design**		The sequential testing procedure uses the assumption that it is unlikely that the new treatment will be effective in the biomarker-negative patients unless it is effective in the biomarker-positive patients. First treatment effect is tested in the biomarker-positive subpopulation using the overall two-sided significance level α = 0.05 (Type I error); if this test is significant then treatment effect is tested in the biomarker-negative subgroup using the same level of significance α. (Antoniou2017)	[…] requires a smaller number of positive patients as compared to the second type of subgroup-specific design, the so-called parallel subgroup-specific design (Antoniou2017)
		**Parallel-subgroup specific design**	[…] evaluates treatment effects separately in the positive biomarker-defined subgroup and in the negative biomarker-defined subgroup simultaneously. (Antoniou2017)	In order to control the overall type I error rate of the design at the overall level of significance (Type I error) it is required to allocate this overall between the test for the biomarker-positive subgroup and the test for the biomarker-negative subgroup using the Bonferroni correction method for multiple testing.This trial design is powered in such a way so as to detect the treatment effect in each biomarker-defined subgroup separately. A higher portion of the type I error rate can be given for the test within the biomarker-positive subgroup in order to maximize the power of the trial to identify the treatment effect in this subpopulation. However, even if there is a slight increase in the type I error probability spent on the test of one of the biomarker-define subgroups, the power would probably not change much. (Antoniou2017)	
	**Biomarker-positive and overall strategies**	**Biomarker-positive and overall strategies with parallel assessment**	In the parallel version, we test both the overall population and biomarker-positive subgroup simultaneously. (Antoniou2017)	In this approach the treatment effect is tested in both the entire study population and in the biomarker-positive patients while controlling the type I error by allocating the overall significance level between the two tests. The significance level a can be considered as one-sided or two-sided. (Antoniou2017)	As this design comprises two sequential stages, it follows that the sample size calculation should also be staged. At the first stage, the standard formula for a traditional randomized trial can be used for the biomarker-positive subgroup using the significance level a to estimate the treatment effect in that subset. More precisely, the formula used in the enrichment design for the required total number of events or the required number of patients can be used at the first stage of this design. At the second stage, the sample size must be adjusted in order to yield appropriate power for the entire population. (Antoniou2017)

For peer review only

		**Biomarker-positive and overall strategies with sequential assessment**		In this sequential version of the biomarker-positive and overall strategies, we first test the biomarker-positive subgroup using the significance level a; if the test is significant, then we test the treatment effect in the overall population using the same a level. The significance levels a can be considered as one-sided or two-sided significance levels. (Antoniou2017)	
		**Biomarker-positive and overall strategies with fall-back analysis**	It evaluates both the treatment effect in the overall study population and in the biomarker-positive subgroup sequentially. (Antoniou2017)	In the fall-back design, we first test the overall population using the reduced significancance level α^1 and if the test is significant, we consider that the novel treatment is effective in the overall population; however, if the result is not significant then we test the treatment effect in the biomarker-positive subgroup using the level of significance $\alpha^2 = \alpha - \alpha^1$,where a is the overall significance level (Type I error rate). The significance levels a can be considered as one-sided or two-sided significance levels. (Antoniou2017)	The sample size should be set in such a way so as to yield adequate power for the overall test at the reduced significance level $\alpha^1$and for the potential biomarker positive subgroup analysis at significance level $\alpha - \alpha^1$, (Antoniou2017)
		**Marker sequential test design**	[…] allows sequential testing of the treatment effect in the biomarker subgroups and overall population while controlling the relevant type I error rates. (Freidlin2014)	This design sequentially tests the treatment effect in the subgroups and the overall population. First, the biomarker-positive subgroup is tested at a reduced level α^1. If it is significant, then the biomarker negative subgroup is tested at the level α. If the biomarker-positive subgroup test is not significant, then the overall population is tested at the $\alpha^2 = \alpha - \alpha^1$ level. For any choice of a1 (in [0, α]), the design controls the probability of rejecting H0+ or H0- under the global null at level a. (Freidlin2014)	
			[…] it evaluates not only the biomarker-positive and biomarker-negative subgroups but also the entire population sequentially to limit the assessment of treatment effect in the overall population when it seems that the biomarker-positive subgroup does not benefit from the novel treatment. (Antoniou2017)	In this design which owns an adaptive nature, first the biomarker-positive subgroup is tested at a reduced level α^1 in [0, α] and if the results is significant, then the biomarker-negative subgroup is tested at the global significance level a. Otherwise, if the result is not significant, then the overall population is tested at level $\alpha^2 = \alpha - \alpha^1$ in order to make a treatment recommendation for the biomarker-negative patients. (Antoniou2017)	
		**Auxiliary variable–enriched biomarker-stratified design (AEBSD)**	[…] we focus on a new auxiliary variable-enriched biomarker-stratified design (AEBSD) where the M+ subpopulation is enriched through an inexpensive auxiliary variable that is moderately or highly correlated to the true biomarker. This design retains the assessment of the treatment effects for the desired		

For peer review only

			subpopulation and the overall population while maintaining the "enriched" feature of trial design for efficiency. (Wang2018)		
**Hybrid design**			In this approach, only the biomarker-positive patients are randomly assigned to either the experimental treatment group or to the control treatment group whereas the biomarker-negative patients receive the control treatment. (Antoniou2017)	Similar to the enrichment design, hybrid designs are powered to identify treatment effect only in the biomarker-defined subgroup, which is randomly assigned to the experimental or control treatment groups. Consequently, the same formula used for the required number of patients or events for the enrichment designs can be used for hybrid designs. (Antoniou2017)	
			The most straightforward hybrid design is an extension from enrichment design: subjects who do not have predicted responsive biomarker will stay in the study and receive standard care. (Lin2015)		
			[…] an enrichment flow is combined in parallel with a single-arm trial of standard therapy in biomarker-negative patients (Tajik2013)		
**Biomarker strategy design with biomarker assessment in the control arm**			Biomarker status is assessed in all patients enrolled in the trial, who are then randomly allocated to either the biomarker-strategy arm or to standard treatment. (Tajik2013)	First, the study population enrolled in the trial is tested for its marker status. Next, patients irrespective of their biomarker status are randomized either to the biomarker-based strategy arm (also referred to as personalized arm) or to the non-biomarker-based strategy arm. In the biomarker-based strategy arm, biomarker-positive patients receive the experimental treatment, whereas, biomarker-negative patients receive the control treatment. Patients who are randomized to the non-biomarker-based strategy arm receive the control treatment irrespective of their biomarker status. (Antoniou2017)	
			A design that focuses specifically on the role of a biomarker in the treatment decision-making process […]. (Renfro2016_Clinical trial designs incorporating)	In this design, patients are randomized at the time of screening to a treatment strategy (often standard of care) that ignores the biomarker versus a strategy taking biomarker status into account, through direct assignment to targeted therapies matched to the biomarker status of each eligible patient. Primary outcome analyses are then made between treatment strategies rather than specific treatments, with the hypothesis that better outcomes will be observed among those patients treated according to (versus independent of) their biomarker status. At the same time, questions regarding the best treatment for patient subgroups may remain unanswered as treatment randomization within marker	

For peer review only - http://bmjopen.bmj.com/site/about/guidelines.xhtml

				subgroups may not occur. (Renfro2016_Clinical trial designs incorporating)	
				In this design, patients are screened for biomarkers and then randomized to a treatment strategy that takes biomarker status into account (often a targeted therapy) versus a treatment that ignores the biomarker (often a stardard care.) (Renfro2017_Precision oncology)	
**Biomarker strategy design without biomarker assessment in the control arm**			In settings where it is not feasible or ethical to evaluate the biomarker in all patients, biomarker status is only acquired in patients allocated to the biomarker-strategy arm. (Tajik2013)	In this approach, patients are again randomized between testing strategies (i.e.,biomarker-based strategy and non-biomarker-based strategy) but it differs in terms of the timing of biomarker evaluation. More precisely, first, patients are randomized to either the biomarker-based strategy or to the non-biomarker-based strategy. Next, this design evaluates the biomarkers only in patients who are assigned to the biomarker-based strategy. Patients who are found to be biomarker-positive will receive the experimental treatment and patients who are biomarker-negative will receive the control treatment. On the other hand, the population which is randomized to the non-biomarker-based strategy will receive the control treatment. (Antoniou2017)	The same mathematical formula for sample size calculation assuming a continuous clinical outcome proposed by Young et al. (2010) and the formula assuming binary outcome proposed by Eng, 2014 for the biomarker-strategy design with biomarker assessment in the control arm could be applied. Further, in terms of survival outcome, the same formula provided for the required number of events in the first version of biomarker-strategy designs (i.e., biomarker-strategy design with biomarker assessment in the control arm) could be considered. (Antoniou2017)
				In the marker-based strategy design, each patient with known marker status is randomly assigned to two strategy groups: the marker-based strategy group, and the non marker-based strategy group. All patients assigned to the marker-based strategy group are assigned to different treatments (standard or experimental) based on their biomarker status, while patients assigned to the non marker-based strategy group all receive the standard treatment. (Galanis2011)	• Requires strong predictive marker evidence<br>• Requires excellent assay performance<br>• Requires fast assay turn-around time (From Table 1. Renfro2016_Clinical trial designs incorporating)

				Biomarker strategy design recruits eligible subjects regardless of their biomarker status, just like all-comer design. The subjects will then be randomized to control arm (to receive placebo or standard care) or experimental arm. For the subjects in the experimental arm, their biomarker status will be tested before they are assigned to intervention treatment group or control group depending on their biomarker status. (Lin2015)	
				Patients are randomized to either the control (without screening) or the biomarker-guided treatment strategy arm. Within the latter arm, the biomarker status is determined and all biomarker positive patients receive the experimental treatment T whereas the biomarker-negative patients receive the control C. (Ondra2016)	
				The control arm determines treatment using practice standards based on staging and existing prognostic factors. The new biomarker is not measured for patients that are randomized to the control arm. Patients randomized to the experimental arm have the candidate biomarker measured and this is used in conjunction with staging and other prognostic factors to determine treatment. This design is very flexible, but often very inefficient in the sense that the same objectives can be obtained with fewer patients using other designs. (Simon2010_Clinical trial designs for evaluating)	
**Biomarker strategy design with treatment randomisation in the control arm**			The biomarker-strategy design with treatment randomization in the control treatment is able to inform us about whether the biomarker-based strategy is better than not only the standard treatment but also better than the experimental treatment in the overall population. (Antoniou2017)	Patients are first randomly assigned to either the biomarker-based strategy arm or to the non-biomarker-based strategy arm. Next, patients who are allocated to the non-biomarker-based strategy are again randomized either to the experimental treatment arm or to the standard treatment arm irrespective of their biomarker status. Patients who are allocated to the biomarker-based strategy and who are biomarker-positive are given the experimental treatment and patients who are biomarker-negative are given the control treatment. (Antoniou2017)	This design may require a larger sample size because some of the biomarker-negative patients in the randomization arm also receive the control treatment and some of the biomarker-positive patients the experimental treatment. This leads to a diluted treatment effect and may result in lower statistical power. (Ondra2016)

For peer review only

			[…] patients randomized to the non-biomarker strategy arm are again randomized between the experimental treatment and control. This design tests the impact of the biomarker-guided strategy against a random allocation procedure which does not take the biomarker into account. (Ondra2016)	[…] all patients in the non marker-based strategy group will have a second randomization and are assigned to one of the two treatments being used in the marker-based group. (Galanis2011)	
			[…] modification of the biomarker-strategy design, wherein a second randomization between experimental versus control therapy replaces the control arm. (Tajik2013)		
**Reverse marker based strategy**			[…] version of biomarker-strategy designs where the non-biomarker-based strategy arm which is included in the three aforementioned subtypes of biomarker-strategy designs is replaced by the reverse marker-strategy arm. (Antoniou2017)	In this design patients are randomized either to the biomarker-based strategy arm or the reverse biomarker-based strategy arm. As in the previous three biomarker-strategy subtype designs, patients who are allocated to the biomarker-strategy arm receive the experimental treatment if they are biomarker-positive whereas biomarker-negative patients receive the control treatment. By contrast, patients who are randomly assigned to the reverse biomarker-based strategy arm receive control treatment if they are biomarker-positive, whereas biomarker-negative patients receive experimental treatment. (Antoniou2017)	
			[…] it employs a two-arm randomization scheme, provides a direct estimate of the marker-strategy response rate, and evaluates the interaction between the marker and possible treatments. (Eng2014)	Patients are randomly assigned to one of the two treatment strategies. In the first arm biomarker-positive patients receive the experimental treatment whereas biomarker-negative patients are allocated to receive the control. By contrast, in the second arm biomarker-positive patients receive the control and biomarker-negative patients receive the treatment. (Ondra2016)	
**Modified biomarker strategy design**			[…] is similar to a marker strategy design, except that it includes multiple targeted molecular profiles, thereby accommodating a more heterogeneous patient population. (Renfro2017_Precision oncology)	In this framework, the final analysis compares the marker-based strategy arm versus the non marker- based strategy arm (i.e. conventional, physician-directed) across all profiles. (Renfro2017_Precision oncology)	
			[…] measuring the test in all patients and only randomizing patients for whom the treatment assignment is influenced by marker result (Simon2010_Clinical trial designs for evaluating)	Before randomization, the practice standard-determined treatment and the marker-based treatment are identified. Only patients for whom the two treatments differ are randomized. (Simon2010_Clinical trial designs for evaluating)	
			[…] only patients for whom the treatment assignment is influenced by biomarker results are randomized (Tajik2013)		

For peer review only

**Sequential Multiple Assignment Randomised Trial (SMART) design**			The SMART design is used to sequence interventions based on a person's response. As such, the SMART design involves comparing sequences of interventions in terms of the effectiveness of the intervention, as well as the adjustment of intervention components and duration. SMART designs provide a systematic approach for testing decision rules involved in sequencing interventions (Doorenbos2019)<br>The SMART design allows for the assessment and comparison of adaptive treatment strategies (ATSs, also known as dynamic treatment regimes), which consist of a sequence of individually tailored therapies during the course of treatment. (Kidwell2013)	[…] the planning process can be broken into four main components or key steps: (a) Formulate the research question(s) to be answered, (b) identify and decide the intervention sequences, (c) define the response to the interventions, and (d) identify tailoring variables. (Doorenbos2019)	
**Adaptive biomarker design**			This method allows adaptations to trial design based on interim analysis of the treatment responses of biomarkers, such as genomic markers. This design can be used to select patient populations for subsequent trials, identify the natural course of a disease, achieve early detection of a disease and/or help in developing personalised medicine. (Bothwell2018)<br>Adaptations to the trial design based on interim analysis of the treatment responses of biomarkers, such as genomic markers. This design can be used to select patient populations for subsequent trials, identify the natural course of a disease, achieve early detection of a disease, and/or help in developing personalized medicine. (Cerqueira2019)<br>Interim analysis of treatment responses of biomarkers allows pre-specified adaptations to trial design (Van Norman2019)	Let S(k) denote the log-likelihood measure of treatment effect for patients who are positive for biomarker Bk and let k* denote the biomarker for which S(k) is maximum. The statistical significance of S(k*) is determined by permuting the treatment group labels of the patients and then re-evaluating the treatment effects within the positive subsets of the K binary classifiers. Using bootstrap resampling, one can evaluate the proportion of the times that each patient is included in the positive subset of the selected biomarker and obtain a confidence interval for the treatment effect in the selected subset. (Simon2010_Clinical trial designs for evaluating)	
**Adaptive strategy for biomarker with measurement error**				The trial is comprised of two stages: in the first stage, patients are randomized to treatment driven by the gold-standard biomarker versus standard of care chemotherapy, while the secondary marker value is also recorded. In the second stage, the trial may switch toward use of the cheaper secondary marker if the two markers are highly concordant for predicting strategy benefit at an interim	

For peer review only

				analysis between the stages. At the trial's conclusion, the primary objective is comparison of treatment strategies with or without use of the primary or secondary biomarker. (Renfro2016_Clinical trial designs incorporating)	
**Adaptive signature design**		**Adaptive threshold design**	[…] the Adaptive Threshold design was suggested for settings in which a putative biomarker is measured on a continuous or graded scale with its threshold for detecting individuals who would benefit from the novel treatment not predefined at the initial stage of a Phase III trial. (Antoniou2016)	The difference between the main design (Adaptive Signature design) and this variant corresponds to the biomarker-positive subset. More precisely, in the main design, if there is no claim of treatment effectiveness in the entire population, then a portion of individuals is used to develop a predictive biomarker signature and the remaining portion is used to compare the treatment effect. However, in this variant if there is no claim of treatment effectiveness in the entire population, the design identifies and validates a cut-off point for a prospectively selected biomarker. Adaptations here are referred to the subgroup and there are no modifications regarding the required number of patients or randomization ratio. In this design, human samples are collected to measure a pre-specified biomarker from the entire population at the beginning of the study but the value of biomarker is not used as an eligibility criteria. (Antoniou2016)	Two analysis plans compose this approach, the so-called 'analysis plan A' and 'analysis plan B'. The first plan is identical to the strategy proposed for the Adaptive Signature design. The second plan uses a more effective method to accommodate the multiplicity issue when combining the statistical tests for the entire population and the biomarker-defined subgroup by incorporating the correlation structure of the two test statistics. (Antoniou2016)
			[…] tumor specimens are collected from all patients at trial entry, but the value of the predictive index is not used as an eligibility criteria (Simon2010_Clinical trial designs for evaluating)	Analysis plan A begins with comparing the outcomes for all patients receiving the new treatment with those for all control patients. If this difference in outcomes is significant at a prespecified significance level (α_1), the new treatment is considered effective for the eligible population as a whole. Otherwise, a second stage test is performed using the significance threshold of α_2 = 0.05-α_1. The second-stage test involves finding the cut-point b* for which the difference in outcome of the treatment versus control (i.e., the treatment effect) is maximized when the comparison is restricted to patients with predictive index scores above that cut-point. The statistical significance of that maximized treatment effect is determined by generating the null distribution of the maximized treatment effect under random permutations of the treatment labels. If the maximized treatment effect is significant at level α_2 of this null distribution, the test treatment is considered effective for the subset of patients with a biomarker value above the cut-point at which the maximum treatment effect occurred.	

For peer review only

				(Simon2010_Clinical trial designs for evaluating)	
			• […] a new adaptive enrichment design (AED)<br>• […] does not adaptively adjust the total sample size after stage 1 or the sample size in stage 2 (Diao2018)	For example, with the adaptive threshold design we assumed that a predictive biomarker score was prospectively defined in a randomized clinical trial comparing a new treatment T to a control C. The score is not used for restricting eligibility and no cut-point for the score is prospectively indicated. A fallback analysis begins as described above by comparing T to C for all randomized patients using a significance threshold α_1, say 0.03, less than the traditional 0.05. If the treatment effect is not significant at that level, then one finds the cut-point s* for the biomarker score which leads to the largest treatment effect in comparing T to C restricted to patients with score greater than s*. (Simon2010_Clinical trials for predictive)	
			The biomarker-adaptive threshold design (BATD) allows researchers to simultaneously study the efficacy of treatment in the overall group and to investigate the relationship between a hypothesized predictive biomarker and the treatment effect on the primary outcome. (Riddell2016)	With the adaptive threshold design we assumed that a predictive biomarker score was prospectively defined in a randomized clinical trial comparing a new treatment T to a control C. The score is not used for restricting eligibility and no cut-point for the score is prospectively indicated. A fallback analysis begins as described above by comparing T to C for all randomized patients a1, using a significance threshold say 0.03, less than the traditional 0.05. If the treatment effect is not significant at that level, then one finds the cut-point s* for the biomarker score which leads to the largest treatment effect in comparing T to C restricted to patients with score greater than s*. (Simon2010_Clinical trial designs for evaluating)	
				The stage-1 analysis can be based on historical or pilot studies. The enrichment in stage 2 is expected to increase power for hypothesis testing using either data from stage 2 alone or combined data from both stages. The Cox regression model for survival endpoints is employed for the AED. However, the proposed methods can be easily generalized to any other applications where a regression model is mainly used for inference. Different criteria for determination of the biomarker cutpoint based on stage-1 data are proposed. (Diao2018)	
		**Molecular**	It is a Phase III design which collects	After the collection of tissue samples from the	This approach makes the comparison of the novel

		**signature design**	tissue samples from the entire population at the start of the trial and analyse them when the study is near completion. (Antoniou2016)	entire population, all patients are randomized to either the experimental treatment or the standard treatment. The methodology is similar to the Adaptive Signature design. (Antoniou2016)	drug with the standard of care, but on a primary outcome measure which here is the overall survival using the significance level of 0.04. In case that the results show the effectiveness of an experimental treatment over the control arm, we claim the effectiveness of treatment in the overall population. Otherwise, an analysis is conducted for the identification and validation of the biomarker classifier (i.e., a combination of biomarkers), which gives the best primary outcome measure. A portion of subjects is used for the detection of a biomarker classifier and the remainder of patients for its validation. It is considered as a promising strategy without statistical considerations mentioned. (Antoniou2016)
		**Cross-validated adaptive signature design**	Similar to the Adaptive signature approach it is a Phase III frequentist trial design based on a fall back strategy in order to identify candidate biomarkers in the training set of the study and evaluate them in the validation set. (Antoniou2016)	The difference between Adaptive signature design and Cross-validated Adaptive Signature design is in terms of the methodology analysis. The former is composed of a split-sample approach, using approximately half of patients to develop the biomarker signature and the remainder of patients to validate it, whereas, the latter uses the K-fold cross validation procedure, i.e., there are K cross-validated training sets which are used to classify subjects in the corresponding K cross-validated validation sets. After the classification of all patients, we compare the experimental treatment versus the control treatment in the biomarker-positive patients (i.e., subgroup of classifier positive patients). The Cross-validated Adaptive Signature design may yield larger power but it faces the same challenges with its main design and also includes the multiplicity problem. (Antoniou2016)	
			[…] develop a predictive combination of biomarkers in a training set of the trial and consequently evaluate it in a test set (Tajik2013)<br><br>[…] extension of the adaptive signature design, which allows use of entire study population for signature development and validation. (Zhang2018_Advancing cancer)	Similar to the adaptive signature design, the initial null hypothesis is to test the benefit of the targeted therapy against the control is conducted in the overall population, which is conducted at a slightly lower significance level α_1 than the overall alpha α. The sensitive subset is determined by developing the classifier using the full population. It is done by the following steps:<br>(1) Test the initial null hypothesis of no treatment benefit in the overall population at α_1, which is a slightly lower significance level than the overall α. If this hypothesis is rejected, then the targeted therapy is declared superior to the control treatment for the overall population and analysis is completed. If the	

For peer review only

				first hypothesis is not rejected, then the following steps for signature development and validation need to be performed.<br>(2) Split study population into "k" subsamples.<br>(3) One of the "k" subsamples is omitted to form a training subsample. Similar to the adaptive signature design, develop a model to predict the treatment difference between targeted therapy and control as a function of baseline covariates using training subsample. Apply the developed model to each subject not in this training subsample so as to classify patients as sensitive or nonsensitive.<br>(4) Repeat the same process leaving out a different sample from the "k" subsamples to form training subsample. After "k" iterations, every patient in the trial will be classified as sensitive or nonsensitive.<br>(5) Compare the treatment difference within the subgroup of patients classified as sensitive using a test statistic (T). Generate the null distribution of T by permuting the two treatments and repeating the entire "k" iterations of the cross-validation process. Perform the test at α -α_1. If the test is rejected, then the superiority is claimed for the targeted therapy in the sensitive subgroup. (Zhang2018_Advancing cancer)	
		**Generalized adaptive signature design**	It uses the training set of the trial to select among candidate biomarkers and to optimize cut-points; the selected biomarker is evaluated in the test set (Simon2010_Clinical trial designs for evaluating. In Table 1)	Firstly, candidate biomarkers are selected and the cut-off points are optimized using a training set and secondly, the chosen biomarkers are assessed in the validation set. (Antoniou2016)	
		**Adaptive signature design with subgroup plots**	Adaptive Signature design with Subgroup Plots is an extension of Adaptive Signature design which has been proposed in order to add flexibility. (Antoniou2016)	It uses tail-oriented or sliding window subgroup plots in order to identify a subset of patients which is most likely to respond to a particular experimental treatment after taking into account several cut-off points of the benefit score obtained by the subgroup plots. In this way it provides broader confidence intervals of the estimated treatment benefit. (Antoniou2016)	
**Outcome-based adaptive randomisation design**			It aims to test simultaneously both biomarkers and treatments while providing more patients with effective therapies according to their biomarker profiles. (Antoniou2016)	The process starts with the biomarker profile assessment of all eligible patients and then according to the profile of each individual, the study population will be assigned to the different biomarker groups. The trial begins with equal randomization so that each treatment by biomarker subgroup is composed of at least one individual with a known disease	A requirement of the Bayesian adaptive trial design is timely measuring and reporting of the study outcomes such that the randomization probability and the posterior probability for futility monitoring can be calculated accurately on the basis of the most recent data. (Liu2015)

				control status. Next, the trial continues with adaptive randomization of patients; this is achieved by using the Bayesian probit model to calculate the posterior disease control rate. After the posterior rate is found, we define the randomization rate as the posterior mean of the disease control rate of each treatment in each biomarker-defined subgroup. The adaptive randomization process continuous until the last individual is enrolled and can stop early only in case that all treatments are dropped due to inefficacy. (Antoniou2016)	
				[…] an initial learning period within each treatment arm was used to subsequently randomize patients with increasing probability to the treatment showing the most benefit (in terms of 8-week disease control rate) within his or her marker group. (Renfro2016_Clinical trial designs incorporating)	
				Like the umbrella trial, a Bayesian marker-adaptive design may include multiple therapies and molecular subgroups. However, the efficacy of the drug is assessed in an ongoing manner through out the trial, allowing for biomarker-based adaptive randomization (i.e., changing of the randomization ratio(s) according to patient outcomes observed to date) and removal of ineffective therapies midtrial. The success of such a design requires a rapid and reliable endpoint and real-time access to all clinical and biologic data. (Renfro2017_Precision oncology)	• Requires strong predictive marker evidence<br>• Requires excellent assay performance<br>• Requires fast assay turn-around time (Renfro2016_Clinical trial designs incorporating)
			[…] Bayesian trials specifically designed to investigate differential biomarker-driven treatment effects (Renfro2016_Clinical trial designs incorporating)	Over the course of the trial, accumulating data are used to adjust the randomization probabilities to preferentially assign future patients to better-performing treatment arms. Typically, the first block of patients are randomized to each arm in equal proportion and randomization probabilities for subsequent blocks are calculated based on information accumulated prior to starting the block. (Talisa2018)	• Strong scientific rationale, and preliminary evidence for the molecular marker-drug pairing<br>• Reliable assay, with rapid turn-around times<br>• Short term, reliable endpoint to make the adaptation meaningful<br>• Sufficient infrastructure set up and real time data availability (Renfro2017_Precision oncology)
				These proposals generally start with a small sample burn-in period followed by assigning the next dose based on accumulating short term responses or outcomes or the immediately previous cohort response until the pre-specified maximum number of patients randomized is reached. In addition, the learning stage may employ longitudinal models linking the intermediate efficacy biomarker with clinical outcome, dose's response models,	[…] one must define the decision rules for adaptation upfront of study initiation, monitor the randomisation weights to avoid instable estimates, account for time dependency of the outcome (if necessary) and has to rely on a short-time outcome. (Kesselmeier2019)

For peer review only

				and/or clinical outcome dropout models. (Wang2011)	
		**Bayesian covariate adjusted response-adaptive randomisation**	This strategy which combines a Bayesian, an adaptive and biomarker classification approach aims to match patients with the most efficacious treatments by utilizing patient's biomarker information becoming available during the conduct of the clinical trial. (Antoniou2016)	The general procedure of this approach is composed of four steps according to Eickhoff et al. (2010): (i) randomly assign the first n^*>=J^* (K+1) patients to the different treatment arms where J the number of different treatment groups and K the number of biomarkers. At least one response should be observed in each of the different treatment groups before moving to the Bayesian response adaptive randomization; (ii) after each new individual has been enrolled in the study, predictive biomarker-defined groups are determined by utilizing a partial least squares logistic regression strategy (PLSLR) which can predict whether the patient can benefit from the treatment. The biomarker status is determined before the randomization; (iii) after the establishment of the biomarker status and biomarker-defined groups of each new individual, the individual is then randomly assigned into one of the treatment arms using a BCARA randomization; (iv) according to the results of the BCARA randomization the trial either stops or continues based on decision rules proposed by Eickhoff et al. (2010) [53]. The Bayesian covariate adjusted response-adaptive trial design has the ability to identify the biomarker-defined groups likely to respond to a treatment but it does not control the Type I error and in order to ensure that the identified result is true, a Phase III study should be conducted. (Antoniou2016)	
		**Bayesian hierarchical model for response-adaptive randomised design**		[…] the model incorporates a continuous monitoring for futility and a final analysis of efficacy that are conditioned on the integral biomarkers. (Barry2015)	
**Adaptive threshold sample-enrichment design**			It is a two-stage design in a Phase III setting to adaptively modify accrual in order to broaden the targeted patient population (Antoniou2016)	At the interim analysis stage, the treatment effect of a sample of patients (n1) from the biomarker-positive subset is estimated. If an improvement is seen in the experimental treatment arm which is greater than a pre-specified threshold value (i.e. the estimated treatment difference between the novel treatment arm and the control treatment arm for this subpopulation is greater than a threshold value c divided by the square root of the aforementioned sample size n1) the trial	

				continues with accrual of patients from the entire biomarker-positive subgroup and additional patients are also accrued from the biomarker-negative subpopulation; otherwise the trial is stopped for futility. At the end of the trial, the treatment effect is estimated for all subpopulations. Researchers should choose the sample size n1 so that a persuasive result can be reached when the first stage of the trial is completed. (Antoniou2016)<br>After an interim analysis separating two stages of patient enrollment, such a trial may stop for futility or efficacy, continue on as a randomized trial, or switch toward direct assignment of patients to the experimental treatment based on initially promising, but not definitive, results. (Renfro2016_Clinical trial designs incorporating)<br>[…] starts with accruing only biomarker-positive patients during the initial stage of the trial. At the end of the first stage, an interim analysis is conducted comparing the outcome of the experimental versus control treatment in biomarker-positives. If the results are not promising for the new treatment, accrual stops and no treatment benefit is claimed. Otherwise, accrual continues with recruiting unselected population. This design is a combination of an enrichment and a traditional flow, conditional on the result of the interim analysis. (Tajik2013)<br>The design consists of two stages, where in stage 1, patients are recruited in the full population. Stage 1 outcome data are then used to perform interim analysis to decide whether the trial continues to stage 2 with the full population or a subpopulation. The subpopulation is defined based on one of the candidate threshold values of a numerical predictive biomarker. The final confirmatory analysis uses data from both stages. (Kimani2018)	
**Adaptive patient enrichment design**			Adaptive enrichment designs offer the potential to enrich for patients with a particular molecular feature that is predictive of benefit for the test treatment based on accumulating evidence from the trial. (Mandrekar2015)	A pre-planned total sample size with futility stopping is considered for this two-stage adaptive design. The trial assesses the treatment effect both in the entire population and in the biomarker-positive population. (Antoniou2016)	One forewarning to apply the adaptive enrichment design is that the end point for interim analysis should be properly chosen, in that the end point should be measurable and that sufficient data are attainable to give investigators reliable guidance to move forward into the next stage. (Lin2015)

For peer review only

				In this design, all of the eligible subjects are recruited in the first stage, followed by an interim analysis to determine the study design between enrichment design and all-comer design. The sample size, end points, randomization ratio or enrichment hypothesis may also be adjusted using interim data before moving forward to Stage 2. Bayesian methods are proposed for the adjustment of randomization scheme using interim data. (Lin2015)	• Requires strong predictive marker evidence<br>• Requires excellent assay performance<br>• Requires fast assay turn-around time<br>• Requires moderate to high marker prevalence<br><br>(Renfro2016_Clinical trial designs incorporating)
				Patients are screened with the diagnostic test and those who are considered "test-positive" are eligible for the clinical trial. Eligible patients are randomized to receive either the test drug or an appropriate control regimen. In some cases, the randomization may be between the test drug and standard chemotherapy, or between standard chemotherapy alone versus standard chemotherapy plus the test drug. When there is no standard chemotherapy, the randomization may be between the test drug and best supportive care. (Mandrekar2015)	Statistically, a challenge of using adaptive accrual design relates to type I error control. There are several sources that could contribute to potential type I error inflation, including the potential enrichment of the accrual population with sample size modification as well as the adaptive selection of the hypotheses that to be tested at the final stage. Appropriate statistical correction needs to be applied to ensure type I error rate is controlled for adaptive accrual design. (Zhang2018_Advancing cancer)
				The adaptive enrichment design initially randomizes an unselected patient population to experimental versus control treatment, and if the experimental treatment effect reaches a futility threshold in the marker negative group at an interim analysis, accrual of marker-negative patients is terminated and the remaining sample size re-allocated to marker-positive patients. In that case, the primary hypothesis tested at the trial's conclusions is the treatment effect in the marker-positive subgroup. Otherwise, if futility is not reached in the marker-negative group at an interim analysis, the trial continues unselected and performs both overall and subgroup-specific tests of treatment benefit at the final analysis time point with trial-wise type I error control. (Renfro2016_Clinical trial designs incorporating)	
			[…] biomarker-based clinical trial designs with allowed mid-trial adaptation based on the results of interim analyses. (Renfro2016_Clinical trial designs incorporating)	At the interim analysis after stage 1, a decision is made about enrollment in stage 2, based on the stage 1 data. The 3 choices are to enroll the combined population, only subpopulation 1, or to stop all enrollment. Adaptive enrichment designs with >2 stages involve such choices at the interim analysis after each stage. (Rosenblum2017)	

For peer review only

			[…] initially randomizes an unselected patient population to experimental versus control treatment, and if the experimental treatment effect reaches a futility threshold in the marker-negative group at an interim analysis, accrual of marker-negative patients is terminated and the remaining sample size re-allocated to marker-positive patients (Renfro2017_Precision oncology)	[…] the trial begins with a biomarker-stratified first stage in which it accrues both biomarker-positive and -negative patients. If the results of an interim analysis comparing the outcome of the experimental versus control treatment in biomarker negatives are not promising, accrual to biomarker-negative subgroup is terminated and the second stage continues as an enrichment trial in biomarker-positive patients until the planned total sample size is reached. (Tajik2013)	
			Designs with prespecified rules for modifying the enrollment criteria based on data accrued in an ongoing trial […] (Rosenblum2017)	An interim look will be prospectively planned in a two-stage adaptive accrual design, and the adaptations will primarily be in two aspects based on the interim results: 1) The patient population to enroll at the second stage of the trial (overall or only g+); 2) The test population(s) at the final analysis (full population or marker+ population or both full and marker+ as co-primary population). (Zhang2018_Advancing cancer)	
			Adaptive designs can also be considered in order to bring the effective treatment to the right subset of patients sooner. (Zhang2018_Advancing cancer)		
			[…] two-stage adaptive enrichment design (AED) that retains some of the flexibility of the Simon design and yields a subgroup for treatment indication together with a specific test of treatment efficacy for the chosen subgroup. Like the Simon design, the proposed design does not require predefined subgroups; it allows a subgroup to be selected at an interim analysis on the basis of a prespecified collection of baseline covariates. We do require that the algorithm for subgroup selection be prespecified. The selected subgroup will be used for patient enrollment in the second stage and eventually for treatment indication. The treatment effect in the selected subgroup can be estimated using a weighted average of separate estimates from the 2 stages. It is straightforward to obtain a treatment effect estimate from the second-stage data. However, treatment effect estimation in the first stage is subject to a resubstitution bias due to the fact that the same set of data is used to select a subgroup and estimate the treatment effect in the selected subgroup. We consider the use of cross-validation and bootstrap methods to correct for the resubstitution bias. (Zhang2018_Treatment evaluation)		

For peer review only

		**Modified Bayesian version of the two-stage design**	It is a Phase III Bayesian two-stage design proposed by Karuri and Simon (2012) for the evaluation of both treatment and biomarker. (Antoniou2016)		
			A Bayesian version of the adaptive enrichment design that allows for formal specification of prior confidence in a biomarker's predictive ability […] (Renfro2016_Clinical trial designs incorporating)		
		**Multistage adaptive biomarker-directed targeted (MAT) design**	The target patient population with a specific disease etiology is identified by a biomarker (positive) and randomized into treatment arms; whereas patients with biomarker negative status in the initial screening are taken off from the study. (Gao2015)		
		**Run- in phase design**	[…] design for phase III trials with a candidate predictive pharmacodynamic biomarker measured after a short run-in period (Hong2013)	We assumed patients meeting broad eligibility criteria consist of a group of true responders (R+) who will benefit from the targeted therapy and a group of true nonresponders (R-) who will not. However, the true response class is, in general, nonobservable. We used this simple dichotomization for purposes of better understanding and quantifying the conditions when the run-in design is or is not beneficial but recognize that graded levels of sensitivity to therapy often exist. **All patients receive the new treatment for a defined short run-in period. Biomarker status will be assessed afterward, and patients will be classified as either biomarker positive (M+) or negative (M-).** The pharmacodynamic biomarker provides an (imperfect) estimate of the underlying responder status. **Depending on the strength of prior evidence, the design would either randomize only the M+ patients or all patients, stratified on biomarker status, at 1:1 ratio to receive the experimental treatment (new drug or new drug plus standard therapy) or control (standard therapy).** The objective is to test whether the experimental treatment prolongs survival compared with the control. When all patients are randomized, two statistical tests will be performed, and the thresholds of significance will be adjusted to preserve the overall study-wise type I error. The first test (test all) includes all patients at a two-sided significance level of α_{all}. If the first test is not	

For peer review only

				statistically significant, the second test (test positive) is performed for the M+ subset at a two-sided significance threshold of α_+. If $\alpha_{all} + \alpha_+$ = 0.05, then the study-wise type I error, which is the probability that either of the tests will be found statistically significant when the treatment is uniformly ineffective, will be no greater than 0.05. (Hong2013)	
**Adaptive parallel Simon two-stage design**			The design aims to test a novel treatment which possibly has a different treatment effect in the biomarker-positive versus the biomarker-negative subgroups. (Antoniou2016)	The design begins with two parallel phase II studies. During the first stage, two separate studies are performed in the biomarker-positive and biomarker-negative subgroups. Next, depending on the interim results of the first stage, the trial either stops or continues into a second stage with the enrollment from either the entire patient population (unselected patients) or from the biomarker-positive subpopulation only (selected patients). If a preliminary efficacy is observed during the first stage of the study for the experimental treatment in both the biomarker-positive and biomarker-negative subset, then additional patients from the general patient population will be enrolled in the second stage; if the interim result during the first stage of the trial shows that the efficacy is limited to the biomarker-positive subjects, then the recruitment of additional biomarker-positive patients only continues during the second stage. (Antoniou2016)	The approach assumes that there is a sound scientific rationale as to why the biomarker may potentially affect response rate. Further, it is also assumed that there is reasonable knowledge of the prevalence of the marker and that identification of subjects as marker positive or negative is well established (Jones2007)
				If preliminary efficacy based upon the first stage suggests that the drug is active in both marker positive and marker negative patients then subsequent enrollment will be unrestricted and an additional N^{un} subjects are to be enrolled during the second stage. At the end of the second stage a total of N^+ and N^-, marker positive and marker negative subjects, respectively, will have been enrolled, and of these subjects there will be a total of X_T^+ and X_T^- responders. In this setting N^+ and $N^-$are unknown a priori but based upon the known marker prevalence a reasonable value can be postulated. If based on the outcome of the first stage there is preliminary evidence that efficacy is restricted to the marker positive subgroup then enrollment of N_2^+ additional marker positive subjects continues during the second stage for a total enrollment of $N^+ = N_1^+ + N_2^+$marker positive subjects. (Jones2007)	

		**Parashar design**	An extension of the Jones design was proposed by Parashar et al. by adding go-decision rules in either the unselected population or the biomarker-positive subgroup at interim analysis. (Cabarrou2018)	As for the Jones design, it is necessary to anticipate some type of hierarchy between the two subgroups before beginning the study, and it is assumed that the response rate will be higher in the biomarker-positive than in the biomarker-negative subgroup. The study begins with the inclusion of N_1^- and N_2^+ patients, respectively, in biomarker-negative and biomarker-positive subgroups. (Cabarrou2018)	
**Multi-arm multi-stage design**	**Seamless design**		It has the ability to simultaneously compare multiple experimental treatments with the standard treatment in order to achieve more reliable results in less time as compared with separate Phase II trials to assess each novel treatment individually. (Antoniou2016)<br><br>Where there is more than one clinically important question to be addressed (which is commonly the case), a multi-arm trial approach can simultaneously and systematically test each of these approaches against the current standard of care (the control arm). (Kaplan2015)	The first stage of the trial (the Phase II stage) involves randomization within one of two arms which simultaneously compare two experimental treatments with the standard of care (control) using an intermediate outcome measure (e.g. progression free survival). The arm within which a patient is included depends on their biomarker status, for example patients positive for biomarker 1 may be randomized in arm 1 to either standard of care or experimental treatment 1 whilst patients positive for biomarker 2 may be randomized in arm 2 to either standard of care or experimental treatment 2. At the end of this first stage, an interim analysis is undertaken in each arm, comparing the experimental treatment with standard of care. Depending on the outcome of the interim analysis, accrual of patients either continues within an arm to the second stage of the trial or the accrual of additional patients stops within that arm. (Antoniou2016)	
		**Two-stage adaptive seamless design**	It uses the MAMS approach combining two separate studies into one single study and uses interim monitoring as well as multi-arm design features. (Antoniou2016)	the general procedure of this Phase II/III strategy is presented by Brannath et al. (2009) as follows: When half of individuals are recruited in the study, an interim analysis is performed in order to decide whether to accept or not a biomarker-defined subpopulation identified in a separate exploratory study. At this interim stage, a decision is also made about whether to continue accruing patients from the aforementioned biomarker-defined subset or from the entire study population. If the first case occurs, the treatment effect is assessed only in this biomarker subpopulation and if the second case happens, the treatment effect is tested in the entire population and biomarker-defined subgroup at the same time. In case that there is no identified biomarker-defined subpopulation from the separate exploratory study, the trial continues in the	According to Scher et al. (2011), formulas for sample size calculation/allocation are proposed in situations where the study endpoints are continuous, discrete, and contain time-to-event data supposing the availability of a well-established relationship between the study endpoints at different stages, and that the study objectives at different stages are the same. Ang et al. (2010) have stated that even in case that the trial stops early, a Phase III infrastructure should be developed. Such strategies have been proposed by Ellenberg and Eisenberger (1985) and Inoue et al. (2002) for evaluating the possibility to stop early or to continue to the confirmatory phase III repeatedly during the explanatory phase. (Antoniou2016)

For peer review only

				overall population using a classical group sequential design. An extension of the above approach by Brannath et al. (2009) is proposed by Jenkins et al. (2011) which can result in the rapid approval of novel treatments to the most appropriate individuals who are likely to benefit from the new drug. During the Phase II trial an interim analysis is conducted using a short-term intermediate outcome measure (i.e., survival endpoint) in order to select the population (either the entire population or the biomarker-positive patients) which will be used in the Phase III study with a long–term endpoint. Mehta et al. (2014) proposed an alternative seamless approach for subgroup selection in time-to-event-data for situations where there is no a priory assumption that a biomarker is predictive of treatment efficacy; consequently their design tests whether there is treatment effect in both biomarker-negative and biomarker-positive subpopulation separately instead of testing the null hypothesis of no treatment effect in the entire study population and in biomarker-positive subset. (Antoniou2016)	
			[…] combine the learning stage of Phase II and confirmatory stage of Phase III (Lin2015)	In the beginning of Phase II, subjects are randomized into the treatment arms of A, B, combined therapy of A and B, or control. An interim analysis is then performed to determine which active arm should be dropped. In the confirmatory stage of Phase III study, the treatment groups with only one active arm and control arm will be investigated. (Lin2015)	
			Seamless designs consolidate multiple phases into a single protocol that is designed, approved, and executed as a single trial. (Talisa2018)	After an interim analysis between the phases, which uses the shorter-term endpoint, the trial can either continue to phase III in the co-primary overall and subgroup populations, continue in the subgroup only, continue in the full population without consideration of the subgroup, or stop for futility. (Renfro2016_Clinical trial designs incorporating)	
				Initially, patients are randomized between multiple new therapies and a control. At the end of the Phase II stage, an intermediate (early) end point is employed to make a decision as to whether to continue the trial to the Phase III stage and, if so, to select the most promising experimental arms for evaluation of the definitive clinical outcome. (Freidlin2010_Biomarker-adaptive clinical trial designs)	

For peer review only

		**Adaptive design for population selection using correlated time to event endpoints**	We extend the previous methods (Brannath et al., 2009; Jenkins et al., 2011) in two aspects. First, the interim analysis is conducted by incorporating information on progression-free survival (PFS) as well as overall survival (OS). Second, we consider a scenario in which OS is calculated based on PPS, if the progression is observed before death. (Uozumi2017)		
		**Bayesian adaptive patient enrolment restriction (BAPER) approach**	Consider a two-arm randomized phase 2 clinical trial in which an experimental treatment is compared with a control treatment based on a primary endpoint of time-to-event data (e.g., PFS), and there exists a single continuous biomarker that is prospectively hypothesized to be predictive. It is assumed that the continuous biomarkers for all patients are available before randomization and that a higher value of the biomarker indicates greater improvement of efficacy if the biomarker is truly predictive. (Ohwada2016)	The objective of the trial is to identify a sensitive patient population **and make a final decision for a subsequent phase 3 trial** (i.e., no-go, go with entire population, or go with subpopulation) based on a pre-defined target efficacy level (e.g., HRD0.6), which may be provided by physicians or a clinical study team taking its clinical relevance into consideration. Two or three interim analyses are planned to narrow down the patient population to be enrolled in the next cohort of the trial, as well as to decide early termination due to futility or efficacy.<br><br>We apply a four-parameter change-point model to the relationship between the single continuous biomarker and HR and calculate the posterior distribution of the cutoff parameter of the biomarker, thus identifying the subpopulation that truly exhibits the target HR or a more efficacious HR. Using the posterior distribution, we identify the patients who are unlikely to reach the target HR and stop enrollment of such patients at the interim analysis. In addition to our proposed restriction on patient enrollment, we also incorporate criteria for futility and efficacy stopping at the interim analysis; finally, we make the following decision for the next step: no-go (futility), go for the next study with the entire population, or go for the next study with the sensitive subpopulation. (Ohwada2016)	

		**Bayesian subgroup based adaptive design (SUBA)**	[…] designs that simultaneously search for prognostic subgroups and allocate patients adaptively to the best subgroup-specific treatments throughout the course of the trial. (Xu2014)	If one treatment is inferior to all other treatments, then that treatment should be dropped from the trial. If there is only one treatment left after dropping inferior treatments, then the trial should be stopped early due to the ethical and logistics reasons. The SUBA design starts a trial with a run-in phase during which patients are equally randomized to treatments. After the initial run-in, we continuously monitor the trial until either the trial is stopped early based on a stopping rule, or the trial is stopped after reaching a prespecified maximum sample size N. (Xu2014)	
			SUBA applies a Bayesian random partition model to search for a suitable partition (clustering) of the patient space based on selected variables. (Simon2018)	SUBA can accommodate 3 independent variables, which are chosen a priori based on the specific project (described below). For each of the patients enrolled in phase 1, SUBA uses information on these 3 factors, their treatment assignment and their outcome. Based on the partition, SUBA calculates the posterior predictive probability that a future patient with specific variable values will respond to a particular treatment if the patient is assigned to the treatment. This treatmentspecific posterior predictive probability is then used to randomize the patient. If the posterior predictive probability is larger for one treatment, the patient will have a larger randomization probability to be assigned to that treatment. In other words, patients are assigned adaptively to treatments based on predictive response. The posterior predictive probability for each future patient is continuously updated when new outcomes are observed from previous patients. This allows the trial to continue the learning until the end, potentially providing better benefits for patients in the trial by giving them a larger chance to be randomized to more desirable treatments. (Simon2018)	
		**Group sequential design**	This strategy aims to find the most beneficial treatment for future patients based on their biomarker profiles, with a guaranteed probability of correct selection. (Antoniou2016)	According to an interim data analysis, sequential decisions about whether to continue the study or not, are taken. It is considered a simple approach where selection of cut-off points is not required before the conduct of the first interim analysis. (Antoniou2016)	

			[…] allows both sequential assessment across marker-defined subgroups and adaptive subgroup selection, while retaining an assessment using the entire patient cohort at the final analysis stage, possibly using established marker-based multiple testing procedures (Matsui2018)	We assume a reliable marker hypothesis where the treatment is more effective in the marker-positive than in the marker-negative patients. One-sided statistical tests are used. […] The proposed design approach is summarized in Fig. 1. This can be viewed as concurrent subgroup-focused trials with a futility stopping rule in the marker-negative subgroup and a superiority stopping rule in the marker-positive subgroup. In case I, both boundaries are crossed, and the trial is stopped with a conclusion of efficacy in the marker-positive subgroup. In case II, only the superiority boundary is crossed, and there is sequential testing in the marker-negative subgroup. In cases III and IV, the marker-positive subgroup or the overall population is adaptively selected for the final analysis depending on whether the futility boundary is crossed in the marker negatives. In case IV, the subgroup data are combined for the final analysis. Thus, the possible complexities in performing an overall test at the final analysis in case of early stopping in some subgroup is avoided by restricting the implementation of the analysis using all patient data to only the case with no early stopping in both subgroups. Extension to multiple interim looks is possible, but we suppose a single interim analysis within subgroups for ease of presentation and practical application.<br><br>The marker-positive cohort is designed as if it were an enrichment trial. This is sized for large, but slightly conservative effects for the new treatment. The marker-negative cohort is designed as if it were a second trial in the sequential enrichment approach. This is because the chance to evaluate this cohort solely when the treatment effect is significant in marker-positive patients is also embedded in our approach, not sequentially, but concurrently. (Matsui2018)	The interim analysis for superiority in the marker-positive patients, deemed most likely to benefit fromthe treatment, is to detect substantially large treatment effects and to quickly deliver the treatment to such patients. Although futility stopping rules can also be introduced in this subgroup, we propose no specification of such rules and no adjustment on the final analysis. In any case, futility stopping for marker positives would lead to the termination of the trial under the marker hypothesis. On the other hand, for marker-negative patients, a futility stopping rule would be warranted from an ethical perspective due to presumably limited treatment efficacy in marker negatives under the marker hypothesis. We propose a monitoring plan that accounts for the two possible errors: (i) futility stopping even when treatment has, in truth, a minimum effect size of clinical importance and (ii) continuing the trial for the marker negatives even when there is no treatment efficacy. In addition, we could introduce a superiority stopping rule, but we do not consider this option because large treatment effects are generally implausible for marker negatives under the marker hypothesis. When there is not sufficient evidence for early stopping in both subgroups (case IV in Fig. 1), an overall test is a simple but most effective choice in detecting an average treatment effect in the overall population at the final analysis. Alternatively, when the marker hypothesis is deemed strong, hierarchical tests may be used, such as a fixed-sequence procedure that first tests treatment efficacy in the marker positives, followed by testing in the marker negatives if the first test is significant. Otherwise, a split-alpha procedure that allocates the alpha to be spent between a test in the markerpositive subgroup and one in the overall population may be a reasonable choice. The significance levels of all statistical tests are determined to preserve a study-wise alpha level of 0.025 based on the joint null distribution of the test statistics for the marker-positive and marker-negative subgroups and the overall population across different analysis stages, that is, the global null hypothesis. We do not consider an alpha control under another possible null hypothesis, where the treatment is efficacious in marker positives, but not in marker negatives. (Matsui2018)
**Stratified adaptive design**			It is alternative approach to dealing with stratification in a phase II setting and aims to demonstrate whether an experimental treatment (a control arm is not included,	The first stage is consisted of an interim analysis where the response rate is estimated in the biomarker positive and biomarker negative subgroups separately. The trial then	

For peer review only

			thus it's about a single arm approach) is beneficial for at least one biomarker-defined subgroup rather than the entire study population. (Antoniou2016)	enters a second stage and depending on the results of the interim assessment, accrual continues either from the entire patient population if there is treatment efficacy of both biomarker-defined subgroups, or from one of the distinct biomarker subpopulations only in which treatment efficacy has been observed. (Antoniou2016)	
			Tournoux et al. proposed a stratified adaptive Fleming two-stage design not requiring any assumption prioritizing the two pre-defined subgroups. (Cabarrou2018)	It is assumed that the ratio between the number of patients in the biomarker negative and biomarker-positive subgroups is constant and is defined by ω=N+ / N-. This design provides stopping rules for both activity and futility at the end of the first or second stage. Heterogeneity between the two subgroups is also tested at each stage at level which can be set between 0 and 1. (Cabarrou2018)	
**Tandem two stage design**			It is composed of 2 optimal trials in a Phase II settings. (Antoniou2016)	In this design, a predefined biomarker is assumed. In the first stage of the trial, patients from the entire population enter the trial irrespective of their biomarker status. An interim analysis is then undertaken and if a sufficient number of events (defined in terms of clinical benefit rate or response rate) have been observed during the first stage, the study proceeds to a second stage whereby further patients are accrued from the unselected population to establish the benefit rate more precisely in unselected patients. However, if an insufficient number of events have been observed during the first stage, rather than stopping accrual for futility, a second trial commences whereby its first stage involves continued accrual of biomarker positive patients only. An interim analysis is then conducted and if a sufficient number of events have been occurred, this second trial continues into a second stage of biomarker positive patient accrual. Otherwise, if an insufficient number of events have occurred, the predefined biomarker is rejected. (Antoniou2016)	The sample size for this approach is calculated with the same rules as a classic two-stage or Bayesian phase II design. (Antoniou2016)
**Platform design**			To study multiple-targeted therapies in the context of a single disease in a perpetual manner, with therapies allowed to enter or leave the platform on the basis of a decision algorithm (Heerspink2018_New clinical trial designs)	First, a shared master protocol is used for common elements of the multiple individual trials within the platform with relatively subtle trial design differences due to unique individual drug characteristics reflected in study-specific appendices, enabling sharing of clinical trial documents and procedures among trials. This facilitates clinically consistent trial conduct and increased efficiency. Second, the platform	

				approach commonly involves some form of adaptive design to assign patients to the most promising drugs on the basis of new data accrued during the trial. In addition, the platform trial is not static, but it is flexible, which means that new promising drugs can enter the platform, while other drugs can be dropped due to lack of efficacy or adverse events. Declaring superiority or futility can be assessed continuously on the basis of data as they are accrued during the trial and is another adaptive design element (Heerspink2018_Trial design innovations)	
				[…] patients are assigned to a treatment arm based on concentration levels of a set of predictive markers for the available treatment options. Markers and renal function parameters are used for patient monitoring and identification of responders who remain in the assigned treatment arm, whereas nonresponses are shifted to the next-best suitable treatment based on marker profiles. (Perco2019)	
			[…] in platform trials (or ˜standing trials") patients with a specific tumor type are randomized to a common control arm or one of the several experimental arms that enter and exit the trial after interim analyses aimed to evaluate the efficacy or futility of each targeted treatment through Bayesian method. (Leonetti2019)	Platform trials are often Bayesian in nature, utilizing Bayesian decision rules based on posterior or posterior predictive probabilities to eliminate or graduate treatments within certain cohorts. (Renfro2018_Definitions and statistical properties)	
			[…] designs that evaluate multiple treatments simultaneously […] (Mazzarella2020)	Initially the treatments are randomized with equal weights to the patients of a stratum. As data accumulates, the randomization weights change to favor assignment of drugs with higher within-stratum response rates. The endpoint used must be observed early enough to enable adaption of randomization weights. (Simon2017_Critical review)	
			Platform trials, also referred to as multi-arm, multi-stage (MAMS) design trials, are trials that evaluate several interventions against a common control group and can be perpetual. This design has pre-specified adaptation rules to allow dropping of Ineffective intervention(s) and flexibility of adding new intervention(s) during the trial. (Park2019)	In a platform trial, the feedback loop involving collecting data, updating the Bayesian statistical model and updating RAR weights is modified to enable new arms to be added, and old arms to either be dropped or "graduate" to the next phase of testing (Talisa2018)<br>In both umbrella and platform trials, each arm is typically enriched with a biomarker and patients are enrolled and assigned to a	

For peer review only

			Another type of master protocol described in the literature is the platform trial (or "standing trial"), a generic term for a randomized design with a common control arm and many different experimental arms that enter and exit the trial as futility or efficacy are demonstrated, often according to Bayesian decision rules. (Renfro2017_Statistical controversies)	cohort based on their biomarker status. Platform trials may be distinguished from umbrella studies in that they are thought to incorporate more adaptationsâ€"as responses are observed, patients are algorithmically allocated to specific treatment arms according to the best match between treatment effect and their tumor type. Experimental drugs drop out for lack of efficacy or they can "graduate" for efficacy testing depending on the observed response. Randomization is adapted such that the number of patients needed to determine efficacy across biomarker groups is minimized (Cecchini2019)	
			Lastly, a platform trial may be generally defined as a type of master protocol in which sub-trials continually enter and exit, where the latter may occur due to futility or due to graduation of a marker-treatment combination to further study. (Renfro2018_Definitions and statistical properties)		
			A platform trial is a single histology randomized phase II clinical trial involving multiple biomarkers and multiple drugs. Rather than assuming that we know which drug is appropriate for which biomarker stratum, randomization among drugs is used in the platform trial. (Simon2017_Critical review)		
			[…] the adaptive platform trial is capable of being a platform for testing experimental treatments in a perpetual manner via a common master protocol, by dropping treatments lacking efficiency and adding new treatments going into the future. (Talisa2018)		
			Other trial designs include platform trials, which use a single analytic technique, such as NGS (next generation sequencing), to identify genomic or other biomarkers in tumors with multiple histologies; (Tsimberiou2020)		
			A parallel group design with a shared control evaluates two or more investigational treatment arms relative to a control arm in the same tumour type in a single clinical trial. (Verweij2019)		
			Platform trials randomize patients to different cohorts and take umbrella studies a step further by following algorithms to adapt and add new therapies or drop existing therapies from an ongoing study. (Cecchini2019)		

For peer review only

			[…] multi-arm because many treatment approaches can be tested simultaneously; multi-stage because prespecified interim analyses can be used to stop recruitment early to arms showing insufficient evidence of activity. (Gilson2017)		
			[…] a type of adaptive trial in which multiple treatment arms are simultaneous studies,and interim analysis allows early termination of various arms due to futility or lack of efficacy (Van Norman2019)		
			A platform trial is defined as a trial using a single master protocol and research infrastructure to simultaneously evaluate multiple interventions and/or disease subpopulations in multiple substudies. Platform trials gain efficiencies from shared control groups, adaptive borrowing of information from similar groups of patients, and shared infrastructure and governance. (Semler2020)		
			[…] study multiple targeted therapies in the context of a single disease in a perpetual manner, with therapies allowed to enter or leave the platform on the basis of a decision algorithm. (Alexander2019)		
	**Open adaptive platform**		The trial is "open" with respect to adding new treatments to replace ineffective treatments during the trial. (Saville2016)		
		**Randomised, embedded multifactorial adaptive platform (REMAP)**	Randomized, embedded, multifactorial adaptive platform (REMAP) trials utilize all of the features of a perpetual adaptive platform trials like I-SPY 2 or GBM-AGILE, the key distinction being that a REMAP trial is executed directly within clinical practice through the electronic medical record. (Talisa2018)		
		**Bayesian Adaptive Platform Trial**		As the trial progresses, randomization probabilities adapt on the basis of accumulating results using Bayesian estimation of the biomarker-specific probability of treatment impact on progression-free	[…] uses biomarker subgroup-specific randomization probabilities to allow data generated during the trial to drive the biomarker specificity of arm assignments. (Alexander2019)

For peer review only

				survival. Treatment arms may drop because of low probability of treatment impact on overall survival, and new arms may be added. (Alexander2019)	
	**Closed platform**		The trial is a ‘‘closed’’ platform trial, meaning no additional treatments are added beyond those included at the start of the trial. (Saville2016)		
**Basket design**			Evaluates the effect of a particular targeted therapy on a particular genetic or molecular aberration across cancer organ types. Variant of indication finder but the therapy is not evaluated for its off-target effects. (Berry2015)	Molecular profiling-based targeted therapies are prescribed to treat patients with advanced metastatic solid tumours that are usually incurable or not controlled by standard treatments. NCI-MPACT randomly assigns patients with a mutation in a specific genetic pathway to either a targeted therapy for that pathway or a treatment not known to be pathway specific. (Gómez-López2017)	[…] basket trials should be stratified by histology, taking into consideration the reported frequencies of the genomic event. (Garralda2019)
			In this framework, patients with different tumor histologies but who harbor the same molecular aberration receive a matched targeted in the context of expansion cohorts of a Phase 1 trial or as a separate Phase 2 trial, with efficacy as the primary endpoint. (Dienstmann2015)		
			This is an innovative, histology agnostic trial design, where patients with tumours of different histologies can be enroled in the study protocol on the basis of the presence of a commonly shared molecular aberration. (Fadoukhair2016)		[…] the lower the prevalence of the biomarker, the larger the effect size needs to be for the trial to be meaningful (Janiaud2019)
			Basket trials include patients with different tumour types with a common molecular alteration who are treated with the same matched therapy (Garralda2019)	Commonly, basket trials are early stage, single-arm, phase II, proof-of-concept trials where in each basket or cohort is itself a single-arm trial studying a preliminary target-response hypothesis. Such cohorts are generally small (say, 20-30 patients) and only powered to detect strong signals of activity meant to motivate further study in a randomized context, though toxicity is often a key secondary endpoint in sub-studies where drug tolerability is not yet well understood. Each arm may further be constructed as a single-stage, two-stage, or multi-stage design, and futility-stopping rules may be incorporated. (Renfro2018_Definitions and statistical properties)	From a statistical perspective, the efficiency of basket trials comes from pulling data across all tumor subgroups to estimate the treatment effect. However, this pooled approach only works well when response to the therapy is relatively homogeneous across all tumor subgroups. Heterogeneous responses across tumor subgroups may lead to potential bias and/or inflation of the false-positive rates. A new calibrated Bayesian hierarchical model has recently been proposed to better control the type I error rate in basket trials. (Le-Rademacher2018)
			To study a single-targeted therapy in the context of multiple disease or disease subtypes (Heerspink2018_New clinical trial designs)	Patients are assigned a regimen that is expected to be active for tumors containing that alteration. Often this expectation is based on knowledge of the target of the drug and its role in the progression of the disease as well as previous approval of the drug, or a similar drug, for patients with the same genomic	

For peer review only

				alteration in some specified histology. In this case, the basket trial is a phase II screening trial for off-lable use of the drug in patients with the same genomic alterations for which it was approved. (Simon2017_Critical review)	
			The distinguishable feature of basket trials is their inclusion of multiple tumor types and cancer histologies, and the term histology independentâ is often used to characterize this feature. The different tumor types can express the same mutation or different ones and are targeted by either one unique therapy or biomarker-specific therapies. (Janiaud2019)	Eligibility depends on the presence in the tumor of a specified type of genomic alteration. A few multidrug basket trials have involved randomization to a test drug that targets a mutation in the patient's tumor or to a control drug. The use of randomization in a multidrug basket trial permits the trial to test the general policy of trying to match the drug to the genomics of the tumor. (Simon2016_Genomic alteration)	• Requires strong predictive marker evidence<br>• Requires excellent assay performance<br>• Requires fast assay turn-around time (Renfro2016_Clinical trial designs incorporating)
			Basket trials assess the effectiveness of a candidate drug based on the mechanism rather than the underlying cancer type. (Joshi2018)	For each drug studied in a basket design, all of the patients generally share a common mutation, but have different primary disease sites. The standard phase II designs used for most basket clinical trials ignore this heterogeneity and pool all patients containing the same actionable mutations for analysis. (Simon2018_New designs for basket clinical trials)	• By adjusting the decision rules or sample size within each basket, investigators can limit the overall false-positive rate.<br>• […] the use of statistical modeling can enable efficacy information to be shared among the baskets, improving efficiency and thereby theoretically allowing for enrollment of fewer patients.(Tao2018)
			Basket trials usually test the effect of one drug in a single/multiple arms of cancer patients who share a specific biomarker or molecular aberration, regardless of histology or organ involvement. (Leonetti2019)	In this design, individual histologic subtypes (indications) are grouped together each with its own control group. A shared control group may be used for indications with a common standard of care. Single arm designs using a concurrent registry control may be considered. Concurrent registries control for disease stage migration (the process by which progressively improved sensitivity of diagnostic techniques translates over time into patients with less disease burden being assigned to a given disease stage) and for progressive improvements in outcome due to improved supportive care, but do not control for patient selection (the ability and tendency of physicians to select patients who will do well, inflating the results on non-randomized studies). The use of registry data should be pre-agreed with health authorities.<br>Each indication cohort would be sized for accelerated approval based on a predetermined surrogate endpoint (i.e. response rate, RR, or progression free survival, PFS) reasonably likely to predict	• In order for a confirmatory basket trial to meet acceptance from health authorities, it will be necessary for the false positive rate of the pooled analysis to be rigorously controlled.<br>• […] we recommend that the trial include a testing platform such as sequencing which may identify other options for ineligible patients. (Beckman2016)

For peer review only

				clinical benefit (i.e. overall survival, OS).<br>The false positive rate for the surrogate would be pre-agreed with health authorities.<br>Effect sizes of benefit judged by hazard ratio (or by percentage improvement in median) are typically larger for surrogate endpoints compared to OS, and larger benefits can be detected with smaller sample sizes. Therefore, multiple indication cohorts can generally be pooled into a basket study of comparable size to a standard confirmatory study.<br>Tumor indications failing to meet the surrogate hurdle for accelerated approval would be "pruned"(removed from the basket). To adjust for inflation of the false positive rate of the final pooled analysis by "random high bias" due to selective pruning (please see random high bias, pruning of indications, and the false positive rate below), a prospectively designed adjustment would lower the nominal false positive rate (false positive rate before adjustment for random high bias) for the remaining indications. This adjustment amounts to a statistical penalty for using information within the study for adaptation.<br>Additional indications may be pruned based on external data such as maturing early stage data involving the definitive clinical benefit endpoint (Figure 3), or data from other agents in the class. Pruning based on external data does not inflate the false positive rate of the pooled analysis, and does not incur a statistical penalty. To maintain the power of the pooled analysis after pruning, a sample size adjustment for the remaining indications may be required.<br>(Beckman2016)	
			Basket trial designs offer the possibility to include multiple molecularly defined subpopulations, often across histology or tumor types, but included in one cohesive design to evaluate the targeted therapy in question. (Mandrekar2015)		Adjusted posterior probabilities were computed in accordance with the trial's reported design strategy, for which hypothesis testing assumed identical null response rates for all organ sites. This assumption, if violated, would preclude implementation of basket trials devised to pool patients harboring common molecular tumor types arising from disparate clinical subtypes.<br>(Hobbs2018_Statistical challenges)
			[…] trials designed to evaluate single drugs across multiple populations (Mazzarella2020)		
			[…] evaluate whether a certain actionable mutations of interest (aMOI) or biomarker signature is predictive of response to a targeted drug regardless of the tumor of origin. (Moore2016)		

For peer review only

			Basket trials are a histologically agnostic trial design which recruit patients whose tumours contain a specific genomic aberration of interest. (O'Brien2017)		
			Basket trials refer to designs in which a targeted therapy is evaluated on multiple diseases that have common molecular alternations (Park2020)		
			[…] marker-specific but tumor agnostic and conducted in parallel without analyses across protocols (Renfro2016_Clinical trial designs incorporating)		
			A basket trial is similar to an umbrella trial in that there may be a common genetic screening platform, multiple study therapies, and multiple molecular subgroups. However, a basket trial typically enrolls multiple disease types to each of several marker-based cohorts, and these are conducted under a single protocol. (Renfro2017_Precision oncology)		
			A basket trial is a master protocol for which patient eligibility is defined by the presence of a particular biomarker or molecular alteration rather than a particular cancer type. Basket trials are predicted on the hypothesis that the molecular characterization of a particular tumor predicts response to a matched (tar geted) treatment to a greater extent or independent of tumor histology. (Renfro2017_Statistical controversies)		
			Basket trials (also referred as pan-tumor or tissue-agnostic trials) are designed to evaluate the effect of a drug that targets a single mutation or a specific pathway in various tumor types. These trials are simple, including specific treatment arms for various tumors of origin and location "baskets" or complex, evaluating multiple drugs across selected genetic alterations in various tumor types (Said2019)		
			Basket trials are focused on the underlying target and not the disease or clinical syndrome per se. (Shah2017)		
			In contrast to umbrella and platform trials, Basket trials are not focused on patients with a single disease histology. Basket trials are focused instead on patients with a single genomic alteration or class of		

For peer review only

			alterations. (Simon2017_Critical review)		
			[…] patient eligibility is based on a defined genomic alteration rather than on primary site. Basket trials are phase 2 trials. They can be nonrandomized or randomized and include a single drug or multiple individual drugs (Simon2016_Genomic alteration)		
			[…] patient eligibility is based on a defined genomic alteration rather than on primary site. (Simon2018_New designs for basket clinical trials)		
			"Basket trials" test whether a drug is effective in patients with specific genetic alterations regardless of their disease of origin. (Soldatos2019)		
			Unlike most clinical trials, which test a drug against a specific cancer type, the central organizing principle of a basket trial is themolecular alteration. The term basket arises from each collection of patients that harbors a particular mutation. (Tao2018)		
			A basket trial is a histology-independent design where each sub-trial enrols multiple tumour types (the basket) with one common genetic mutation. (Verweij2019)		
			[…] innovative, histology-independent trial design, in which patients with cancer diagnoses of different histologies can be enrolled in the study protocol based on the presence of a specific molecular aberration. (Zardavas2015)		
			Basket or a bucket trials address a single targeted agent or subgroup across multiple histologic indications, the premise being that the fundamental classification of cancer is molecular, not histologic, and that core molecular signatures will be common across multiple histologies. (Beckman2016)		
	**Randomised basket design**		A few multi-drug basket trials have been conducted which involve randomization to either a test drug which targets a mutation in the patient's tumor or to a control drug (Simon2018_New designs for basket clinical trials)	With randomization the trial may test the general policy of trying to match the drug to the genomics of the tumor. The null hypothesis here relates to a matching policy for a given set of drugs and genomic alterations used in the study. This policy is also determined by the type of genomic characterization performed and by the "rules" for matching drug to tumor.	

For peer review only

				Rejection of the null hypothesis provides a proof of principle that matching can be useful overall but that null hypothesis is specific for the genomic alterations and the drugs on which the study is based. (Simon2018_New designs for basket)	
				[…] in a randomized controlled basket trial, each individual tumor indication has its own control group. A shared control group may be used for indications with a common standard of care as appropriate. (Chen2016)	
	**Non randomise d basket design**				
		**Bayesian basket design**	[…] a different kind of Bayesian design for evaluating the response probabilities for the primary sites included in a basket trial of a drug. (Simon2018_New designs for basket)	At any interim analysis one can compute the posterior probability of activity (i.e. pj=phi) for each of the stratum. If that posterior probability is too small, one may close accrual to that stratum. If that posterior probability is very large, one might wish to proceed with the next stage of development of the drug in that stratum. One might wish to cap the total accrual to the trial, accepting that drug evaluation for some strata of very low prevalence may remain uncertain. (Simon2018_New designs for basket)	
			[…] flexible design that could accommodate varying hypotheses while making pre-trial choices explicit. (Alexander2016)	We generated a procedure that utilizes prior knowledge of biomarker information by quantifying the belief in the strength of the biomarker-effect linkage and combined the procedure with a Bayesian adaptive randomization algorithm. (Alexander2016)	
				In this design, a Bayesian approach is used to model the response probabilities for the various histologic strata, and two hypotheses are considered: (1) the response probabilities for a particular targeted agent are equal across the corresponding histologic strata, and (2) the activity of the drug is independent across these strata. (Ou2019)	
			[…] a design to support multiarm biomarker-driven trials that is flexible by allowing several treatments with varying biomarker hypothesis strengths in the same framework. (Trippa2017)	Bayesian basket (BB) design evaluates multiple overlapping biomarker subgroups and associated experimental therapies. It starts with explicit a priori estimates regarding the predictive utility of a biomarker for each experimental arm and then learns during the trial, thereby generating valuable information about the biomarker while providing the efficiencies of biomarker-selected clinical trials.	

				(Trippa2017)	
		**Sequential basket trial design with Bayesian monitoring rules**		[…] the sequential design strategy uses interim analyses based on the multisource exchangeability modeling (MEM) approach to identify exchangeable metabaskets and terminate enrollment to ineffective subtypes. (Hobbs2018_Bayesian basket trial)	
		**Bayesian latent subgroup trial (BLAST) design**	The BLAST design makes the interim go/no-go treatment decision in a group sequential fashion for each cancer type based on accumulating data. (Yuan2018)	Conditional on the latent subgroup membership of the cancer type, we jointly model the binary treatment response and the longitudinal biomarker measurement that represents the biological activity of the targeted agent. (Yuan2018)	
		**Bayesian hierarchical adaptive design**	Hierarchical modeling allows information about the treatment effect in one group to be “borrowed” when estimating the treatment effect in another group. (Berry2013)	In effect, the estimate of treatment effect in each group is shrunk toward the overall mean. The amount of shrinkage depends on the results, including the relative precision of estimates in the various groups.<br>In this design, the four patient groups are considered together in a single, integrated trial, and a Bayesian hierarchical model borrows information across the groups. (Berry2013)	
**Basket of basket design**			The BoB study is testing therapies in multiple disease settings/genetic contexts, encompassed by the development of companion diagnostics based on specific biomarkers in these genetic contexts, including circulating tumour DNA (ctDNA) analysis as a way to select patients for any of the tested drugs and thus increase the efficacy of treatments. (Garralda2019)	The study consists of two parts: (a) I-Profiler will allow the molecular characterization of tumours from patients with metastatic or recurrent solid tumours using a new profiling tool and select the most suitable treatment for these patients; and (b) I-Basket is a multimodular basket trial, with different cohorts for genomically selected populations. (Garralda2019)	
				First, the patient’s tumour (biopsy, plasma) is molecularly profiled by various multiplexed assays. Cancer patients with an appropriate molecular profile can then participate either in industry sponsored basket trials or in iBasket, a multi-modular investigator-initiated basket protocol. Modules can be added or dropped based on the results and may have different statistical designs (Bayesian, adaptive). Each module has individual arms with genomically selected patient populations. (Verweij2019)	

For peer review only

**Umbrella design**			Patients with exactly one of the targeted biomarkers are assigned to the associated sub-study evaluating an investigational therapy targeted against that aberration. For patients with more than one of the targeted biomarkers, assignment is randomized between the sub-studies they are eligible for using an algorithm that gives more weight to studies with lower prevalence biomarkers. Patients whose tumors alterations don't fall into any of the available matched drug-biomarker sub-studies are assigned to a non-match sub-study. Therefore all screened patients who satisfy the clinical eligibility criteria have a study in which to enroll. (Ferrarotto2015)	The sample size for each sub-study is determined based on the biomarker prevalence, maintaining all other design parameters the same across sub-studies. (Ferrarotto2015)	1. Consistency of biomarker assay across sites is important<br>2. Planning requires wellcoordinated efforts among members of multidisciplinary team<br>3. Often needs international partnerships to make it feasible (Le-Rademacher2018)
			An umbrella trial is a master protocol for which the patient's eligibility is defined by the presence of a tumour type that is substratified according to specific molecular alterations matched to different anticancer therapies. (Garralda2019)	Within a conventionally defined disease (eg, diabetic kidney disease [DKD]), various biomarker-based subgroups are defined and different drugs are tested in these subgroups. This approach supports individualizing treatments and personalized medicine. (Heerspink2018_New clinical trial designs)	The randomization is adaptive, which means as certain subtypes respond better to a certain arm, the randomization probability for a patient with that subtype being randomized to that arm increases. In the same manner, if a certain subtype has no responses to a certain arm, the randomization probability of that arm for that subtype decreases and may even go to 0 if the arm is completely dropped for that subtype. (Moore2016)
			To study multiple targeted therapies in the context of a single disease. (Heerspink2018_New clinical trial designs)	In an umbrella trial design, patients are first screened for and assigned to a specific biomarker subgroup. Patients in each subgroup are then assigned to one of the therapies specifically targeting the biomarker they harbor. Some umbrella trials allow inclusion of a subgroup of patients with no actionable biomarker. Each of these biomarker subgroups forms a substudy of the overall trial (Le-Rademacher2018)	***Refers to both umbrella and basket design:***<br>• Careful evaluations of the pre-existing clinical evidence and underlying biologic assumptions are required to ensure that there is a biologic plausibility for the targeted interventions<br>• Accuracy of biomarker tests is important; however, because all medical tests will have some degree of inaccuracy, it is important to account for inaccuracy (ie, false-positive rates) in the trial planning stage to avoid underpowering the trial<br>• If there are multiple tumor types involved, the accuracy of biomarker tests should be similar between these tumors<br>• The biospecimen collection process should be easy, and relatively uniform high biospecimen quality and biospecimen yield must be achievable, especially for basket trials that have multiple diseases<br>• Prevalence of the biomarker(s) used should be anticipated with possible recruitment challenges<br>• The sample size calculations for umbrella

For peer review only

					trials, conversely, may be done for each of the subgroups because there are multiple targeted interventions being evaluated in umbrella trials<br>• Targeted intervention strategies rely on predictive risk factors that determine whether the patient will respond to a given intervention<br>• Use of randomization and a control group with adequate sample size can determine whether the risk factor is predictive or not<br>• If randomization is not feasible, statistical adjustments can be made. However, there are issues with making statistical adjustments with smaller data sets<br>• If there is adequate sample size, it is important to note that statistical adjustments can only account for measurable factors (Park2020)
			The umbrella design tests multiple targeted therapies in different biomarker-matched subgroups of patients, all of whom present the same tumor type or cancer histology. (Janiaud2019)	Patients are screened for a specific set of biomarkers and assigned to a biomarker-driven substudy (targeted design) if it is determined that they have one of the target biomarkers. (Mandrekar2015)	• Requires excellent assay performance<br>• Requires fast assay turn-around time<br><br>(Renfro2016_Clinical trial designs incorporating)
			Umbrella trials take patients with the same type of cancer, and assign them to treatment arms based on unique mutations (Joshi2018)	• Risk factors are used to stratify patients into multiple subgroups (patient stratification);<br>• Umbrella trials have multiple interventions, with intervention assignment being determined based on their risk factor;<br>• Similar to basket trials, intervention assignment may or may not be determined using randomization;<br>• Compared with basket trials, it may be easier to pick the choice in the control group for umbrella trials because there is one disease being studied;<br>• The existing standard of care (or placebo, if there is no established care) for the disease being studied may be used as the control for all of the subgroups (Park2020)	

For peer review only

			Umbrella trials select on the basis of a tumor type or histology […] (Lam2018_Accelerating therapeutic)	In an umbrella trial, patients with tumors from the specified cancer type are centrally screened and assigned to one of several molecularly defined subtrials where they receive (or perhaps are randomized to) a matched targeted treatment. In such trials, the relevant markers are regarded as refinements of (rather than replacements of) tumor type. (Renfro2017_Statistical controversies)	In an umbrella trial, the opportunity for pooling is across substudies defined by different biomarkers. (Yee2019)
			[…] umbrella trials evaluate multiple targeted therapies in a single-tumor type. (Lam2018_Master protocols)		• In umbrella trials, in which different experimental treatments in different biomarker subgroups within the same protocol are evaluated, an overarching statistical design that is common to all treatment arms can be deployed.<br>• […] rates of recruitment to each cohort can vary dramatically requiring interim analyses at multiple time points. (Blagden2020)
			Umbrella trials enroll patients with a single type or class of tumor. After central screening, patients are assigned to one of the many sub-trials on the basis of their molecular alteration, where they are treated (or can be treated, when randomized) with a matched targeted compound. (Leonetti2019)	In the umbrella design a separate enrichment trial is conducted for each biomarker stratum. The enrichment design for a given stratum uses as the test regimen a drug expected to be active for the alteration defining that stratum. (Simon2017_Critical review)	Thus, an umbrella trial consists of multiple substudies, each with independent subgroups of patients receiving different therapies and with the option of assuming different statistical parameters for independent designs. The substudies, however, exist under an overarching master protocol that uses a common infrastructure for screening and treatment assignment to reduce the cost and time associated with enrollment to unrelated and often sequential biomarker-informed studies. (Ou2019)
			Umbrella trials include a central infrastructure for screening and identification of patients, and focus on a single tumor type or histology with multiple subtrials, each testing a targeted therapy within a molecularly defined subset. (Mandrekar2015)	As with a basket trial, the tumor molecular screening can be performed as part of the trial or in the community. Any subtrial can be a single-arm trial designed to evaluate the efficacy of a targeted agent, or a randomized trial with a standard-treatment control arm (which could be observation). Unlike basket trials, patients without a target match in an umbrella trial can easily be put on a randomized subtrial of 2 relevant treatments for the histology. However, because patients with the designated alterations have been excluded from the nonmatch subtrial, there may be some question as to what population the results will generalize. (Yee 2019)	
			[…] trials designed to evaluate […] multiple drugs on a single population (Mazzarella2020)		
			Use of adaptive randomization and a common platform design is revolutionizing how we screen new drugs. When this strategy is applied to one tumor type with		

For peer review only

			multiple different sub studies, we are describing an umbrella trial. (Moore2016)		
			Umbrella trials, in contrast to basket trials, recruit patients with one histological diagnosis, but then allocate patients to specific arms within the trial based on the presence of specific molecular alterations in their tumours. (O'Brien2017)		
			Umbrella trials, on the other hand, evaluate multiple targeted therapies for a single disease that is stratified into subgroups by molecular alternation. (Park2019_Systematic review)		
			Umbrella trials, conversely, are prospective clinical trials that test multiple targeted interventions for a single disease based on predictive biomarkers or other predictive patient risk factors. (Park2020)		
			In an umbrella trial, a common genomic screening platform and central screening infrastructure are used to assign patients to unique marker-enriched protocols. (Renfro2017_Precision oncology)		
			[…] an umbrella trial generally restricts enrollment to a single type or class of cancers (Renfro2017_Statistical controversies)		
			An umbrella trial is another type of master protocol where patients with a common disease type (e.g., advanced non-squamous cell lung cancer) are enrolled to parallel cohorts or sub-trials that are similarly marker-driven. In this instance, the umbrella "over" the various sub-trials is the larger disease population from which the marker-based cohorts were derived. Umbrella trials may include phase II or phase II/III trials, wherein the individual marker-specific sub-trials or cohorts may be either single-arm studies of paired targeted agents, or randomized studies comparing targeted agents versus placebo or standard of care. (Renfro2018_Definitions and statistical)		
			In an umbrella trial design, a variety of targeted treatments are tested in parallel. (Shah2017)		
			In the umbrella design a separate enrichment trial is conducted for each		

For peer review only

			biomarker stratum. The enrichment design for a given stratum uses as the test regimen a drug expected to be active for the alteration defining that stratum. (Simon2017_Critical review)		
			enroll many marker-defined cohorts in parallel under the "umbrella" of one disease area (Simon_clinical trial designs)		
			An umbrella trial is restricted to patients with a single primary site of cancer but uses different drugs to target patients with different genomic alterations. (Simon2016_Genomic alterations)		
			Umbrella phase 3 designs consist of a combination of several enrichment designs conducted with a common genomic alteration testing infrastructure […]. (Simon2016_Genomic alterations)		
			Umbrella designs involve several molecularly targeted test drugs and a single primary site population of patients. (Simon2018_New designs for basket)		
			These protocols generally offer multiple therapeutic options matched to the patient's individual tumor genome. (Tao2018)		
			Umbrella trials involve a single histology and different treatments based on the genomic alterations in patient subgroups. (Tsimberidou2020)		
			An umbrella trial evaluates the efficacy of different targeted agents each against a different genetic mutations (sub-trials) within a single histology (“the umbrella”). (Verweij2019)		
			An umbrella trial is designed to enroll patients with a specific histology, and any of multiple potential tumor molecular alterations, who are assigned to different subtrials based on those alterations. (Yee2019)		
			Umbrella trials assign patients to one of potentially many treatment arms, based on a specific cancer type and genetic markers. (Soldatos2019)		

For peer review only

			Patients are screened for a panel of biochemical, genetic, and/or immunologic markers associated with their disease and, on the basis of the markers detected, assigned to a biomarker-driven treatment strategy or targeted therapy that is most likely to result in favorable outcomes. (Ou2019)		
	**Randomised umbrella design**				
	**Non randomised umbrella design**				
		**Bayesian adaptive umbrella design**			
**Umbrella-basket hybrid**					

References

1. Ahmad T, O'Connor CM. Therapeutic Implications of Biomarkers in Chronic Heart Failure. Clin Pharmacol Ther. 2013 Oct;94(4):468–79.
2. Alexander BM, Lorenzo T. Bayesian baskets: A novel approach to biomarker-based clinical trial design. J Clin Oncol. 2016;34: e14057
3. Alexander BM, Trippa L, Gaffey S, Arrillaga-Romany IC, Lee EQ, Rinne ML, et al. Individualized Screening Trial of Innovative Glioblastoma Therapy (INSIGhT): A Bayesian Adaptive Platform Trial to Develop Precision Medicines for Patients With Glioblastoma. JCO Precis Oncol. 2019 Dec;(3):1–13.
4. Antoniou M, Jorgensen AL, Kolamunnage-Dona R. Biomarker-Guided Adaptive Trial Designs in Phase II and Phase III: A Methodological Review. Soyer HP, editor. PLOS ONE. 2016 Feb 24;11(2):e0149803.
5. Antoniou M, Kolamunnage-Dona R, Jorgensen A. Biomarker-Guided Non-Adaptive Trial Designs in Phase II and Phase III: A Methodological Review. J Pers Med. 2017 Jan 25;7(1):1.
6. Barry WT, Perou CM, Marcom PK, Carey LA, Ibrahim JG. The Use of Bayesian Hierarchical Models for Adaptive Randomization in Biomarker-Driven Phase II Studies. J Biopharm Stat. 2015 Jan 2;25(1):66–88.
7. Beckman R, Antonijevic Z, Kalamegham R, Chen C. Adaptive Design for a Confirmatory Basket Trial in Multiple Tumor Types Based on a Putative Predictive Biomarker. Clin Pharmacol Ther. 2016 Dec;100(6):617–25.
8. Berry DA. The Brave New World of clinical cancer research: Adaptive biomarker-driven trials integrating clinical practice with clinical research. Mol Oncol. 2015 May;9(5):951–9.
9. Berry SM, Broglio KR, Groshen S, Berry DA. Bayesian hierarchical modeling of patient subpopulations: Efficient designs of Phase II oncology clinical trials. Clin Trials J Soc Clin Trials. 2013 Oct;10(5):720–34.
10. Blagden SP, Billingham L, Brown LC, Buckland SW, Cooper AM, Ellis S, et al. Effective delivery of Complex Innovative Design (CID) cancer trials—A consensus statement. Br J Cancer. 2020 Feb 18;122(4):473–82.
11. Bothwell LE, Avorn J, Khan NF, Kesselheim AS. Adaptive design clinical trials: a review of the literature and ClinicalTrials.gov. BMJ Open. 2018 Feb;8(2):e018320.
12. Cabarrou B, Sfumato P, Leconte E, Boher JM, Filleron T. Designing phase II clinical trials to target subgroup of interest in a heterogeneous population: A case study using an R package. Comput Biol Med. 2018 Sep;100:239–46.
13. Cecchini M, Rubin EH, Blumenthal GM, Ayalew K, Burris HA, Russell-Einhorn M, et al. Challenges with Novel Clinical Trial Designs: Master Protocols. Clin Cancer Res. 2019 Apr 1;25(7):2049–57.
14. Cerqueira FP, Jesus AMC, Cotrim MD. Adaptive Design: A Review of the Technical, Statistical, and Regulatory Aspects of Implementation in a Clinical Trial. Ther Innov Regul Sci. 2019 Feb 27;216847901983124.
15. Chen C, Li X (Nicole), Yuan S, Antonijevic Z, Kalamegham R, Beckman RA. Statistical Design and Considerations of a Phase 3 Basket Trial for Simultaneous Investigation of Multiple Tumor Types in One Study. Stat Biopharm Res. 2016 Jul 2;8(3):248–57.
16. Diao G, Dong J, Zeng D, Ke C, Rong A, Ibrahim JG. Biomarker threshold adaptive designs for survival endpoints. J Biopharm Stat. 2018 Nov 2;28(6):1038–54.

For peer review only

17. Dienstmann R, Rodon J, Tabernero J. Optimal design of trials to demonstrate the utility of genomically-guided therapy: Putting Precision Cancer Medicine to the test. Mol Oncol. 2015 May;9(5):940–50.
18. Doorenbos AZ, Haozous EA, Jang MK, Langford D. Sequential multiple assignment randomization trial designs for nursing research. Res Nurs Health. 2019 Dec;42(6):429–35.
19. Eng KH. Randomized reverse marker strategy design for prospective biomarker validation. Stat Med. 2014 Aug 15;33(18):3089–99.
20. Fadoukhair Z, Zardavas D, Chad MA, Goulioti T, Aftimos P, Piccart M. Evaluation of targeted therapies in advanced breast cancer: the need for large-scale molecular screening and transformative clinical trial designs. Oncogene. 2016 Apr;35(14):1743–9.
21. Ferrarotto R, Redman MW, Gandara DR, Herbst RS, Papadimitrakopoulou V. Lung-MAP-framework, overview, and design principles. Chin Clin Oncol. 2015;4(3):1–6.
22. Freidlin B, Korn EL. Biomarker-adaptive clinical trial designs. Pharmacogenomics. 2010 Dec;11(12):1679–82.
23. Freidlin B, Korn EL, Gray R. Marker Sequential Test (MaST) design. Clin Trials J Soc Clin Trials. 2014 Feb;11(1):19–27.
24. Galanis E, Wu W, Sarkaria J, Chang SM, Colman H, Sargent D, et al. Incorporation of Biomarker Assessment in Novel Clinical Trial Designs: Personalizing Brain Tumor Treatments. Curr Oncol Rep. 2011 Feb;13(1):42–9.
25. Gao Z, Roy A, Tan M. Multistage adaptive biomarker-directed targeted design for randomized clinical trials. Contemp Clin Trials. 2015 May;42:119–31.
26. Garralda E, Dienstmann R, Piris-Giménez A, Braña I, Rodon J, Tabernero J. New clinical trial designs in the era of precision medicine. Mol Oncol. 2019 Mar;13(3):549–57.
27. Gilson C, Chowdhury S, Parmar MKB, Sydes MR. Incorporating Biomarker Stratification into STAMPEDE: an Adaptive Multi-arm, Multi-stage Trial Platform. Clin Oncol. 2017 Dec;29(12):778–86.
28. Gómez-López G, Dopazo J, Cigudosa JC, Valencia A, Al-Shahrour F. Precision medicine needs pioneering clinical bioinformaticians. 2017. :1–15.
29. Heerspink HJL, List J, Perkovic V. New clinical trial designs for establishing drug efficacy and safety in a precision medicine era. Diabetes Obes Metab. 2018 Oct;20:14–8.
30. Heerspink HJL, Perkovic V. Trial Design Innovations to Accelerate Therapeutic Advances in Chronic Kidney Disease: Moving from Single Trials to an Ongoing Platform. Clin J Am Soc Nephrol. 2018 Jun 7;13(6):946–8.
31. Hobbs BP, Kane MJ, Hong DS, Landin R. Statistical challenges posed by uncontrolled master protocols: sensitivity analysis of the vemurafenib study. Ann Oncol. 2018 Dec;29(12):2296–301.
32. Hobbs BP, Landin R. Bayesian basket trial design with exchangeability monitoring: Bayesian Basket Trial Design with Exchangeability Monitoring. Stat Med. 2018 Nov 10;37(25):3557–72.
33. Hong F, Simon R. Run-In Phase III Trial Design With Pharmacodynamics Predictive Biomarkers. J Natl Cancer Inst. 2013;105(21):6.
34. Janiaud P, Serghiou S, Ioannidis JPA. New clinical trial designs in the era of precision medicine: An overview of definitions, strengths, weaknesses, and current use in oncology. Cancer Treat Rev. 2019 Feb;73:20–30.
35. Johnson DR, Galanis E. Incorporation of Prognostic and Predictive Factors Into Glioma Clinical Trials. Curr Oncol Rep. 2013 Feb;15(1):56–63.
36. Jones CL, Holmgren E. An adaptive Simon Two-Stage Design for Phase 2 studies of targeted therapies. Contemp Clin Trials. 2007 Sep;28(5):654–61.
37. Joshi YB, Light GA. Using EEG-Guided Basket and Umbrella Trials in Psychiatry: A Precision Medicine Approach for Cognitive Impairment in Schizophrenia. Front Psychiatry. 2018 Nov 19;9:554.
38. Kaplan R. The FOCUS4 design for biomarker stratified trials. Chin Clin Oncol. 2015;4(3):1–10.
39. Kesselmeier M, Scherag A. Adaptive clinical trials in sepsis research: pros and cons. Infection. 2019; 47 (Suppl 1): S1-S67.
40. Kidwell KM, Wahed AS. Weighted log-rank statistic to compare shared-path adaptive treatment strategies. Biostatistics. 2013 Apr 1;14(2):299–312.
41. Kimani PK, Todd S, Renfro LA, Stallard N. Point estimation following two-stage adaptive threshold enrichment clinical trials: Estimators for adaptive threshold enrichment clinical trials. Stat Med. 2018 Sep 30;37(22):3179–96.
42. Lam M, Loree JM, Lima AAP, Chun YS, Kopetz S. Accelerating Therapeutic Development through Innovative Trial Design in Colorectal Cancer. Curr Treat Options Oncol. 2018 Feb;19(2):11.
43. Lam VK, Papadimitrakopoulou V. Master protocols in lung cancer: experience from Lung Master Protocol. Curr Opin Oncol. 2018 Mar;30(2):92–7.
44. Le-Rademacher J, Dahlberg S, Lee JJ, Adjei AA, Mandrekar SJ. Biomarker Clinical Trials in Lung Cancer: Design, Logistics, Challenges, and Practical Considerations. J Thorac Oncol. 2018 Nov;13(11):1625–37.
45. Leonetti A, Boyd L, Giuliani J, Giovannetti E, Garajová I. Light and shadow on innovative clinical trial designs: reflections from the EORTC-PAMM course on 'preclinical and early-phase clinical pharmacology.' Expert Rev Clin Pharmacol. 2019 Nov 2;12(11):1033–6.
46. Lin J-A, He P. Reinventing clinical trials: a review of innovative biomarker trial designs in cancer therapies. Br Med Bull. 2015 Jun;114(1):17–27.
47. Liu S, Lee JJ. An overview of the design and conduct of the BATTLE trials. Chin Clin Oncol. 2015;4(3):13.
48. Mandrekar SJ, Dahlberg SE, Simon R. Improving Clinical Trial Efficiency: Thinking outside the Box. Am Soc Clin Oncol Educ Book. 2015 May;(35):e141–7.
49. Matsui S, Crowley J. Biomarker-Stratified Phase III Clinical Trials: Enhancement with a Subgroup-Focused Sequential Design. Clin Cancer Res. 2018 Mar 1;24(5):994–1001.
50. Mazzarella L, Morganti S, Marra A, Trapani D, Tini G, Pelicci P, et al. Master protocols in immuno-oncology: do novel drugs deserve novel designs? J Immunother Cancer. 2020 Mar;8(1):e000475.
51. Moore KN, Mannel RS. Is the NCI MATCH trial a match for gynecologic oncology? Gynecol Oncol. 2016 Jan;140(1):161–6.
52. O'Brien C, Carter L, Cook N, Dean E. Novel Early Phase Clinical Trial Design in Oncology. Pharm Med. 2017 Oct;31(5):297–307.
53. Ohwada S, Morita S. Bayesian adaptive patient enrollment restriction to identify a sensitive subpopulation using a continuous biomarker in a randomized phase 2 trial. Pharm Stat. 2016 Sep;15(5):420–9.
54. Ondra T, Dmitrienko A, Friede T, Graf A, Miller F, Stallard N, et al. Methods for identification and confirmation of targeted subgroups in clinical trials: A systematic review. J Biopharm Stat. 2016 Jan 2;26(1):99–119.

For peer review only

55. Ou F-S, An M-W, Ruppert AS, Mandrekar SJ. Discussion of Trial Designs for Biomarker Identification and Validation Through the Use of Case Studies. JCO Precis Oncol. 2019 Dec;(3):1–10.
56. Park JJH, Siden E, Zoratti MJ, Dron L, Harari O, Singer J, et al. Systematic review of basket trials, umbrella trials, and platform trials: a landscape analysis of master protocols. Trials. 2019 Dec;20(1):572.
57. Park JJH, Hsu G, Siden EG, Thorlund K, Mills EJ. An overview of precision oncology basket and umbrella trials for clinicians. CA Cancer J Clin. 2020 Mar;70(2):125–37.
58. Perco P, Pena M, Heerspink HJL, Mayer G. Multimarker Panels in Diabetic Kidney Disease: The Way to Improved Clinical Trial Design and Clinical Practice? Kidney Int Rep. 2019 Feb;4(2):212–21.
59. Renfro LA, Mallick H, An M-W, Sargent DJ, Mandrekar SJ. Clinical trial designs incorporating predictive biomarkers. Cancer Treat Rev. 2016 Feb;43:74–82.
60. Renfro LA, An M-W, Mandrekar SJ. Precision oncology: A new era of cancer clinical trials. Cancer Lett. 2017 Feb;387:121–6.
61. Renfro LA, Sargent DJ. Statistical controversies in clinical research: basket trials, umbrella trials, and other master protocols: a review and examples. Ann Oncol. 2017 Jan;28(1):34–43.
62. Renfro LA, Mandrekar SJ. Definitions and statistical properties of master protocols for personalized medicine in oncology. J Biopharm Stat. 2018 Mar 4;28(2):217–28.
63. Riddell CA, Zhao Y, Petkau J. An adaptive clinical trials procedure for a sensitive subgroup examined in the multiple sclerosis context. Stat Methods Med Res. 2016 Aug;25(4):1330–45.
64. Rosenblum M, Hanley DF. Adaptive Enrichment Designs for Stroke Clinical Trials. Stroke. 2017 Jul;48(7):2021–5.
65. Said R, Tsimberidou A-M. Basket Trials and the MD Anderson Precision Medicine Clinical Trials Platform: Cancer J. 2019;25(4):282–6.
66. Saville BR, Berry SM. Efficiencies of platform clinical trials: A vision of the future. Clin Trials J Soc Clin Trials. 2016 Jun;13(3):358–66.
67. Semler MW, Bernard GR, Aaron SD, Angus DC, Biros MH, Brower RG, et al. Identifying Clinical Research Priorities in Adult Pulmonary and Critical Care. NHLBI Working Group Report. Am J Respir Crit Care Med. 2020 Aug 15;202(4):511–23.
68. Shah SJ. Innovative Clinical Trial Designs for Precision Medicine in Heart Failure with Preserved Ejection Fraction. J Cardiovasc Transl Res. 2017 Jun;10(3):322–36.
69. Simon R. Clinical trial designs for evaluating the medical utility of prognostic and predictive biomarkers in oncology. Pers Med. 2010 Jan;7(1):33–47.
70. Simon R. Clinical trials for predictive medicine: new challenges and paradigms. Clin Trials J Soc Clin Trials. 2010 Oct;7(5):516–24.
71. Simon R. Genomic Alteration–Driven Clinical Trial Designs in Oncology. Ann Intern Med. 2016 Aug 16;165(4):270.
72. Simon R. Critical Review of Umbrella, Basket, and Platform Designs for Oncology Clinical Trials: Review of umbrella, basket, and platform trial designs. Clin Pharmacol Ther. 2017 Dec;102(6):934–41.
73. Simon R. New designs for basket clinical trials in oncology. J Biopharm Stat. 2018 Mar 4;28(2):245–55.
74. Simon KC, Tideman S, Hillman L, Lai R, Jathar R, Ji Y, et al. Design and implementation of pragmatic clinical trials using the electronic medical record and an adaptive design. JAMIA Open. 2018 Jul 1;1(1):99–106.
75. Soldatos, Kaduthanam, Jackson. Precision Oncology—The Quest for Evidence. J Pers Med. 2019 Sep 5;9(3):43.
76. Spencer AV, Harbron C, Mander A, Wason J, Peers I. An adaptive design for updating the threshold value of a continuous biomarker: An Adaptive Design for Updating the Threshold of a Biomarker. Stat Med. 2016 Nov 30;35(27):4909–23.
77. Tajik P, Zwinderman AH, Mol BW, Bossuyt PM. Trial Designs for Personalizing Cancer Care: A Systematic Review and Classification. Clin Cancer Res. 2013 Sep 1;19(17):4578–88.
78. Talisa VB, Yende S, Seymour CW, Angus DC. Arguing for Adaptive Clinical Trials in Sepsis. Front Immunol. 2018 Jun 28;9:1502.
79. Tao JJ, Schram AM, Hyman DM. Basket Studies: Redefining Clinical Trials in the Era of Genome-Driven Oncology. Annu Rev Med. 2018 Jan 29;69(1):319–31.
80. Trippa L, Alexander BM. Bayesian Baskets: A Novel Design for Biomarker-Based Clinical Trials. J Clin Oncol. 2017 Feb;35(6):JCO.2016.68.286.
81. Tsimberidou AM, Fountzilas E, Nikanjam M, Kurzrock R. Review of precision cancer medicine: Evolution of the treatment paradigm. Cancer Treat Rev. 2020 Jun;86:102019.
82. Uozumi R, Hamada C. Interim decision-making strategies in adaptive designs for population selection using time-to-event endpoints. J Biopharm Stat. 2017 Jan 2;27(1):84–100.
83. Van Norman GA. Phase II Trials in Drug Development and Adaptive Trial Design. JACC Basic Transl Sci. 2019 Jun;4(3):428–37.
84. Verweij J, Hendriks HR, Zwierzina H, Hanauske, Wacheck V, Collignon O, et al. Innovation in oncology clinical trial design. Cancer Treat Rev. 2019 Mar;74:15–20.
85. Wang S-J, Hung HMJ, O'Neill R. Adaptive design clinical trials and trial logistics models in CNS drug development. Eur Neuropsychopharmacol. 2011 Feb;21(2):159–66.
86. Wang T, Wang X, Zhou H, Cai J, George SL. Auxiliary variable–enriched biomarker-stratified design. Stat Med. 2018 Dec 30;37(30):4610–35.
87. Xu Y, Trippa L, Müller P, Ji Y. Subgroup-Based Adaptive (SUBA) Designs for Multi-arm Biomarker Trials. Stat Biosci. 2016 Jun;8(1):159–80.
88. Yee LM, McShane LM, Freidlin B, Mooney MM, Korn EL. Biostatistical and Logistical Considerations in the Development of Basket and Umbrella Clinical Trials: Cancer J. 2019;25(4):254–63.
89. Yuan Y. Invited session 11 - Recent developments in umbrella, basket and platform trial designs. Clinical Trials. 2018; 15(S2);35-192
90. Zardavas D, Piccart-Gebhart M. Clinical Trials of Precision Medicine through Molecular Profiling: Focus on Breast Cancer. Am Soc Clin Oncol Educ Book. 2015 May;(35):e183–90.
91. Zhang W, Wang J, Menon S. Advancing cancer drug development through precision medicine and innovative designs. J Biopharm Stat. 2018 Mar 4;28(2):229–44.
92. Zhang Z, Chen R, Soon G, Zhang H. Treatment evaluation for a data-driven subgroup in adaptive enrichment designs of clinical trials: Treatment evaluation for a data-driven subgroup in adaptive enrichment designs of clinical trials. Stat Med. 2018 Jan 15;37(1):1–11.

For peer review only

Supplementary file V. Examples of clinical trials

**Type of trial designs**	**Sub-type of trial designs**	**Variations**	**Example(s)**	**Trial registration num.**	**Recruitment status as of 12 March 2021**	**Clinical Field**	**Phase**	**Reference**
Marker stratified design			CALGB-30506	NCT00863512	Completed	Lung cancer	III	(1)
			EORTC10994 P53	NCT00017095	Completed	Breast cancer	III	(2)
			IBCSG trial IX	nf[1]	nf[1]	Breast cancer	nf[1]	(1)
			MARVEL	NCT00738881	Completed	Lung cancer	III	(1,3–6)
			MINDACT	NCT00433589	Ongoing	Breast cancer	III	(1)
			RTOG0825	NCT00884741	Completed	Glioblastoma	III	(1,7)
	Subgroup specific design	Sequential-subgroup specific design	PRIME	NCT00364013	Completed	Colorectal cancer	III	(1)
	Biomarker-positive and overall strategies	Biomarker-positive and overall strategies with parallel assessment	ARCHER	NCT01360554	Completed	Lung cancer	III	(1)
			MERiDIAN	NCT01663727	Completed	Breast cancer	III	(1)
			MONET1	NCT00460317	Completed	Lung cancer	III	(1)
			S0819	NCT00946712	Completed	Lung cancer	III	(1)
			SATURN	NCT00556712	Completed	Lung cancer	III	(1)
			ZODIAC	NCT00312377	Completed	Lung cancer	III	(1)
		Biomarker-positive and overall strategies with sequential assessment	N0147	NCT00079274	Completed	Colorectal cancer	III	(1)

For peer review only

		Marker sequential test design	ECOG E1910	NCT02003222	Ongoing	Leukemia	III	(1)
Hybrid design			TAILORx	NCT00310180	Completed	Breast cancer	III	(1,8)
Biomarker strategy design with biomarker assessment in the control arm			ERCC1	NCT00801736	Completed	Lung cancer	III	(9)
			GILT docetaxel	NCT00174629	Completed	Lung cancer	III	(1)
			LIFT	NCT02498977	Completed	Transplantation, Liver	IV	(10)
Biomarker strategy design without biomarker assessment in the control arm			GUIDE-IT	NCT01685840	Completed	Chronic Heart Failure	n/a[2]	(11)
			iPEGASUS	NCT03021525	Ongoing	Hemodynamic Instability; Cardiac Output, High; Peroperative Complication	n/a[2]	(12)
			OCTOPUS	ISRCTN81464462	Completed	Mild head injury	n/a[2]	(1)
			PUFFIN	NCT03654508	Ongoing	Asthma	n/a[2]	(13)
Modified biomarker strategy design			MINDACT	NCT00433589	Ongoing	Breast cancer	III	(8,14)
			NCI-MPACT	NCT01827384	Completed	Advanced malignant solid neoplasm	II	(5)
			SHIVA	NCT01771458	Unknown[3]	Reccurent/Metastatic Solid; Tumor Disease	II	(5,6,15,16)

Sequential Multiple Assignment Randomised Trial (SMART) design			Siyaphambili Study	NCT03500172	Completed	HIV	n/a[2]	(17)
Adaptive biomarker design			I-SPY 2	NCT01042379	Ongoing	Breast cancer	II	(18)
Adaptive strategy for biomarker with measurement error			OPTIMA	ISRCTN42400492	Ongoing	Breast cancer	n/a[2]	(6)
Outcome-based adaptive randomization design			BATTLE	NCT00409968	Completed	Lung cancer	II	(5,6,19–21)
			I-SPY 2	NCT01042379	Ongoing	Breast cancer	II	(1,5,7,22–25)
			ProBio	NCT03903835	Ongoing	Prostate cancer	III	(26–28)
			SEPSIS-ACT	NCT02508649	Completed	Septic shock	II/III	(29)
Adaptive patient enrichment design			MISTIE	NCT01827046	Completed	Intracerebral Hemorrhage	III	(30)
			MK-0462-082 AM7	NCT01001234	Completed	Migraine	III	(31)
			THRIVE	NCT00543725	Completed	HIV	III	(32)
Adaptive parallel Simon two-stage design			-	NCT00958971	Completed	Breast cancer	II	(31)
Multi-arm multi-stage design			ATLANTIS	ISRCTN25859465	Ongoing	Bladder	II	(33)
			BIOMEDE	NCT02233049	Unknown[3]	Diffuse Intrinsic Pontine Glioma	II	(34,35)

For peer review only

			PanACEA MAMS	NCT01785186	Ongoing	Tuberculosis	II	(36)
			PLATFORM	NCT02678182	Ongoing	Gastric	II	(37)
			STAMPEDE	NCT00268476	Ongoing	Prostate cancer	II/III	(25,31,38)
		Two-stage adaptive seamless design	SEPSIS-ACT	NCT02508649	Completed	Septic shock	II/III	(29)
		Group sequential design	SHARP	NCT00105443	Completed	Liver cancer	III	(18)
Tandem two stage design			-	NCT00735917	Completed	Pancreas cancer	II	(31)
Platform design			BATTLE	NCT00409968	Completed	Lung cancer	II	(39)
			DIAN-TU	NCT01760005	Ongoing	Alzheimer's Disease	II/III	(40,41)
			EPAD	NCT02804789	Completed	Alzheimer's Disease	n/a[2]	(41)
			FOCUS4	ISRCTN90061546	Ongoing	Colorectal cancer	II/III	(42)
			FRACTION-GC	NCT2935634	Ongoing	Gastric Cancer	II	(43,44)
			FRACTION-Lung	NCT02750514	Ongoing	Lung cancer	II	(43,45)
			FRACTION-RCC	NCT2996110	Ongoing	Renal Cell Carcinoma	II	(43)
			GBM AGILE	NCT03970447	Ongoing	Glioblastoma	II/III	(46)
			I-SPY 2	NCT01042379	Ongoing	Breast cancer	II	(29)
			-	NCT03739710	Ongoing	Neoplasms	II	(48)

For peer review only

			ORCHARD	NCT03944772	Ongoing	Lung cancer	II	(49)
			PANGEA-IMBBP	NCT02213289	Ongoing	Adenocarcinoma	II	(50)
			PLATforM	NCT03484923	Ongoing	Melanoma	II	(51)
			SHIVA	NCT01771458	Unknown[3]	Reccurent/Metastatic Solid; Tumor Disease	II	(52)
			STAMPEDE	NCT00268476	Ongoing	Prostate cancer	II/III	(53,54)
		Bayesian adaptive platform trial	INSIGhT	NCT02977780	Ongoing	Glioblastoma	II	(47)
	Randomized embedded multifactorial adaptive platform (REMAP)		REMAP-CAP	NCT02735707	Ongoing	Community-acquired Pneumonia, Influenza, COVID-19	IV	(29)
			UPMC REMAP	NCT03861767	Ongoing	Aging	III	(55)
Basket design			ALCHEMIST	NCT02194738	Ongoing	Lung cancer	III	(53)
			BASKET 1	NCT00928525	Unknown[3]	Advanced Desmoid Tumor, Advanced Chondrosarcoma	II	(2)
			CAPTUR	NCT03297606	Ongoing	Lymphoma, Non-Hodgkin Multiple Myeloma Advanced Solid Tumors	II	(56)
			CLUSTER	NCT02059291	Completed	Fever	III	(41)
			CREATE	NCT01524926	Ongoing	Locally Advanced and/or Metastatic Anaplastic Large Cell Lymphoma; Locally Advanced and/or Metastatic	II	(57)

For peer review only

						Inflammatory Myofibroblastic Tumor; Locally Advanced and/or Metastatic Papillary Renal Cell Carcinoma Type; Locally Advanced and/or Metastatic Alveolar Soft Part Sarcoma; Locally Advanced and/or Metastatic Clear Cell		
			CUSTOM	NCT01306045	Ongoing	Lung cancer	II	(58)
			DART SWOG 1609	NCT02834013	Ongoing	Rare tumors	II	(59)
			DRUP	NCT02925234	Ongoing	Solid tumor, multiple myeloma or B cell non-Hodgkin lymphoma	II	(60)
			IMPACT 2	NCT02152254	Ongoing	Metastatic Malignant Neoplasm Recurrent Malignant Neoplasm	n/a[2]	(22)
			IGNYTE-ESO	NCT03967223	Ongoing	Neoplasms	II	(61)
			K-BASKET	NCT03491345 NCT03017521	Unknown[3]	Solid tumor	II	(2)
			Keynote 158	NCT02628067	Ongoing	Anal Cancer;Colorectal Cancer;Lung Cancer;Pancreas cancer;Endometrial, small intestine, cervical, vulvar, salivary gland carcinoma , mesothelioma and other advanced solid tumor	II	(62,63)

For peer review only

For peer review only - http://bmjopen.bmj.com/site/about/guidelines.xhtml

			MEDIOLA	NCT02734004	Ongoing	Ovarian Breast SCLC Gastric Cancers	II	(64–66)
			METADUR	NCT02811497	Ongoing	Colorectal carcinoma, ovarian and breast cancer	II	(2)
			MiMe-A	NCT03339843	Ongoing	Esophageal Adenocarcinoma, Esophagus SCC, Cholangiocarcinoma, Urothelial/Bladder Cancer, Nos Endometrial Cancer	II	(2)
			MOBILITY-001	NCT02399943	Ongoing	Colorectal cancer	II	(2)
			MOBILITY-002	NCT02428270	Ongoing	Pancreatic cancer, Adenocarcinoma	II	(2)
			MOBILITY-003	NCT02506517	Ongoing	Solid tumors	II	(2)
			MyPathway	NCT02091141	Ongoing	Neoplasms Solid Tumors; Biliary Cancer; Salivary Cancer; Bladder Cancer	II	(67)
			MoST	ACTRN12616000 908437	Ongoing	Solid tumor	II	(68,69)
			_	NCT03836352	Ongoing	Ovarian Cancer Hepatocellular Carcinoma Non-small Cell Lung Cancer Bladder Cancer Microsatellite Instability-High	II	(70)

For peer review only

			n/a	NCT02675829	Ongoing	Solid tumors	II	(71)
			NAVIGATE	NCT02576431	Ongoing	Solid Tumors Harboring NTRK Fusion	II	
			NCI CTRP	NCT02478320	Ongoing	Advanced cancers	II	(2)
			NCI-MATCH	NCT02465060	Ongoing	Advanced malignant solid neoplasm	II	(5,6,19,39,72–81)
			NCI-MPACT	NCT01827384	Ongoing	Advanced malignant solid neoplasm	II	(58,73,82,83)
			P10s Basket trial	NCT03003195	Ongoing	Neoplasms by Site Metastatic Cancer	II	(2)
			Paragon	ACTRN12610000796088 (prospectively registered)	Ongoing	Ovarian cancer	II	(2)
			SHIVA	NCT01771458	Unknown[3]	Reccurent/Metastatic Solid; Tumor Disease	II	(15,16,84)

For peer review only

			SIGNATURE	NCT01831726<br>NCT01885195<br>NCT01981187<br>NCT02002689<br>NCT02160041<br>NCT02186821<br>NCT02187783<br>NCT01833169	Completed	Solid tumor, hematologic malignancies	II	(2)
			STARTRK-2	NCT02568267	Ongoing	Solid tumor	II	(2)
			SUMMIT	NCT01953926	Ongoing	Solid Tumors Harboring Somatic HER2 or EGFR Exon 18 Mutations	II	(2)
			TAPUR	NCT02693535	Ongoing	Lymphoma, Non-Hodgkin Multiple Myeloma Advanced Solid Tumors	II	(22)
			TMB-H basket	UMIN000033182	Ongoing	Colorectal cancer, Gastric cancer, Esophageal cancer, Biliary tract cancer, Pancreatic cancer, and Other gastrointestinal cancer	II	(85)
			VE-BASKET	NCT01524978	Completed	Multiple Myeloma, Neoplasms	II	(2,86)
Basket of basket design			-	NCT03767075	Ongoing	Advanced Solid Tumor	II	(88–90)
Umbrella design			ADAPT	NCT01779206	Ongoing	Breast Cancer	II/III	(91–93)
			ALCHEMIST	NCT02194738<br>NCT02193282<br>NCT02201992<br>NCT02595944	Ongoing	Lung cancer	III	(2,5,19,39,42,74,78,94,95)

For peer review only

			BATTLE-1	NCT00411632<br>NCT00411671<br>NCT00410189<br>NCT00410059	Completed	Lung cancer	II	(2,96)
			BATTLE-2	NCT01248247	Ongoing	Lung cancer	II	(2)
			BFAST	NCT03178552	Ongoing	Lung cancer	II/III	(88)
			FOCUS4	ISRCTN90061546	Ongoing	Colorectal cancer	II/III	(2,33)
			HUDSON	NCT03334617	Ongoing	Lung cancer	II	(2)
			I-SPY 2	NCT01042379	Ongoing	Breast cancer	II	(2)
			Lung-MAP	NCT02154490<br>NCT02766335<br>NCT02785913<br>NCT02785939<br>NCT02965378<br>NCT02926638<br>NCT03373760<br>NCT03377556<br>NCT02785952	Ongoing	Lung cancer	II/III	(2,5,6,19,74,76–80,82,94,97–101)
			MiST	NCT03654833	Ongoing	Mesothelioma, Malignant	II	(102)
			MODUL	NCT02291289	Ongoing	Colorectal cancer	II	(103)
			MOSCATO	NCT01566019	Ongoing	Metastatic Solid Tumors (Any Localization)	n/a[2]	(90)
			-	NCT02276027	Completed	Lung cancer	II	(104)
			Pediatric MATCH	NCT03155620	Ongoing	Advanced Malignant Solid Neoplasm	II	(2)
			plasmaMATCH	NCT03182634	Ongoing	Breast cancer	II	(105)
			PLATO	ISRCTN88455282	Ongoing	Anal cancer	II/III	(106,107)
			Precision-Panc: PRIMUS	NCT04161417	Ongoing	Pancreas cancer	n/a[2]	(108)

For peer review only

			PRIMUS 002	ISRCTN34129115	Ongoing	Pancreas cancer	II	(109)
			SAFIR02_Lung	NCT02117167	Completed	Lung cancer	II	(57)
			SAFIR02_Breast	NCT02299999	Completed	Breast cancer	II	(57)
			SUKSES-S	NCT02688894	Ongoing	Small Cell Lung Cancers	II	(110,111)
			TRIUMPH	NCT03292250 NCT03356587	Unknown[3]	Head and neck squamous cell carcinoma	II	(2)
			TRUMP	NCT03574402	Ongoing	Lung cancer	II	(2)
			UPSTREAM	NCT03088059	Ongoing	Head and Neck Squamous Cell Carcinoma	II	(112)
			VIKTORY	NCT02299648	Ongoing	Molecular profiling	n/a[2]	(113)
			WINTHER	NCT01856296	Completed	Metastatic cancer	n/a[2]	(114)
			WSG ADAPT	NCT01781338	Ongoing	Breast cancer	II/III	(2)
		Bayesian adaptive umbrella design	National Lung Matrix Trial	NCT02664935	Ongoing	Lung cancer	II	(2,33,100)
		Randomized umbrella design	AMBITION	NCT03699449	Ongoing	Ovarian cancer	II	(115)
Umbrella-basket hybrid			MASTER KEY	UMIN000027552	Ongoing	Cancer	II	(116)
Umbrella-basket hybrid			NCI-MATCH	NCT02465060	Ongoing	Advanced malignant solid neoplasm	II	(83)

[1] Not found

[2] Not applicable is used on the Clinicaltrilas.gov website to describe trials without FDA-defined phases including trials of devices or behavioural interventions.

[3] Unknown is used to indicate a trial status that has not been verified within the past two years on the Clinical trials.gov website

References

1. Antoniou M, Kolamunnage-Dona R, Jorgensen A. Biomarker-Guided Non-Adaptive Trial Designs in Phase II and Phase III: A Methodological Review. J Pers Med. 2017 Jan 25;7(1):1.
2. Janiaud P, Serghiou S, Ioannidis JPA. New clinical trial designs in the era of precision medicine: An overview of definitions, strengths, weaknesses, and current use in

For peer review only

oncology. Cancer Treat Rev. 2019 Feb;73:20–30.
3. Buch MH, Pavitt S, Parmar M, Emery P. Creative trial design in RA: optimizing patient outcomes. Nat Rev Rheumatol. 2013 Mar;9(3):183–94.
4. Ondra T, Dmitrienko A, Friede T, Graf A, Miller F, Stallard N, et al. Methods for identification and confirmation of targeted subgroups in clinical trials: A systematic review. J Biopharm Stat. 2016 Jan 2;26(1):99–119.
5. Renfro LA, An M-W, Mandrekar SJ. Precision oncology: A new era of cancer clinical trials. Cancer Lett. 2017 Feb;387:121–6.
6. Renfro LA, Mallick H, An M-W, Sargent DJ, Mandrekar SJ. Clinical trial designs incorporating predictive biomarkers. Cancer Treat Rev. 2016 Feb;43:74–82.
7. Galanis E, Wu W, Sarkaria J, Chang SM, Colman H, Sargent D, et al. Incorporation of Biomarker Assessment in Novel Clinical Trial Designs: Personalizing Brain Tumor Treatments. Curr Oncol Rep. 2011 Feb;13(1):42–9.
8. Tajik P, Zwinderman AH, Mol BW, Bossuyt PM. Trial Designs for Personalizing Cancer Care: A Systematic Review and Classification. Clin Cancer Res. 2013 Sep 1;19(17):4578–88.
9. Freidlin B, McShane LM, Korn EL. Randomized Clinical Trials With Biomarkers: Design Issues. JNCI J Natl Cancer Inst. 2010 Feb 3;102(3):152–60.
10. Clinicaltrials.gov. Liver Immunosuppression Free Trial (LIFT) [Internet]. Available from: https://clinicaltrials.gov/ct2/show/NCT02498977
11. Ahmad T, O'Connor CM. Therapeutic Implications of Biomarkers in Chronic Heart Failure. Clin Pharmacol Ther. 2013 Oct;94(4):468–79.
12. Funcke S, Saugel B, Koch C, Schulte D, Zajonz T, Sander M, et al. Individualized, perioperative, hemodynamic goal-directed therapy in major abdominal surgery (iPEGASUS trial): study protocol for a randomized controlled trial. Trials. 2018 Dec;19(1):273.
13. Vijverberg SJ, Pijnenburg MW, Hövels AM, Koppelman GH, Maitland-van der Zee A-H. The need for precision medicine clinical trials in childhood asthma: rationale and design of the PUFFIN trial. Pharmacogenomics. 2017 Mar;18(4):393–401.
14. Simon R. Clinical trial designs for evaluating the medical utility of prognostic and predictive biomarkers in oncology. Pers Med. 2010 Jan;7(1):33–47.
15. Dienstmann R, Rodon J, Tabernero J. Optimal design of trials to demonstrate the utility of genomically-guided therapy: Putting Precision Cancer Medicine to the test. Mol Oncol. 2015 May;9(5):940–50.
16. Paoletti X, Asselain B, Kamal M, Servant N, Huppé P, Bieche I, et al. Design and statistical principles of the SHIVA trial. Chin Clin Oncol. 2015;4(3):1–10.
17. Clinicaltrials.gov. HIV Treatment Retention Interventions for Women Living With HIV (Siyaphambili Study) [Internet]. Available from: https://clinicaltrials.gov/ct2/show/NCT03500172
18. Cheng A-L. Combining Adaptive Design and Omics for Future HCC Trials. Liver Cancer 2015. 4: 1-257.
19. Le-Rademacher J, Dahlberg S, Lee JJ, Adjei AA, Mandrekar SJ. Biomarker Clinical Trials in Lung Cancer: Design, Logistics, Challenges, and Practical Considerations. J Thorac Oncol. 2018 Nov;13(11):1625–37.
20. Liu S, Lee JJ. An overview of the design and conduct of the BATTLE trials. Chin Clin Oncol. 2015;4(3):13.
21. Bradbury P, Hilton J, Seymour L. Early-phase oncology clinical trial design in the era of molecularly targeted therapy: pitfalls and progress. Clin Investig. 2011 Jan;1(1):33–44.
22. Fountzilas E, Tsimberidou AM. Overview of precision oncology trials: challenges and opportunities. Expert Rev Clin Pharmacol. 2018 Aug 3;11(8):797–804.
23. Johnson DR, Galanis E. Incorporation of Prognostic and Predictive Factors Into Glioma Clinical Trials. Curr Oncol Rep. 2013 Feb;15(1):56–63.
24. Maitland ML, Schilsky RL. Clinical trials in the era of personalized oncology. CA Cancer J Clin. 2011 Nov;61(6):365–81.
25. Ocana A, Amir E, Vera-Badillo F, Seruga B, Tannock IF. Phase III Trials of Targeted Anticancer Therapies: Redesigning the Concept. Clin Cancer Res. 2013 Sep 15;19(18):4931–40.
26. Lindberg J, De Laere B, Crippa A, Eklund M, Grönberg H. ProBio: An outcome-adaptive, multi-arm, open-label, multiple assignment randomised controlled biomarker-driven trial in patients with metastatic castration-resistant prostate cancer. Ann Oncol. 2019 Oct;30:v354
27. Clinicaltrials.gov. ProBio: A Biomarker Driven Study in Patients With Metastatic Castrate Resistant Prostate Cancer (ProBio) [Internet]. Available from: https://clinicaltrials.gov/ct2/show/NCT03903835
28. Gronberg H, Eklund M, Lindberg J, Ullén A, Bjartell A, Andren O, et al. ProBio II: An adaptive and randomized multi-arm biomarker driven phase 2 study in men with castrate resistant prostate cancer (CRPC). J Clin Oncol. 2018; 36: TPS397
29. Talisa VB, Yende S, Seymour CW, Angus DC. Arguing for Adaptive Clinical Trials in Sepsis. Front Immunol. 2018 Jun 28;9:1502.
30. Rosenblum M, Hanley DF. Adaptive Enrichment Designs for Stroke Clinical Trials. Stroke. 2017 Jul;48(7):2021–5.
31. Antoniou M, Jorgensen AL, Kolamunnage-Dona R. Biomarker-Guided Adaptive Trial Designs in Phase II and Phase III: A Methodological Review. Soyer HP, editor. PLOS ONE. 2016 Feb 24;11(2):e0149803.
32. Zhang W, Wang J, Menon S. Advancing cancer drug development through precision medicine and innovative designs. J Biopharm Stat. 2018 Mar 4;28(2):229–44.
33. Antoniou M, Kolamunnage-Dona R, Wason J, Bathia R, Billingham C, Bliss JM, et al. Biomarker-guided trials: Challenges in practice. Contemp Clin Trials Commun. 2019

For peer review only

Dec;16:100493.
34. Grill J, Teuff GL, Nysom K, Blomgren K, Hargrave D, McCowage G, et al. PDCT-01. Biological medicine for diffuse intrinsic pontine gliomas eradication (BIOMEDE): Results of the three-arm biomarker-driven randomized trial in the first 230 patients from Europe and Australia. In Neuro-Oncology; p. vi183.
35. Debily M-A, Kergrohen T, Varlet P, Le Teuff G, Nysom K, Blomgren K, et al. PDTM-36. Whole exome sequencing (WES) of DIPG patients from the BIOMEDE trial reveals new prognostic subgroups with specific oncogenis programmes. In Neuro-Oncology; 2019. p. vi195.
36. PanACEA consortium, Phillips P, Hoelscher M, Bratton D, Rehal S, Heinrich N, et al. Modifying the multi-arm multi-stage (MAMS) design for use in a phase II tuberculosis trial in sub-Saharan Africa with a time-to-event primary outcome. Trials. 2013 Nov;14(S1):P26, 1745-6215-14-S1-P26.
37. Cafferkey C, Chau I, Thistlethwaite F, Petty RD, Starling N, WatkinsSheela Rao D, et al. PLATFORM: Planning treatment of oesophago-gastric (OG) cancer randomised maintenance therapy trial. In San Francisco, California; 2016.
38. Kaplan R. The FOCUS4 design for biomarker stratified trials. Chin Clin Oncol. 2015;4(3):1–10.
39. Simon R. Critical Review of Umbrella, Basket, and Platform Designs for Oncology Clinical Trials: Review of umbrella, basket, and platform trial designs. Clin Pharmacol Ther. 2017 Dec;102(6):934–41.
40. Bateman RJ, Benzinger TL, Berry S, Clifford DB, Duggan C, Fagan AM, et al. The DIAN-TU Next Generation Alzheimer's prevention trial: Adaptive design and disease progression model. Alzheimers Dement. 2017 Jan;13(1):8–19.
41. Parke T. D2.1. Report on Terminology, References, and Scenarios for Platform Trials and Master Protocols [Internet]. Berry Consultants; 2020 Jun. Available from: https://eu-pearl.eu/wp-content/uploads/2020/06/EU-PEARL_D2.1_Report-on-Terminology-and-Scenarios-for-Platform-Trials-and-Masterprotocols.pdf
42. Renfro LA, Mandrekar SJ. Definitions and statistical properties of master protocols for personalized medicine in oncology. J Biopharm Stat. 2018 Mar 4;28(2):217–28.
43. Simonsen KL, Fracasso PM, Bernstein SH, Wind-Rotolo M, Gupta M, Comprelli A, et al. The Fast Real-time Assessment of Combination Therapies in Immuno-ONcology (FRACTION) program: innovative, high-throughput clinical screening of immunotherapies. Eur J Cancer. 2018 Nov;103:259–66.
44. Aanur P, Gutierrez M, Kelly RJ, Ajani JA, Ku GY, Denlinger CS, et al. FRACTION (Fast Real-time Assessment of Combination Therapies in Immuno-Oncology)-gastric cancer (GC): A randomized, open-label, adaptive, phase 2 study of nivolumab in combination with other immuno-oncology (IO) agents in patients with advanced GC. In 2017.
45. Fracasso PM, Freeman DJ, Simonsen K, Shen Y, Gupta M, Comprelli A, et al. A phase 2, fast real-time assessment of combination therapies in immuno-oncology trial in patients with advanced non-small cell lung cancer (FRACTION-lung). Ann Oncol. 2016 Oct;27:vi451.
46. Alexander BM, Ba S, Berger MS, Berry DA, Cavenee WK, Chang SM, et al. Adaptive Global Innovative Learning Environment for Glioblastoma: GBM AGILE. Clin Cancer Res. 2018 Feb 15;24(4):737–43.
47. Alexander BM, Trippa L, Gaffey S, Arrillaga-Romany IC, Lee EQ, Rinne ML, et al. Individualized Screening Trial of Innovative Glioblastoma Therapy (INSIGhT): A Bayesian Adaptive Platform Trial to Develop Precision Medicines for Patients With Glioblastoma. JCO Precis Oncol. 2019 Dec;(3):1–13.
48. Spigel D, Garassino M, Besse B, Sacher A, Barve M, Cousin S, et al. P1.01-110 Novel Regimens Versus Standard-of-Care in NSCLC: A Phase II, Randomized, Open-Label, Platform Trial Using a Master Protocol. J Thorac Oncol. 2019 Oct;14(10):S404–S405
49. Yu H, Goldberg S, Le X, Piotrowska Z, Smith P, Mensi I, et al. P2.01-22 ORCHARD: A Phase II Platform Study in Patients with Advanced NSCLC Who Have Progressed on First-Line Osimertinib Therapy. J Thorac Oncol. 2019 Oct;14(10):S647.
50. Joshi SS, Maron SB, Lomnicki S, Polite BN, Sharma M, Ibe J, et al. Personalized antibodies for gastroesophageal adenocarcinoma (PANGEA): A phase II precision medicine trial (NCT02213289) .J Clin Oncol. 2018; 36: TPS198
51. Weber J, Long GV, Haanen JB, Arance A, Dummer R, Nathan P, et al. A randomized, open-label, phase II open platform study evaluating the efficacy and safety of novel spartalizumab (PDR001) combinations in previously treated unresectable or metastatic melanoma (PLATForM). Ann Oncol. 2018;29:viii442-viii466
52. Leonetti A, Boyd L, Giuliani J, Giovannetti E, Garajová I. Light and shadow on innovative clinical trial designs: reflections from the EORTC-PAMM course on 'preclinical and early-phase clinical pharmacology.' Expert Rev Clin Pharmacol. 2019 Nov 2;12(11):1033–6.
53. Park JJH, Siden E, Zoratti MJ, Dron L, Harari O, Singer J, et al. Systematic review of basket trials, umbrella trials, and platform trials: a landscape analysis of master protocols. Trials. 2019 Dec;20(1):572.
54. Gilson C, Chowdhury S, Parmar MKB, Sydes MR. Incorporating Biomarker Stratification into STAMPEDE: an Adaptive Multi-arm, Multi-stage Trial Platform. Clin Oncol. 2017 Dec;29(12):778–86.
55. Semler MW, Bernard GR, Aaron SD, Angus DC, Biros MH, Brower RG, et al. Identifying Clinical Research Priorities in Adult Pulmonary and Critical Care. NHLBI Working Group Report. Am J Respir Crit Care Med. 2020 Aug 15;202(4):511–23.
56. Skamene T, Siu LL, Renouf DJ, Laskin JJ, Bedard PL, Jones SJM, et al. Canadian profiling and targeted agent utilization trial (CAPTUR/PM.1): A phase II basket precision medicine trial. J Clin Oncol 2018. 36: TPS12127
57. Hernandez-Martinez J-M, Sánchez-Reyes R, De la Garza-Salazar JG, Arrieta O. Onco-omics Approaches and Applications in Clinical Trials for Cancer Patients. In:

For peer review only

Translational Research and Onco-Omics Applications in the Era of Cancer Personal Genomics. Springer; 2019. p. 79–90.
58. Lopez-Chavez A, Thomas A, Rajan A, Raffeld M, Morrow B, Kelly R, et al. Molecular Profiling and Targeted Therapy for Advanced Thoracic Malignancies: A Biomarker-Derived, Multiarm, Multihistology Phase II Basket Trial. J Clin Oncol. 2015 Mar 20;33(9):1000–7.
59. Patel SP, Othus M, Chae YK, Giles FJ, Hansel DE, Singh PP, et al. A Phase II Basket Trial of Dual Anti–CTLA-4 and Anti–PD-1 Blockade in Rare Tumors (DART SWOG 1609) in Patients with Nonpancreatic Neuroendocrine Tumors. Clin Cancer Res. 2020 May 15;26(10):2290–6.
60. Timmers L, van Waalwijk van Doorn S, Pisters A, van Saase L, Voest E. PPM1 A RISK SHARING MODEL FOR BIOMARKER-DRIVEN TREATMENT OF RARE SUBGROUPS OF CANCER PATIENTS. Value Health. 2019 Nov;22:S837.
61. D'Angelo S, Blay J, Chow W, Demetri G, Thistlethwaite F, Wagner M. Autologous T cells with NY-ESO-1-specific T-cell receptor (GSK3377794) in HLA-A*02+previously-treated and -untreated advanced metastatic/unresectable synovial sarcoma: A master protocol study design. In Journal for Immunotherapy of Cancer; 2019. p. 282.
62. Cecchini M, Rubin EH, Blumenthal GM, Ayalew K, Burris HA, Russell-Einhorn M, et al. Challenges with Novel Clinical Trial Designs: Master Protocols. Clin Cancer Res. 2019 Apr 1;25(7):2049–57.
63. Marabelle A, Le DT, Ascierto PA, Di Giacomo AM, De Jesus-Acosta A, Delord J-P, et al. Efficacy of Pembrolizumab in Patients With Noncolorectal High Microsatellite Instability/Mismatch Repair–Deficient Cancer: Results From the Phase II KEYNOTE-158 Study. J Clin Oncol. 2020 Jan 1;38(1):1–10.
64. Bang Y-J, Kaufman B, Geva R, Stemmer SM, Hong S-H, Lee J-S, et al. An open-label, phase II basket study of olaparib and durvalumab (MEDIOLA): Results in patients with relapsed gastric cancer. J Clin Oncol. 2019;37:140
65. Domchek SM, Postel-Vinay S, Im S-A, Hee Park Y, Delord J-P, Italiano A, et al. An open-label, phase II basket study of olaparib and durvalumab (MEDIOLA): Updated results in patients with germline BRCA-mutated (gBRCAm) metastatic breast cancer (MBC). Cancer Res. 2019;79: PD5-04
66. Krebs M, Ross K, Kim S, De Jonge M, Barlesi F, Postel-Vinay S, et al. P1.15-004 An Open-Label, Multitumor Phase II Basket Study of Olaparib and Durvalumab (MEDIOLA): Results in Patients with Relapsed SCLC. J Thorac Oncol. 2017 Nov;12(11):S2044–5.
67. Hyman DM, Taylor BS, Baselga J. Implementing Genome-Driven Oncology. Cell. 2017 Feb;168(4):584–99.
68. Thavaneswaran S, Sebastian L, Ballinger M, Best M, Hess D, Lee CK, et al. Cancer Molecular Screening and Therapeutics (MoST): a framework for multiple, parallel signal-seeking studies of targeted therapies for rare and neglected cancers. Med J Aust. 2018 Oct;209(8):354–5.
69. Thavaneswaran S, Sebastian L, Ballinger M, Cowley M, Grady J, Joshua A, et al. The Cancer Molecular Screening and Therapeutics Program (MoST) – A molecular screening platform with multiple, parallel, signal-seeking therapeutic substudies. In Annals of Oncology; 2018. p. viii133–48.
70. Conter HJ, MacDonald LD, Fiset S, Bramhecha YM, Chaney M, Rosu GN. Safety and efficacy results of the combination of DPX-Survivac, pembrolizumab and intermittent low dose cyclophosphamide (CPA) in subjects with advanced and metastatic solid tumours: Preliminary results from the hepatocellular carcinoma (HCC), NSCLC, bladder cancer, & MSI-H cohorts. Ann Oncol. 2019 Oct;30:v494.
71. Li BT, Zauderer M, Chaft J, Drilon A, Eng J, Sima C, et al. Ado-trastuzumab emtansine for HER2 amplified or HER2 overexpressed cancers: A phase II "basket" trial. Cancer Res. 2015; 75: CT225
72. Barroilhet L, Matulonis U. The NCI-MATCH trial and precision medicine in gynecologic cancers. Gynecol Oncol. 2018 Mar;148(3):585–90.
73. Do K, Coyne GO, Chen AP. An overview of the NCI precision medicine trials—NCI MATCH and MPACT. Chin Clin Oncol. 2015;4(3):8.
74. Mandrekar SJ, Sargent DJ. All-Comers versus Enrichment Design Strategy in Phase II Trials. J Thorac Oncol. 2011 Apr;6(4):658–60.
75. Mazzarella L, Morganti S, Marra A, Trapani D, Tini G, Pelicci P, et al. Master protocols in immuno-oncology: do novel drugs deserve novel designs? J Immunother Cancer. 2020 Mar;8(1):e000475.
76. Moore A, Jones R. Supporting and enhancing peer review in the *BJGP*. Br J Gen Pract. 2014 Jul;64(624):e459–61.
77. O'Brien C, Carter L, Cook N, Dean E. Novel Early Phase Clinical Trial Design in Oncology. Pharm Med. 2017 Oct;31(5):297–307.
78. Renfro LA, Sargent DJ. Statistical controversies in clinical research: basket trials, umbrella trials, and other master protocols: a review and examples. Ann Oncol. 2017 Jan;28(1):34–43.
79. Simon R. Genomic Alteration–Driven Clinical Trial Designs in Oncology. Ann Intern Med. 2016 Aug 16;165(4):270.
80. Soldatos, Kaduthanam, Jackson. Precision Oncology—The Quest for Evidence. J Pers Med. 2019 Sep 5;9(3):43.
81. Zardavas D, Piccart-Gebhart M. Clinical Trials of Precision Medicine through Molecular Profiling: Focus on Breast Cancer. Am Soc Clin Oncol Educ Book. 2015 May;(35):e183–90.
82. Simon R. New designs for basket clinical trials in oncology. J Biopharm Stat. 2018 Mar 4;28(2):245–55.
83. Coyne GO, Takebe N, Chen AP. Defining precision: The precision medicine initiative trials NCI-MPACT and NCI-MATCH. Curr Probl Cancer. 2017 May;41(3):182–93.
84. Tao JJ, Schram AM, Hyman DM. Basket Studies: Redefining Clinical Trials in the Era of Genome-Driven Oncology. Annu Rev Med. 2018 Jan 29;69(1):319–31.
85. Nakamura Y, Komatsu Y, Kato K, Shinozaki E, Bando H, Kato T, et al. bTMB-High Basket trial: A multicenter phase II trial of nivolumab monotherapy in patients with

For peer review only

advanced gastrointestinal cancers with high blood tumor mutational burden (bTMB). J Clin Oncol 2019; 37: TPS179
86. Kummar S, Chen A, Lih J, Williams M, Rubinstein L, Conley B, et al. SP007 Molecular profiling based assignment of cancer therapy (MPACT). Eur J Cancer. 2013 Nov;49:S3.
87. Ou F-S, An M-W, Ruppert AS, Mandrekar SJ. Discussion of Trial Designs for Biomarker Identification and Validation Through the Use of Case Studies. JCO Precis Oncol. 2019 Dec;(3):1–10.
88. Verweij J, Hendriks HR, Zwierzina H, Hanauske, Wacheck V, Collignon O, et al. Innovation in oncology clinical trial design. Cancer Treat Rev. 2019 Mar;74:15–20.
89. Brana I, Massard C, Baird RD, Opdam F, Schlenk RF, De Petris L, et al. Basket of baskets (BoB): A modular, open label, phase II, multicenter study to evaluate targeted agents in molecularly selected populations with advanced solid tumors. J Clin Oncol. 2019; 37: TPS3151
90. Garralda E, Dienstmann R, Piris-Giménez A, Braña I, Rodon J, Tabernero J. New clinical trial designs in the era of precision medicine. Mol Oncol. 2019 Mar;13(3):549–57.
91. Hofmann D, Nitz U, Gluz O, Kates RE, Schinkoethe T, Staib P, et al. WSG ADAPT – adjuvant dynamic marker-adjusted personalized therapy trial optimizing risk assessment and therapy response prediction in early breast cancer: study protocol for a prospective, multi-center, controlled, non-blinded, randomized, investigator initiated phase II/III trial. Trials. 2013;14(1):261.
92. Nitz U, Gluz O, von Schumann R, Hofmann D, Kates RE, Kuemmel S, et al. ADAPT - Adjuvant Dynamic marker-Adjusted Personalized Therapy trial optimizing risk assessment and therapy response prediction in early breast cancer. Cancer Res. 2015; 75: OT3-2-04
93. Cochrane Library. Trial for the optimisation of risk assessment and therapy success predicition in patients with early breast cancer by the use of biomarkers in advance to therapy decission-making to personalize therapies [Internet]. Available from: https://www.cochranelibrary.com/central/doi/10.1002/central/CN-01873376/full
94. Abrams J, Conley B, Mooney M, Zwiebel J, Chen A, Welch JJ, et al. National Cancer Institute's Precision Medicine Initiatives for the New National Clinical Trials Network. Am Soc Clin Oncol Educ Book. 2014 May;(34):71–6.
95. Yee LM, McShane LM, Freidlin B, Mooney MM, Korn EL. Biostatistical and Logistical Considerations in the Development of Basket and Umbrella Clinical Trials: Cancer J. 2019;25(4):254–63.
96. Heckman-Stoddard BM, Smith JJ. Precision Medicine Clinical Trials: Defining New Treatment Strategies. Semin Oncol Nurs. 2014 May;30(2):109–16.
97. Ferrarotto R, Redman MW, Gandara DR, Herbst RS, Papadimitrakopoulou V. Lung-MAP-framework, overview, and design principles. Chin Clin Oncol. 2015;4(3):1–6.
98. Gandara DR, Hammerman PS, Sos ML, Lara PN, Hirsch FR. Squamous Cell Lung Cancer: From Tumor Genomics to Cancer Therapeutics. Clin Cancer Res. 2015 May 15;21(10):2236–43.
99. Lam VK, Papadimitrakopoulou V. Master protocols in lung cancer: experience from Lung Master Protocol. Curr Opin Oncol. 2018 Mar;30(2):92–7.
100. Hollingsworth SJ. Precision medicine in oncology drug development: a pharma perspective. Drug Discov Today. 2015 Dec;20(12):1455–63.
101. Redman MW, Allegra CJ. The Master Protocol Concept. Semin Oncol. 2015 Oct;42(5):724–30.
102. Fennell D, Hudka M, Darlison L, Lord K, Bzura A, Dzialo J, et al. P2.06-02 Mesothelioma Stratified Therapy (MiST): A Phase IIA Umbrella Trial for Accelerating the Development of Precision Medicines. J Thorac Oncol. 2019 Oct;14(10):S755–6.
103. Schmoll H-J, Arnold D, de Gramont A, Ducreux M, Grothey A, O'Dwyer PJ, et al. MODUL—a multicenter randomized clinical trial of biomarker-driven maintenance therapy following first-line standard induction treatment of metastatic colorectal cancer: an adaptable signal-seeking approach. J Cancer Res Clin Oncol. 2018 Jun;144(6):1197–204.
104. Zhou Q, Zhang X-C, Tu H-Y, Gan B, Wang B-C, Xu C-R, et al. Biomarker-integrated study of single agent targeting molecular alterations of PI3KCA, MET, ALK, ROS1, KRAS, NRAS or BRAF in advanced NSCLC: Phase 2 umbrella trial in China (CTONG1505). Ann Oncol. 2018 Nov;29:ix113..
105. Park JJH, Hsu G, Siden EG, Thorlund K, Mills EJ. An overview of precision oncology basket and umbrella trials for clinicians. CA Cancer J Clin. 2020 Mar;70(2):125–37.
106. Bell S, Copel J, Smith A. The pros and cons of an "umbrella" trial design for a rare disease from a trial management and data management perspective. Trials 2017; 18(Suppl 1): 200
107. Sebag-Montefiore D, Adams R, Bell S, Berkman L, Gilbert DC, Glynne-Jones R, et al. The Development of an Umbrella Trial (PLATO) to Address Radiation Therapy Dose Questions in the Locoregional Management of Squamous Cell Carcinoma of the Anus. Int J Radiat Oncol. 2016 Oct;96(2):E164–5.
108. ISRCTN Registry. Precision Panc: Advancing personalised medicine treatment strategies for pancreatic cancer [Internet]. Available from: https://www.isrctn.com/ISRCTN14879538
109. Grose DB, McKay CJ, Cooke S, Graham JS, Duthie F, Jamieson N, et al. PRIMUS-002: A multicentre, open-label, phase II study examining FOLFOX and nab-paclitaxel (FA) and nab-paclitaxel and gemcitabine (AG) as neoadjuvant therapy for (borderline) resectable pancreatic cancer (PC), focusing on biomarker and liquid biopsy development. J Clin Oncol. 2019; 37: TPS4166
110. Park S, Hur JY, Yoon SE, Lee K, Kim Y, Cho JH, et al. P2.12-05 SUKSES (Small Cell Lung Cancer Umbrella Korea Studies): A Phase II Biomarker-Driven Umbrella Study in Relapsed or Refractory SCLC. J Thorac Oncol. 2018 Oct;13(10):S792.

For peer review only

111. Park S, Shim J, Jung HA, Sun J-M, Lee S-H, Park W-Y, et al. Biomarker driven phase II umbrella trial study of AZD1775, AZD2014, AZD2811 monotherapy in relapsed small cell lung cancer. J Clin Oncol 2019: 37: 8514.
112. Galot R, Le Tourneau C, Saada-Bouzid E, Daste A, Even C, Debruyne PR, et al. A phase II study of monalizumab in patients with recurrent/metastatic (RM) squamous cell carcinoma of the head and neck (SCCHN): Results of the I1 cohort of the EORTC-HNCG-1559 trial (UPSTREAM). Ann Oncol. 2019 Oct;30:v449–50.
113. Lee J, Kim ST, Kim K, Lee H, Kozarewa I, Mortimer PG, et al. Tumor genomic profiling guides metastatic gastric cancer patients to targeted treatment: The VIKTORY Umbrella Trial. Cancer Discov. 2019 Jul 17;CD-19-0442.
114. De Mattos-Arruda L, Rodon J. Pilot Studies for Personalized Cancer Medicine: Focusing on the Patient for Treatment Selection. The Oncologist. 2013 Nov;18(11):1180–8.
115. Lee J-Y, Yi JY, Kim H-S, Lim J, Kim S, Nam BH, et al. An umbrella study of biomarker-driven targeted therapy in patients with platinum-resistant recurrent ovarian cancer: a Korean Gynecologic Oncology Group study (KGOG 3045), AMBITION. Jpn J Clin Oncol. 2019 Aug 1;49(8):789–92.
116. Noda S, Yonemori K, Shirakawa N, Okuma HS, Shimizu T, Hirakawa A, et al. MASTER KEY project: A basket/umbrella trial for rare cancers in Japan.Ann Oncol. 2018 Oct;29:viii148

For peer review only

Supplementary file VI. Trials evaluating personalised versus no personalised medicine

Type of trial designs	Example(s)	Trial registration num.	Recruitment status as of 12 March 2021	Clinical Field	Phase	References
Adaptive strategy designs for biomarkers with measurement error	OPTIMA	ISRCTN42400492	Ongoing	Breast Cancer	n/a[1]	(1)
Basket design	NCI-MPACT	NCT01827384	Completed	Advanced malignant solid neoplasm	II	(2–4)
	SHIVA	NCT01771458	Unknown*	Reccurent/Metastatic Solid; Tumor Disease	II	(5)
	IMPACT II	NCT02152254	Completed	Reccurent/Metastatic Solid; Tumor Disease	II	(6)
Biomarker strategy design with biomarker assessment in the control arm	ERCC1	NCT00801736	Completed	Lung cancer	III	(7)
	GILT docetaxel	NCT00174629	Completed	Lung cancer	III	(8)
	LIFT	NCT02498977	Completed	Transplantation, Liver	IV	(9)
Biomarker-strategy design without biomarker assessment in the control arm	GUIDE-IT	NCT01685840	Completed	Chronic Heart Failure	n/a[1]	(10)
	iPEGASUS	NCT03021525	Ongoing	Hemodynamic Instability, Cardiac Output (High), Peroperative Complication	n/a[1]	(11)
	OCTOPUS	ISRCTN81464462	Completed	Mild head injury	n/a[1]	(8)
	PUFFIN	NCT03654508	Ongoing	Asthma	n/a[1]	(12)
Modified biomarker	SHIVA	NCT01771458	Unknown*	Reccurent/Metastatic Solid; Tumor Disease	II	(1,13–15)

For peer review only

strategy design	NCI-MPACT	NCT01827384	Completed	Advanced malignant solid neoplasm	II	(15)
Outcome-based adaptive randomization design	ProBio	NCT03903835	Ongoing	Prostate cancer	III	(16)
Platform	SHIVA	NCT01771458	Unknown*	Reccurent/Metastatic Solid; Tumor Disease	II	(17)
Sequential Multiple Assignment Randomized Trial (SMART)	Siyaphambili Study	NCT03500172	Ongoing	HIV	n/a[1]	(18)
Umbrella	UPSTREAM	NCT03088059	Ongoing	Head and Neck Squamous Cell Carcinoma	II	(19)
	SAFIR02_Braest	NCT02299999	Completed	Breast Cancer	II	(20)
	SAFIR02_Lung	NCT02117167	Completed	Lung cancer	II	(17)

[1]Not applicable is used on the Clinicaltrilas.gov website to describe trials without FDA-defined phases including trials of devices or behavioural interventions.

References

1. Renfro LA, Mallick H, An M-W, Sargent DJ, Mandrekar SJ. Clinical trial designs incorporating predictive biomarkers. Cancer Treat Rev. 2016 Feb;43:74–82.
2. Coyne GO, Takebe N, Chen AP. Defining precision: The precision medicine initiative trials NCI-MPACT and NCI-MATCH. Curr Probl Cancer. 2017 May;41(3):182–93.
3. Lopez-Chavez A, Thomas A, Rajan A, Raffeld M, Morrow B, Kelly R, et al. Molecular Profiling and Targeted Therapy for Advanced Thoracic Malignancies: A Biomarker-Derived, Multiarm, Multihistology Phase II Basket Trial. J Clin Oncol. 2015 Mar 20;33(9):1000–7.
4. Simon R. New designs for basket clinical trials in oncology. J Biopharm Stat. 2018 Mar 4;28(2):245–55.
5. Tao JJ, Schram AM, Hyman DM. Basket Studies: Redefining Clinical Trials in the Era of Genome-Driven Oncology. Annu Rev Med. 2018 Jan 29;69(1):319–31.
6. Fountzilas E, Tsimberidou AM. Overview of precision oncology trials: challenges and opportunities. Expert Rev Clin Pharmacol. 2018 Aug 3;11(8):797–804.
7. Freidlin B, McShane LM, Korn EL. Randomized Clinical Trials With Biomarkers: Design Issues. JNCI J Natl Cancer Inst. 2010 Feb 3;102(3):152–60.
8. Antoniou M, Kolamunnage-Dona R, Jorgensen A. Biomarker-Guided Non-Adaptive Trial Designs in Phase II and Phase III: A Methodological Review. J Pers Med. 2017 Jan 25;7(1):1.
9. Clinicaltrials.gov. Liver Immunosuppression Free Trial (LIFT) [Internet]. Available from: https://clinicaltrials.gov/ct2/show/NCT02498977
10. Ahmad T, O'Connor CM. Therapeutic Implications of Biomarkers in Chronic Heart Failure. Clin Pharmacol Ther. 2013 Oct;94(4):468–79.
11. Funcke S, Saugel B, Koch C, Schulte D, Zajonz T, Sander M, et al. Individualized, perioperative, hemodynamic goal-directed therapy in major abdominal surgery (iPEGASUS trial): study protocol for a randomized controlled trial. Trials. 2018 Dec;19(1):273.
12. Vijverberg SJ, Pijnenburg MW, Hövels AM, Koppelman GH, Maitland-van der Zee A-H. The need for precision medicine clinical trials in childhood asthma: rationale and design of the PUFFIN trial. Pharmacogenomics. 2017 Mar;18(4):393–401.

For peer review only

13. Dienstmann R, Rodon J, Tabernero J. Optimal design of trials to demonstrate the utility of genomically-guided therapy: Putting Precision Cancer Medicine to the test. Mol Oncol. 2015 May;9(5):940–50.
14. Paoletti X, Asselain B, Kamal M, Servant N, Huppé P, Bieche I, et al. Design and statistical principles of the SHIVA trial. Chin Clin Oncol. 2015;4(3):1–10.
15. Renfro LA, An M-W, Mandrekar SJ. Precision oncology: A new era of cancer clinical trials. Cancer Lett. 2017 Feb;387:121–6.
16. Lindberg J, De Laere B, Crippa A, Eklund M, Grönberg H. ProBio: An outcome-adaptive, multi-arm, open-label, multiple assignment randomised controlled biomarker-driven trial in patients with metastatic castration-resistant prostate cancer. In Barcelona, Spain;
17. Leonetti A, Boyd L, Giuliani J, Giovannetti E, Garajová I. Light and shadow on innovative clinical trial designs: reflections from the EORTC-PAMM course on 'preclinical and early-phase clinical pharmacology.' Expert Rev Clin Pharmacol. 2019 Nov 2;12(11):1033–6.
18. Clinicaltrials.gov. HIV Treatment Retention Interventions for Women Living With HIV (Siyaphambili Study) [Internet]. Available from: https://clinicaltrials.gov/ct2/show/NCT03500172
19. Galot R, Le Tourneau C, Saada-Bouzid E, Daste A, Even C, Debruyne PR, et al. A phase II study of monalizumab in patients with recurrent/metastatic (RM) squamous cell carcinoma of the head and neck (SCCHN): Results of the I1 cohort of the EORTC-HNCG-1559 trial (UPSTREAM). Ann Oncol. 2019 Oct;30:v449–50.
20. Hernandez-Martinez J-M, Sánchez-Reyes R, De la Garza-Salazar JG, Arrieta O. Onco-omics Approaches and Applications in Clinical Trials for Cancer Patients. In: Translational Research and Onco-Omics Applications in the Era of Cancer Personal Genomics. Springer; 2019. p. 79–90.

For peer review only

Preferred Reporting Items for Systematic reviews and Meta-Analyses extension for Scoping Reviews (PRISMA-ScR) Checklist

SECTION	ITEM	PRISMA-ScR CHECKLIST ITEM	REPORTED ON PAGE #
**TITLE**			
Title	1	Identify the report as a scoping review.	1
**ABSTRACT**			
Structured summary	2	Provide a structured summary that includes (as applicable): background, objectives, eligibility criteria, sources of evidence, charting methods, results, and conclusions that relate to the review questions and objectives.	2
**INTRODUCTION**			
Rationale	3	Describe the rationale for the review in the context of what is already known. Explain why the review questions/objectives lend themselves to a scoping review approach.	3
Objectives	4	Provide an explicit statement of the questions and objectives being addressed with reference to their key elements (e.g., population or participants, concepts, and context) or other relevant key elements used to conceptualize the review questions and/or objectives.	3
**METHODS**			
Protocol and registration	5	Indicate whether a review protocol exists; state if and where it can be accessed (e.g., a Web address); and if available, provide registration information, including the registration number.	4
Eligibility criteria	6	Specify characteristics of the sources of evidence used as eligibility criteria (e.g., years considered, language, and publication status), and provide a rationale.	4
Information sources*	7	Describe all information sources in the search (e.g., databases with dates of coverage and contact with authors to identify additional sources), as well as the date the most recent search was executed.	4
Search	8	Present the full electronic search strategy for at least 1 database, including any limits used, such that it could be repeated.	Supplementary file 1
Selection of sources of evidence†	9	State the process for selecting sources of evidence (i.e., screening and eligibility) included in the scoping review.	4
Data charting process‡	10	Describe the methods of charting data from the included sources of evidence (e.g., calibrated forms or forms that have been tested by the team before their use, and whether data charting was done independently or in duplicate) and any processes for obtaining and confirming data from investigators.	5
Data items	11	List and define all variables for which data were sought and any assumptions and simplifications made.	5
Critical appraisal of individual sources of evidence§	12	If done, provide a rationale for conducting a critical appraisal of included sources of evidence; describe the methods used and how this information was used in any data synthesis (if appropriate).	na
Synthesis of results	13	Describe the methods of handling and summarizing the data that were charted.	5

SECTION	ITEM	PRISMA-ScR CHECKLIST ITEM	REPORTED ON PAGE #
**RESULTS**			
Selection of sources of evidence	14	Give numbers of sources of evidence screened, assessed for eligibility, and included in the review, with reasons for exclusions at each stage, ideally using a flow diagram.	Figure 1
Characteristics of sources of evidence	15	For each source of evidence, present characteristics for which data were charted and provide the citations.	6 - Suppl. file 3
Critical appraisal within sources of evidence	16	If done, present data on critical appraisal of included sources of evidence (see item 12).	na
Results of individual sources of evidence	17	For each included source of evidence, present the relevant data that were charted that relate to the review questions and objectives.	Supplementary files 4-5-6
Synthesis of results	18	Summarize and/or present the charting results as they relate to the review questions and objectives.	5-6-7-8-9
**DISCUSSION**			
Summary of evidence	19	Summarize the main results (including an overview of concepts, themes, and types of evidence available), link to the review questions and objectives, and consider the relevance to key groups.	9
Limitations	20	Discuss the limitations of the scoping review process.	10
Conclusions	21	Provide a general interpretation of the results with respect to the review questions and objectives, as well as potential implications and/or next steps.	10
**FUNDING**			
Funding	22	Describe sources of funding for the included sources of evidence, as well as sources of funding for the scoping review. Describe the role of the funders of the scoping review.	14

JBI = Joanna Briggs Institute; PRISMA-ScR = Preferred Reporting Items for Systematic reviews and Meta-Analyses extension for Scoping Reviews.

* Where *sources of evidence* (see second footnote) are compiled from, such as bibliographic databases, social media platforms, and Web sites.

† A more inclusive/heterogeneous term used to account for the different types of evidence or data sources (e.g., quantitative and/or qualitative research, expert opinion, and policy documents) that may be eligible in a scoping review as opposed to only studies. This is not to be confused with *information sources* (see first footnote).

‡ The frameworks by Arksey and O'Malley (6) and Levac and colleagues (7) and the JBI guidance (4, 5) refer to the process of data extraction in a scoping review as data charting.

§ The process of systematically examining research evidence to assess its validity, results, and relevance before using it to inform a decision. This term is used for items 12 and 19 instead of "risk of bias" (which is more applicable to systematic reviews of interventions) to include and acknowledge the various sources of evidence that may be used in a scoping review (e.g., quantitative and/or qualitative research, expert opinion, and policy document).

From: Tricco AC, Lillie E, Zarin W, O'Brien KK, Colquhoun H, Levac D, et al. PRISMA Extension for Scoping Reviews (PRISMAScR): Checklist and Explanation. Ann Intern Med. 2018;169:467–473. doi: 10.7326/M18-0850.

For peer review only - http://bmjopen.bmj.com/site/about/guidelines.xhtml

Study designs for clinical trials applied to personalised medicine: a scoping review

Journal:	*BMJ Open*
Manuscript ID	bmjopen-2021-052926.R1
Article Type:	Original research
Date Submitted by the Author:	24-Jan-2022

Complete List of Authors:	Superchi, Cecilia; Université de Paris<br>Brion Bouvier, Florie; Université de Paris<br>Gerardi, Chiara; Istituto di Ricerche Farmacologiche Mario Negri, Center for Health Regulatory Policies<br>Carmona, Montserrat; Instituto de Salud Carlos III, Agencia de Evaluación de Tecnologias Sanitarias; Red de Investigación en Servicios de Salud en Enfermedades Crónicas (REDISSEC)<br>San Miguel, Lorena; Belgian Health Care Knowledge Centre (KCE)<br>Sanchez-Gomez, Luis; Instituto de Salud Carlos III, Agencia de Evaluación de Tecnologias Sanitarias; Red de Investigación en Servicios de Salud en Enfermedades Crónicas (REDISSEC)<br>Imaz-Iglesia, Iñaki; Instituto de Salud Carlos III, Agencia de Evaluación de Tecnologias Sanitarias; Red de Investigación en Servicios de Salud en Enfermedades Crónicas (REDISSEC)<br>Garcia, Paula; European Clinical Research Infrastructure Network (ECRIN)<br>Demotes, Jacques; European Clinical Research Infrastructure Network (ECRIN)<br>Banzi, Rita; Istituto di Ricerche Farmacologiche Mario Negri, Center for Health Regulatory Policies<br>Porcher, Raphaël; Université de Paris

**Primary Subject Heading**:	Research methods
Secondary Subject Heading:	Patient-centred medicine, Epidemiology
Keywords:	EPIDEMIOLOGY, STATISTICS & RESEARCH METHODS, PUBLIC HEALTH

SCHOLARONE™
Manuscripts

I, the Submitting Author has the right to grant and does grant on behalf of all authors of the Work (as defined in the below author licence), an exclusive licence and/or a non-exclusive licence for contributions from authors who are: i) UK Crown employees; ii) where BMJ has agreed a CC-BY licence shall apply, and/or iii) in accordance with the terms applicable for US Federal Government officers or employees acting as part of their official duties; on a worldwide, perpetual, irrevocable, royalty-free basis to BMJ Publishing Group Ltd ("BMJ") its licensees and where the relevant Journal is co-owned by BMJ to the co-owners of the Journal, to publish the Work in this journal and any other BMJ products and to exploit all rights, as set out in our licence.

The Submitting Author accepts and understands that any supply made under these terms is made by BMJ to the Submitting Author unless you are acting as an employee on behalf of your employer or a postgraduate student of an affiliated institution which is paying any applicable article publishing charge ("APC") for Open Access articles. Where the Submitting Author wishes to make the Work available on an Open Access basis (and intends to pay the relevant APC), the terms of reuse of such Open Access shall be governed by a Creative Commons licence – details of these licences and which Creative Commons licence will apply to this Work are set out in our licence referred to above.

Other than as permitted in any relevant BMJ Author's Self Archiving Policies, I confirm this Work has not been accepted for publication elsewhere, is not being considered for publication elsewhere and does not duplicate material already published. I confirm all authors consent to publication of this Work and authorise the granting of this licence.

For peer review only

Title

Study designs for clinical trials applied to personalised medicine: a scoping review

Authors

Cecilia Superchi[1], Florie Brion Bouvier[1], Chiara Gerardi[2], Montserrat Carmona[3,4], Lorena San Miguel[5], Luis María Sánchez-Gómez[3,4], Iñaki Imaz-Iglesia[3.4], Paula Garcia[6], Jacques Demotes-Mainard[6], Rita Banzi[2], Raphaël Porcher[1], the PERMIT Group

Affiliations

1. Centre of Research in Epidemiology and Statistics, Université de Paris (CRESS-UMR1153), Paris, France
2. Center for Health Regulatory Policies, Istituto di Ricerche Farmacologiche Mario Negri IRCCS, Milan, Italy
3. Agencia de Evaluación de Tecnologias Sanitarias (AETS), Instituto de Salud Carlos III, Madrid, Spain
4. Red de Investigación en Servicios de Salud en Enfermedades Crónicas (REDISSEC). Madrid, Spain
5. Belgian Health Care Knowledge Centre (KCE), Brussel, Belgium
6. European Clinical Research Infrastructure Network (ECRIN), Paris, France

Corresponding author:

Cecilia Superchi

Centre de Recherche Épidémiologie et StatistiqueS Université de Paris (CRESS-UMR1153)

Hôpital Hôtel-Dieu, 1 Place du Parvis Notre-Dame, 75004 Paris, France

cecilia.superchi@parisdescartes.fr | cecilia.superchi@gmail.com

ORCID IDs

Cecilia Superchi: https://orcid.org/0000-0002-5375-6018

Florie Brion Bouvier https://orcid.org/0000-0001-6364-6106

Chiara Gerardi https://orcid.org/0000-0002-2459-4769

Montserrat Carmona Rodriguez https://orcid.org/0000-0002-4680-7745

Lorena San Miguel https://orcid.org/0000-0002-7155-7303

Luis María Sánchez-Gómez https://orcid.org/0000-0003-4358-6032

Iñaki Imaz-Iglesia https://orcid.org/0000-0002-7864-419

Paula Garcia

Jacques Demotes-Mainard: https://orcid.org/0000-0002-0807-0746

Rita Banzi https://orcid.org/0000-0002-2211-3300

Raphaël Porcher: https://orcid.org/0000-0002-5277-4679

Word count: 300 (abstract, word limit: 300), 4907 (main text, word limit: 4000)

For peer review only

Abstract

Objective: Personalised medicine (PM) allows treating patients based on their individual demographic, genomic or biological characteristics for tailoring the *'right treatment for the right person at the right time'*. Robust methodology is required for PM clinical trials, to correctly identify groups of participants and treatments. As an initial step for the development of new recommendations on trial designs for PM, we aimed to present an overview of the study designs that have been used in this field.

Design: Scoping review

Methods: We searched (April 2020) PubMed, EMBASE and the Cochrane Library for all reports in English, French, German, Italian and Spanish, describing study designs for clinical trials applied to PM. Study selection and data extraction were performed in duplicate resolving disagreements by consensus or by involving a third expert reviewer. We extracted information on the characteristics of trial designs and examples of current applications of these approaches. The extracted information was used to generate a new classification of trial designs for PM.

Results: We identified 21 trial designs, 10 subtypes, and 30 variations of trial designs applied to PM, which we classified into four core categories (namely, Master protocol, Randomise-all, Biomarker strategy and Enrichment). We found 131 clinical trials using these designs, of which the great majority were master protocols (86/131, 65.6%). Most of the trials were phase II studies (75/131, 57.2%) in the field of oncology (113/131, 86.2%). We identified 34 main features of trial designs regarding different aspects (e.g., framework, control group, randomisation). The four core categories and 34 features were merged into a double-entry table to create a new classification of trial designs for PM.

Conclusions: A variety of trial designs exists and is applied to PM. A new classification of trial designs is proposed to help readers to navigate the complex field of PM clinical trials.

Keywords

Precision medicine, Clinical trial, Study design, Scoping review

Article Summary

- This is the first review, which systematically searched for all trial designs applied to personalised medicine.
- The screening process and data extraction were performed in duplicate.
- A new classification of trial designs for personalised medicine has been proposed.
- We cannot exclude that we missed some relevant designs since we restricted the search to the last 15 years.

For peer review only

Introduction

Personalised medicine is an evolving field, which allows treating patients by providing them a specific therapy according to their individual demographic, genomic or biological characteristics (1). It was defined by the European Council Conclusion on personalised medicine as 'a medical model using characterisation of individuals' phenotypes and genotypes (e.g. molecular profiling, medical imaging, lifestyle data) for tailoring the right therapeutic strategy for the right person at the right time, and/or to determine the predisposition to disease and/or to deliver timely and targeted prevention' (2).

Many trial designs have been used to evaluate personalised treatment or interventions (3). The most common design is the enrichment design, whereby only biomarker positive patients are randomly assigned to the targeted or control arm (4). Despite its popularity, the use of enrichment designs is recommended only when the biomarker is a perfect predictor of the response in order not to deny biomarker-negative patients a treatment they would have otherwise benefited from (5). Prospective validation of the candidate biomarker is therefore strongly recommended before applying these trials designs.

Over the last years, more complex study designs have been increasingly proposed in the field of personalised medicine (4). According to the Clinical Trials Facilitation and Coordination Group, a clinical trial is considered as using a complex design "if it has separate parts that could constitute individual clinical trials and/or is characterised by extensive prospective adaptations such as planned additions of new Investigational Medicinal Products (IMPs) or new target populations" (6). These designs are particularly efficient because allow answering multiple clinical research questions within a single study (7). Examples of common complex designs are the so-called basket, umbrella, and platform trials, which are frequently applied in the field of oncology (8). Basket trials refer to designs in which patients with heterogeneous diagnoses but with similar disease mechanisms are tested using the same targeted therapy. While, umbrella trials evaluate multiple treatment options in patient groups, which present the same disease, but with different genetic mutations. Finally, platform trials allow testing multiple targeted therapies in patients with the same disease in a perpetual manner, using interim evaluations and allowing therapies to enter or leave the trial (9). However, these designs are often challenging (6) because they often require independent statistical analyses for each sub-protocol, including interim analyses driving prospective adaptation with the addition of new interventions or populations, and/or termination of sub-protocols based on futility or safety issues.

Numerous methodological challenges, covering many aspects of the study design (e.g., randomization, use of control arm, biomarker stratification, biomarker validation), are associated with trial designs applied to personalised medicine. The application of robust methodologies is especially important for clinical trials applied to personalised medicine to correctly select participants and treatments to be tested. As a starting point for the development of new recommendations on the use of trial designs applied to personalised medicine, we aimed to map the landscape of the existing study designs for clinical trials applied to this medical field.

Our specific objectives were to answer to the following five research questions:

1. What are the available designs for clinical trials applied to personalised medicine?
2. What are the examples of current applications of these approaches?
3. What are the pros and cons of the different approaches?
4. How is a personalised medicine strategy vs. non-personalised strategy evaluated?
5. What are the gaps in the current research on personalised medicine clinical trials?

This scoping review is part of the PERMIT project (PERsonalised MedIcine Trials) aimed at mapping the methods for personalised medicine research and building recommendations on robustness and reproducibility of different stages of the development programmes. Although several categorization may be proposed, the PERMIT project considers four main building blocks of the personalised medicine research pipeline: 1) design, building and management of

For peer review only

stratification and validation cohorts; 2) application of machine learning methods for patient stratification; 3) use of preclinical methods for translational development, including the use of preclinical models used to assign treatments to patient clusters; 4) evaluation of treatments in randomised clinical trials. This scoping review covers the fourth building block in this framework.

Methods

We conducted a scoping review following the methodological framework suggested by the Joanna Briggs Institute (10). The framework consists of six stages: 1) identifying the research questions, 2) identifying relevant studies, 3) selecting the studies, 4) charting the data, 5) collating, summarising and reporting results and 6) pursuing a consultation.

A study protocol was published in Zenodo before conducting the review (11). Due to the iterative nature of scoping reviews, deviations from the protocol were expected and duly reported when occurred. We used the PRISMA-ScR (Preferred Reporting Items for Systematic reviews and Meta-Analyses extension for Scoping Reviews) checklist to report our results (12).

Study identification

Relevant studies and documents were identified balancing feasibility with breadth and comprehensiveness of searches. We searched PubMed, EMBASE and the Cochrane Library (search date: April 7-8, 2020) for all reports describing a study design for clinical trials applied to personalised medicine. Online supplementary file 1 reports the search strategies applied. We did not restrict the search to any publication type. Because many systematic and narrative reviews on trial designs applied to personalised medicine have already been published over the last years, we limited our search from 2005 to April 2020. We restricted inclusion to English, French, German Italian, and Spanish languages. We searched for the grey literature on websites of existing projects about innovative clinical trials (e.g., EU-PEARL) and by consulting partners of the PERMIT project.

Eligibility criteria and deviation from the protocol

We included all reports describing a trial design applied to personalised medicine. The operational definition of personalised medicine used in the present study is reported in Box 1. Because of the extensive volume of literature related to trial designs in personalised medicine, we restricted the inclusion criteria to trial designs for Phase II, III and IV. We excluded single-arm trials, which are not part of a master protocol, non-adaptive enrichment design and N-of-1 trials. We also excluded publications such as prefaces to a special issue and speaker, symposium and panel abstracts, posters and letters to the editor due to the limited information usually provided. These exclusion criteria were not specified in the protocol, but they were agreed among the authors before starting the screening process. The research question "*What are the pros and cons of the different approaches?*" (i.e., objective 3) is not reported in the present paper, and will be subject to a specific study.

Study selection

We exported the references retrieved from the searches into the Rayyan online tool (13). Duplicates were removed automatically using the reference manager Endnote X9 (Clarivate Analytics, Philadelphia, United States) and manually by one author (CS). Eligible reports applying a particular trial design were retrieved from the search strategies and screened by reviewers. Five reviewers (II, LMSG, LSM, PJ) screened all the records and four reviewers, we conducted a pilot screening using 56 articles (2.5%), corresponding to the articles published from January 1, 2020 to search date (April 7-8, 2020), to verify whether all reviewers used the same inclusion and exclusion criteria. We retrieved full-text copies of potentially eligible reports for further assessment. Six

For peer review only

reviewers independently confirmed the eligibility: one reviewer (CS) examined all full-text copies and five reviewers (IB, II, LMSG, MMPS, SLM) assessed 20% of references each. Disagreements were solved by consensus or by involving a third expert reviewer (RP).

Charting the data

We designed a data extraction form using Google® Forms (Online supplementary file 2). General study characteristics extracted were as follows: first author name, title of article, contact detail of corresponding author, year of publication and type of publication. In addition, for each trial design referred to in the paper, we collected information on its definition, methodology, statistical considerations, advantages, disadvantages, utility, gaps and examples of actual trials, which adopted the design. A list of trial designs, which were retrieved from two previously conducted systematic reviews (14,15), was included in the data extraction form to harmonise the names used to report the same trial design. This initial list of trial designs was used as starting point to classify the identified trial designs and then modified and expanded on based on the results obtained in the present scoping review. When the trial design name reported in the paper did not match any of the trial design names included in the list, reviewers recorded the trial name verbatim.

Two reviewers (CS, FBB) piloted and refined the data extraction form using three reviews (4%). Since many narrative reviews were already published about trial designs applied to personalised medicine, the data extraction was conducted in two phases. Firstly, two reviewers (CS, FBB) independently extracted data from the identified systematic and narrative reviews. Secondly, three reviewers (CS, FBB, MC) working independently extracted data for all the remaining selected records, which were neither a systematic nor narrative review, only if they provided new information, which was not extracted in the previous phase. One reviewer (FBB) extracted data from all records and two reviewers (CS, MC) extracted 60% and 40% of articles, respectively. Differences in terminology were discussed between reviewers to ensure that the same trial designs were included in the same category. Disagreements were solved by consensus or by involving a third expert reviewer (RP).

It was not within the remit of this scoping review to assess the methodological quality of individual studies included in the analysis.

Collating, summarising and reporting results

We summarised the extracted data in tables and figures. Information on the definition, methodology, statistical considerations, advantages, disadvantages, utility and gaps of trial designs was extracted verbatim. Data on the examples of clinical trials adopting the different approaches were summarised using frequencies and percentages.

A researcher (CS) listed all study designs and identified the central feature(s) for each of them, which were grouped into feature domains. The initial list was reviewed by a senior statistician with expertise in designing clinical trials (RP). A final list was created and agreed on with members of the PERMIT steering committee and co-authors of the present study. The list of features was therefore based on the identified study designs and also the expertise of members of the PERMIT project.

New classification of trial designs in personalised medicine

Based on the identified trial designs and features, we proposed a new classification of trial designs for personalised medicine. Other attempts in classifying trial designs applied to personalised medicine have been proposed in the literature. However, they were limited to classifying the designs into categories (3,4,8) or identifying the design based on a specific feature (e.g., adaptive or non-adaptive trials) (14,15). This new classification goes a step further, proposing a new approach in classifying the trial designs considering two variables, which are core designs and design features, into a double-entry table.

For peer review only

Consultation exercise

The members of the PERMIT consortium, associated partners, and the PERMIT project Scientific Advisory Board discussed the preliminary findings of the scoping review in a 2-hour online workshop. A first version of the classification of the trial designs in personalised medicine was presented and discussed.

Patient and public involvement

The European Patients' Forum is a member of PERMIT project. Although not directly involved in the conduction of the scoping review, they received the draft review protocol for collecting comments and feedback.

Results

Study selection and general characteristics of reports

We retrieved 2350 citations from the electronic search and after removing the duplicates, 2301 remained. We excluded 1841 records based on titles and abstracts. After full-text assessment, 323 publications were excluded, and 163 met the inclusion criteria (see flow chart in Figure 1 and online supplementary file 3; the data extraction including information on the general study characteristics and definition, methodology, statistical considerations, and examples of each study design referred to in each included paper, is available on the online platform Zenodo (16)). From these 163 publications, we identified 5 systematic reviews, 66 narrative reviews, 8 original research articles, 26 methodological studies, 4 study protocols, 37 conference abstracts, 4 commentaries, 2 discussion papers, 3 reports, 1 book chapter, 1 editorial, 1 guidance document, and 5 links about trial registration (e.g., clinicaltrials.gov).

Trial designs and core designs in personalised medicine

We identified 21 trial designs, 10 sub-types, and 30 variations of trial designs applied to personalised medicine (Online supplementary file 4). Information on the definition, methodology, and statistical considerations of identified trial designs are reported on the online supplementary file 5.

We classified the trial designs into four core categories named as *Master protocols*, *Randomise-all, Biomarker-strategy,* and *Enrichment*. Building on the definitions provided by Tajik et al. (3) and Park et al. (8), we defined the four core categories as:

- *Master protocols*: trial design, which includes multiple parallel sub studies under a common infrastructure.
- *Randomise-all*: trial design where eligible patients, irrespective of their biomarker status, are randomised to either an experimental or control treatment. This category also includes those hybrid designs, which first use a *Randomise-all* design, and then only a specific biomarker defined subgroup is randomised to either an experimental or control treatment.
- *Biomarker-strategy*: trial design where eligible patients are randomised to either a marker-based treatment strategy or non-marker-based treatment strategy.
- *Enrichment*: trial design where eligibility is determined according to the biomarker status and patients are then randomised to either an experimental or control treatment. A specific biomarker defined subgroup (usually biomarker positives) is believed to benefit more from a treatment compared to the other subgroup (usually biomarker negatives).

An example of a study design for each core category, including its definition and methodology

For peer review only

used, is shown in Box 2. Overall, we identified 5 trial designs, 6 sub-types and 7 variations for *Master protocols*, and 10 trial designs, 2 sub-types and 22 variations for *Randomise-all*, 5 trial designs for *Biomarker-strategy* and 1 trial design, 2 sub-types, and 1 variation for *Enrichment.*

From the identified designs, we found 34 main features of trial designs in personalised medicine, which were clustered into 11 features domains (Table 1). The feature domains include the key design features that characterise a trial design for personalised medicine such as framework, model, control group, randomisation, biomarker assessment and adaptive aspects, and that should be carefully considered when designing a trial. A new classification of the trials designs for personalised medicine has been proposed and is reported in Table 2. The classification is presented in a double entry table, which includes the main trial features on the y-axis and core categories of the trial designs on the x-axis.

General characteristics of clinical trials in personalised medicine

We found 131 clinical trials, which used the identified designs (Online supplementary file 6). Table 3 presents the general characteristics of the identified trials.

Most trials used a basket (35/131, 26.7%), umbrella (30/131, 22.9%), platform (18/131, 13.7%) or marker stratified (15/131, 11.5%) design. The great majority of the trials were in the field of oncology (113/131, 86.3%). At the time of writing (March 2021), the recruitment status was ongoing for 48.1% (63/131) of the trials. A trial (0.8%) was not registered and seven (5.3%) presented an unknown status (meaning that the trial status has not been verified within the past two years on the clinicaltrials.gov website). Out of 131, 75 (57.3%) trials were phase II studies. For five trial designs, we did not find any examples of current applications.

Trial designs for assessing personalised versus non-personalised strategy

We identified 16 trials (16/131, 12.2%) evaluating a personalised vs. a non-personalised medicine strategy, which used nine different study designs (Online supplementary file 7).

Three trials used a biomarker design with a biomarker assessment in the control group (14,17,18). This study design consists of first testing the marker status of the entire study population and then randomises the patients either to a biomarker-based strategy arm or a non-biomarker strategy arm (14). In the GILT docetaxel trial (NCT00174629), patients with advanced non-small-cell lung cancer (NSCLC) were randomly assigned to either the control arm receiving a standard therapy of docetaxel plus cisplatin or the genotypic arm in which patients with low ERCC1 levels received docetaxel plus cisplatin and those with high levels received docetaxel plus gemcitabine. In the LIFT trial (NCT02498977), liver transplant recipients were randomised to either non-biomarker-based immunosuppression (IS) weaning or a biomarker-based IS weaning. ERCC1 gene expression was assessed in patients with NSCLC, which were then randomised to either to platinum therapy or non-platinum therapy in the ERCC1 trial (NCT00801736).

Four trials used a biomarker strategy design without biomarker assessment in the control arm (14,19–21). This design only evaluates the biomarker status in patients who are assigned to the biomarker-based strategy (14). Patients were randomised to either the NT-pro-BNP-guided therapy or usual care in the GUIDE-IT trial (NCT01685840) and either an algorithm driven individualized hemodynamic goal-directed therapy or standard care in the iPEGASUS trial (NCT03021525). Patients with mild head injury were randomly assigned to computed tomography or observation in the hospital in the OCTOPUS trial (ISRCTN81464462) and children with a doctor's diagnosis of asthma were randomised to a personalised medicine genotype-guided treatment arm or to usual care, nongenotype-guided, control arm in the PUFFIN trial (NCT03654508).

A modified strategy design, which differs from the previous strategy designs in including multiple targeted molecular profiles (22), was used in two trials (22–25). Patients with refractory cancer in

For peer review only

the SHIVA trial (NCT01771458) were randomised to receive a molecularly targeted therapy based on metastasis molecular profiling or a conventional chemotherapy. In the NCI-MPACT trial (NCT01827384), patients with an actionable mutation of interest (aMOI) were assigned to a targeted therapy based on mutation status or a therapy, chosen from the four regimes, not targeting the aMOI. We found that these two trials were also labelled as basket trials (26–28) as well as platform trial in the case of the SHIVA trial (29).

One trial used an adaptive strategy design for biomarkers with measurement error (25). This design is used when a second cheaper biomarker exists and may be concordant with a more expensive one, which is considered the gold standard. This design was used with some modifications in the OPTIMA trial (ISRCTN42400492). Oestrogen receptor-positive, HER-2 negative breast cancer patients were randomised to be either in the control arm receiving the standard care (i.e., chemotherapy and endocrine therapy) or in the treatment arm receiving the marker-guided therapy (i.e., endocrine therapy). Patients in the treatment arm, which obtained a high-risk test, also received chemotherapy.

The Siyaphambili Study (NCT03500172) used a sequential multiple assignment randomised (SMART) design to compare an individualised intervention (i.e., peer-led, individualised case management) or non-individualised intervention (i.e., nurse-led mobile decentralised treatment programs) to standard care (i.e., South African standard of care) or combination of both interventions in women living with HIV (30). The SMART design allows comparing adaptive treatment strategies (ATSs), which consist of a series of tailored therapies during the course of a treatment (31).

ProBio (NCT03903835) used an outcome-randomization adaptive design to investigate whether a treatment based on molecular biomarker signature is more effective than standard care in men with metastatic castrate-resistant prostate cancer.

Finally, we found four trials, which evaluated a personalised versus a non-personalised strategy using a master protocol design (32–35). IMPACT II (NCT02152254) used a basket design and UPSTREAM (NCT03088059), SAFIR02_Breast (NCT02299999) and SAFIR02_Lung (NCT02117167) an umbrella design.

Gaps in the current research on clinical trials applied to personalised medicine

The results of this scoping review also allowed us to identify some gaps in the current research on clinical trials in personalised medicine. We identified three main gaps, which concern 1) the terminology used in labelling trial designs applied to personalised medicine, 2) the applications of complex innovative trial designs to fields outside of oncology and 3) the implementation of trials for evaluating personalised medicine strategy vs. non-personalised strategy.

We found that trial designs are often labelled in different ways or mislabelled, despite this gap having been identified previously (3,4,14,15). An example is the *Marker stratified design*, which was named using 18 different labels (Online supplementary file 4). We also found that a study design adopted in a clinical trial was defined differently across the literature. For instance, the I-SPY 2 trial (NCT01042379) has been labelled as outcome-based adaptive randomisation (15), platform (36) or umbrella design (37). The I-SPY 2 is an on-going platform trial, which studies multiple therapies in the context of breast cancer in a perpetual manner with arms being added or dropped based on current knowledge and collected data. Moreover, the study design adopted in the I-SPY 2 trial includes Bayesian adaptation algorithms in order to make decisions on estimated posterior probabilities, which are calculated at frequent interim-analysis points and response-adaptive randomisation (9). According to the new proposed classification, I-SPY 2 trial would be classified as *Master protocol* because it includes multiple sub studies under the same framework, with common/shared control group, early stopping, interim analysis and outcome-based adaptive randomisation as main design features.

For peer review only

Moreover, another gap in the current research on personalised medicine is the lack of application of novel complex study designs to fields outside of oncology. We found that 94% (80/85) of the clinical trials which used a master protocol design were in the field of oncology.

Finally, a strong need exists for clinical trials evaluating the effectiveness of a personalised medicine strategy vs. non-personalised strategy. This constitutes the third gap that we identified by mapping the evidence on clinical trials applied to personalised medicine. We found only 16 trials using nine different trial designs, which compared the two strategies.

Discussion

The present study provides a broad overview and proposes a new classification of the trial designs applied to personalised medicine.

The scoping review approach was considered to be the most suitable to respond to the extensive scope of the field. Compared to systematic reviews that aim to answer specific questions, scoping reviews are used to present a broad overview of the evidence pertaining to a topic and they are useful to examine areas that are emerging, to clarify key concepts and identify gaps (38,39).

To our knowledge, this is the first study, which systematically reviews all trial designs, including complex innovative designs (i.e., basket, umbrella and platform), applied to personalised medicine. Other systematic reviews have been performed on specific trial designs such as biomarker-guided adaptive trial designs (15), biomarker-guided non-adaptive trials designs (14) and master protocols (8) or without considering master protocols in the search strategy (3).

We identified 21 trial designs, 10 sub-types, and 30 variations of trial designs applied to personalised medicine, which have been classified into four core categories: *Master protocols*, *Randomise-all*, *Biomarker strategy* and *Enrichment*. *Randomise-all* encompasses the largest number of trial designs (i.e., 10 trial designs, 2 sub-types and 22 variations) and *Master protocols* includes those study designs which are more frequently used in clinical trials (86/131, 65.6%). A variation of the enrichment design called *Multistage adaptive biomarker-directed targeted (MAT) design* (40), which combines some features of both targeted and adaptive designs, was included in the present review because does not present the standard characteristics of a classical enrichment design but not in our classification. In the MAT design, biomarker-positive patients are first randomised to either treatment or standard of care and interim analyses are then conducted to monitor if the primary study objectives can be achieved.

From the different approaches applied to personalised medicine, we identified 34 central features, which were combined with the four core categories in a double entry table. The proposed table constitutes a novel manner to classify trial designs applied to personalised medicine, considering its corresponding core category and main features (e.g., PM specific or generic adaptive aspects) The classification only includes features, which are strictly related to trial designs. Methods for stratification and validation of clusters in a clinical trial (e.g., data-driven subgroup identification) were considered not eligible and therefore were not included. In particular, those methods were identified and described in another recent scoping review (2021) (41). Due to the variety and diversity of trial designs currently available, this classification provides a clearer and more accessible picture of the different trial designs available in personalised medicine, helping the readers to navigate this complex field. In addition, it could be particularly helpful for researchers as a first step for understanding the different methodological approaches available for their trials.

Also, it permits to consider all the relevant features associated with a trial design reducing confusion in reporting and labelling. We believe that this classification is more accurate and appropriate for describing a trial design applied to personalised medicine in its complexity. Moreover, it could help researchers and clinicians in using a harmonised terminology for labelling a trial.

For peer review only

Based on the results obtained, we identified three main gaps in the current research on clinical trials applied to personalised medicine. We found that more research is needed to evaluate the efficiency of personalised medicine approach vs. non-personalised standard of care. A few clinical trials (16/131, 12.2%), using nine different study designs, were found evaluating these different strategies. In addition, these trials would be particularly relevant for Health Technologies Assessment (HTA) bodies to evaluate the incremental benefit of personalised medicine over that of non-personalised approaches, from both a clinical and economical perspective, in those situations in which a non-personalised strategy is considered standard practice. We also need more research to apply trial designs to fields outside of oncology. This last result was consistent with what was found in a recent systematic review of master protocols (8). The review showed that the great majority of basket, umbrella and platform studies (76/83, 91.6%) were conducted in the field of oncology. In particular, no umbrella trials were found outside of oncology. Finally, in line with two previous systematic reviews (3,4), we found that a harmonised terminology was required because it would permit increase clarity among the variety of trial designs applied to personalised medicine.

Furthermore, current applications of the identified trial designs, together with the input of some experts in the field, helped us to identify four typologies of personalised medicine. For *targeted or precision medicine*, a targeted treatment, which is specific for one disease, is identified and used to treat patients with heterogeneous diagnoses but similar disease mechanisms (e.g., basket trials). *Stratified medicine* includes trials in which patients are stratified in different clusters based on the collection of data characterised by the genotype or phenotype of the individuals (e.g., adaptive signature trials). The treatment is tailored to each patient in the *individualised medicine* (e.g., trials using pharmacokinetic models). Finally, in *individualised medicine with a dynamic regime*, the treatment tailored to each patient is adjusted over time based on the patient's response (e.g., SMART trials).

The new classification and the four typologies of personalised medicine clinical trials provide the basis for the future recommendations on the use of trial designs applied to personalised medicine and on trials assessing personalised versus non-personalised medicine strategy. These recommendations are strongly needed to conduct new studies within the context of personalised medicine and, consequently, have new direct high-quality evidence in the evaluation of co-dependent personalised medicine technologies (42).

The present study has strengths but also limitations. This is the first scoping review, which presents an overview of all trial designs applied to personalised medicine. We followed a systematic approach to map the evidence and described the process using the PRISMA-ScR guideline. However, we restricted the search strategy to the last 15 years proving a comprehensive overview rather than an exhaustive list of trial designs used in personalised medicine. In addition, by excluding single-arm trials, which are not part of a master protocol, non-adaptive enrichment design and N-of-1 trials, we might misrepresent certain study designs used for personalised medicine. Moreover, although we conducted a pilot screening for verifying the use of the same inclusion and exclusion criteria among reviewers, we cannot exclude that we did not identify some relevant publications. The information on the definition, methodology, statistical considerations, advantages, disadvantages, utility and gaps of trial designs was extracted verbatim from the included records. However, the selection of this information could be affected by the perception of the three reviewers who conducted the data extraction. Also, even if we built on existing reviews (14,15) and carefully developed a comprehensive classification, all attempts at categorisation are reductive in nature, and different classification schemes could be proposed. We believe that all classifications are based on decisions, some of which are inevitably arbitrary. Nonetheless, our proposal allows separating between core design features that characterise the main objective of the trial and the patient flow, important aspects of the trial, and more accessory design features. It may form the basis of the evaluation of which design, and which features would be best suited for a given situation. For instance, HTA representatives could use our classification as a first step to better understand the design choice taken by the researchers and successively evaluate it.

The information extracted on the pros and cons of each approach (i.e., objective 3) will be subject of further analysis and will be publish in a separate study due to considerable volume of

For peer review only

information collected. We will also explore the pros and cons of each approach in more detail, together with experts from academia and regulatory agencies, when preparing the recommendations on the use of trial designs applied to personalised medicine.

Conclusions

The findings of this scoping review show that several existing trial designs are applied to personalised medicine, which can be grouped into four core categories. A new classification has been proposed that allows describing trial designs taking into account their corresponding core category and main features. It can be used by readers to explore and better understand the complex field of personalised medicine clinical trials.

For peer review only

Ethics approval

This study was based entirely on a scoping review of relevant published literature and did not require an ethics approval.

Acknowledgments

The authors thank Vanna Pistotti for her assistance with search strategy development and conduction, Ines Bouajila for collaborating to the screening process and Frank Bretz, Frank Petavy and Stephen Senn for their excellent inputs and feedback.

List of Figures

Figure 1: Study selection flow diagram

References

1. Heckman-Stoddard BM, Smith JJ. Precision Medicine Clinical Trials: Defining New Treatment Strategies. Semin Oncol Nurs. 2014 May;30(2):109–16.

2. Council of the European Union. Notices from European Union Institutions, bodies, offices and agencies. OJEU. 2015;C 421/2-C 421/5.

3. Tajik P, Zwinderman AH, Mol BW, Bossuyt PM. Trial Designs for Personalizing Cancer Care: A Systematic Review and Classification. Clin Cancer Res. 2013 Sep 1;19(17):4578–88.

4. Janiaud P, Serghiou S, Ioannidis JPA. New clinical trial designs in the era of precision medicine: An overview of definitions, strengths, weaknesses, and current use in oncology. Cancer Treat Rev. 2019 Feb;73:20–30.

5. Vivot A, Li J, Zeitoun J-D, Mourah S, Crequit P, Ravaud P, et al. Pharmacogenomic biomarkers as inclusion criteria in clinical trials of oncology-targeted drugs: a mapping of ClinicalTrials.gov. Genet Med. 2016 Aug;18(8):796–805.

6. Clinical Trials Facilitation and Coordination Group. Recommendation Paper on the Initiation and Conduct of Complex Clincial Trials. 2019.

7. Blagden SP, Billingham L, Brown LC, Buckland SW, Cooper AM, Ellis S, et al. Effective delivery of Complex Innovative Design (CID) cancer trials—A consensus statement. Br J Cancer. 2020 Feb 18;122(4):473–82.

8. Park JJH, Siden E, Zoratti MJ, Dron L, Harari O, Singer J, et al. Systematic review of basket trials, umbrella trials, and platform trials: a landscape analysis of master protocols. Trials. 2019 Dec;20(1):572.

9. Woodcock J, LaVange LM. Master Protocols to Study Multiple Therapies, Multiple Diseases, or Both. Drazen JM, Harrington DP, McMurray JJV, Ware JH, Woodcock J, editors. N Engl J Med. 2017 Jul 6;377(1):62–70.

10. Peters MDJ, Godfrey CM, Khalil H, McInerney P, Parker D, Soares CB. Guidance for conducting systematic scoping reviews. Int J Evid Based Healthc. 2015 Sep;13(3):141–6.

11. Banzi R, Gerardi C, Fratelli M, Garcia P, Torres T, Haro Abad JM, et al. Methodological approaches for personalised medicine: protocol for a series of scoping reviews. 2020 Apr; Available from: https://zenodo.org/record/3770937 doi: 10.5281/zenodo.3770937

For peer review only

12. Tricco AC, Lillie E, Zarin W, O'Brien KK, Colquhoun H, Levac D, et al. PRISMA Extension for Scoping Reviews (PRISMA-ScR): Checklist and Explanation. Ann Intern Med. 2018 Oct 2;169(7):467.

13. Ouzzani M, Hammady H, Fedorowicz Z, Elmagarmid A. Rayyan—a web and mobile app for systematic reviews. Syst Rev. 2016 Dec;5(1):210.

14. Antoniou M, Kolamunnage-Dona R, Jorgensen A. Biomarker-Guided Non-Adaptive Trial Designs in Phase II and Phase III: A Methodological Review. J Pers Med. 2017 Jan 25;7(1):1.

15. Antoniou M, Jorgensen AL, Kolamunnage-Dona R. Biomarker-Guided Adaptive Trial Designs in Phase II and Phase III: A Methodological Review. PLOS ONE. 2016 Feb 24;11(2):e0149803.

16. [dataset] Superchi C, Brion Bouvier F, Gerardi C, Carmona M, San Miguel L, Sánchez Gómez LM, et al. Data from: scoping review of study designs for clinical trials applied to personalised medicine. Zenodo. 2022 Jan 19; Available from: https://zenodo.org/record/5874552#.Ye7wJmDEVQM doi:10.5281/zenodo.5874552

17. Clinicaltrials.gov. Liver Immunosuppression Free Trial (LIFT) [Internet]. Available from: https://clinicaltrials.gov/ct2/show/NCT02498977

18. Freidlin B, McShane LM, Korn EL. Randomized Clinical Trials With Biomarkers: Design Issues. JNCI J Natl Cancer Inst. 2010 Feb 3;102(3):152–60.

19. Ahmad T, O'Connor CM. Therapeutic Implications of Biomarkers in Chronic Heart Failure. Clin Pharmacol Ther. 2013 Oct;94(4):468–79.

20. Funcke S, Saugel B, Koch C, Schulte D, Zajonz T, Sander M, et al. Individualized, perioperative, hemodynamic goal-directed therapy in major abdominal surgery (iPEGASUS trial): study protocol for a randomized controlled trial. Trials. 2018 Dec;19(1):273.

21. Vijverberg SJ, Pijnenburg MW, Hövels AM, Koppelman GH, Maitland-van der Zee A-H. The need for precision medicine clinical trials in childhood asthma: rationale and design of the PUFFIN trial. Pharmacogenomics. 2017 Mar;18(4):393–401.

22. Renfro LA, An M-W, Mandrekar SJ. Precision oncology: A new era of cancer clinical trials. Cancer Lett. 2017 Feb;387:121–6.

23. Dienstmann R, Rodon J, Tabernero J. Optimal design of trials to demonstrate the utility of genomically-guided therapy: Putting Precision Cancer Medicine to the test. Mol Oncol. 2015 May;9(5):940–50.

24. Paoletti X, Asselain B, Kamal M, Servant N, Huppé P, Bieche I, et al. Design and statistical principles of the SHIVA trial. Chin Clin Oncol. 2015;4(3):1–10.

25. Renfro LA, Mallick H, An M-W, Sargent DJ, Mandrekar SJ. Clinical trial designs incorporating predictive biomarkers. Cancer Treat Rev. 2016 Feb;43:74–82.

26. Coyne GO, Takebe N, Chen AP. Defining precision: The precision medicine initiative trials NCI-MPACT and NCI-MATCH. Curr Probl Cancer. 2017 May;41(3):182–93.

27. Gómez-López G, Dopazo J, Cigudosa JC, Valencia A, Al-Shahrour F. Precision medicine needs pioneering clinical bioinformaticians. Brief Bioinform. 2019 May 21;20(3):752–66.

28. Simon R. New designs for basket clinical trials in oncology. J Biopharm Stat. 2018 Mar 4;28(2):245–55.

29. Leonetti A, Boyd L, Giuliani J, Giovannetti E, Garajová I. Light and shadow on innovative

For peer review only

clinical trial designs: reflections from the EORTC-PAMM course on 'preclinical and early-phase clinical pharmacology.' Expert Rev Clin Pharmacol. 2019 Nov 2;12(11):1033–6.

30. Clinicaltrials.gov. HIV Treatment Retention Interventions for Women Living With HIV (Siyaphambili Study) [Internet]. Available from: https://clinicaltrials.gov/ct2/show/NCT03500172

31. Kidwell KM, Wahed AS. Weighted log-rank statistic to compare shared-path adaptive treatment strategies. Biostatistics. 2013 Apr 1;14(2):299–312.

32. Lindberg J, De Laere B, Crippa A, Eklund M, Grönberg H. ProBio: An outcome-adaptive, multi-arm, open-label, multiple assignment randomised controlled biomarker-driven trial in patients with metastatic castration-resistant prostate cancer. In Ann Oncol; 2019. p. v354.

33. Galot R, Le Tourneau C, Saada-Bouzid E, Daste A, Even C, Debruyne PR, et al. A phase II study of monalizumab in patients with recurrent/metastatic (RM) squamous cell carcinoma of the head and neck (SCCHN): Results of the I1 cohort of the EORTC-HNCG-1559 trial (UPSTREAM). Ann Oncol. 2019 Oct;30:v449–50.

34. Hernandez-Martinez J-M, Sánchez-Reyes R, De la Garza-Salazar JG, Arrieta O. Onco-omics Approaches and Applications in Clinical Trials for Cancer Patients. In: Translational Research and Onco-Omics Applications in the Era of Cancer Personal Genomics. Springer; 2019. p. 79–90.

35. Park JJH, Hsu G, Siden EG, Thorlund K, Mills EJ. An overview of precision oncology basket and umbrella trials for clinicians. CA Cancer J Clin. 2020 Mar;70(2):125–37.

36. Talisa VB, Yende S, Seymour CW, Angus DC. Arguing for Adaptive Clinical Trials in Sepsis. Front Immunol. 2018 Jun 28;9:1502.

37. Tao JJ, Schram AM, Hyman DM. Basket Studies: Redefining Clinical Trials in the Era of Genome-Driven Oncology. Annu Rev Med. 2018 Jan 29;69(1):319–31.

38. Pham MT, Rajić A, Greig JD, Sargeant JM, Papadopoulos A, McEwen SA. A scoping review of scoping reviews: advancing the approach and enhancing the consistency. Res Synth Methods. 2014 Dec;5(4):371–85.

39. Munn Z, Peters MDJ, Stern C, Tufanaru C, McArthur A, Aromataris E. Systematic review or scoping review? Guidance for authors when choosing between a systematic or scoping review approach. BMC Med Res Methodol. 2018 Dec;18(1):143.

40. Gao Z, Roy A, Tan M. Multistage adaptive biomarker-directed targeted design for randomized clinical trials. Contemp Clin Trials. 2015 May;42:119–31.

41. Glaab E, Rauschenberger A, Banzi R, Gerardi C, Garcia P, Demotes J. Biomarker discovery studies for patient stratification using machine learning analysis of omics data: a scoping review. BMJ Open. 2021;11(e053674):16.

42. European Network for Health Technology Assessment. Personalised medicine and co-dependent technologies, with a special focus on issues of study design [Internet]. 2015. Available from: https://eunethta.eu/wp-content/uploads/2018/03/Personalized_Medicine_2016-03-07_reflection_paper_pm_2nd_draft.pdf

43. Simon R. Critical Review of Umbrella, Basket, and Platform Designs for Oncology Clinical Trials: Review of umbrella, basket, and platform trial designs. Clin Pharmacol Ther. 2017 Dec;102(6):934–41.

44. Parke T, Pericàs JM, Posch M, Heimbach N, Spiertz C, Mesenbrink P, et al. D2.1. Report on Terminology, References and Scenarios for Platform Trials and Master Protocols [Internet].

For peer review only

2020. Available from: https://eu-pearl.eu/wp-content/uploads/2020/06/EU-PEARL_D2.1_Report-on-Terminology-and-Scenarios-for-Platform-Trials-and-Masterprotocols.pdf

45. Hong F, Simon R. Run-In Phase III Trial Design With Pharmacodynamics Predictive Biomarkers. J Natl Cancer Inst. 2013;105(21):6.

46. Sheiner LB, Rosenberg B, Melmon KL. Modelling of individual pharmacokinetics for computer-aided drug dosage. Comput Biomed Res. 1972 Oct;5(5):411-59. doi: 10.1016/0010-4809(72)90051-1. PMID: 4634367.

For peer review only

Authors' contributions

Study conception and design: CG, CS, II, JDM, LSM, LMSG, PG, RB and RP

Methodology: CG, CS, RB

Data collection and analysis: CS, FBB, MCR, II, LSM and LMSG.

Trial design classification: CS and RP

Original draft preparation: CS

Review and editing: CG, II, LSM, LMSG, MCR, PG, RB and RP.

All authors read and approved the final version of the manuscript.

The members of the PERMIT group were involved in the preparation or revision of the joint protocol of the four scoping reviews of the PERMIT series, attended the joint workshop (consultation exercise) or contributed to one of the other scoping reviews of the PERMIT series.

PG and JDM coordinate the PERMIT project. JDM obtained funding.

Collaborators

PERMIT Group: Antonio L Andreu, Florence Bietrix, Maria del Mar Polo-de Santos,, Maddalena Fratelli, Vibeke Fosse, Enrico Glaab, Rainer Girgenrath, Alexander Grundmann, Josep Maria Haro, Frank Hulstaert, Pascale Jonckheer, Setefilla Luengo Matos, Emmet McCormack, Anna Monistrol Mula, Albert Sanchez Niubo, Emanuela Oldoni, Teresa Torres.

Funding statement

This project has received funding from the European Union's Horizon 2020 research and innovation programme under grant agreement No. 874825.

Competing interests statement

None declared

Patient consent

Not required

Data sharing statement

Data are available in a public, open access repository. The dataset supporting the conclusions of the research reported in this paper is available in the Zenodo repository in the PERMIT community (https://zenodo.org/communities/permit-project/?page=1&size=20). The dataset can be accessed via Zenodo at https://zenodo.org/record/5874552#.Ye7wJmDEVQM with doi:10.5281/zenodo.5874552

For peer review only

Box. 1 Personalised medicine definition

What is Personalised Medicine?

According to the European Council Conclusion on personalised medicine for patients personalised medicine is 'a medical model using characterisation of individuals' phenotypes and genotypes (e.g. molecular profiling, medical imaging, lifestyle data) for tailoring the right therapeutic strategy for the right person at the right time, and/or to determine the predisposition to disease and/or to deliver timely and targeted prevention (2).

In the context of the Permit project, we applied the following common operational definition of personalised medicine research: a set of comprehensive methods, (methodological, statistical, validation or technologies) to be applied in the different phases of the development of a personalised approach to treatment, diagnosis, prognosis, or risk prediction. Ideally, robust and reproducible methods should cover all the steps between the generation of the hypothesis (e.g., a given stratum of patients could better respond to a treatment), its validation and pre-clinical development, and up to the definition of its value in a clinical setting (11).

For peer review only

Box 2. Examples of core categories

Core category	Study design example	Study design definition	Study design methodology
**Master protocols**	Platform	"A platform trial is a single histology randomized phase II clinical trial involving multiple biomarkers and multiple drugs. Rather than assuming that we know which drug is appropriate for which biomarker stratum, randomization among drugs is used in the platform trial." (43)	"Initially the treatments are randomized with equal weights to the patients of a stratum. As data accumulates, the randomization weights change to favour assignment of drugs with higher within-stratum response rates. The endpoint used must be observed early enough to enable adaption of randomization weights." (43)
**Randomise-all**	Biomarker-positive and overall strategies with fall-back analysis	"It evaluates both the treatment effect in the overall study population and in the biomarker-positive subgroup sequentially." (14)	"In the fall-back design, we first test the overall population using the reduced significance level α_1 and if the test is significant, we consider that the novel treatment is effective in the overall population; however, if the result is not significant then we test the treatment effect in the biomarker-positive subgroup using the level of significance $\alpha_2 = \alpha - \alpha_1$,where α is the overall significance level (Type I error rate). The significance levels α can be considered as one-sided or two-sided significance levels."(14)
**Biomarker strategy**	Biomarker-strategy design with treatment randomization in the control arm	"The biomarker-strategy design with treatment randomization in the control treatment is able to inform us about whether the biomarker-based strategy is better than not only the standard treatment but also better than the experimental treatment in the overall population." (14)	"Patients are first randomly assigned to either the biomarker-based strategy arm or to the non-biomarker-based strategy arm. Next, patients who are allocated to the non-biomarker-based strategy are again randomized either to the experimental treatment arm or to the standard treatment arm irrespective of their biomarker status. Patients who are allocated to the biomarker-based strategy and who are biomarker-positive are given the experimental treatment and patients who are biomarker-negative are given the control treatment." (14)
**Enrichment**	Adaptive threshold sample-enrichment design	"It is a two-stage design in a Phase III setting […] to adaptively modify accrual in order to broaden the targeted patient population." (15)	"At the interim analysis stage, the treatment effect of a sample of patients (n_1) from the biomarker-positive subset is estimated. If an improvement is seen in the experimental treatment arm which is greater than a pre-specified threshold value (i.e. the estimated treatment difference between the novel treatment arm and the control treatment arm for this subpopulation is greater than a threshold value c divided by the square root of the aforementioned sample size n_1) the trial continues with accrual of patients from the entire biomarker-positive subgroup and additional patients are also accrued from the biomarker-negative subpopulation; otherwise the trial is stopped for futility. At the end of the trial, the treatment effect is estimated for all subpopulations. Researchers should choose the sample size $n_1$so that a persuasive result can be reached when the first stage of the trial is completed." (15)

For peer review only

Table 1. Main features of trial designs applied to personalised medicine

Feature domains	Features
Inference framework	Bayesian
	Frequentist
Model[1]	Disease progression[1]
	Longitudinal[1]
	Hierarchical
Control group	Common/Shared[2]
	Contemporaneous[3]
	Historical[4]
Randomisation	With treatment randomisation in both biomarker-positive and biomarker-negative subgroups
	Without treatment randomisation in the biomarker-negative subgroup[5]
	Only for patients with discordant clinical and genomic risk evaluation[6]
Randomisation in the non-biomarker based strategy arm	With treatment randomisation
	Without treatment randomisation[7]
	Reverse biomarker strategy[8]
Subgroup specific	Sequential subgroup specific[9]
	Parallel subgroup specific[10]
Biomarker positive and overall strategies[11]	With sequential assessment
	With parallel assessment
	With fall-back analysis[12]
	Marker sequential test[13]
Biomarker assessment	With biomarker assessment in the entire population
	Without biomarker assessment in the control arm
Personalised medicine (PM) specific adaptive aspects[14]	Adaptive enrichment
	Adaptive signature
	Threshold determination[15]
Generic adaptive aspects	Adding a new arm
	Early stopping[16]
	Interim analysis[17]
	Outcome-based adaptive randomisation
	Sample size reassessment
	Seamless
Treatment tailoring aspects	Pharmacodynamic biomarker assessment after run-in phase period[18]
	Dynamic treatment regime[19]
	PK/PD modeling[20]

[1]Model used for analysis. A disease progression model takes into account the patient disease state and other patient baseline characteristics for charactering patient clinical outcome(s) (44). Longitudinal model permits including in the analysis the partial information of patients who have not yet reached their final outcome at an interim analysis (44).

[2] A common/shared control group can be used in a trial design in which multiple treatments are being tested, instead of each treatment having its own control arm.

[3] If patients in the common/shared control group receive a 'Standard of care' that may change over time or the profile of the patients enrolled on the trial may change over time, a trial design can use a contemporaneous control group meaning that the comparison of treatment's effects may be restricted to those patients who were enrolled/randomised in the same period as those patients who were allocated to the treatment.

[4] If a comparison group is not available in the existing trial or sub-study or at the same time but in a different setting, a trial design can use a historical control consisted of a group of individuals treated in the past.

[5] Patients in the biomarker-negative subgroup receive the control treatment.

For peer review only

[6] Only patients with discordant results (i.e., either high clinical risk an low genomic risk or low clinical risk and high genomic risk) are randomly assigned to either the control or intervention arm.

[7] Patients, which are randomly assigned to the non-biomarker-based strategy arm, receive the control treatment.

[8] Patients which are randomly assigned to reverse-based strategy receive the control treatment if they are biomarker-positive and the experimental treatment if they are biomarker-negative.

[9] Study designs testing the treatment effect first in the biomarker-positive subpopulation and if the result is positive in the biomarker-negative subgroup.

[10] Study designs testing the treatment effect in both biomarker-positive and biomarker negative subgroups simultaneously.

[11] Study designs testing the treatment effect in the entire study population and in the biomarker-positive subgroup separately.

[12] Study designs testing the treatment effect in the overall population and in the biomarker-positive subgroup sequentially.

[13] Study designs testing the treatment effect not only in the biomarker-positive and biomarker-negative subgroups but also in the entire population sequentially.

[14] PM-specific adaptive aspects could be used to stratify the patients to the treatment. Generic adaptive aspects could be considered when planning a PM trial, but they could be also found in fields outside PM.

[15] A threshold is used to divide the population into 'biomarker positive' and 'biomarker negative'.

[16] A trial arm or clinical trial is stopped early due to pre-specified rules related to treatment efficacy and safety risk.

[17] Interim analyses are pre-planned analyses, which use accumulating data in order to make an early decision or adaptation.

[18] All patients receive the new treatment for a run-in period and then are classified as either biomarker positive or negative using a pharmacodynamics biomarker (45).

[19] A dynamic treatment regime consists of a sequence of individually tailored therapies during the course of a treatment.

[20] Models to suggest optimal dosage regimes of drugs for individual patients (46).

For peer review only

Table 2. Trial designs classification

Design features \ Core designs		Biomarker strategy	Enrichment	Master protocols	Randomise-all
**Framework**	Bayesian				
	Frequentist				
**Model**	Disease progression				
	Longitudinal				
	Hierarchical				
**Control group**	Common/shared				
	Contemporaneous				
	Historical				
**Randomisation**	With treatment randomisation in both biomarker-positive and biomarker-negative subgroups				
	Without treatment randomisation in the biomarker-negative subgroup				
	Only for patients with discordant clinical and genomic risk evaluation				
**Randomisation in the non-biomarker based strategy arm**	With treatment randomisation				
	Without treatment randomisation				
	Reverse biomarker strategy				
**Subgroup specific**	Sequential subgroup specific				
	Parallel subgroup specific				

For peer review only

**Biomarker positive and overall strategies**	With sequential assessment				
	With parallel assessment				
	With fall-back analysis				
	Marker sequential test				
**Biomarker assessment**	With biomarker assessment in the entire population				
	Without biomarker assessment in the control arm				
**Personalised medicine (PM) specific adaptive aspects[14]**	Adaptive enrichment				
	Adaptive signature				
	Threshold determination				
**Generic adaptive aspects**	Adding a new arm				
	Early stopping				
	Interim analysis				
	Outcome-based adaptive randomisation				
	Sample size reassessment				
	Seamless				
**Treatment tailoring aspects**	Pharmacodynamic biomarker assessment after run-in phase period				
	Dynamic treatment regime				
	PK/PD modeling				

For peer review only

Table 3. General characteristics of clinical trials in personalised medicine

Trial design	Clinical trial[1]	Recruitment status of clinical trial as for March 2021				Disease area		Phases					
		Ongoing	Completed	nf[2]	Unknown[3]	Cancer	No cancer	II	II/III	III	IV	n/a[4]	nf[2]
	n=131 (%)	n=63 (%)	n=60 (%)	n=1 (%)	n=7 (%)	n=113 (%)	n=18 (%)	n=75 (%)	n=13 (%)	n=28 (%)	n=2 (%)	n=12 (%)	n=1 (%)
**Adaptive biomarker design**	0 (0)	0 (0)	0 (0)	0 (0)	0 (0)	0 (0)	0 (0)	0 (0)	0 (0)	0 (0)	0 (0)	0 (0)	0 (0)
**Adaptive parallel Simon two-stage design**	1 (0.8)	0 (0)	1 (1.7)	0 (0)	0 (0)	1 (0.9)	0 (0)	1 (1.3)	0 (0)	0 (0)	0 (0)	0 (0)	0 (0)
**Adaptive patient enrichment design**	4 (3.1)	0 (0)	4 (6.7)	0 (0)	0 (0)	0 (0)	4 (22.2)	0 (0)	0 (0)	4 (14.3)	0 (0)	0 (0)	0 (0)
**Adaptive signature design**	0 (0)	0 (0)	0 (0)	0 (0)	0 (0)	0 (0)	0 (0)	0 (0)	0 (0)	0 (0)	0 (0)	0 (0)	0 (0)
**Adaptive strategy for biomarker with measurement error**	1 (0.8)	1 (1.6)	0 (0)	0 (0)	0 (0)	1 (0.9)	0 (0)	0 (0)	0 (0)	0 (0)	0 (0)	1 (8.3)	0 (0)
**Adaptive stratified design**	0 (0)	0 (0)	0 (0)	0 (0)	0 (0)	0 (0)	0 (0)	0 (0)	0 (0)	0 (0)	0 (0)	0 (0)	0 (0)
**Adaptive threshold sample-enrichment design**	0 (0)	0 (0)	0 (0)	0 (0)	0 (0)	0 (0)	0 (0)	0 (0)	0 (0)	0 (0)	0 (0)	0 (0)	0 (0)
**Basket**	35 (26.7)	19 (30.2)	13 (21.7)	0 (0)	3 (42.9)	34 (30.1)	1 (5.6)	32 (42.1)	0 (0)	2 (7.1)	0 (0)	1 (8.3)	0 (0)
**Basket of basket design**	1 (0.8)	1 (1.6)	0 (0)	0 (0)	0 (0)	1 (0.9)	0 (0)	1 (1.3)	0 (0)	0 (0)	0 (0)	0 (0)	0 (0)
**Biomarker strategy design with biomarker assessment in the control arm**	3 (2.3)	0 (0)	3 (5.0)	0 (0)	0 (0)	2 (1.8)	1 (5.6)	0 (0)	0 (0)	2 (7.1)	1 (50.0)	0 (0)	0 (0)
**Biomarker strategy design with treatment randomisation in the control arm**	0 (0)	0 (0)	0 (0)	0 (0)	0 (0)	0 (0)	0 (0)	0 (0)	0 (0)	0 (0)	0 (0)	0 (0)	0 (0)
**Biomarker strategy design without biomarker assessment in the control arm**	4 (3.1)	2 (3.2)	2 (3.3)	0 (0)	0 (0)	0 (0)	4 (22.2)	0 (0)	0 (0)	0 (0)	0 (0)	4 (33.3)	0 (0)
**Hybrid design**	1 (0.8)	0 (0)	1 (1.7)	0 (0)	0 (0)	1 (0.9)	0 (0)	0 (0)	0 (0)	1 (3.6)	0 (0)	0 (0)	0 (0)
**Marker stratified design**	15 (11.5)	0 (0)	14 (23.3)	1 (100)	0 (0)	15 (13.3)	0 (0)	0 (0)	0 (0)	14 (50.0)	0 (0)	0 (0)	1 (100.0)
**Modified biomarker strategy design**	3 (2.3)	0 (0)	2 (3.3)	0 (0)	1 (14.3)	3 (2.7)	0 (0)	2 (2.7)	0 (0)	1 (3.6)	0 (0)	0 (0)	0 (0)

For peer review only

**Multi-arm multi-stage design**	7 (5.3)	3 (4.8)	3 (5.0)	0 (0)	1 (14.3)	5 (4.4)	2 (11.1)	4 (5.3)	2 (15.4)	1 (3.6)	0 (0)	0 (0)	0 (0)
**Outcome-based adaptive randomisation design**	4 (3.1)	2 (3.2)	2 (3.3)	0 (0)	0 (0)	3 (2.7)	1 (5.6)	2 (2.7)	1 (7.7)	1 (3.6)	0 (0)	0 (0)	0 (0)
**Platform**	18 (13.7)	13 (20.6)	4 (6.7)	0 (0)	1 (14.3)	14 (12.4)	4 (22.2)	11 (14.7)	4 (30.8)	1 (3.6)	1 (50.0)	1 (8.3)	
**Reverse marker biased strategy**	0 (0)	0 (0)	0 (0)	0 (0)	0 (0)	0 (0)	0 (0)	0 (0)	0 (0)	0 (0)	0 (0)	0 (0)	0 (0)
**Sequential Multiple Assignment Randomised trial**	1 (0.8)	0 (0)	1 (1.7)	0 (0)	0 (0)	0 (0)	1 (5.6)	0 (0)	0 (0)	0 (0)	0 (0)	1 (8.3)	0 (0)
**Tandem two stage design**	1 (0.8)	0 (0)	1 (1.7)	0 (0)	0 (0)	1 (0.9)	0 (0)	1 (1.3)	0 (0)	0 (0)	0 (0)	0 (0)	0 (0)
**Umbrella**	30 (22.9)	20 (31.7)	9 (15.0)	0 (0)	1 (14.3)	30 (26.5)	0 (0)	19 (25.3)	6 (46.2)	1 (3.6)	0 (0)	4 (33.3)	0 (0)
**Umbrella-basket hybrid**	2 (1.5)	2 (3.2)	0 (0)	0 (0)	0 (0)	2 (1.8)	0 (0)	2 (2.7)	0 (0)	0 (0)	0 (0)	0 (0)	0 (0)

[1] If the same clinical trial was labelled differently across articles, we considered the trial as example of the design reported in the paper. For instance, I-SPY 2 has been labelled as outcome-based adaptive randomisation (15), platform (36) or umbrella design (37) and it was considered as an example for each of those trial designs.

[2] Not found.

[3] Unknown is used to indicate a trial status that has not been verified within the past two years on the Clinicaltrials.gov website.

[4] Not applicable is used on the Clinicaltrials.gov website to describe trials without FDA-defined phases including trials of devices or behavioural interventions.

For peer review only

PRISMA 2009 Flow Diagram

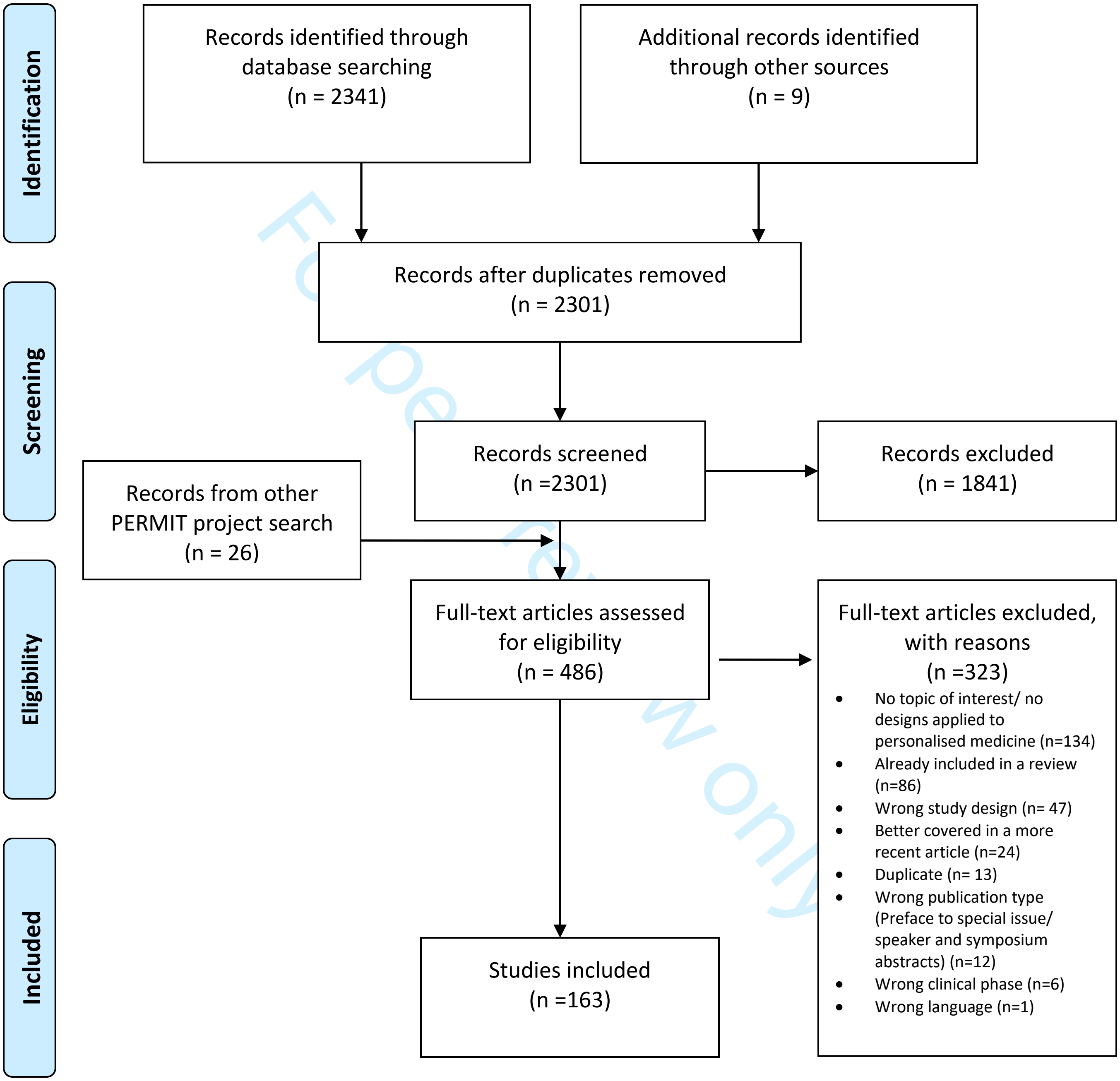

From: Moher D, Liberati A, Tetzlaff J, Altman DG, The PRISMA Group (2009). *P*referred *R*eporting *I*tems for *S*ystematic Reviews and *M*eta-*A*nalyses: The PRISMA Statement. PLoS Med 6(7): e1000097. doi:10.1371/journal.pmed1000097

For more information, visit www.prisma-statement.org.

For peer review only - http://bmjopen.bmj.com/site/about/guidelines.xhtml

Supplementary file I. Search strategies

Pubmed 7/4/2020

No.	Query	Results
#37	Search: #27 OR #30 Filters: English, French, German, Italian, Spanish Sort by: Publication Date	1221
#32	Search: #27 OR #30 Filters: from 2005 - 2020 Sort by: Publication Date	1232
#31	Search: #27 OR #30 Sort by: Publication Date	1277
#30	Search: #28 AND #29 Sort by: Publication Date	375
#29	Search: ("2019/09/01"[Date - Entry] : "3000"[Date - Entry]) Sort by: Publication Date	752605
#28	Search: #2 AND #25 AND (“clinical trial“ [tiab] OR “clinical trials“ [tiab]) Sort by: Publication Date	5359
#27	Search: #1 AND #2 AND #25 Sort by: Publication Date	918
#25	Search: design*[tiab] OR methods[ti] OR method[tiab] OR Research design[Majr] Sort by: Publication Date	3787147
#2	Search: “stratified medicine”[tiab] OR biomarker*[tiab] OR “precision medicine”[tiab] OR “personalized medicine”[tiab] OR “personalised medicine”[tiab] OR “individualized Medicine“[tiab] OR “individualised Medicine“[tiab] OR “individualized therapy“[tiab] OR “individualised therapy“[tiab] OR "Biomarkers"[Majr] OR "Precision Medicine"[Majr]	486778
#1	Search: “umbrella study“[tiab] OR “umbrella studies”[tiab] OR “umbrella trial“[tiab] OR “umbrella trials*“[tiab] OR “adaptive study“[tiab] OR “adaptive studies“[tiab] OR “adaptive trial“[tiab] OR “adaptive trials“[tiab] OR “ basket trial“[tiab] OR “basket trials“[tiab] OR “basket studies“[tiab] OR “basket study“[tiab] OR “multi arm“[tiab] OR “multi arms“[tiab] OR “master protocol“[tiab] OR “master protocols“[tiab]OR “platform study“[tiab] OR “platform studies“[tiab] OR “platform trial“[tiab] OR “platform trials“[tiab] OR"Clinical Trials as Topic"[Majr]	55630

For peer review only

Embase 7/4/202

No.	Query	Results
#14	#11 AND #12 AND ([english]/lim OR [french]/lim OR [german]/lim OR [italian]/lim OR [spanish]/lim)	927
#13	#11 AND #12	929
#12	[embase]/lim NOT [medline]/lim	9610086
#11	#7 OR #10	1221
#10	#4 AND #5 AND #8 AND [2020-2020]/py	202
#9	#4 AND #5 AND #8	7669
#8	'clinical trial*':ti,ab	514125
#7	#3 AND #4 AND #5 AND [2005-2020]/py	1026
#6	#3 AND #4 AND #5	1033
#5	design*:ti,ab OR methods:ti OR method:ti,ab	4793126
#4	'biological marker'/exp/mj OR 'personalized medicine'/exp/mj OR 'stratified medicine':ti,ab OR biomarker*:ti,ab OR 'precision medicine':ti,ab OR 'personalized medicine':ti,ab OR 'personalised medicine':ti,ab OR 'individualized medicine':ti,ab OR 'individualised medicine':ti,ab OR 'individualized therapy':ti,ab OR 'individualised therapy':ti,ab	431819
#3	#1 OR #2	52941
#2	'clinical trial'/exp/mj	50652
#1	'basket trial*':ti,ab OR 'basket stud*':ti,ab OR 'multi arm*':ti,ab OR 'master protocol*':ti,ab OR 'platform stud*':ti,ab OR 'platform trial*':ti,ab OR 'umbrella trial*':ti,ab OR 'adaptive stud*':ti,ab OR 'adaptive trial*':ti,ab OR 'umbrella stud*':ti,ab	2402

Cochrane Library 8/4/2020

No.	Query	Results
#1	'basket trial*':ti,ab OR 'basket stud*':ti,ab OR 'multi arm*':ti,ab OR 'master protocol*':ti,ab OR 'platform stud*':ti,ab OR 'platform trial*':ti,ab OR 'umbrella trial*':ti,ab OR 'adaptive stud*':ti,ab OR 'adaptive trial*':ti,ab OR 'umbrella stud*':ti,ab	22497

#2	'stratified medicine':ti,ab OR biomarker*:ti,ab OR 'precision medicine':ti,ab OR 'personalized medicine':ti,ab OR 'personalised medicine':ti,ab OR 'individualized medicine':ti,ab OR 'individualised medicine':ti,ab OR 'individualized therapy':ti,ab OR 'individualised therapy':ti,ab	29297
#3	design*:ti,ab OR methods:ti OR method:ti,ab	355698
#4	#1 and #2 and #3 with Publication Year from 2005 to 2020, in Trials	560
#5	"accession number" near pubmed	662135
#6	"accession number" near embase	536983
#7	#5 or #6	998271
#8	#4 not #7	193

For peer review only

Supplementary file II. Data extraction form

No	
First author:	
Title of article:	
Contact details of author:	
Publication year:	
Type of paper:	○ Original research article reporting a clinical trial<br>○ Study protocol<br>○ Methodological study<br>○ Methodological review<br>○ Systematic review<br>○ Conference abstract<br>○ Commentary<br>○ Letter to the editor<br>○ Clinicaltrial.gov link<br>○ Guidance document<br>○ Please specify the regulatory or health technologies assessment agency, which issued the report<br>○ Other (please specify): ____________________
Study design type:	○ Umbrella design<br>○ Basket design<br>○ Bayesian basket design<br>○ Basket of baskets design<br>○ Marker stratified design (part of randomize-all design. Marker stratified design includes 1) Marker sequential test design, 2) Biomarker-positive and overall strategies with fall-back analysis, 3) Biomarker-positive and overall strategies with sequential assessment, 4) Biomarker-positive and overall strategies with parallel assessment)<br>○ Hybrid design (part of randomize-all design)<br>○ Biomarker-strategy design with biomarker assessment in the control arm (part of biomarker-based strategy design)<br>○ Biomarker-strategy design without biomarker assessment in the control arm (part of biomarker-based strategy design)<br>○ Biomarker-strategy design with treatment randomization in the control arm (part of biomarker-based strategy design)<br>○ Reverse marker-based strategy design (part of biomarker-based strategy design)<br>○ Two-stage adaptive seamless design<br>○ Multi-arm multi-stage design (MAMS) (also called Platform design. It is an extension of 2-stage adaptive seamless design)<br>○ Adaptive signature design (also called Two-stage

For peer review only

	adaptive signature design, adaptive two-stage design, Biomarker-adaptive signature design)<br>○ Outcome-based adaptive randomization design (also called Adaptive randomization Bayesian adaptive, Bayesian adaptive randomization, Combined dynamic multi-arm, Outcome-Adaptive randomization, Outcome-based Bayesian adaptive randomization)<br>○ Adaptive threshold sample-enrichment design (also called Threshold sample-enrichment approach, two-stage sample enrichment, two-stage sample-enrichment design strategy)<br>○ Adaptive patient enrichment design (also called adaptive accrual, adaptive accrual based on interim analysis, adaptive enrichment, adaptive modification of target population, adaptive population enrichment, two-stage adaptive design, two stage adaptive accrual)<br>○ Adaptive parallel Simon two-stage design (also called pick-the-winner, biomarker-adaptive parallel two stage, adaptive parallel, two-parallel Simon, two-stage design)<br>○ Stratified adaptive design<br>○ Tandem two stage design (also called Tandem two-step phase II trial, tandem-two step trial (phase II), Tandem two-step phase 2 trial design, Tandem two-step)<br>○ Other (please specify): ____________________
**Definition** of the trial design referred to in the paper (if reported):	Please copy and paste the exact text.<br>E.g., The design begins with a comparison between the experimental treatment and the standard treatment in the entire study population at a pre-specified level of significance. In case that the overall result is positive, it is considered that the treatment is beneficial and the trial is closed. If the comparison in the overall population is not promising, then the entire population is divided in order to develop and validate a biomarker, using a split sample strategy. More precisely, a portion of patients is used to detect a biomarker signature that best distinguishes subjects for which the novel treatment is better than the standard treatment. Hence, this approach (i) identifies patients who are more susceptible to a specific treatment during the initial stage of the study (at the interim analysis); (ii) it assesses the global treatment effect of the entire randomized study population through a powered test, and (iii) finally, it assesses the treatment effect for the biomarker-positive subgroup identified during the initial stages of the study but only with patients randomized in the remainder of the trial, the so-called 'validation test'.

For peer review only

**Methodology** of the trial design referred to in the paper (if reported):	Analysis	Please copy and paste the exact text.<br>E.g., The analysis is undertaken as follows: At the interim analysis stage, if the overall treatment effect is not significant at a reduced level a1 (< 0.05), the full set of P patients in the clinical trial is partitioned into a training set Tr and a validation set V. A pre-specified algorithmic analysis plan is applied to the training set to generate a classifier Cl(x;Tr) where x is a biomarker vector.
	Other (please specify): ______________	Please copy and paste the exact text.
**Statistical considerations** of the trial design referred to in the paper (if reported):		Please copy and paste the exact text.<br>E.g., Although the adaptive signature design allows for approval of the novel treatment in a quick and efficient way, the main statistical challenges to be taken into account include the potential increase in the number of patients and the limited power to assess the treatment effect in the biomarker-defined subgroup. However, this approach avoids introduction of bias since the adaptations do not involve modifications in allocation ratio and eligibility criteria. Further, it prevents the inflation Type I error rate as the design does not use the study population which was employed to develop the predictive signature for the assessment of the treatment effect.
**Utility** of the trial design referred to in the paper (if reported):		Please list the reasons why it is recommended to use the study design by coping and pasting the exact text. Each point corresponds to a reason.<br>E.g., 1) In cases where we want to know whether the biomarker is not only prognostic but also predictive, this design is preferable.<br>○ ______________<br>○ ______________<br>○ ______________<br>○ ______________<br>○ ______________<br>○ ______________
**Advantages** of the trial design referred to in the paper (if reported):		Please list the advantages by coping and pasting the exact text. Each point corresponds to strength of the study design.<br>E.g., 1) Identification of optimal group of patients which benefit the most from a specific treatment; 2) Identification and validation of candidate biomarker in a single trial, etc.<br>○ ______________<br>○ ______________<br>○ ______________<br>○ ______________<br>○ ______________<br>○ ______________
**Disadvantages** of the trial design referred to in the paper (if reported):		Please list the disadvantages by coping and pasting the exact text. Each point corresponds to a limitation of the study design.<br>○ ______________<br>○ ______________<br>○ ______________

For peer review only

	○ ______<br>○ ______<br>○ ______<br>○ ______
**Gaps** in the study design methodology to be addressed in future research (if reported):	Please list the gaps by coping and pasting the exact text. Each point corresponds to a gap of the study design.<br>○ ______<br>○ ______<br>○ ______<br>○ ______<br>○ ______<br>○ ______
Example of actual trial(s), which have adopted the design mentioned.	Please report the exact name of the trial (e.g., NCI-MATCH trial)
Current status of the trial(s):	○ Ongoing trial<br>○ Completed trial
Trial registration number:	Please report the number
Clinical field:	○ Cancer<br>▪ (please specify):______<br>○ No cancer<br>▪ (please specify):______
Type of intervention:	○ Pharmaceutical<br>○ Non pharmaceutical
Clinical trial phase	○ Phase II<br>○ Phase III
Eligibility criteria:	○ ______<br>○ ______
Patient subgroups:	○ ______<br>○ ______
Intervention(s):	○ ______<br>○ ______
Control group:	○ ______<br>○ ______
Primary outcome measure(s):	○ ______<br>○ ______
External validity:	○ ______<br>○ ______
Did the study assess a personalised vs. non-personalised strategy?	○ Yes<br>○ No
Other considerations related to the study design:	

For peer review only

Supplementary file III. Included studies

1	Aanur P, Gutierrez M, Kelly RJ, Ajani JA, Ku GY, Denlinger CS, et al. FRACTION (Fast Real-time Assessment of Combination Therapies in Immuno-Oncology)-gastric cancer (GC): A randomized, open-label, adaptive, phase 2 study of nivolumab in combination with other immuno-oncology (IO) agents in patients with advanced GC. J Clin Oncol. 2017;35:TPS4137	Conference abstract
2	Abrams J, Conley B, Mooney M, Zwiebel J, Chen A, Welch JJ, et al. National Cancer Institute's Precision Medicine Initiatives for the New National Clinical Trials Network. Am Soc Clin Oncol Educ Book. 2014 May;(34):71–6.	Narrative review
3	Ahmad T, O'Connor CM. Therapeutic Implications of Biomarkers in Chronic Heart Failure. Clin Pharmacol Ther. 2013 Oct;94(4):468–79.	Narrative review
4	Alexander BM, Ba S, Berger MS, Berry DA, Cavenee WK, Chang SM, et al. Adaptive Global Innovative Learning Environment for Glioblastoma: GBM AGILE. Clin Cancer Res. 2018 Feb 15;24(4):737–43.	Narrative review
5	Alexander BM, Lorenzo T. Bayesian baskets: A novel approach to biomarker-based clinical trial design. J Clin Oncol. 2016;34: e14057	Conference abstract
6	Alexander BM, Trippa L, Gaffey S, Arrillaga-Romany IC, Lee EQ, Rinne ML, et al. Individualized Screening Trial of Innovative Glioblastoma Therapy (INSIGhT): A Bayesian Adaptive Platform Trial to Develop Precision Medicines for Patients With Glioblastoma. JCO Precis Oncol. 2019 Dec;(3):1–13.	Original research article reporting a clinical trial
7	Antoniou M, Jorgensen AL, Kolamunnage-Dona R. Biomarker-Guided Adaptive Trial Designs in Phase II and Phase III: A Methodological Review. PLOS ONE. 2016 Feb 24;11(2):e0149803.	Systematic review
8	Antoniou M, Kolamunnage-Dona R, Jorgensen A. Biomarker-Guided Non-Adaptive Trial Designs in Phase II and Phase III: A Methodological Review. J Pers Med. 2017 Jan 25;7(1):1.	Systematic review
9	Antoniou M, Kolamunnage-Dona R, Wason J, Bathia R, Billingham C, Bliss JM, et al. Biomarker-guided trials: Challenges in practice. Contemp Clin Trials Commun. 2019 Dec;16:100493.	Discussion paper
10	Bang Y-J, Kaufman B, Geva R, Stemmer SM, Hong S-H, Lee J-S, et al. An open-label, phase II basket study of olaparib and durvalumab (MEDIOLA): Results in patients with relapsed gastric cancer. J Clin Oncol. 2019;37:140	Conference abstract
11	Barroilhet L, Matulonis U. The NCI-MATCH trial and precision medicine in gynecologic cancers. Gynecol Oncol. 2018 Mar;148(3):585–90.	Narrative review
12	Barry WT, Perou CM, Marcom PK, Carey LA, Ibrahim JG. The Use of Bayesian Hierarchical Models for Adaptive Randomization in Biomarker-Driven Phase II Studies. J Biopharm Stat. 2015 Jan 2;25(1):66–88.	Methodological study
13	Bateman RJ, Benzinger TL, Berry S, Clifford DB, Duggan C, Fagan AM, et al. The DIAN-TU Next Generation Alzheimer's prevention trial: Adaptive design and disease progression model. Alzheimers Dement. 2017 Jan;13(1):8–19.	Original research article reporting a clinical trial

For peer review only

14	Beckman R, Antonijevic Z, Kalamegham R, Chen C. Adaptive Design for a Confirmatory Basket Trial in Multiple Tumor Types Based on a Putative Predictive Biomarker. Clin Pharmacol Ther. 2016 Dec;100(6):617–25.	Methodological study
15	Bell S, Copel J, Smith A. The pros and cons of an “umbrella” trial design for a rare disease from a trial management and data management perspective. Trials 2017; 18(Suppl 1): 200	Conference abstract
16	Berry DA. The Brave New World of clinical cancer research: Adaptive biomarker-driven trials integrating clinical practice with clinical research. Mol Oncol. 2015 May;9(5):951–9.	Narrative review
17	Berry SM, Broglio KR, Groshen S, Berry DA. Bayesian hierarchical modeling of patient subpopulations: Efficient designs of Phase II oncology clinical trials. Clin Trials J Soc Clin Trials. 2013 Oct;10(5):720–34.	Methodological study
18	Blagden SP, Billingham L, Brown LC, Buckland SW, Cooper AM, Ellis S, et al. Effective delivery of Complex Innovative Design (CID) cancer trials—A consensus statement. Br J Cancer. 2020 Feb 18;122(4):473–82.	Guidance document
19	Bradbury P, Hilton J, Seymour L. Early-phase oncology clinical trial design in the era of molecularly targeted therapy: pitfalls and progress. Clin Investig. 2011 Jan;1(1):33–44.	Narrative review
20	Brana I, Massard C, Baird RD, Opdam F, Schlenk RF, De Petris L, et al. Basket of baskets (BoB): A modular, open label, phase II, multicenter study to evaluate targeted agents in molecularly selected populations with advanced solid tumors. J Clin Oncol. 2019; 37: TPS3151	Conference abstract
21	Buch MH, Pavitt S, Parmar M, Emery P. Creative trial design in RA: optimizing patient outcomes. Nat Rev Rheumatol. 2013 Mar;9(3):183–94.	Narrative review
22	Cabarrou B, Sfumato P, Leconte E, Boher JM, Filleron T. Designing phase II clinical trials to target subgroup of interest in a heterogeneous population: A case study using an R package. Comput Biol Med. 2018 Sep;100:239–46.	Methodological study
23	Cafferkey C, Chau I, Thistlethwaite F, Petty RD, Starling N, WatkinsSheela Rao D, et al. PLATFORM: Planning treatment of oesophago-gastric (OG) cancer randomised maintenance therapy trial. J Clin Oncol. 2016; 34: TPS187	Conference abstract
24	Cecchini M, Rubin EH, Blumenthal GM, Ayalew K, Burris HA, Russell-Einhorn M, et al. Challenges with Novel Clinical Trial Designs: Master Protocols. Clin Cancer Res. 2019 Apr 1;25(7):2049–57.	Discussion paper
25	Chen C, Li X (Nicole), Yuan S, Antonijevic Z, Kalamegham R, Beckman RA. Statistical Design and Considerations of a Phase 3 Basket Trial for Simultaneous Investigation of Multiple Tumor Types in One Study. Stat Biopharm Res. 2016 Jul 2;8(3):248–57.	Methodological study
26	Cheng A-L. Combining Adaptive Design and Omics for Future HCC Trials. Liver Cancer 2015. 4: 1-257	Conference abstract
27	Clinicaltrials.gov. HIV Treatment Retention Interventions for Women Living With HIV (Siyaphambili Study) [Internet]. Available from: https://clinicaltrials.gov/ct2/show/NCT03500172	Link

For peer review only

28	Clinicaltrials.gov. Liver Immunosuppression Free Trial (LIFT) [Internet]. Available from: https://clinicaltrials.gov/ct2/show/NCT02498977	Link
29	Clinicaltrials.gov. ProBio: A Biomarker Driven Study in Patients With Metastatic Castrate Resistant Prostate Cancer (ProBio) [Internet]. Available from: https://clinicaltrials.gov/ct2/show/NCT03903835	Link
30	Cochrane Library. Trial for the optimisation of risk assessment and therapy success predicition in patients with early breast cancer by the use of biomarkers in advance to therapy decission-making to personalize therapies [Internet]. Available from: https://www.cochranelibrary.com/central/doi/10.1002/central/CN-01873376/full	Link
31	Conter HJ, MacDonald LD, Fiset S, Bramhecha YM, Chaney M, Rosu GN. Safety and efficacy results of the combination of DPX-Survivac, pembrolizumab and intermittent low dose cyclophosphamide (CPA) in subjects with advanced and metastatic solid tumours: Preliminary results from the hepatocellular carcinoma (HCC), NSCLC, bladder cancer, & MSI-H cohorts. Ann Oncol. 2019 Oct;30:v494.	Conference abstract
32	Coyne GO, Takebe N, Chen AP. Defining precision: The precision medicine initiative trials NCI-MPACT and NCI-MATCH. Curr Probl Cancer. 2017 May;41(3):182–93.	Narrative review
33	D'Angelo S, Blay J, Chow W, Demetri G, Thistlethwaite FC, Wagner M, et al. Autologous T cells with NY-ESO-1-specific T-cell receptor (GSK3377794) in HLA-A*02+previously-treated and -untreated advanced metastatic/unresectable synovial sarcoma: A master protocol study design. Journal for Immunotherapy of Cancer. 2019;7:282	Conference abstract
34	De Mattos-Arruda L, Rodon J. Pilot Studies for Personalized Cancer Medicine: Focusing on the Patient for Treatment Selection. The Oncologist. 2013 Nov;18(11):1180–8.	Narrative review
35	Debily M-A, Kergrohen T, Varlet P, Le Teuff G, Nysom K, Blomgren K, et al. PDTM-36. Whole exome sequencing (WES) of DIPG patients from the BIOMEDE trial reveals new prognostic subgroups with specific oncogenis programmes. Neuro-Oncology 2019;21 (Suppl 6): vi195.	Conference abstract
36	Diao G, Dong J, Zeng D, Ke C, Rong A, Ibrahim JG. Biomarker threshold adaptive designs for survival endpoints. J Biopharm Stat. 2018 Nov 2;28(6):1038–54.	Methodological study
37	Dienstmann R, Rodon J, Tabernero J. Optimal design of trials to demonstrate the utility of genomically-guided therapy: Putting Precision Cancer Medicine to the test. Mol Oncol. 2015 May;9(5):940–50.	Narrative review
38	Do K, Coyne GO, Chen AP. An overview of the NCI precision medicine trials—NCI MATCH and MPACT. Chin Clin Oncol. 2015;4(3):8.	Narrative review
39	Domchek SM, Postel-Vinay S, Im S-A, Hee Park Y, Delord J-P, Italiano A, et al. An open-label, phase II basket study of olaparib and durvalumab (MEDIOLA): Updated results in patients with germline BRCA-mutated (gBRCAm) metastatic breast cancer (MBC). Cancer Res. 2019;79: PD5-04	Conference abstract

For peer review only

40	Doorenbos AZ, Haozous EA, Jang MK, Langford D. Sequential multiple assignment randomization trial designs for nursing research. Res Nurs Health. 2019 Dec;42(6):429–35.	Methodological study
41	Eng KH. Randomized reverse marker strategy design for prospective biomarker validation. Stat Med. 2014 Aug 15;33(18):3089–99.	Methodological study
42	Fadoukhair Z, Zardavas D, Chad MA, Goulioti T, Aftimos P, Piccart M. Evaluation of targeted therapies in advanced breast cancer: the need for large-scale molecular screening and transformative clinical trial designs. Oncogene. 2016 Apr;35(14):1743–9.	Narrative review
43	Fennell D, Hudka M, Darlison L, Lord K, Bzura A, Dzialo J, et al. P2.06-02 Mesothelioma Stratified Therapy (MiST): A Phase IIA Umbrella Trial for Accelerating the Development of Precision Medicines. J Thorac Oncol. 2019 Oct;14(10):S755–6.	Conference abstract
44	Ferrarotto R, Redman MW, Gandara DR, Herbst RS, Papadimitrakopoulou V. Lung-MAP-framework, overview, and design principles. Chin Clin Oncol. 2015;4(3):1–6.	Narrative review
45	Fountzilas E, Tsimberidou AM. Overview of precision oncology trials: challenges and opportunities. Expert Rev Clin Pharmacol. 2018 Aug 3;11(8):797–804.	Narrative review
46	Fracasso PM, Freeman DJ, Simonsen K, Shen Y, Gupta M, Comprelli A, et al. A phase 2, fast real-time assessment of combination therapies in immuno-oncology trial in patients with advanced non-small cell lung cancer (FRACTION-lung). Ann Oncol. 2016 Oct;27:vi451.	Conference abstract
47	Freidlin B, Korn EL, Gray R. Marker Sequential Test (MaST) design. Clin Trials J Soc Clin Trials. 2014 Feb;11(1):19–27.	Methodological study
48	Freidlin B, Korn EL. Biomarker-adaptive clinical trial designs. Pharmacogenomics. 2010 Dec;11(12):1679–82.	Editorial
49	Freidlin B, McShane LM, Korn EL. Randomized Clinical Trials With Biomarkers: Design Issues. JNCI J Natl Cancer Inst. 2010 Feb 3;102(3):152–60.	Commentary
50	Funcke S. Individualized, perioperative, hemodynamic goal-directed therapy in major abdominal surgery (iPEGASUS trial): study protocol for a randomized controlled trial. 2018;10.	Study protocol
51	Galanis E, Wu W, Sarkaria J, Chang SM, Colman H, Sargent D, et al. Incorporation of Biomarker Assessment in Novel Clinical Trial Designs: Personalizing Brain Tumor Treatments. Curr Oncol Rep. 2011 Feb;13(1):42–9.	Narrative review
52	Galot R, Le Tourneau C, Saada-Bouzid E, Daste A, Even C, Debruyne PR, et al. A phase II study of monalizumab in patients with recurrent/metastatic (RM) squamous cell carcinoma of the head and neck (SCCHN): Results of the I1 cohort of the EORTC-HNCG-1559 trial (UPSTREAM). Ann Oncol. 2019 Oct;30:v449–50.	Conference abstract
53	Gandara DR, Hammerman PS, Sos ML, Lara PN, Hirsch FR. Squamous Cell Lung Cancer: From Tumor Genomics to Cancer Therapeutics. Clin Cancer Res. 2015 May 15;21(10):2236–43.	Narrative review
54	Gao Z, Roy A, Tan M. Multistage adaptive biomarker-directed targeted design for randomized clinical trials. Contemp Clin Trials. 2015 May;42:119–31.	Methodological study

For peer review only

55	Garralda E, Dienstmann R, Piris-Giménez A, Braña I, Rodon J, Tabernero J. New clinical trial designs in the era of precision medicine. Mol Oncol. 2019 Mar;13(3):549–57.	Narrative review
56	Gilson C, Chowdhury S, Parmar MKB, Sydes MR. Incorporating Biomarker Stratification into STAMPEDE: an Adaptive Multi-arm, Multi-stage Trial Platform. Clin Oncol. 2017 Dec;29(12):778–86.	Narrative review
57	Gómez-López G, Dopazo J, Cigudosa JC, Valencia A, Al-Shahrour F. Precision medicine needs pioneering clinical bioinformaticians. Brief Bioinform. 2019 May 21;20(3):752–66.	Narrative review
58	Grill J, Teuff GL, Nysom K, Blomgren K, Hargrave D, McCowage G, et al. PDCT-01. Biological medicine for diffuse intrinsic pontine gliomas eradication (BIOMEDE): Results of the three-arm biomarker-driven randomized trial in the first 230 patients from Europe and Australia. Neuro-Oncology 2019; 21 (Suppl 6): vi183.	Conference abstract
59	Gronberg H, Eklund M, Lindberg J, Ullén A, Bjartell A, Andren O, et al. ProBio II: An adaptive and randomized multi-arm biomarker driven phase 2 study in men with castrate resistant prostate cancer (CRPC). J Clin Oncol. 2018; 36: TPS397	Conference abstract
60	Grose DB, McKay CJ, Cooke S, Graham JS, Duthie F, Jamieson N, et al. PRIMUS-002: A multicentre, open-label, phase II study examining FOLFOX and nab-paclitaxel (FA) and nab-paclitaxel and gemcitabine (AG) as neoadjuvant therapy for (borderline) resectable pancreatic cancer (PC), focusing on biomarker and liquid biopsy development. J Clin Oncol. 2019; 37: TPS4166	Conference abstract
61	Heckman-Stoddard BM, Smith JJ. Precision Medicine Clinical Trials: Defining New Treatment Strategies. Semin Oncol Nurs. 2014 May;30(2):109–16.	Narrative review
62	Heerspink HJL, List J, Perkovic V. New clinical trial designs for establishing drug efficacy and safety in a precision medicine era. Diabetes Obes Metab. 2018 Oct;20:14–8.	Narrative review
63	Heerspink HJL, Perkovic V. Trial Design Innovations to Accelerate Therapeutic Advances in Chronic Kidney Disease: Moving from Single Trials to an Ongoing Platform. Clin J Am Soc Nephrol. 2018 Jun 7;13(6):946–8.	Narrative review
64	Hernandez-Martinez J-M, Sánchez-Reyes R, De la Garza-Salazar JG, Arrieta O. Onco-omics Approaches and Applications in Clinical Trials for Cancer Patients. In: Translational Research and Onco-Omics Applications in the Era of Cancer Personal Genomics. Springer; 2019. p. 79–90.	Book chapter
65	Hobbs BP, Kane MJ, Hong DS, Landin R. Statistical challenges posed by uncontrolled master protocols: sensitivity analysis of the vemurafenib study. Ann Oncol. 2018 Dec;29(12):2296–301.	Methodological study
66	Hobbs BP, Landin R. Bayesian basket trial design with exchangeability monitoring: Bayesian Basket Trial Design with Exchangeability Monitoring. Stat Med. 2018 Nov 10;37(25):3557–72.	Methodological study

67	Hofmann D, Nitz U, Gluz O, Kates RE, Schinkoethe T, Staib P, et al. WSG ADAPT – adjuvant dynamic marker-adjusted personalized therapy trial optimizing risk assessment and therapy response prediction in early breast cancer: study protocol for a prospective, multi-center, controlled, non-blinded, randomized, investigator initiated phase II/III trial. Trials. 2013;14(1):261.	Study protocol
68	Hollingsworth SJ. Precision medicine in oncology drug development: a pharma perspective. Drug Discov Today. 2015 Dec;20(12):1455–63.	Narrative review
69	Hong F, Simon R. Run-In Phase III Trial Design With Pharmacodynamics Predictive Biomarkers. J Natl Cancer Inst. 2013;105(21):6.	Methodological study
70	Hyman DM, Taylor BS, Baselga J. Implementing Genome-Driven Oncology. Cell. 2017 Feb;168(4):584–99.	Narrative review
71	ISRCTN Registry. Precision Panc: Advancing personalised medicine treatment strategies for pancreatic cancer [Internet]. Available from: https://www.isrctn.com/ISRCTN14879538	Link
72	Janiaud P, Serghiou S, Ioannidis JPA. New clinical trial designs in the era of precision medicine: An overview of definitions, strengths, weaknesses, and current use in oncology. Cancer Treat Rev. 2019 Feb;73:20–30.	Narrative review
73	Johnson DR, Galanis E. Incorporation of Prognostic and Predictive Factors Into Glioma Clinical Trials. Curr Oncol Rep. 2013 Feb;15(1):56–63.	Narrative review
74	Jones CL, Holmgren E. An adaptive Simon Two-Stage Design for Phase 2 studies of targeted therapies. Contemp Clin Trials. 2007 Sep;28(5):654–61.	Methodological study
75	Joshi SS, Maron SB, Lomnicki S, Polite BN, Sharma M, Ibe J, et al. Personalized antibodies for gastroesophageal adenocarcinoma (PANGEA): A phase II precision medicine trial (NCT02213289) .J Clin Oncol. 2018; 36: TPS198	Conference abstract
76	Joshi YB, Light GA. Using EEG-Guided Basket and Umbrella Trials in Psychiatry: A Precision Medicine Approach for Cognitive Impairment in Schizophrenia. Front Psychiatry. 2018 Nov 19;9:554.	Narrative review
77	Kaplan R. The FOCUS4 design for biomarker stratified trials. Chin Clin Oncol. 2015;4(3):1–10.	Narrative review
78	Kesselmeier M, Scherag A. Adaptive clinical trials in sepsis research: pros and cons. Infection. 2019; 47 (Suppl 1): S1-S67.	Conference abstract
79	Kidwell KM, Wahed AS. Weighted log-rank statistic to compare shared-path adaptive treatment strategies. Biostatistics. 2013 Apr 1;14(2):299–312.	Methodological study
80	Kimani PK, Todd S, Renfro LA, Stallard N. Point estimation following two-stage adaptive threshold enrichment clinical trials: Estimators for adaptive threshold enrichment clinical trials. Stat Med. 2018 Sep 30;37(22):3179–96.	Methodological study
81	Korn EL, Freidlin B. Adaptive Clinical Trials: Advantages and Disadvantages of Various Adaptive Design Elements. JNCI J Natl Cancer Inst [Internet]. 2017 Jun [cited 2021 Mar 9];109(6). Available from: https://academic.oup.com/jnci/article-lookup/doi/10.1093/jnci/djx013	Commentary

For peer review only

82	Kourie HR, Aoun F. Why balls are not put inside the basket? A reflection on testicular cancer clinical trial design. Invest New Drugs. 2016 Aug;34(4):513–4.	Commentary
83	Krebs M, Ross K, Kim S, De Jonge M, Barlesi F, Postel-Vinay S, et al. P1.15-004 An Open-Label, Multitumor Phase II Basket Study of Olaparib and Durvalumab (MEDIOLA): Results in Patients with Relapsed SCLC. J Thorac Oncol. 2017 Nov;12(11):S2044–5.	Conference abstract
84	Kummar S, Williams PM, Lih C-J, Polley EC, Chen AP, Rubinstein LV, et al. Application of Molecular Profiling in Clinical Trials for Advanced Metastatic Cancers. JNCI J Natl Cancer Inst. 2015 Feb 6;107(4):djv003–djv003.	Commentary
85	Lam M, Loree JM, Lima AAP, Chun YS, Kopetz S. Accelerating Therapeutic Development through Innovative Trial Design in Colorectal Cancer. Curr Treat Options Oncol. 2018 Feb;19(2):11.	Narrative review
86	Lam VK, Papadimitrakopoulou V. Master protocols in lung cancer: experience from Lung Master Protocol. Curr Opin Oncol. 2018 Mar;30(2):92–7.	Narrative review
87	Le-Rademacher J, Dahlberg S, Lee JJ, Adjei AA, Mandrekar SJ. Biomarker Clinical Trials in Lung Cancer: Design, Logistics, Challenges, and Practical Considerations. J Thorac Oncol. 2018 Nov;13(11):1625–37.	Narrative review
88	Lee J-Y, Yi JY, Kim H-S, Lim J, Kim S, Nam BH, et al. An umbrella study of biomarker-driven targeted therapy in patients with platinum-resistant recurrent ovarian cancer: a Korean Gynecologic Oncology Group study (KGOG 3045), AMBITION. Jpn J Clin Oncol. 2019 Aug 1;49(8):789–92.	Study protocol
89	Lee J, Kim ST, Kim K, Lee H, Kozarewa I, Mortimer PG, et al. Tumor genomic profiling guides metastatic gastric cancer patients to targeted treatment: The VIKTORY Umbrella Trial. Cancer Discov. 2019 Jul 17;CD-19-0442.	Original research article reporting a clinical trial
90	Leonetti A, Boyd L, Giuliani J, Giovannetti E, Garajová I. Light and shadow on innovative clinical trial designs: reflections from the EORTC-PAMM course on ‘preclinical and early-phase clinical pharmacology.’ Expert Rev Clin Pharmacol. 2019 Nov 2;12(11):1033–6.	Narrative review
91	Li BT, Zauderer M, Chaft J, Drilon A, Eng J, Sima C, et al. Ado-trastuzumab emtansine for HER2 amplified or HER2 overexpressed cancers: A phase II “basket” trial. Cancer Res. 2015; 75: CT225	Conference abstract
92	Lin J-A, He P. Reinventing clinical trials: a review of innovative biomarker trial designs in cancer therapies. Br Med Bull. 2015 Jun;114(1):17–27.	Narrative review
93	Lindberg J, De Laere B, Crippa A, Eklund M, Grönberg H. ProBio: An outcome-adaptive, multi-arm, open-label, multiple assignment randomised controlled biomarker-driven trial in patients with metastatic castration-resistant prostate cancer. Ann Oncol. 2019 Oct;30:v354	Conference abstract
94	Liu S, Lee JJ. An overview of the design and conduct of the BATTLE trials. Chin Clin Oncol. 2015;4(3):13.	Narrative review
95	Lopez-Chavez A, Thomas A, Rajan A, Raffeld M, Morrow B, Kelly R, et al. Molecular Profiling and Targeted Therapy for Advanced Thoracic Malignancies: A Biomarker-Derived, Multiarm, Multihistology Phase II Basket Trial. J Clin Oncol. 2015 Mar 20;33(9):1000–7.	Original research article reporting a clinical trial

For peer review only

96	Maitland ML, Schilsky RL. Clinical trials in the era of personalized oncology. CA Cancer J Clin. 2011 Nov;61(6):365–81.	Narrative review
97	Mandrekar SJ, Dahlberg SE, Simon R. Improving Clinical Trial Efficiency: Thinking outside the Box. Am Soc Clin Oncol Educ Book. 2015 May;(35):e141–7.	Narrative review
98	Marabelle A, Le DT, Ascierto PA, Di Giacomo AM, De Jesus-Acosta A, Delord J-P, et al. Efficacy of Pembrolizumab in Patients With Noncolorectal High Microsatellite Instability/Mismatch Repair–Deficient Cancer: Results From the Phase II KEYNOTE-158 Study. J Clin Oncol. 2020 Jan 1;38(1):1–10.	Original research article reporting a clinical trial
99	Matsui S, Crowley J. Biomarker-Stratified Phase III Clinical Trials: Enhancement with a Subgroup-Focused Sequential Design. Clin Cancer Res. 2018 Mar 1;24(5):994–1001.	Methodological study
100	Mazzarella L, Morganti S, Marra A, Trapani D, Tini G, Pelicci P, et al. Master protocols in immuno-oncology: do novel drugs deserve novel designs? J Immunother Cancer. 2020 Mar;8(1):e000475.	Narrative review
101	Moore KN, Mannel RS. Is the NCI MATCH trial a match for gynecologic oncology? Gynecol Oncol. 2016 Jan;140(1):161–6.	Narrative review
102	Nakamura Y, Komatsu Y, Kato K, Shinozaki E, Bando H, Kato T, et al. bTMB-High Basket trial: A multicenter phase II trial of nivolumab monotherapy in patients with advanced gastrointestinal cancers with high blood tumor mutational burden (bTMB). J Clin Oncol 2019; 37: TPS179	Conference abstract
103	Nitz U, Gluz O, von Schumann R, Hofmann D, Kates RE, Kuemmel S, et al. ADAPT - Adjuvant Dynamic marker-Adjusted Personalized Therapy trial optimizing risk assessment and therapy response prediction in early breast cancer. Cancer Res. 2015; 75: OT3-2-04	Conference abstract
104	Noda S, Yonemori K, Shirakawa N, Okuma HS, Shimizu T, Hirakawa A, et al. MASTER KEY project: A basket/umbrella trial for rare cancers in Japan.Ann Oncol. 2018 Oct;29:viii148	Conference abstract
105	O'Brien C, Carter L, Cook N, Dean E. Novel Early Phase Clinical Trial Design in Oncology. Pharm Med. 2017 Oct;31(5):297–307.	Narrative review
106	Ocana A, Amir E, Vera-Badillo F, Seruga B, Tannock IF. Phase III Trials of Targeted Anticancer Therapies: Redesigning the Concept. Clin Cancer Res. 2013 Sep 15;19(18):4931–40.	Narrative review
107	Ohwada S, Morita S. Bayesian adaptive patient enrollment restriction to identify a sensitive subpopulation using a continuous biomarker in a randomized phase 2 trial. Pharm Stat. 2016 Sep;15(5):420–9.	Methodological study
108	Ondra T, Dmitrienko A, Friede T, Graf A, Miller F, Stallard N, et al. Methods for identification and confirmation of targeted subgroups in clinical trials: A systematic review. J Biopharm Stat. 2016 Jan 2;26(1):99–119.	Systematic review
109	Ou F-S, An M-W, Ruppert AS, Mandrekar SJ. Discussion of Trial Designs for Biomarker Identification and Validation Through the Use of Case Studies. JCO Precis Oncol. 2019 Dec;(3):1–10.	Narrative review

For peer review only

110	PanACEA consortium, Phillips P, Hoelscher M, Bratton D, Rehal S, Heinrich N, et al. Modifying the multi-arm multi-stage (MAMS) design for use in a phase II tuberculosis trial in sub-Saharan Africa with a time-to-event primary outcome. Trials. 2013 Nov;14(S1):P26, 1745-6215-14-S1-P26.	Conference abstract
111	Paoletti X, Asselain B, Kamal M, Servant N, Huppé P, Bieche I, et al. Design and statistical principles of the SHIVA trial. Chin Clin Oncol. 2015;4(3):1–10.	Narrative review
112	Park JJH, Hsu G, Siden EG, Thorlund K, Mills EJ. An overview of precision oncology basket and umbrella trials for clinicians. CA Cancer J Clin. 2020 Mar;70(2):125–37.	Narrative review
113	Park JJH, Siden E, Zoratti MJ, Dron L, Harari O, Singer J, et al. Systematic review of basket trials, umbrella trials, and platform trials: a landscape analysis of master protocols. Trials. 2019 Dec;20(1):572.	Systematic review
114	Park S, Hur JY, Yoon SE, Lee K, Kim Y, Cho JH, et al. P2.12-05 SUKSES (Small Cell Lung Cancer Umbrella Korea Studies): A Phase II Biomarker-Driven Umbrella Study in Relapsed or Refractory SCLC. J Thorac Oncol. 2018 Oct;13(10):S792.	Conference abstract
115	Park S, Shim J, Jung HA, Sun J-M, Lee S-H, Park W-Y, et al. Biomarker driven phase II umbrella trial study of AZD1775, AZD2014, AZD2811 monotherapy in relapsed small cell lung cancer. J Clin Oncol 2019: 37: 8514	Conference abstract
116	Parke T, Pericàs JM, Posch M, Heimbach N, Spiertz C, Mesenbrink P, et al. D2.1. Report on Terminology, References and Scenarios for Platform Trials and Master Protocols. 2020.	Report
117	Patel SP, Othus M, Chae YK, Giles FJ, Hansel DE, Singh PP, et al. A Phase II Basket Trial of Dual Anti–CTLA-4 and Anti–PD-1 Blockade in Rare Tumors (DART SWOG 1609) in Patients with Nonpancreatic Neuroendocrine Tumors. Clin Cancer Res. 2020 May 15;26(10):2290–6.	Original research article reporting a clinical trial
118	Perco P, Pena M, Heerspink HJL, Mayer G. Multimarker Panels in Diabetic Kidney Disease: The Way to Improved Clinical Trial Design and Clinical Practice? Kidney Int Rep. 2019 Feb;4(2):212–21.	Narrative review
119	Redman MW, Allegra CJ. The Master Protocol Concept. Semin Oncol. 2015 Oct;42(5):724–30.	Narrative review
120	Renfro LA, An M-W, Mandrekar SJ. Precision oncology: A new era of cancer clinical trials. Cancer Lett. 2017 Feb;387:121–6.	Narrative review
121	Renfro LA, Mallick H, An M-W, Sargent DJ, Mandrekar SJ. Clinical trial designs incorporating predictive biomarkers. Cancer Treat Rev. 2016 Feb;43:74–82.	Narrative review
122	Renfro LA, Mandrekar SJ. Definitions and statistical properties of master protocols for personalized medicine in oncology. J Biopharm Stat. 2018 Mar 4;28(2):217–28.	Narrative review
123	Renfro LA, Sargent DJ. Statistical controversies in clinical research: basket trials, umbrella trials, and other master protocols: a review and examples. Ann Oncol. 2017 Jan;28(1):34–43.	Narrative review
124	Riddell CA, Zhao Y, Petkau J. An adaptive clinical trials procedure for a sensitive subgroup examined in the multiple sclerosis context. Stat Methods Med Res. 2016 Aug;25(4):1330–45.	Methodological study

For peer review only

125	Rosenblum M, Hanley DF. Adaptive Enrichment Designs for Stroke Clinical Trials. Stroke. 2017 Jul;48(7):2021–5.	Narrative review
126	Said R, Tsimberidou A-M. Basket Trials and the MD Anderson Precision Medicine Clinical Trials Platform: Cancer J. 2019;25(4):282–6.	Narrative review
127	Saville BR, Berry SM. Efficiencies of platform clinical trials: A vision of the future. Clin Trials J Soc Clin Trials. 2016 Jun;13(3):358–66.	Methodological study
128	Schmoll H-J, Arnold D, de Gramont A, Ducreux M, Grothey A, O'Dwyer PJ, et al. MODUL—a multicenter randomized clinical trial of biomarker-driven maintenance therapy following first-line standard induction treatment of metastatic colorectal cancer: an adaptable signal-seeking approach. J Cancer Res Clin Oncol. 2018 Jun;144(6):1197–204.	Original research article reporting a clinical trial
129	Sebag-Montefiore D, Adams R, Bell S, Berkman L, Gilbert DC, Glynne-Jones R, et al. The Development of an Umbrella Trial (PLATO) to Address Radiation Therapy Dose Questions in the Locoregional Management of Squamous Cell Carcinoma of the Anus. Int J Radiat Oncol. 2016 Oct;96(2):E164–5.	Conference abstract
130	Semler MW, Bernard GR, Aaron SD, Angus DC, Biros MH, Brower RG, et al. Identifying Clinical Research Priorities in Adult Pulmonary and Critical Care. NHLBI Working Group Report. Am J Respir Crit Care Med. 2020 Aug 15;202(4):511–23.	Report
131	Shah SJ. Innovative Clinical Trial Designs for Precision Medicine in Heart Failure with Preserved Ejection Fraction. J Cardiovasc Transl Res. 2017 Jun;10(3):322–36.	Narrative review
132	Simon KC, Tideman S, Hillman L, Lai R, Jathar R, Ji Y, et al. Design and implementation of pragmatic clinical trials using the electronic medical record and an adaptive design. JAMIA Open. 2018 Jul 1;1(1):99–106.	Methodological study
133	Simon R. Clinical trial designs for evaluating the medical utility of prognostic and predictive biomarkers in oncology. Pers Med. 2010 Jan;7(1):33–47.	Narrative review
134	Simon R. Clinical trials for predictive medicine: new challenges and paradigms. Clin Trials J Soc Clin Trials. 2010 Oct;7(5):516–24.	Narrative review
135	Simon R. Critical Review of Umbrella, Basket, and Platform Designs for Oncology Clinical Trials: Review of umbrella, basket, and platform trial designs. Clin Pharmacol Ther. 2017 Dec;102(6):934–41.	Narrative review
136	Simon R. Genomic Alteration–Driven Clinical Trial Designs in Oncology. Ann Intern Med. 2016 Aug 16;165(4):270.	Narrative review
137	Simon R. New designs for basket clinical trials in oncology. J Biopharm Stat. 2018 Mar 4;28(2):245–55.	Narrative review
138	Simonsen KL, Fracasso PM, Bernstein SH, Wind-Rotolo M, Gupta M, Comprelli A, et al. The Fast Real-time Assessment of Combination Therapies in Immuno-ONcology (FRACTION) program: innovative, high-throughput clinical screening of immunotherapies. Eur J Cancer. 2018 Nov;103:259–66.	Original research article reporting a clinical trial
139	Skamene T, Siu LL, Renouf DJ, Laskin JJ, Bedard PL, Jones SJM, et al. Canadian profiling and targeted agent utilization trial (CAPTUR/PM.1): A phase II basket precision medicine trial. J Clin Oncol 2018. 36: TPS12127	Conference abstract

For peer review only

140	Soldatos, Kaduthanam, Jackson. Precision Oncology—The Quest for Evidence. J Pers Med. 2019 Sep 5;9(3):43.	Narrative review
141	Spigel D, Garassino M, Besse B, Sacher A, Barve M, Cousin S, et al. P1.01-110 Novel Regimens Versus Standard-of-Care in NSCLC: A Phase II, Randomized, Open-Label, Platform Trial Using a Master Protocol. J Thorac Oncol. 2019 Oct;14(10):S404–S405.	Conference abstract
142	Tajik P, Zwinderman AH, Mol BW, Bossuyt PM. Trial Designs for Personalizing Cancer Care: A Systematic Review and Classification. Clin Cancer Res. 2013 Sep 1;19(17):4578–88.	Systematic review
143	Talisa VB, Yende S, Seymour CW, Angus DC. Arguing for Adaptive Clinical Trials in Sepsis. Front Immunol. 2018 Jun 28;9:1502.	Narrative review
144	Tao JJ, Schram AM, Hyman DM. Basket Studies: Redefining Clinical Trials in the Era of Genome-Driven Oncology. Annu Rev Med. 2018 Jan 29;69(1):319–31.	Narrative review
145	Thavaneswaran S, Sebastian L, Ballinger M, Best M, Hess D, Lee CK, et al. Cancer Molecular Screening and Therapeutics (MoST): a framework for multiple, parallel signal-seeking studies of targeted therapies for rare and neglected cancers. Med J Aust. 2018 Oct;209(8):354–5.	Study protocol
146	Thavaneswaran S, Sebastian L, Ballinger M, Cowley M, Grady J, Joshua A, et al. The Cancer Molecular Screening and Therapeutics Program (MoST) – A molecular screening platform with multiple, parallel, signal-seeking therapeutic substudies. In Annals of Oncology; 2018. p. viii133–48.	Conference abstract
147	Timmers L, Van Waalwijk Van Doorn S, Pisters A, Van Saase L, Voest E. Ppm1 A Risk Sharing Model For Biomarker-Driven Treatment Of Rare Subgroups Of Cancer Patients. Value Health. 2019 Nov;22:S837.	Conference abstract
148	Trippa L, Alexander BM. Bayesian Baskets: A Novel Design for Biomarker-Based Clinical Trials. J Clin Oncol. 2017 Feb;35(6):JCO.2016.68.286.	Methodological study
149	Tsimberidou AM, Fountzilas E, Nikanjam M, Kurzrock R. Review of precision cancer medicine: Evolution of the treatment paradigm. Cancer Treat Rev. 2020 Jun;86:102019.	Narrative review
150	Uozumi R, Hamada C. Interim decision-making strategies in adaptive designs for population selection using time-to-event endpoints. J Biopharm Stat. 2017 Jan 2;27(1):84–100.	Methodological study
151	Verweij J, Hendriks HR, Zwierzina H, Hanauske, Wacheck V, Collignon O, et al. Innovation in oncology clinical trial design. Cancer Treat Rev. 2019 Mar;74:15–20.	Narrative review
152	Vijverberg SJ, Pijnenburg MW, Hövels AM, Koppelman GH, Maitland-van der Zee A-H. The need for precision medicine clinical trials in childhood asthma: rationale and design of the PUFFIN trial. Pharmacogenomics. 2017 Mar;18(4):393–401.	Report
153	Wang S-J, Hung HMJ, O'Neill R. Adaptive design clinical trials and trial logistics models in CNS drug development. Eur Neuropsychopharmacol. 2011 Feb;21(2):159–66.	Narrative review
154	Wang T, Wang X, Zhou H, Cai J, George SL. Auxiliary variable–enriched biomarker-stratified design. Stat Med. 2018 Dec 30;37(30):4610–35.	Methodological study

For peer review only

155	Weber J, Long GV, Haanen JB, Arance A, Dummer R, Nathan P, et al. A randomized, open-label, phase II open platform study evaluating the efficacy and safety of novel spartalizumab (PDR001) combinations in previously treated unresectable or metastatic melanoma (PLATForM). Ann Oncol. 2018;29:viii442-viii466	Conference abstract
156	Xu Y, Trippa L, Müller P, Ji Y. Subgroup-Based Adaptive (SUBA) Designs for Multi-arm Biomarker Trials. Stat Biosci. 2016 Jun;8(1):159–80.	Methodological study
157	Yee LM, McShane LM, Freidlin B, Mooney MM, Korn EL. Biostatistical and Logistical Considerations in the Development of Basket and Umbrella Clinical Trials: Cancer J. 2019;25(4):254–63.	Narrative review
158	Yu H, Goldberg S, Le X, Piotrowska Z, Smith P, Mensi I, et al. P2.01-22 ORCHARD: A Phase II Platform Study in Patients with Advanced NSCLC Who Have Progressed on First-Line Osimertinib Therapy. J Thorac Oncol. 2019 Oct;14(10):S647.	Conference abstract
159	Yuan Y. Invited session 11 - Recent developments in umbrella, basket and platform trial designs. Clinical Trials. 2018; 15(S2);35-192	Conference abstract
160	Zardavas D, Piccart-Gebhart M. Clinical Trials of Precision Medicine through Molecular Profiling: Focus on Breast Cancer. Am Soc Clin Oncol Educ Book. 2015 May;(35):e183–90.	Narrative review
161	Zhang W, Wang J, Menon S. Advancing cancer drug development through precision medicine and innovative designs. J Biopharm Stat. 2018 Mar 4;28(2):229–44.	Narrative review
162	Zhang Z, Chen R, Soon G, Zhang H. Treatment evaluation for a data-driven subgroup in adaptive enrichment designs of clinical trials: Treatment evaluation for a data-driven subgroup in adaptive enrichment designs of clinical trials. Stat Med. 2018 Jan 15;37(1):1–11.	Methodological study
163	Zhou Q, Zhang X-C, Tu H-Y, Gan B, Wang B-C, Xu C-R, et al. Biomarker-integrated study of single agent targeting molecular alterations of PI3KCA, MET, ALK, ROS1, KRAS, NRAS or BRAF in advanced NSCLC: Phase 2 umbrella trial in China (CTONG1505). Ann Oncol. 2018 Nov;29:ix113.	Conference abstract

For peer review only

Supplementary file IV. Trial designs applied to personalised medicine

Trial designs[1]	Sub-type of trial designs	Variations and other names[2]	Core designs	Feature domains[3]
**Marker stratified design** (1-9)<br>1) Marker-stratified design<br>2) Biomarker-stratified design<br>3) Stratified-Randomised design<br>4) Stratification design<br>5) Stratified design<br>6) Stratified Analysis design<br>7) Marker by treatment – interaction design<br>8) Marker-by-treatment interaction design<br>9) Treatment by marker interaction design<br>10) Treatment-by-marker interaction design<br>11) Marker x treatment interaction design<br>12) Treatment-marker interaction design<br>13) Biomarker-by-treatment interaction design<br>14) Non-targeted RCT (stratified by marker) design<br>15) Genomic Signature stratified designs<br>16) Signature-Stratified design<br>17) Randomisation or analysis stratified by biomarker status design<br>18) Marker-interaction design			Randomise-all	• Biomarker assessment<br>• Biomarker-positive and overall strategies<br>• Randomisation<br>• Subgroup specific
	**Subgroup specific design**	**Sequential-subgroup specific design** (1)<br>1) Sequential design<br>2) Sequential testing<br>3) Fixed-sequence 2 design<br>4) Hierarchical fixed sequence testing procedure	Randomise-all	
		**Parallel-subgroup specific design** (1)<br>1) Phase III biomarker-stratified design	Randomise-all	
	**Biomarker-positive and overall strategies**<br>*Trials allowing to study the treatment effect both in biomarker positives and the overall population*	**Biomarker-positive and overall strategies with parallel assessment** (1)<br>1) Overall/biomarker-positive design with parallel assessment<br>2) Prospective subset design<br>3) Hybrid design[4]	Randomise-all	
		**Biomarker-positive and overall strategies with sequential assessment** (1,10)<br>1) Overall/biomarker-positive design with sequential assessment<br>2) Sequential design<br>3) Fixed-sequence 2 design<br>4) Hierarchical fixed sequence testing procedure	Randomise-all	

		**Biomarker-positive and overall strategies with fall-back analysis** (1)<br><br>1) Biomarker-stratified design with fall-back analysis<br>2) Fall-back design<br>3) Prospective subset design<br>4) Sequential design<br>5) Other analysis plan design<br>6) Fallback design	Randomise-all	
		**Marker sequential test design** (1,11)<br><br>1) MaST design<br>2) Hybrid design[4]	Randomise-all	
		**Auxiliary variable–enriched biomarker-stratified design (AEBSD)[5]** (12)	Randomise-all[5]	
**Hybrid design** (1,5,13)<br><br>1) Mixture design<br>2) Combination of trial designs<br>3) Hybrid biomarker design			Randomise-all	• Biomarker assessment<br>• Randomisation
**Biomarker strategy design with biomarker assessment in the control arm** (1, 3-4, 13)<br><br>1) Marker strategy design<br>2) Biomarker-strategy design<br>3) Strategy design<br>4) Marker-based strategy design<br>5) Marker-based design<br>6) Random disclosure design<br>7) Customized strategy design<br>8) Parallel controlled pharmacogenetic study design<br>9) Marker-based strategy design I<br>10) Biomarker-guided design<br>11) Biomarker-based assignment of specific drug therapy design<br>12) Marker-based strategy I design<br>13) Biomarker-strategy design with a standard control<br>14) Marker strategy design for prognostic biomarkers			Biomarker-strategy	• Biomarker assessment<br>• Randomisation in the non-biomarker based strategy arm

**Biomarker strategy design without biomarker assessment in the control arm** (1,4-6,8,13,14)<br><br>1) Biomarker-strategy design with standard control<br>2) Direct-predictive biomarker-based<br>3) RCT of testing<br>4) Test-treatment<br>5) Parallel controlled pharmacogenetic diagnostic study<br>6) Marker strategy<br>7) Marker-based with no randomisation in the non-marker-based arm<br>8) Classical<br>9) Marker-based strategy<br>10) Marker strategy design for prognostic biomarkers			Biomarker-strategy	• Biomarker assessment<br>• Randomisation in the non-biomarker based strategy arm
**Biomarker strategy design with treatment randomisation in the control arm** (1,6,8,13)<br><br>1) Biomarker-strategy design with a randomised control<br>2) Modified marker-based strategy design (for predictive biomarkers)<br>3) Biomarker-strategy design with randomised control<br>4) Marker-based design with randomisation in the non-marker-based arm<br>5) Marker-based strategy design II<br>6) Marker-strategy design<br>7) Augmented strategy design<br>8) Trial design allowing the evaluation of both the treatment and the marker effect			Biomarker-strategy	• Biomarker assessment<br>• Randomisation in the non-biomarker based strategy arm
**Reverse marker based strategy** (1,8,15)			Biomarker-strategy	• Biomarker assessment<br>• Randomisation in the non-biomarker based strategy arm
**Modified biomarker strategy design** (3,13,14)<br><br>1) Modified marker based strategy design			Biomarker-strategy	• Biomarker assessment<br>• Randomisation
**Sequential Multiple Assignment Randomised Trial (SMART) design** (16,17)			Randomise-all	• Control group<br>• Treatment tailoring aspects
**Adaptive biomarker design** (14)<br><br>1) Biomarker adaptive design			Randomise-all	• Generic adaptive aspects<br>• Biomarker assessment<br>• PM specific adaptive aspects

**Adaptive strategy for biomarker with measurement error** (4)			Randomise-all	• Generic adaptive aspects<br>• Biomarker assessment
**Adaptive signature design** (9,14,18,19)<br><br>1) Two-stage adaptive signature design<br>2) Adaptive two-stage design<br>3) Biomarker adaptive signature design			Randomise-all	• Generic adaptive aspects<br>• PM specific adaptive aspects<br>• Biomarker assessment<br>• Inference framework
		**Adaptive threshold design** (14,18,20,21)<br><br>1) Biomarker adaptive threshold design	Randomise-all	
		**Molecular signature design** (18)	Randomise-all	
		**Cross-validated adaptive signature design** (13,18,19)	Randomise-all	
		**Generalized adaptive signature design** (14,18)	Randomise-all	
		**Adaptive signature design with subgroup plots** (18)	Randomise-all	
**Outcome-based adaptive randomisation design** (3,4,18,22-25)<br><br>1) Adaptive randomisation Bayesian adaptive<br>2) Bayesian adaptive randomisation<br>3) Combined dynamic multi-arm<br>4) Outcome-adaptive randomisation<br>5) Outcome-based Bayesian adaptive randomisation			Randomise-all	• Generic adaptive aspects<br>• Biomarker assessment<br>• Inference framework<br>• Model
		**Bayesian covariate adjusted response-adaptive randomisation** (18)	Randomise-all	
**Adaptive enrichment design**			Enrichment	• Generic adaptive aspects<br>• PM specific

	**Adaptive threshold sample-enrichment design** (4,13,14,18,26)<br><br>1) Threshold sample-enrichment approach<br>2) Two-stage sample enrichment<br>3) Two stage sample-enrichment design strategy<br>4) Two-stages adaptive threshold enrichment design		Enrichment	adaptive aspects<br>• Biomarker assessment<br>• Inference framework
	**Adaptive patient enrichment design** (3-5,13,18,19,27-29)<br><br>1) Adaptive accrual<br>2) Adaptive accrual based on interim analysis design<br>3) Adaptive enrichment<br>4) Adaptive modification of target population<br>5) Adaptive population enrichment<br>6) Two-stage adaptive design<br>7) Two stage adaptive accrual	**Modified Bayesian version of the two-stage design** (4,18)<br><br>1) Two-Stage Bayesian design<br>2) Bayesian adaptive enrichment design	Enrichment	
		**Adaptive design for population selection using correlated time to event endpoints** (30)	Randomise-all[6]	
		**Bayesian adaptive patient enrolment restriction (BAPER) approach** (31)	Randomise-all[6]	
		**Bayesian hierarchical model for response-adaptive randomised design** (32)	Randomise-all[6]	
		**Biomarker stratified with a subgroup-focused sequential design** (33)	Randomise-all[6]	

		**Stratified adaptive design** (18,33,34)<br><br>Adaptive stratified design	Randomise-all[6]	
**Adaptive parallel Simon two-stage design** (18,35)<br><br>1) Biomarker-adaptive parallel two-stage<br>2) Adaptive parallel<br>3) Two-parallel Simon<br>4) Two-stage design			Randomise-all	• Generic adaptive aspects<br>• Biomarker assessment
		**Parashar design** (34)	Randomise-all	
**Multi-arm multi-stage design** (18,36-38)<br><br>1) Adaptive biomarker-driven design<br>2) Adaptive analysis<br>3) Adaptive multi-stage designs<br>4) Multi-stage			Randomise-all	• Generic adaptive aspects<br>• Biomarker assessment<br>• PM specific adaptive aspects<br>• Inference framework
		**Two-stage adaptive seamless design** (4,5,18,22,39)<br><br>1) Seamless Phase II/III designs<br>2) Adaptive Seamless<br>3) Phase II/III Adaptive design<br>4) Two-stage Adaptive Seamless design<br>5) Adaptive Seamless Phase II/III design	Randomise-all	
		**Group sequential design** (18)	Randomise-all	
		**Bayesian subgroup based adaptive design (SUBA)** (40,41)	Randomise-all	
**Tandem two stage design** (18)<br><br>1) Tandem two-step phase II trial<br>2) Tandem-two step trial (phase II)<br>3) Tandem two-step phase 2 trial design<br>4) Tandem two-step			Randomise-all	• Generic adaptive aspects<br>• Biomarker assessment
**Platform design** (22,37,38,47,49,42-54)			Master protocols	• Generic adaptive aspects<br>• Control group<br>• Inference framework
	**Open adaptive platform** (55)	**Randomised, embedded multifactorial adaptive platform (REMAP)** (22)	Master protocols	

		**Bayesian Adaptive Platform Trial** (56)	Master protocols	
	**Closed platform** (55)		Master protocols	
**Basket design** (3,4,27,43,44,47,48,49,50,52,57,58,59,60,61,62,63,64,65,66,67, 68, 69,70,71,72,73,74,75,76)			Master protocols	• Biomarker assessment<br>• Inference framework<br>• Model<br>• Randomisation
	**Randomised basket design** (60,77)		Master protocols	
	**Non randomised basket design**		Master protocols	
		**Bayesian basket design** (60,78-80)	Master protocols	
		**Sequential basket trial design with Bayesian monitoring rules** (81)	Master protocols	
		**Bayesian latent subgroup trial (BLAST) design for basket trial** (82)	Master protocols	
		**Bayesian hierarchical adaptive design** (83)	Master protocols	
**Basket of basket design** (52,65)			Master protocols	• Biomarker assessment<br>• Inference framework<br>• Model<br>• Randomisation
**Umbrella design** (3,4,14,27,42, 43,44,47,48,49,50,51,52,57,60,61,62,65,66,67,70,72,74,75,80,8 4,85,86,87,88)			Master protocols	• Biomarker assessment<br>• Inference framework<br>• Model<br>• Randomisation

	**Randomised umbrella design** (89)		Master protocols	
	**Non randomised umbrella design**		Master protocols	
		**Bayesian adaptive umbrella design** (90)	Master protocols	
**Umbrella-basket hybrid** (91)			Master protocols	• Biomarker assessment<br>• Inference framework<br>• Model<br>• Randomisation

[1] The names reported listed under the design name header are alternate names for the same trial design.

[2] The trial designs reported in the *Variations and other names* column were identified in the literature and classified as variations by the research team based on previous classifications (1,18).

[3] The feature domains are referred to the trial designs. The feature domains include the key design features that characterise a trial design for personalised medicine, and that should be carefully considered when designing a trial. They are reported together with the corresponding detailed features in Table 1 (in the main article).

[4] "Marker sequential test design" and "Biomarker-positive and overall strategies with parallel assessment" are also named as "Hybrid design" in the literature, although they present a different trial design compared to what we meant as "Hybrid design"

[5] We classified Auxiliary variable–enriched biomarker-stratified design (AEBSD) as Randomise-all because both patients with positive and negative auxiliary biomarkers are randomised to the control and treatment arm. However, this design enriches the randomized cohort based on an inexpensive auxiliary variable, thereby avoiding testing the true biomarker on all screened patients and reducing treatment waiting time (92).

[6] These designs first use a Randomise-all design and based on the results of the interim analysis could enrich the population.

For peer review only

References

1. Antoniou M, Kolamunnage-Dona R, Jorgensen A. Biomarker-Guided Non-Adaptive Trial Designs in Phase II and Phase III: A Methodological Review. J Pers Med. 2017 Jan 25;7(1):1.

2. Ahmad T, O'Connor CM. Therapeutic Implications of Biomarkers in Chronic Heart Failure. Clin Pharmacol Ther. 2013 Oct;94(4):468–79.

3. Renfro LA, An M-W, Mandrekar SJ. Precision oncology: A new era of cancer clinical trials. Cancer Lett. 2017 Feb;387:121–6.

4. Renfro LA, Mallick H, An M-W, Sargent DJ, Mandrekar SJ. Clinical trial designs incorporating predictive biomarkers. Cancer Treat Rev. 2016 Feb;43:74–82.

5. Lin J-A, He P. Reinventing clinical trials: a review of innovative biomarker trial designs in cancer therapies. Br Med Bull. 2015 Jun;114(1):17–27.

6. Galanis E, Wu W, Sarkaria J, Chang SM, Colman H, Sargent D, et al. Incorporation of Biomarker Assessment in Novel Clinical Trial Designs: Personalizing Brain Tumor Treatments. Curr Oncol Rep. 2011 Feb;13(1):42–9.

7. Johnson DR, Galanis E. Incorporation of Prognostic and Predictive Factors Into Glioma Clinical Trials. Curr Oncol Rep. 2013 Feb;15(1):56–63.

8. Ondra T, Dmitrienko A, Friede T, Graf A, Miller F, Stallard N, et al. Methods for identification and confirmation of targeted subgroups in clinical trials: A systematic review. J Biopharm Stat. 2016 Jan 2;26(1):99–119.

9. Simon R. Clinical trials for predictive medicine: new challenges and paradigms. Clin Trials J Soc Clin Trials. 2010 Oct;7(5):516–24.

10. Yang B, Zhou Y, Zhang L, Cui L. Enrichment design with patient population augmentation. Contemp Clin Trials. 2015 May;42:60–7.

11. Freidlin B, Korn EL, Gray R. Marker Sequential Test (MaST) design. Clin Trials J Soc Clin Trials. 2014 Feb;11(1):19–27.

12. Wang T, Wang X, Zhou H, Cai J, George SL. Auxiliary variable–enriched biomarker-stratified design. Stat Med. 2018 Dec 30;37(30):4610–35.

13. Tajik P, Zwinderman AH, Mol BW, Bossuyt PM. Trial Designs for Personalizing Cancer Care: A Systematic Review and Classification. Clin Cancer Res. 2013 Sep 1;19(17):4578–88.

14. Simon R. Clinical trial designs for evaluating the medical utility of prognostic and predictive biomarkers in oncology. Pers Med. 2010 Jan;7(1):33–47.

15. Eng KH. Randomized reverse marker strategy design for prospective biomarker validation. Stat Med. 2014 Aug 15;33(18):3089–99.

16. Kidwell KM, Wahed AS. Weighted log-rank statistic to compare shared-path adaptive treatment strategies. Biostatistics. 2013 Apr 1;14(2):299–312.

17. Doorenbos AZ, Haozous EA, Jang MK, Langford D. Sequential multiple assignment randomization trial designs for nursing research. Res Nurs Health. 2019 Dec;42(6):429–35.

18. Antoniou M, Jorgensen AL, Kolamunnage-Dona R. Biomarker-Guided Adaptive Trial Designs in Phase II and Phase III: A Methodological Review. PLOS ONE. 2016 Feb 24;11(2):e0149803.

19. Zhang W, Wang J, Menon S. Advancing cancer drug development through precision medicine and innovative designs. J Biopharm Stat. 2018 Mar 4;28(2):229–44.

20. Diao G, Dong J, Zeng D, Ke C, Rong A, Ibrahim JG. Biomarker threshold adaptive designs for survival endpoints. J Biopharm Stat. 2018 Nov 2;28(6):1038–54.

21. Riddell CA, Zhao Y, Petkau J. An adaptive clinical trials procedure for a sensitive subgroup examined in the multiple sclerosis context. Stat Methods Med Res. 2016 Aug;25(4):1330–45.

22. Talisa VB, Yende S, Seymour CW, Angus DC. Arguing for Adaptive Clinical Trials in Sepsis. Front Immunol. 2018 Jun 28;9:1502.

23. Liu S, Lee JJ. An overview of the design and conduct of the BATTLE trials. Chin Clin Oncol. 2015;4(3):13.

24. Kesselmeier M, Scherag A. Adaptive clinical trials in sepsis research: pros and cons. In Infection; 2019. p. S1–67.

25. Wang S-J, Hung HMJ, O'Neill R. Adaptive design clinical trials and trial logistics models in CNS drug development. Eur Neuropsychopharmacol. 2011 Feb;21(2):159–66.

26. Kimani PK, Todd S, Renfro LA, Stallard N. Point estimation following two-stage adaptive threshold enrichment clinical trials: Estimators for adaptive threshold enrichment clinical trials. Stat

For peer review only

Med. 2018 Sep 30;37(22):3179–96.

27. Mandrekar SJ, Dahlberg SE, Simon R. Improving Clinical Trial Efficiency: Thinking outside the Box. Am Soc Clin Oncol Educ Book. 2015 May;(35):e141–7.

28. Rosenblum M, Hanley DF. Adaptive Enrichment Designs for Stroke Clinical Trials. Stroke. 2017 Jul;48(7):2021–5.

29. Zhang Z, Chen R, Soon G, Zhang H. Treatment evaluation for a data-driven subgroup in adaptive enrichment designs of clinical trials: Treatment evaluation for a data-driven subgroup in adaptive enrichment designs of clinical trials. Stat Med. 2018 Jan 15;37(1):1–11.

30. Uozumi R, Hamada C. Interim decision-making strategies in adaptive designs for population selection using time-to-event endpoints. J Biopharm Stat. 2017 Jan 2;27(1):84–100.

31. Ohwada S, Morita S. Bayesian adaptive patient enrollment restriction to identify a sensitive subpopulation using a continuous biomarker in a randomized phase 2 trial. Pharm Stat. 2016 Sep;15(5):420–9.

32. Barry WT, Perou CM, Marcom PK, Carey LA, Ibrahim JG. The Use of Bayesian Hierarchical Models for Adaptive Randomization in Biomarker-Driven Phase II Studies. J Biopharm Stat. 2015 Jan 2;25(1):66–88.

33. Matsui S, Crowley J. Biomarker-Stratified Phase III Clinical Trials: Enhancement with a Subgroup-Focused Sequential Design. Clin Cancer Res. 2018 Mar 1;24(5):994–1001.

34. Cabarrou B, Sfumato P, Leconte E, Boher JM, Filleron T. Designing phase II clinical trials to target subgroup of interest in a heterogeneous population: A case study using an R package. Comput Biol Med. 2018 Sep;100:239–46.

35. Jones CL, Holmgren E. An adaptive Simon Two-Stage Design for Phase 2 studies of targeted therapies. Contemp Clin Trials. 2007 Sep;28(5):654–61.

36. Kaplan R. The FOCUS4 design for biomarker stratified trials. Chin Clin Oncol. 2015;4(3):1–10.

37. Gilson C, Chowdhury S, Parmar MKB, Sydes MR. Incorporating Biomarker Stratification into STAMPEDE: an Adaptive Multi-arm, Multi-stage Trial Platform. Clin Oncol. 2017 Dec;29(12):778–86.

38. Van Norman GA. Phase II Trials in Drug Development and Adaptive Trial Design. JACC Basic Transl Sci. 2019 Jun;4(3):428–37.

39. Freidlin B, Korn EL. Biomarker-adaptive clinical trial designs. Pharmacogenomics. 2010 Dec;11(12):1679–82.

40. Xu Y, Trippa L, Müller P, Ji Y. Subgroup-Based Adaptive (SUBA) Designs for Multi-arm Biomarker Trials. Stat Biosci. 2016 Jun;8(1):159–80.

41. Simon KC, Tideman S, Hillman L, Lai R, Jathar R, Ji Y, et al. Design and implementation of pragmatic clinical trials using the electronic medical record and an adaptive design. JAMIA Open. 2018 Jul 1;1(1):99–106.

42. Park JJH, Siden E, Zoratti MJ, Dron L, Harari O, Singer J, et al. Systematic review of basket trials, umbrella trials, and platform trials: a landscape analysis of master protocols. Trials. 2019 Dec;20(1):572.

43. Leonetti A, Boyd L, Giuliani J, Giovannetti E, Garajová I. Light and shadow on innovative clinical trial designs: reflections from the EORTC-PAMM course on ‘preclinical and early-phase clinical pharmacology.’ Expert Rev Clin Pharmacol. 2019 Nov 2;12(11):1033–6.

44. Heerspink HJL, List J, Perkovic V. New clinical trial designs for establishing drug efficacy and safety in a precision medicine era. Diabetes Obes Metab. 2018 Oct;20:14–8.

45. Heerspink HJL, Perkovic V. Trial Design Innovations to Accelerate Therapeutic Advances in Chronic Kidney Disease: Moving from Single Trials to an Ongoing Platform. Clin J Am Soc Nephrol. 2018 Jun 7;13(6):946–8.

46. Perco P, Pena M, Heerspink HJL, Mayer G. Multimarker Panels in Diabetic Kidney Disease: The Way to Improved Clinical Trial Design and Clinical Practice? Kidney Int Rep. 2019 Feb;4(2):212–21.

47. Renfro LA, Mandrekar SJ. Definitions and statistical properties of master protocols for personalized medicine in oncology. J Biopharm Stat. 2018 Mar 4;28(2):217–28.

48. Mazzarella L, Morganti S, Marra A, Trapani D, Tini G, Pelicci P, et al. Master protocols in immuno-oncology: do novel drugs deserve novel designs? J Immunother Cancer. 2020

For peer review only

Mar;8(1):e000475.

49. Simon R. Critical Review of Umbrella, Basket, and Platform Designs for Oncology Clinical Trials: Review of umbrella, basket, and platform trial designs. Clin Pharmacol Ther. 2017 Dec;102(6):934–41.

50. Renfro LA, Sargent DJ. Statistical controversies in clinical research: basket trials, umbrella trials, and other master protocols: a review and examples. Ann Oncol. 2017 Jan;28(1):34–43.

51. Tsimberidou AM, Fountzilas E, Nikanjam M, Kurzrock R. Review of precision cancer medicine: Evolution of the treatment paradigm. Cancer Treat Rev. 2020 Jun;86:102019.

52. Verweij J, Hendriks HR, Zwierzina H, Hanauske, Wacheck V, Collignon O, et al. Innovation in oncology clinical trial design. Cancer Treat Rev. 2019 Mar;74:15–20.

53. Cecchini M, Rubin EH, Blumenthal GM, Ayalew K, Burris HA, Russell-Einhorn M, et al. Challenges with Novel Clinical Trial Designs: Master Protocols. Clin Cancer Res. 2019 Apr 1;25(7):2049–57.

54. Semler MW, Bernard GR, Aaron SD, Angus DC, Biros MH, Brower RG, et al. Identifying Clinical Research Priorities in Adult Pulmonary and Critical Care. NHLBI Working Group Report. Am J Respir Crit Care Med. 2020 Aug 15;202(4):511–23.

55. Saville BR, Berry SM. Efficiencies of platform clinical trials: A vision of the future. Clin Trials J Soc Clin Trials. 2016 Jun;13(3):358–66.

56. Alexander BM, Trippa L, Gaffey S, Arrillaga-Romany IC, Lee EQ, Rinne ML, et al. Individualized Screening Trial of Innovative Glioblastoma Therapy (INSIGhT): A Bayesian Adaptive Platform Trial to Develop Precision Medicines for Patients With Glioblastoma. JCO Precis Oncol. 2019 Dec;(3):1–13.

57. Janiaud P, Serghiou S, Ioannidis JPA. New clinical trial designs in the era of precision medicine: An overview of definitions, strengths, weaknesses, and current use in oncology. Cancer Treat Rev. 2019 Feb;73:20–30.

58. Dienstmann R, Rodon J, Tabernero J. Optimal design of trials to demonstrate the utility of genomically-guided therapy: Putting Precision Cancer Medicine to the test. Mol Oncol. 2015 May;9(5):940–50.

59. Gómez-López G, Dopazo J, Cigudosa JC, Valencia A, Al-Shahrour F. Precision medicine needs pioneering clinical bioinformaticians. Brief Bioinform. 2019 May 21;20(3):752–66.

60. Simon R. New designs for basket clinical trials in oncology. J Biopharm Stat. 2018 Mar 4;28(2):245–55.

61. Park JJH, Hsu G, Siden EG, Thorlund K, Mills EJ. An overview of precision oncology basket and umbrella trials for clinicians. CA Cancer J Clin. 2020 Mar;70(2):125–37.

62. Tao JJ, Schram AM, Hyman DM. Basket Studies: Redefining Clinical Trials in the Era of Genome-Driven Oncology. Annu Rev Med. 2018 Jan 29;69(1):319–31.

63. Berry DA. The Brave New World of clinical cancer research: Adaptive biomarker-driven trials integrating clinical practice with clinical research. Mol Oncol. 2015 May;9(5):951–9.

64. Fadoukhair Z, Zardavas D, Chad MA, Goulioti T, Aftimos P, Piccart M. Evaluation of targeted therapies in advanced breast cancer: the need for large-scale molecular screening and transformative clinical trial designs. Oncogene. 2016 Apr;35(14):1743–9.

65. Garralda E, Dienstmann R, Piris-Giménez A, Braña I, Rodon J, Tabernero J. New clinical trial designs in the era of precision medicine. Mol Oncol. 2019 Mar;13(3):549–57.

66. Le-Rademacher J, Dahlberg S, Lee JJ, Adjei AA, Mandrekar SJ. Biomarker Clinical Trials in Lung Cancer: Design, Logistics, Challenges, and Practical Considerations. J Thorac Oncol. 2018 Nov;13(11):1625–37.

67. Joshi YB, Light GA. Using EEG-Guided Basket and Umbrella Trials in Psychiatry: A Precision Medicine Approach for Cognitive Impairment in Schizophrenia. Front Psychiatry. 2018 Nov 19;9:554.

68. Simon R. Genomic Alteration–Driven Clinical Trial Designs in Oncology. Ann Intern Med. 2016 Aug 16;165(4):270.

69. Beckman R, Antonijevic Z, Kalamegham R, Chen C. Adaptive Design for a Confirmatory Basket Trial in Multiple Tumor Types Based on a Putative Predictive Biomarker. Clin Pharmacol Ther. 2016 Dec;100(6):617–25.

70. Moore KN, Mannel RS. Is the NCI MATCH trial a match for gynecologic oncology? Gynecol Oncol. 2016 Jan;140(1):161–6.

For peer review only

71. Hobbs BP, Kane MJ, Hong DS, Landin R. Statistical challenges posed by uncontrolled master protocols: sensitivity analysis of the vemurafenib study. Ann Oncol. 2018 Dec;29(12):2296–301.

72. O'Brien C, Carter L, Cook N, Dean E. Novel Early Phase Clinical Trial Design in Oncology. Pharm Med. 2017 Oct;31(5):297–307.

73. Said R, Tsimberidou A-M. Basket Trials and the MD Anderson Precision Medicine Clinical Trials Platform: Cancer J. 2019;25(4):282–6.

74. Shah SJ. Innovative Clinical Trial Designs for Precision Medicine in Heart Failure with Preserved Ejection Fraction. J Cardiovasc Transl Res. 2017 Jun;10(3):322–36.

75. Soldatos, Kaduthanam, Jackson. Precision Oncology—The Quest for Evidence. J Pers Med. 2019 Sep 5;9(3):43.

76. Zardavas D, Piccart-Gebhart M. Clinical Trials of Precision Medicine through Molecular Profiling: Focus on Breast Cancer. Am Soc Clin Oncol Educ Book. 2015 May;(35):e183–90.

77. Chen C, Li X (Nicole), Yuan S, Antonijevic Z, Kalamegham R, Beckman RA. Statistical Design and Considerations of a Phase 3 Basket Trial for Simultaneous Investigation of Multiple Tumor Types in One Study. Stat Biopharm Res. 2016 Jul 2;8(3):248–57.

78. Alexander BM, Lorenzo T. Bayesian baskets: A novel approach to biomarker-based clinical trial design. In 2016.

79. Trippa L, Alexander BM. Bayesian Baskets: A Novel Design for Biomarker-Based Clinical Trials. J Clin Oncol. 2017 Feb;35(6):JCO.2016.68.286.

80. Ou F-S, An M-W, Ruppert AS, Mandrekar SJ. Discussion of Trial Designs for Biomarker Identification and Validation Through the Use of Case Studies. JCO Precis Oncol. 2019 Dec;(3):1–10.

81. Hobbs BP, Landin R. Bayesian basket trial design with exchangeability monitoring: Bayesian Basket Trial Design with Exchangeability Monitoring. Stat Med. 2018 Nov 10;37(25):3557–72.

82. Yuan Y. Invited session 11 - Recent developments in umbrella, basket and platform trial designs. In Clinical Trials; 2018. p. 35–192.

83. Berry SM, Broglio KR, Groshen S, Berry DA. Bayesian hierarchical modeling of patient subpopulations: Efficient designs of Phase II oncology clinical trials. Clin Trials J Soc Clin Trials. 2013 Oct;10(5):720–34.

84. Blagden SP, Billingham L, Brown LC, Buckland SW, Cooper AM, Ellis S, et al. Effective delivery of Complex Innovative Design (CID) cancer trials—A consensus statement. Br J Cancer. 2020 Feb 18;122(4):473–82.

85. Ferrarotto R, Redman MW, Gandara DR, Herbst RS, Papadimitrakopoulou V. Lung-MAP-framework, overview, and design principles. Chin Clin Oncol. 2015;4(3):1–6.

86. Lam M, Loree JM, Lima AAP, Chun YS, Kopetz S. Accelerating Therapeutic Development through Innovative Trial Design in Colorectal Cancer. Curr Treat Options Oncol. 2018 Feb;19(2):11.

87. Lam VK, Papadimitrakopoulou V. Master protocols in lung cancer: experience from Lung Master Protocol. Curr Opin Oncol. 2018 Mar;30(2):92–7.

88. Yee LM, McShane LM, Freidlin B, Mooney MM, Korn EL. Biostatistical and Logistical Considerations in the Development of Basket and Umbrella Clinical Trials: Cancer J. 2019;25(4):254–63.

89. Lee J-Y, Yi JY, Kim H-S, Lim J, Kim S, Nam BH, et al. An umbrella study of biomarker-driven targeted therapy in patients with platinum-resistant recurrent ovarian cancer: a Korean Gynecologic Oncology Group study (KGOG 3045), AMBITION. Jpn J Clin Oncol. 2019 Aug 1;49(8):789–92.

90. Antoniou M, Kolamunnage-Dona R, Wason J, Bathia R, Billingham C, Bliss JM, et al. Biomarker-guided trials: Challenges in practice. Contemp Clin Trials Commun. 2019 Dec;16:100493.

91. Coyne GO, Takebe N, Chen AP. Defining precision: The precision medicine initiative trials NCI-MPACT and NCI-MATCH. Curr Probl Cancer. 2017 May;41(3):182–93.

92. Wang X, Zhou J, Wang T, George SL. On Enrichment Strategies for Biomarker Stratified Clinical Trials. J Biopharm Stat. 2018 Mar 4;28(2):292–308.

For peer review only

Supplementary file V. Definition, methodology, and statistical considerations of identified trial designs
The information on the definition, methodology and statistical considerations was extracted verbatim.

Trial designs	Sub-type of trial designs	Variations	Definition	Methodology	Statistical considerations
**Marker stratified design**			The marker-by-treatment interaction design detects the interaction between biomarker and treatment effect by using biomarker status as stratum (or strata) with the presumption that the entire population can be separated by marker-defined subgroup(s). (Lin2015)	All patients are randomly assigned to treatments, but the results are analyzed according to biomarker status. (Ahmad2013)	Marker-stratified designs can be conducted using two different testing plans; the so-called 1) marker-by-treatment interaction with separate tests and 2) marker-by-treatment interaction with interaction test. Both of these approaches involve conducting two independent clinical trials.<br><br>1) The marker-by-treatment interaction design using separate tests is a testing plan which determines whether the novel treatment is superior to the control treatment separately within each biomarker-defined subgroup. Consequently, the hypothesis to be tested, the calculation of the number of patients required for the trial, the estimation of the statistical power of the design and the randomization procedure of patients to different treatments are independent among the different subgroups. The sample size of the trial should be calculated in such a way so as to yield adequate statistical power when testing whether the experimental treatment is superior to the control treatment separately in the two biomarker-defined subgroups. Hence, this approach is not widely used due to the required large sample size as essentially two separate trials are being conducted. Another limitation of this approach is that when multiple biomarker-defined subsets and treatments are to be investigated, it is difficult to implement in practice.<br><br>2) The marker-by-treatment interaction using interaction test uses a test for interaction between the biomarker status and treatment assignment. A marker stratified design which uses this testing plan is also referred to in the literature as an "interaction design" or "genomic signature stratified design". First, a formal statistical test for interaction between biomarker status and treatment assignment is undertaken. If this interaction is not significant, then the study is continued by testing the different treatments overall at a two-sided significance level of 0.05, otherwise, the treatments are compared within each biomarker-defined subpopulation at a two-sided 0.05 significance level (i.e., the same as in the marker-by-treatment interaction design using separate tests). The sample size for this second testing plan is calculated with reference to the

For peer review only

					treatment effect in the entire study population. Therefore, it might not provide sufficient power for detecting the treatment effect in each biomarker defined-subset individually. More precisely, if the sample size is calculated for the overall analysis and the proportion of the biomarker-defined subpopulation which responds to the novel treatment is very small, the statistical power for the subgroup analysis may be inadequate. In addition, when several biomarker-defined subpopulations and treatments are to be investigated, this strategy is not easy to be implemented. (Antoniou2017)
				Individuals are stratified into biomarker-positive and biomarker-negative subgroups according to the results of the biomarker assessment and then they are randomized either to the experimental or to the control treatment group. The biomarker status in the Marker-Stratified design acts as a stratification factor where stratification is used to ensure balance across treatment groups with regard to biomarkers. Only individuals with valid biomarker results enter the trial. Consequently, we have four treatment groups, i.e., biomarker-positive patients assigned to either the experimental treatment arm or the control treatment arm and biomarker-negative patients assigned to either the experimental treatment arm or the control treatment arm. (Antoniou2017)	***It refers to marker-by-treatment interaction with separate tests***<br><br>The hypothesis to be tested, the sample size calculation and power estimation, and the randomization procedure are independent among subgroups. (Galanis2011)
			[…] a trial randomizing patients to experimental versus control treatments within marker-defined subgroups (Renfro2016_Clinical trial designs incorporating)	***It refers to marker-by-treatment interaction with separate tests***<br><br>[…] all patients with a valid marker result are assigned to a marker-based subgroup, and within each subgroup, patients are randomized between two or more treatment arms. (Galanis2011)	***It refers to marker-by-treatment interaction with interaction test***<br><br>[…] the sample size is calculated to provide adequate power to test for a different treatment effect in the two marker groups (Galanis2011)
				In this design, patients are randomized in different treatment groups. Although their biomarker status is prospectively determined, it does not impact on treatment decision. […] A variation on the marker by treatment interaction design allows for its use in trials in which each arm does not need to be individually powered to evaluate the primary hypothesis, but instead the trial as a whole is powered to assess for interaction between treatment effect and biomarker subgroup. (Johnson2013)	The sample size is, however, calculated to provide adequate power to test for a different treatment effect in the different marker groups (Johnson2013)

For peer review only - http://bmjopen.bmj.com/site/about/guidelines.xhtml

				The subjects are then randomized to treatment arms within marker defined groups. Statistical modeling including interaction effect or statistical test for dependency between two factors, such as interaction term of treatment by biomarker for continuous end point or X^2 for categorical end point, may then be implemented. (Lin2015)	[…] several null hypotheses are tested to examine the efficacy of the experimental treatment. This leads to Type I error rate inflation and a multiplicity adjustment must be applied to control the familywise error rate (FWER) in the strong sense. (Ondra2016)
				This design includes four arms, where patients are screened for biomarker status and randomization, stratified for the biomarker status, is performed. Biomarker-positive as well as biomarker-negative patients are randomized to the treatment T and control C […]. (Ondra2016)	• Requires excellent assay performance<br>• Requires fast assay turn-around time<br><br>(From Table 1. Renfro2016_Clinical trial designs incorporating)
				In this design, all patients are randomized to experimental versus control treatments; however, patients are first stratified by marker status and then randomized to a treatment arm within their given marker cohort. (Renfro2017_Precision oncology)	
				In this case the RCT comparing the new treatment to control includes both test-positive and test-negative patients, but a prospective primary analysis plan stipulating how the test will be used in the analysis of treatment effect is defined in the protocol. (Simon2010_Clinical trials for predictive)	
	**Subgroup specific design**	**Sequential-subgroup specific design**		The sequential testing procedure uses the assumption that it is unlikely that the new treatment will be effective in the biomarker-negative patients unless it is effective in the biomarker-positive patients. First treatment effect is tested in the biomarker-positive subpopulation using the overall two-sided significance level α = 0.05 (Type I error); if this test is significant then treatment effect is tested in the biomarker-negative subgroup using the same level of significance α. (Antoniou2017)	[…] requires a smaller number of positive patients as compared to the second type of subgroup-specific design, the so-called parallel subgroup-specific design (Antoniou2017)
		**Parallel-subgroup specific design**	[…] evaluates treatment effects separately in the positive biomarker-defined subgroup and in the negative biomarker-defined subgroup simultaneously. (Antoniou2017)	In order to control the overall type I error rate of the design at the overall level of significance (Type I error) it is required to allocate this overall between the test for the biomarker-positive subgroup and the test for the biomarker-negative subgroup using the Bonferroni correction method for multiple testing.This trial design is powered in such a way so as to detect the treatment effect in each biomarker-defined subgroup separately. A higher portion of the type I error rate can be given for the test within the biomarker-positive subgroup in order to	

For peer review only

				maximize the power of the trial to identify the treatment effect in this subpopulation. However, even if there is a slight increase in the type I error probability spent on the test of one of the biomarker-define subgroups, the power would probably not change much. (Antoniou2017)	
	**Biomarker-positive and overall strategies**	**Biomarker-positive and overall strategies with parallel assessment**	In the parallel version, we test both the overall population and biomarker-positive subgroup simultaneously. (Antoniou2017)	In this approach the treatment effect is tested in both the entire study population and in the biomarker-positive patients while controlling the type I error by allocating the overall significance level between the two tests. The significance level a can be considered as one-sided or two-sided. (Antoniou2017)	
		**Biomarker-positive and overall strategies with sequential assessment**		In this sequential version of the biomarker-positive and overall strategies, we first test the biomarker-positive subgroup using the significance level a; if the test is significant, then we test the treatment effect in the overall population using the same a level. The significance levels a can be considered as one-sided or two-sided significance levels. (Antoniou2017)	As this design comprises two sequential stages, it follows that the sample size calculation should also be staged. At the first stage, the standard formula for a traditional randomized trial can be used for the biomarker-positive subgroup using the significance level a to estimate the treatment effect in that subset. More precisely, the formula used in the enrichment design for the required total number of events or the required number of patients can be used at the first stage of this design. At the second stage, the sample size must be adjusted in order to yield appropriate power for the entire population. (Antoniou2017)
		**Biomarker-positive and overall strategies with fall-back analysis**	It evaluates both the treatment effect in the overall study population and in the biomarker-positive subgroup sequentially. (Antoniou2017)	In the fall-back design, we first test the overall population using the reduced significancance level α^1 and if the test is significant, we consider that the novel treatment is effective in the overall population; however, if the result is not significant then we test the treatment effect in the biomarker-positive subgroup using the level of significance $\alpha^2 = \alpha - \alpha^1$,where a is the overall significance level (Type I error rate). The significance levels a can be considered as one-sided or two-sided significance levels. (Antoniou2017)	The sample size should be set in such a way so as to yield adequate power for the overall test at the reduced significance level $\alpha^1$and for the potential biomarker positive subgroup analysis at significance level $\alpha - \alpha^1$, (Antoniou2017)

For peer review only

		**Marker sequential test design**	[…] allows sequential testing of the treatment effect in the biomarker subgroups and overall population while controlling the relevant type I error rates. (Freidlin2014)	This design sequentially tests the treatment effect in the subgroups and the overall population. First, the biomarker-positive subgroup is tested at a reduced level α^{1}. If it is significant, then the biomarker negative subgroup is tested at the level α. If the biomarker-positive subgroup test is not significant, then the overall population is tested at the $\alpha^{2} = \alpha - a^{1}$ level. For any choice of a1 (in [0, α]), the design controls the probability of rejecting H0+ or H0- under the global null at level a. (Freidlin2014)	
			[…] it evaluates not only the biomarker-positive and biomarker-negative subgroups but also the entire population sequentially to limit the assessment of treatment effect in the overall population when it seems that the biomarker-positive subgroup does not benefit from the novel treatment. (Antoniou2017)	In this design which owns an adaptive nature, first the biomarker-positive subgroup is tested at a reduced level a^{1} in [0, α] and if the results is significant, then the biomarker-negative subgroup is tested at the global significance level a. Otherwise, if the result is not significant, then the overall population is tested at level $\alpha^{2} = \alpha - a^{1}$ in order to make a treatment recommendation for the biomarker-negative patients. (Antoniou2017)	
		**Auxiliary variable–enriched biomarker-stratified design (AEBSD)**	[…] we focus on a new auxiliary variable-enriched biomarker-stratified design (AEBSD) where the M+ subpopulation is enriched through an inexpensive auxiliary variable that is moderately or highly correlated to the true biomarker. This design retains the assessment of the treatment effects for the desired subpopulation and the overall population while maintaining the "enriched" feature of trial design for efficiency. (Wang2018)		
**Hybrid design**			In this approach, only the biomarker-positive patients are randomly assigned to either the experimental treatment group or to the control treatment group whereas the biomarker-negative patients receive the control treatment. (Antoniou2017)	Similar to the enrichment design, hybrid designs are powered to identify treatment effect only in the biomarker-defined subgroup, which is randomly assigned to the experimental or control treatment groups. Consequently, the same formula used for the required number of patients or events for the enrichment designs can be used for hybrid designs. (Antoniou2017)	
			The most straightforward hybrid design is an extension from enrichment design: subjects who do not have predicted responsive biomarker will stay in the study and receive standard care. (Lin2015)		

For peer review only

			[…] an enrichment flow is combined in parallel with a single-arm trial of standard therapy in biomarker-negative patients (Tajik2013)		
**Biomarker strategy design with biomarker assessment in the control arm**			Biomarker status is assessed in all patients enrolled in the trial, who are then randomly allocated to either the biomarker-strategy arm or to standard treatment. (Tajik2013)	First, the study population enrolled in the trial is tested for its marker status. Next, patients irrespective of their biomarker status are randomized either to the biomarker-based strategy arm (also referred to as personalized arm) or to the non-biomarker-based strategy arm. In the biomarker-based strategy arm, biomarker-positive patients receive the experimental treatment, whereas, biomarker-negative patients receive the control treatment. Patients who are randomized to the non-biomarker-based strategy arm receive the control treatment irrespective of their biomarker status. (Antoniou2017)	• Requires strong predictive marker evidence<br>• Requires excellent assay performance<br>• Requires fast assay turn-around time<br>• Enrolls and treats all eligible patients∑<br><br>(From Table 1. Renfro2016_Clinical trial designs incorporating)
			A design that focuses specifically on the role of a biomarker in the treatment decision-making process […]. (Renfro2016_Clinical trial designs incorporating)	In this design, patients are randomized at the time of screening to a treatment strategy (often standard of care) that ignores the biomarker versus a strategy taking biomarker status into account, through direct assignment to targeted therapies matched to the biomarker status of each eligible patient. Primary outcome analyses are then made between treatment strategies rather than specific treatments, with the hypothesis that better outcomes will be observed among those patients treated according to (versus independent of) their biomarker status. At the same time, questions regarding the best treatment for patient subgroups may remain unanswered as treatment randomization within marker subgroups may not occur. (Renfro2016_Clinical trial designs incorporating)	
				In this design, patients are screened for biomarkers and then randomized to a treatment strategy that takes biomarker status into account (often a targeted therapy) versus a treatment that ignores the biomarker (often a stardard care.) (Renfro2016_Precision oncology)	

For peer review only

**Biomarker strategy design without biomarker assessment in the control arm**			In settings where it is not feasible or ethical to evaluate the biomarker in all patients, biomarker status is only acquired in patients allocated to the biomarker-strategy arm. (Tajik2013)	In this approach, patients are again randomized between testing strategies (i.e.,biomarker-based strategy and non-biomarker-based strategy) but it differs in terms of the timing of biomarker evaluation. More precisely, first, patients are randomized to either the biomarker-based strategy or to the non-biomarker-based strategy. Next, this design evaluates the biomarkers only in patients who are assigned to the biomarker-based strategy. Patients who are found to be biomarker-positive will receive the experimental treatment and patients who are biomarker-negative will receive the control treatment. On the other hand, the population which is randomized to the non-biomarker-based strategy will receive the control treatment. (Antoniou2017)	The same mathematical formula for sample size calculation assuming a continuous clinical outcome proposed by Young et al. (2010) and the formula assuming binary outcome proposed by Eng, 2014 for the biomarker-strategy design with biomarker assessment in the control arm could be applied. Further, in terms of survival outcome, the same formula provided for the required number of events in the first version of biomarker-strategy designs (i.e., biomarker-strategy design with biomarker assessment in the control arm) could be considered. (Antoniou2017)
				In the marker-based strategy design, each patient with known marker status is randomly assigned to two strategy groups: the marker-based strategy group, and the non marker-based strategy group. All patients assigned to the marker-based strategy group are assigned to different treatments (standard or experimental) based on their biomarker status, while patients assigned to the non marker-based strategy group all receive the standard treatment. (Galanis2011)	
				Biomarker strategy design recruits eligible subjects regardless of their biomarker status, just like all-comer design. The subjects will then be randomized to control arm (to receive placebo or standard care) or experimental arm. For the subjects in the experimental arm, their biomarker status will be tested before they are assigned to intervention treatment group or control group depending on their biomarker status. (Lin2015)	
				Patients are randomized to either the control (without screening) or the biomarker-guided treatment strategy arm. Within the latter arm, the biomarker status is determined and all biomarker positive patients receive the experimental treatment T whereas the biomarker-negative patients receive the control C. (Ondra2016)	

For peer review only

				The control arm determines treatment using practice standards based on staging and existing prognostic factors. The new biomarker is not measured for patients that are randomized to the control arm. Patients randomized to the experimental arm have the candidate biomarker measured and this is used in conjunction with staging and other prognostic factors to determine treatment. This design is very flexible, but often very inefficient in the sense that the same objectives can be obtained with fewer patients using other designs. (Simon2010_Clinical trial designs for evaluating)	
**Biomarker strategy design with treatment randomisation in the control arm**			The biomarker-strategy design with treatment randomization in the control treatment is able to inform us about whether the biomarker-based strategy is better than not only the standard treatment but also better than the experimental treatment in the overall population. (Antoniou2017)	Patients are first randomly assigned to either the biomarker-based strategy arm or to the non-biomarker-based strategy arm. Next, patients who are allocated to the non-biomarker-based strategy are again randomized either to the experimental treatment arm or to the standard treatment arm irrespective of their biomarker status. Patients who are allocated to the biomarker-based strategy and who are biomarker-positive are given the experimental treatment and patients who are biomarker-negative are given the control treatment. (Antoniou2017)	This design may require a larger sample size because some of the biomarker-negative patients in the randomization arm also receive the control treatment and some of the biomarker-positive patients the experimental treatment. This leads to a diluted treatment effect and may result in lower statistical power. (Ondra2016)
			[…] patients randomized to the non-biomarker strategy arm are again randomized between the experimental treatment and control. This design tests the impact of the biomarker-guided strategy against a random allocation procedure which does not take the biomarker into account. (Ondra2016)	[…] all patients in the non marker-based strategy group will have a second randomization and are assigned to one of the two treatments being used in the marker-based group. (Galanis2011)	
			[…] modification of the biomarker-strategy design, wherein a second randomization between experimental versus control therapy replaces the control arm. (Tajik2013)		
**Reverse marker based strategy**			[…] version of biomarker-strategy designs where the non-biomarker-based strategy arm which is included in the three aforementioned subtypes of biomarker-strategy designs is replaced by the reverse marker-strategy arm. (Antoniou2017)	In this design patients are randomized either to the biomarker-based strategy arm or the reverse biomarker-based strategy arm. As in the previous three biomarker-strategy subtype designs, patients who are allocated to the biomarker-strategy arm receive the experimental treatment if they are biomarker-positive whereas biomarker-negative patients receive the control treatment. By contrast, patients who are	

				randomly assigned to the reverse biomarker-based strategy arm receive control treatment if they are biomarker-positive, whereas biomarker-negative patients receive experimental treatment. (Antoniou2017)	
			[…] it employs a two-arm randomization scheme, provides a direct estimate of the marker-strategy response rate, and evaluates the interaction between the marker and possible treatments. (Eng2014)	Patients are randomly assigned to one of the two treatment strategies. In the first arm biomarker-positive patients receive the experimental treatment whereas biomarker-negative patients are allocated to receive the control. By contrast, in the second arm biomarker-positive patients receive the control and biomarker-negative patients receive the treatment. (Ondra2016)	
**Modified biomarker strategy design**			[…] is similar to a marker strategy design, except that it includes multiple targeted molecular profiles, thereby accommodating a more heterogeneous patient population. (Renfro2017_Precision oncology)	In this framework, the final analysis compares the marker-based strategy arm versus the non marker- based strategy arm (i.e. conventional, physician-directed) across all profiles. (Renfro2017_Precision oncology)	
			[…] measuring the test in all patients and only randomizing patients for whom the treatment assignment is influenced by marker result (Simon2010_Clinical trial designs for evaluating)	Before randomization, the practice standard-determined treatment and the marker-based treatment are identified. Only patients for whom the two treatments differ are randomized. (Simon2010_Clinical trial designs for evaluating)	
			[…] only patients for whom the treatment assignment is influenced by biomarker results are randomized (Tajik2013)		
**Sequential Multiple Assignment Randomised Trial (SMART) design**			The SMART design is used to sequence interventions based on a person's response. As such, the SMART design involves comparing sequences of interventions in terms of the effectiveness of the intervention, as well as the adjustment of intervention components and duration. SMART designs provide a systematic approach for testing decision rules involved in sequencing interventions (Doorenbos2019)	[…] the planning process can be broken into four main components or key steps: (a) Formulate the research question(s) to be answered, (b) identify and decide the intervention sequences, (c) define the response to the interventions, and (d) identify tailoring variables. (Doorenbos2019)	
			The SMART design allows for the assessment and comparison of adaptive treatment strategies (ATSs, also known as dynamic treatment regimes), which consist of a sequence of individually tailored therapies during the course of treatment. (Kidwell2013)		

For peer review only - http://bmjopen.bmj.com/site/about/guidelines.xhtml

**Adaptive biomarker design**				Let S(k) denote the log-likelihood measure of treatment effect for patients who are positive for biomarker Bk and let k* denote the biomarker for which S(k) is maximum. The statistical significance of S(k*) is determined by permuting the treatment group labels of the patients and then re-evaluating the treatment effects within the positive subsets of the K binary classifiers. Using bootstrap resampling, one can evaluate the proportion of the times that each patient is included in the positive subset of the selected biomarker and obtain a confidence interval for the treatment effect in the selected subset. (Simon2010_Clinical trial designs for evaluating)	
**Adaptive strategy for biomarker with measurement error**				The trial is comprised of two stages: in the first stage, patients are randomized to treatment driven by the gold-standard biomarker versus standard of care chemotherapy, while the secondary marker value is also recorded. In the second stage, the trial may switch toward use of the cheaper secondary marker if the two markers are highly concordant for predicting strategy benefit at an interim analysis between the stages. At the trial's conclusion, the primary objective is comparison of treatment strategies with or without use of the primary or secondary biomarker. (Renfro2016_Clinical trial designs incorporating)	
**Adaptive signature design**			It is a two-stage Phase III non-Bayesian trial design for settings where an assay or signature that identifies sensitive patients (i.e, biomarker-positive patients) is not known at the outset. (Antoniou2016)	The design begins with a comparison between the experimental treatment and the standard treatment in the entire study population at a pre-specified level of significance. In case that the overall result is positive, it is considered that the treatment is beneficial and the trial is closed. If the comparison in the overall population is not promising, then the entire population is divided in order to develop and validate a biomarker, using a split sample strategy. More precisely, a portion of patients is used to detect a biomarker signature that best distinguishes subjects for which the novel treatment is better than the standard treatment. (Antoniou2016)	Although the adaptive signature design allows for approval of the novel treatment in a quick and efficient way, the main statistical challenges to be taken into account include the potential increase in the number of patients and the limited power to assess the treatment effect in the biomarker-defined subgroup. However, this approach avoids introduction of bias since the adaptations do not involve modifications in allocation ratio and eligibility criteria. Further, it prevents the inflation Type I error rate as the design does not use the study population which was employed to develop the predictive signature for the assessment of the treatment effect. (Antoniou2016)
			Develops a predictive signature in a training set of the trial and evaluates the treatment effect for signature and patients in the test set. (Simon2010_Clinical trial designs for evaluating)	If the overall treatment effect is not significant at a reduced level a1, the patients in the clinical trial are partitioned into a training set and a validation set. A classifier is developed in the training set. The classifier identifies the patients who appear to benefit from the new treatment T compared to the control C. Freidlin and Simon provided methods for developing this classifier based on whole genome transcript expression	Statistical tests should be conducted appropriately in this design to account for multiplicity. (Zhang2017_Advancing cancer drug)

For peer review only

				data, but the analysis approach can be used much more broadly. For example, the training set can be used just to select among a set of candidate single gene/protein classifiers or to optimize a pre-defined classifier with regard to a new platform for measurement. In any case, the classifier defined on the training set is used to classify the patients in the validation set as either sensitive, that is, predicted likely to benefit from the new treatment T relative to C or not sensitive. One then compares outcomes for the sensitive patients in the validation set who received T versus the sensitive patients in the validation set who received C. Let L denote the log-rank statistic (if outcomes are time-to-event) for this comparison of T versus C of sensitive patients in the validation set. If the statistical significance L is less than 0.05-a1 (e.g., 0.02), then treatment T is considered superior to C for the subset of the patients predicted to be sensitive using the classifier developed in the training set. Freidlin et al. [22] recently demonstrated that the power of this approach can be substantially increased by embedding the classifier development and validation process in a K-fold cross-validation. (Simon2010_Clinical trials for predictive)	
			The adaptive signature design (Freidlin et al., 2010) is a design proposed to select the subgroup using a large number of potential biomarkers by dividing patients into two groups: a training group and a validation group. (Zhang2017_Advancing cancer drug)	At the conclusion of the trial, the new treatment is compared with the control overall, using a threshold of significance of a1, which is somewhat less than the total. A finding of statistical significance at that level is taken as support of a claim that the treatment is broadly effective. At that point, no biomarkers have been tested on the patients, although patients must have tumor specimens collected to be eligible for the clinical trial. If the overall treatment effect is not significant at the a1 level, a second stage of analysis takes place. The patients are divided into a training set and a testing set. The data for patients in the training set is used to define a single subset of patients who are expected to be most likely to benefit from the new treatment compared with the control. Freidlin and Simon used a machine learning algorithm based on screening thousands of genes for those with expression values that interact with the treatment effect, but the design can be used with other algorithms and even with candidate	

				classifiers that do not involve gene expression. When that subset has been explicitly defined, the new treatment is compared with the control for patients in the test set who display the characteristics defined by that subset. The comparison of the new treatment with the control in the subset is restricted to patients in the test set in order to preserve the principle of separating the data used to develop a classifier from the data used to test treatment effects in subsets defined by that classifier. The comparison of treatment with control for the subset uses a threshold of significance of a-a1 in order to ensure that the overall chance of a false-positive conclusion is no greater than a. These thresholds can be sharpened using the methods of Song and Chi [39]. (Simon2010_Clinical trial designs for evaluating)	
				It combines a definitive test for treatment effect in entire patient population with identification and validation of a biomarker signature for the subgroup sensitive patient population. There are three elements in this design: (a) trial powered to detect the overall treatment effect at the end of the trial; (b) identification of the subgroup of patients who are likely to benefit to the targeted therapy at the first stage of the trial; (c) statistical hypothesis test to detect the treatment difference in sensitive patient population based only the subgroup of patients randomized in the latter half of the trial. These elements are pre-specified prospectively. (Zhang2017_Advancing cancer drug)	
		**Adaptive threshold design**	[…] the Adaptive Threshold design was suggested for settings in which a putative biomarker is measured on a continuous or graded scale with its threshold for detecting individuals who would benefit from the novel treatment not predefined at the initial stage of a Phase III trial. (Antoniou2016)	The difference between the main design (Adaptive Signature design) and this variant corresponds to the biomarker-positive subset. More precisely, in the main design, if there is no claim of treatment effectiveness in the entire population, then a portion of individuals is used to develop a predictive biomarker signature and the remaining portion is used to compare the treatment effect. However, in this variant if there is no claim of treatment effectiveness in the entire population, the design identifies and validates a cut-off point for a prospectively selected biomarker. Adaptations here are referred to the subgroup and there are no modifications regarding the required number of patients or randomization ratio. In this design, human samples are collected to measure a pre-	Two analysis plans compose this approach, the so-called 'analysis plan A' and 'analysis plan B'. The first plan is identical to the strategy proposed for the Adaptive Signature design. The second plan uses a more effective method to accommodate the multiplicity issue when combining the statistical tests for the entire population and the biomarker-defined subgroup by incorporating the correlation structure of the two test statistics. (Antoniou2016)

For peer review only

				specified biomarker from the entire population at the beginning of the study but the value of biomarker is not used as an eligibility criteria. (Antoniou2016)	
			[…] tumor specimens are collected from all patients at trial entry, but the value of the predictive index is not used as an eligibility criteria (Simon2010_Clinical trial designs for evaluating)	Analysis plan A begins with comparing the outcomes for all patients receiving the new treatment with those for all control patients. If this difference in outcomes is significant at a prespecified significance level (α_1), the new treatment is considered effective for the eligible population as a whole. Otherwise, a second stage test is performed using the significance threshold of α_2 = 0.05-α_1. The second-stage test involves finding the cut-point b* for which the difference in outcome of the treatment versus control (i.e., the treatment effect) is maximized when the comparison is restricted to patients with predictive index scores above that cut-point. The statistical significance of that maximized treatment effect is determined by generating the null distribution of the maximized treatment effect under random permutations of the treatment labels. If the maximized treatment effect is significant at level α_2 of this null distribution, the test treatment is considered effective for the subset of patients with a biomarker value above the cut-point at which the maximum treatment effect occurred. (Simon2010_Clinical trial designs for evaluating)	
			• […] a new adaptive enrichment design (AED)<br>• […] does not adaptively adjust the total sample size after stage 1 or the sample size in stage 2 (Diao2018)	For example, with the adaptive threshold design we assumed that a predictive biomarker score was prospectively defined in a randomized clinical trial comparing a new treatment T to a control C. The score is not used for restricting eligibility and no cut-point for the score is prospectively indicated. A fallback analysis begins as described above by comparing T to C for all randomized patients using a significance threshold α_1, say 0.03, less than the traditional 0.05. If the treatment effect is not significant at that level, then one finds the cut-point s* for the biomarker score which leads to the largest treatment effect in comparing T to C restricted to patients with score greater than s*. (Simon2010_Clinical trials for predictive)	

For peer review only

			The biomarker-adaptive threshold design (BATD) allows researchers to simultaneously study the efficacy of treatment in the overall group and to investigate the relationship between a hypothesized predictive biomarker and the treatment effect on the primary outcome.(Riddell2016)	The stage-1 analysis can be based on historical or pilot studies. The enrichment in stage 2 is expected to increase power for hypothesis testing using either data from stage 2 alone or combined data from both stages. The Cox regression model for survival endpoints is employed for the AED. However, the proposed methods can be easily generalized to any other applications where a regression model is mainly used for inference. Different criteria for determination of the biomarker cutpoint based on stage-1 data are proposed. (Diao2018)	
		**Molecular signature design**	It is a Phase III design which collects tissue samples from the entire population at the start of the trial and analyse them when the study is near completion. (Antoniou2016)	After the collection of tissue samples from the entire population, all patients are randomized to either the experimental treatment or the standard treatment. The methodology is similar to the Adaptive Signature design. (Antoniou2016)	This approach makes the comparison of the novel drug with the standard of care, but on a primary outcome measure which here is the overall survival using the significance level of 0.04. In case that the results show the effectiveness of an experimental treatment over the control arm, we claim the effectiveness of treatment in the overall population. Otherwise, an analysis is conducted for the identification and validation of the biomarker classifier (i.e., a combination of biomarkers), which gives the best primary outcome measure. A portion of subjects is used for the detection of a biomarker classifier and the remainder of patients for its validation. It is considered as a promising strategy without statistical considerations mentioned. (Antoniou2016)
		**Cross-validated adaptive signature design**	Similar to the Adaptive signature approach it is a Phase III frequentist trial design based on a fall back strategy in order to identify candidate biomarkers in the training set of the study and evaluate them in the validation set. (Antoniou2016)	The difference between Adaptive signature design and Cross-validated Adaptive Signature design is in terms of the methodology analysis. The former is composed of a split-sample approach, using approximately half of patients to develop the biomarker signature and the remainder of patients to validate it, whereas, the latter uses the K-fold cross validation procedure, i.e., there are K cross-validated training sets which are used to classify subjects in the corresponding K cross-validated validation sets. After the classification of all patients, we compare the experimental treatment versus the control treatment in the biomarker-positive patients (i.e., subgroup of classifier positive patients). The Cross-validated Adaptive Signature design may yield larger power but it faces the same challenges with its main design and also includes the multiplicity problem. (Antoniou2016)	

			[…] develop a predictive combination of biomarkers in a training set of the trial and consequently evaluate it in a test set (Tajik2013)<br>[…] extension of the adaptive signature design, which allows use of entire study population for signature development and validation. (Zhang2018_Advancing cancer)	Similar to the adaptive signature design, the initial null hypothesis is to test the benefit of the targeted therapy against the control is conducted in the overall population, which is conducted at a slightly lower significance level α_1 than the overall alpha α. The sensitive subset is determined by developing the classifier using the full population. It is done by the following steps:<br>(1) Test the initial null hypothesis of no treatment benefit in the overall population at α_1, which is a slightly lower significance level than the overall α. If this hypothesis is rejected, then the targeted therapy is declared superior to the control treatment for the overall population and analysis is completed. If the first hypothesis is not rejected, then the following steps for signature development and validation need to be performed.<br>(2) Split study population into "k" subsamples.<br>(3) One of the "k" subsamples is omitted to form a training subsample. Similar to the adaptive signature design, develop a model to predict the treatment difference between targeted therapy and control as a function of baseline covariates using training subsample. Apply the developed model to each subject not in this training subsample so as to classify patients as sensitive or nonsensitive.<br>(4) Repeat the same process leaving out a different sample from the "k" subsamples to form training subsample. After "k" iterations, every patient in the trial will be classified as sensitive or nonsensitive.<br>(5) Compare the treatment difference within the subgroup of patients classified as sensitive using a test statistic (T). Generate the null distribution of T by permuting the two treatments and repeating the entire "k" iterations of the cross-validation process. Perform the test at α -α_1. If the test is rejected, then the superiority is claimed for the targeted therapy in the sensitive subgroup. (Zhang2018_Advancing cancer)	
		**Generalized adaptive signature design**	It uses the training set of the trial to select among candidate biomarkers and to optimize cut-points; the selected biomarker is evaluated in the test set (Simon2010_Clinical trial designs for evaluating. In Table 1)	Firstly, candidate biomarkers are selected and the cut-off points are optimized using a training set and secondly, the chosen biomarkers are assessed in the validation set. (Antoniou2016)	

For peer review only

		**Adaptive signature design with subgroup plots**	Adaptive Signature design with Subgroup Plots is an extension of Adaptive Signature design which has been proposed in order to add flexibility. (Antoniou2016)	It uses tail-oriented or sliding window subgroup plots in order to identify a subset of patients which is most likely to respond to a particular experimental treatment after taking into account several cut-off points of the benefit score obtained by the subgroup plots. In this way it provides broader confidence intervals of the estimated treatment benefit. (Antoniou2016)	
**Outcome-based adaptive randomisation design**			It aims to test simultaneously both biomarkers and treatments while providing more patients with effective therapies according to their biomarker profiles. (Antoniou2016)	The process starts with the biomarker profile assessment of all eligible patients and then according to the profile of each individual, the study population will be assigned to the different biomarker groups. The trial begins with equal randomization so that each treatment by biomarker subgroup is composed of at least one individual with a known disease control status. Next, the trial continues with adaptive randomization of patients; this is achieved by using the Bayesian probit model to calculate the posterior disease control rate. After the posterior rate is found, we define the randomization rate as the posterior mean of the disease control rate of each treatment in each biomarker-defined subgroup. The adaptive randomization process continuous until the last individual is enrolled and can stop early only in case that all treatments are dropped due to inefficacy. (Antoniou2016)	A requirement of the Bayesian adaptive trial design is timely measuring and reporting of the study outcomes such that the randomization probability and the posterior probability for futility monitoring can be calculated accurately on the basis of the most recent data. (Liu2015)
				[…] an initial learning period within each treatment arm was used to subsequently randomize patients with increasing probability to the treatment showing the most benefit (in terms of 8-week disease control rate) within his or her marker group. (Renfro2016_Clinical trial designs incorporating)	
				Like the umbrella trial, a Bayesian marker-adaptive design may include multiple therapies and molecular subgroups. However, the efficacy of the drug is assessed in an ongoing manner through out the trial, allowing for biomarker-based adaptive randomization (i.e., changing of the randomization ratio(s) according to patient outcomes observed to date) and removal of ineffective therapies midtrial. The success of such a design requires a rapid and reliable endpoint and real-time access to all clinical and biologic data. (Renfro2017_Precision oncology)	• Requires strong predictive marker evidence<br>• Requires excellent assay performance<br>• Requires fast assay turn-around time (Renfro2016_Clinical trial designs incorporating)
			[…] Bayesian trials specifically designed to investigate differential biomarker-driven treatment effects (Renfro2016_Clinical trial designs incorporating)	Over the course of the trial, accumulating data are used to adjust the randomization probabilities to preferentially assign future patients to better-performing treatment arms. Typically, the first block of patients are	• Strong scientific rationale, and preliminary evidence for the molecular marker-drug pairing<br>• Reliable assay, with rapid turn-around times<br>• Short term, reliable endpoint to make the

For peer review only

				randomized to each arm in equal proportion and randomization probabilities for subsequent blocks are calculated based on information accumulated prior to starting the block. (Talisa2018)	adaptation meaningful<br>• Sufficient infrastructure set up and real time data availability (Renfro2017_Precision oncology)
				These proposals generally start with a small sample burn-in period followed by assigning the next dose based on accumulating short term responses or outcomes or the immediately previous cohort response until the pre-specified maximum number of patients randomized is reached. In addition, the learning stage may employ longitudinal models linking the intermediate efficacy biomarker with clinical outcome, dose's response models, and/or clinical outcome dropout models. (Wang2011)	[…] one must define the decision rules for adaptation upfront of study initiation, monitor the randomisation weights to avoid instable estimates, account for time dependency of the outcome (if necessary) and has to rely on a short-time outcome. (Kesselmeier2019)
		**Bayesian covariate adjusted response-adaptive randomisation**	This strategy which combines a Bayesian, an adaptive and biomarker classification approach aims to match patients with the most efficacious treatments by utilizing patient's biomarker information becoming available during the conduct of the clinical trial. (Antoniou2016)	The general procedure of this approach is composed of four steps according to Eickhoff et al. (2010): (i) randomly assign the first n^*>=J^*(K+1) patients to the different treatment arms where J the number of different treatment groups and K the number of biomarkers. At least one response should be observed in each of the different treatment groups before moving to the Bayesian response adaptive randomization; (ii) after each new individual has been enrolled in the study, predictive biomarker-defined groups are determined by utilizing a partial least squares logistic regression strategy (PLSLR) which can predict whether the patient can benefit from the treatment. The biomarker status is determined before the randomization; (iii) after the establishment of the biomarker status and biomarker-defined groups of each new individual, the individual is then randomly assigned into one of the treatment arms using a BCARA randomization; (iv) according to the results of the BCARA randomization the trial either stops or continues based on decision rules proposed by Eickhoff et al. (2010) [53]. The Bayesian covariate adjusted response-adaptive trial design has the ability to identify the biomarker-defined groups likely to respond to a treatment but it does not control the Type I error and in order to ensure that the identified result is true, a Phase III study should be conducted. (Antoniou2016)	
**Adaptive enrichment**	**Adaptive threshold sample-**		It is a two-stage design in a Phase III setting to adaptively modify accrual in order to broaden the targeted	At the interim analysis stage, the treatment effect of a sample of patients (n1) from the biomarker-positive subset is estimated. If an	

For peer review only

	**enrichment design**		patient population (Antoniou2016)	improvement is seen in the experimental treatment arm which is greater than a pre-specified threshold value (i.e. the estimated treatment difference between the novel treatment arm and the control treatment arm for this subpopulation is greater than a threshold value c divided by the square root of the aforementioned sample size n1) the trial continues with accrual of patients from the entire biomarker-positive subgroup and additional patients are also accrued from the biomarker-negative subpopulation; otherwise the trial is stopped for futility. At the end of the trial, the treatment effect is estimated for all subpopulations. Researchers should choose the sample size n1 so that a persuasive result can be reached when the first stage of the trial is completed. (Antoniou2016)<br><br>After an interim analysis separating two stages of patient enrollment, such a trial may stop for futility or efficacy, continue on as a randomized trial, or switch toward direct assignment of patients to the experimental treatment based on initially promising, but not definitive, results. (Renfro2016_Clinical trial designs incorporating)<br><br>[…] starts with accruing only biomarker-positive patients during the initial stage of the trial. At the end of the first stage, an interim analysis is conducted comparing the outcome of the experimental versus control treatment in biomarker-positives. If the results are not promising for the new treatment, accrual stops and no treatment benefit is claimed. Otherwise, accrual continues with recruiting unselected population. This design is a combination of an enrichment and a traditional flow, conditional on the result of the interim analysis. (Tajik2013)<br><br>The design consists of two stages, where in stage 1, patients are recruited in the full population. Stage 1 outcome data are then used to perform interim analysis to decide whether the trial continues to stage 2 with the full population or a subpopulation. The subpopulation is defined based on one of the candidate threshold values of a numerical predictive biomarker. The final confirmatory analysis uses data from both stages. (Kimani2018)	
	**Adaptive patient enrichment design**		Adaptive enrichment designs offer the potential to enrich for patients with a particular molecular feature that is predictive of benefit for the test treatment based on	A pre-planned total sample size with futility stopping is considered for this two-stage adaptive design. The trial assesses the treatment effect both in the entire population and in the biomarker-positive population.	One forewarning to apply the adaptive enrichment design is that the end point for interim analysis should be properly chosen, in that the end point should be measurable and that sufficient data are attainable to give investigators reliable guidance to

For peer review only

			accumulating evidence from the trial. (Mandrekar2015)	(Antoniou2016)	move forward into the next stage. (Lin2015)
				In this design, all of the eligible subjects are recruited in the first stage, followed by an interim analysis to determine the study design between enrichment design and all-comer design. The sample size, end points, randomization ratio or enrichment hypothesis may also be adjusted using interim data before moving forward to Stage 2. Bayesian methods are proposed for the adjustment of randomization scheme using interim data. (Lin2015)	• Requires strong predictive marker evidence<br>• Requires excellent assay performance<br>• Requires fast assay turn-around time<br>• Requires moderate to high marker prevalence<br><br>(Renfro2016_Clinical trial designs incorporating)
				Patients are screened with the diagnostic test and those who are considered "test-positive" are eligible for the clinical trial. Eligible patients are randomized to receive either the test drug or an appropriate control regimen. In some cases, the randomization may be between the test drug and standard chemotherapy, or between standard chemotherapy alone versus standard chemotherapy plus the test drug. When there is no standard chemotherapy, the randomization may be between the test drug and best supportive care. (Mandrekar2015)	Statistically, a challenge of using adaptive accrual design relates to type I error control. There are several sources that could contribute to potential type I error inflation, including the potential enrichment of the accrual population with sample size modification as well as the adaptive selection of the hypotheses that to be tested at the final stage. Appropriate statistical correction needs to be applied to ensure type I error rate is controlled for adaptive accrual design. (Zhang2018_Advancing cancer)
				The adaptive enrichment design initially randomizes an unselected patient population to experimental versus control treatment, and if the experimental treatment effect reaches a futility threshold in the marker negative group at an interim analysis, accrual of marker-negative patients is terminated and the remaining sample size re-allocated to marker-positive patients. In that case, the primary hypothesis tested at the trial's conclusions is the treatment effect in the marker-positive subgroup. Otherwise, if futility is not reached in the marker-negative group at an interim analysis, the trial continues unselected and performs both overall and subgroup-specific tests of treatment benefit at the final analysis time point with trial-wise type I error control. (Renfro2016_Clinical trial designs incorporating)	
			[…] biomarker-based clinical trial designs with allowed mid-trial adaptation based on the results of interim analyses. (Renfro2016_Clinical trial designs incorporating)	At the interim analysis after stage 1, a decision is made about enrollment in stage 2, based on the stage 1 data. The 3 choices are to enroll the combined population, only subpopulation 1, or to stop all enrollment. Adaptive enrichment designs with >2 stages involve such choices at the interim analysis after each stage. (Rosenblum2017)	

For peer review only

			[…] initially randomizes an unselected patient population to experimental versus control treatment, and if the experimental treatment effect reaches a futility threshold in the marker-negative group at an interim analysis, accrual of marker-negative patients is terminated and the remaining sample size re-allocated to marker-positive patients (Renfro2017_Precision oncology)	[…] the trial begins with a biomarker-stratified first stage in which it accrues both biomarker-positive and -negative patients. If the results of an interim analysis comparing the outcome of the experimental versus control treatment in biomarker negatives are not promising, accrual to biomarker-negative subgroup is terminated and the second stage continues as an enrichment trial in biomarker-positive patients until the planned total sample size is reached. (Tajik2013)	
			Designs with prespecified rules for modifying the enrollment criteria based on data accrued in an ongoing trial […] (Rosenblum2017)	An interim look will be prospectively planned in a two-stage adaptive accrual design, and the adaptations will primarily be in two aspects based on the interim results: 1) The patient population to enroll at the second stage of the trial (overall or only g+); 2) The test population(s) at the final analysis (full population or marker+ population or both full and marker+ as co-primary population). (Zhang2018_Advancing cancer)	
			Adaptive designs can also be considered in order to bring the effective treatment to the right subset of patients sooner. (Zhang2018_Advancing cancer)		
			[…] two-stage adaptive enrichment design (AED) that retains some of the flexibility of the Simon design and yields a subgroup for treatment indication together with a specific test of treatment efficacy for the chosen subgroup. Like the Simon design, the proposed design does not require predefined subgroups; it allows a subgroup to be selected at an interim analysis on the basis of a prespecified collection of baseline covariates. We do require that the algorithm for subgroup selection be prespecified. The selected subgroup will be used for patient enrollment in the second stage and eventually for treatment indication. The treatment effect in the selected subgroup can be estimated using a weighted average of separate estimates from the 2 stages. It is straightforward to obtain a treatment effect estimate from the second-stage data. However, treatment effect estimation in the first stage is subject to a resubstitution bias due to the fact that the same set of data is used to select a subgroup and estimate the treatment effect in the selected		

For peer review only

			subgroup. We consider the use of cross-validation and bootstrap methods to correct for the resubstitution bias. (Zhang2018_Treatment evaluation)		
		**Modified Bayesian version of the two-stage design**	It is a Phase III Bayesian two-stage design proposed by Karuri and Simon (2012) for the evaluation of both treatment and biomarker. (Antoniou2016)<br><br>A Bayesian version of the adaptive enrichment design that allows for formal specification of prior confidence in a biomarker's predictive ability […] (Renfro2016_Clinical trial designs incorporating)		
		**Bayesian hierarchical model for response adaptive randomised design**		the model incorporates a continuous monitoring for futility and a final analysis of efficacy that are conditioned on the integral biomarkers (Barry2015)	
		**Bayesian adaptive patient enrolment restriction (BAPER) approach**	Consider a two-arm randomized phase 2 clinical trial in which an experimental treatment is compared with a control treatment based on a primary endpoint of time-to-event data (e.g., PFS), and there exists a single continuous biomarker that is prospectively hypothesized to be predictive. It is assumed that the continuous biomarkers for all patients are available before randomization and that a higher value of the biomarker indicates greater improvement of efficacy if the biomarker is truly predictive. (Ohwada2016)	The objective of the trial is to identify a sensitive patient population **and make a final decision for a subsequent phase 3 trial** (i.e., no-go, go with entire population, or go with subpopulation) based on a pre-defined target efficacy level (e.g., HRD0.6), which may be provided by physicians or a clinical study team taking its clinical relevance into consideration. Two or three interim analyses are planned to narrow down the patient population to be enrolled in the next cohort of the trial, as well as to decide early termination due to futility or efficacy.<br><br>We apply a four-parameter change-point model to the relationship between the single continuous biomarker and HR and calculate the posterior distribution of the cutoff parameter of the biomarker, thus identifying the subpopulation that truly exhibits the target HR or a more efficacious HR. Using the posterior distribution, we identify the patients who are unlikely to reach the target HR and stop enrollment of such patients at the interim analysis. In addition to our proposed restriction on patient enrollment, we also incorporate criteria for futility and efficacy stopping at the interim analysis; finally, we make the following decision for the next step: no-go	

For peer review only

				(futility), go for the next study with the entire population, or go for the next study with the sensitive subpopulation. (Ohwada2016)	
		**Adaptive design for population selection using correlated time to event endpoints**	We extend the previous methods (Brannath et al., 2009; Jenkins et al., 2011) in two aspects. First, the interim analysis is conducted by incorporating information on progression-free survival (PFS) as well as overall survival (OS). Second, we consider a scenario in which OS is calculated based on PPS, if the progression is observed before death. (Uozumi2017)		
		**Biomarker stratified with a subgroup-focused sequential design**	[…] allows both sequential assessment across marker-defined subgroups and adaptive subgroup selection, while retaining an assessment using the entire patient cohort at the final analysis stage, possibly using established marker-based multiple testing procedures (Matsui2018)	We assume a reliable marker hypothesis where the treatment is more effective in the marker-positive than in the marker-negative patients. One-sided statistical tests are used. […] The proposed design approach is summarized in Fig. 1. This can be viewed as concurrent subgroup-focused trials with a futility stopping rule in the marker-negative subgroup and a superiority stopping rule in the marker-positive subgroup. In case I, both boundaries are crossed, and the trial is stopped with a conclusion of efficacy in the marker-positive subgroup. In case II, only the superiority boundary is crossed, and there is sequential testing in the marker-negative subgroup. In cases III and IV, the marker-positive subgroup or the overall population is adaptively selected for the final analysis depending on whether the futility boundary is crossed in the marker negatives. In case IV, the subgroup data are combined for the final analysis. Thus, the possible complexities in performing an overall test at the final analysis in case of early stopping in some subgroup is avoided by restricting the implementation of the analysis using all patient data to only the case with no early stopping in both subgroups. Extension to multiple interim looks is possible, but we suppose a single interim analysis within subgroups for ease of presentation and practical application.<br><br>The marker-positive cohort is designed as if it were an enrichment trial. This is sized for large, but slightly conservative effects for the new treatment. The marker-negative cohort is designed as if it were a second trial in the sequential enrichment approach. This is	The interim analysis for superiority in the marker-positive patients, deemed most likely to benefit fromthe treatment, is to detect substantially large treatment effects and to quickly deliver the treatment to such patients. Although futility stopping rules can also be introduced in this subgroup, we propose no specification of such rules and no adjustment on the final analysis. In any case, futility stopping for marker positives would lead to the termination of the trial under the marker hypothesis. On the other hand, for marker-negative patients, a futility stopping rule would be warranted from an ethical perspective due to presumably limited treatment efficacy in marker negatives under the marker hypothesis. We propose a monitoring plan that accounts for the two possible errors: (i) futility stopping even when treatment has, in truth, a minimum effect size of clinical importance and (ii) continuing the trial for the marker negatives even when there is no treatment efficacy. In addition, we could introduce a superiority stopping rule, but we do not consider this option because large treatment effects are generally implausible for marker negatives under the marker hypothesis. When there is not sufficient evidence for early stopping in both subgroups (case IV in Fig. 1), an overall test is a simple but most effective choice in detecting an average treatment effect in the overall population at the final analysis. Alternatively, when the marker hypothesis is deemed strong, hierarchical tests may be used, such as a fixed-sequence procedure that first tests treatment efficacy in the marker positives, followed by testing in the marker negatives if the first test is significant. Otherwise, a split-alpha procedure that allocates the alpha to be spent between a test in the markerpositive subgroup and one in the overall

For peer review only

				because the chance to evaluate this cohort solely when the treatment effect is significant in marker-positive patients is also embedded in our approach, not sequentially, but concurrently. (Matsui2018)	population may be a reasonable choice. The significance levels of all statistical tests are determined to preserve a study-wise alpha level of 0.025 based on the joint null distribution of the test statistics for the marker-positive and marker-negative subgroups and the overall population across different analysis stages, that is, the global null hypothesis. We do not consider an alpha control under another possible null hypothesis, where the treatment is efficacious in marker positives, but not in marker negatives. (Matsui2018)
		**Stratified adaptive design**	It is alternative approach to dealing with stratification in a phase II setting and aims to demonstrate whether an experimental treatment (a control arm is not included, thus it's about a single arm approach) is beneficial for at least one biomarker-defined subgroup rather than the entire study population. (Antoniou2016)	The first stage is consisted of an interim analysis where the response rate is estimated in the biomarker positive and biomarker negative subgroups separately. The trial then enters a second stage and depending on the results of the interim assessment, accrual continues either from the entire patient population if there is treatment efficacy of both biomarker-defined subgroups, or from one of the distinct biomarker subpopulations only in which treatment efficacy has been observed. (Antoniou2016)	It is alternative approach to dealing with stratification in a phase II setting and aims to demonstrate whether an experimental treatment (a control arm is not included, thus it's about a single arm approach) is beneficial for at least one biomarker-defined subgroup rather than the entire study population. (Antoniou2016)
			Tournoux et al. proposed a stratified adaptive Fleming two-stage design not requiring any assumption prioritizing the two pre-defined subgroups. (Cabarrou2018)	It is assumed that the ratio between the number of patients in the biomarker negative and biomarker-positive subgroups is constant and is defined by ω=N+ / N-. This design provides stopping rules for both activity and futility at the end of the first or second stage. Heterogeneity between the two subgroups is also tested at each stage at level which can be set between 0 and 1. (Cabarrou2018)	
**Adaptive parallel Simon two-stage design**			The design aims to test a novel treatment which possibly has a different treatment effect in the biomarker-positive versus the biomarker-negative subgroups. (Antoniou2016)	The design begins with two parallel phase II studies. During the first stage, two separate studies are performed in the biomarker-positive and biomarker-negative subgroups. Next, depending on the interim results of the first stage, the trial either stops or continues into a second stage with the enrollment from either the entire patient population (unselected patients) or from the biomarker-positive subpopulation only (selected patients). If a preliminary efficacy is observed during the first stage of the study for the experimental treatment in both the biomarker-positive and biomarker-negative subset, then additional patients from the general patient population will be enrolled in the second stage; if the interim result during the first stage of the trial shows that the efficacy is limited to the biomarker-positive subjects, then the recruitment of additional biomarker-positive patients only continues during the second stage. (Antoniou2016)	The approach assumes that there is a sound scientific rationale as to why the biomarker may potentially affect response rate. Further, it is also assumed that there is reasonable knowledge of the prevalence of the marker and that identification of subjects as marker positive or negative is well established (Jones2007)

				If preliminary efficacy based upon the first stage suggests that the drug is active in both marker positive and marker negative patients then subsequent enrollment will be unrestricted and an additional N^{un} subjects are to be enrolled during the second stage. At the end of the second stage a total of N^+ and N^-, marker positive and marker negative subjects, respectively, will have been enrolled, and of these subjects there will be a total of X_T^+ and X_T^- responders. In this setting N^+ and $N^-$are unknown a priori but based upon the known marker prevalence a reasonable value can be postulated. If based on the outcome of the first stage there is preliminary evidence that efficacy is restricted to the marker positive subgroup then enrollment of N_2^+ additional marker positive subjects continues during the second stage for a total enrollment of $N^+ = N_1^+ + N_2^+$marker positive subjects. (Jones2007)	
		**Parashar design**	An extension of the Jones design was proposed by Parashar et al. by adding go-decision rules in either the unselected population or the biomarker-positive subgroup at interim analysis. (Cabarrou2018)	As for the Jones design, it is necessary to anticipate some type of hierarchy between the two subgroups before beginning the study, and it is assumed that the response rate will be higher in the biomarker-positive than in the biomarker-negative subgroup. The study begins with the inclusion of N_1^- and N_2^+ patients, respectively, in biomarker-negative and biomarker-positive subgroups. (Cabarrou2018)	
**Multi-arm multi-stage design**			It has the ability to simultaneously compare multiple experimental treatments<br>with the standard treatment in order to achieve more reliable results in less time as compared with separate Phase II trials to assess each novel treatment individually. (Antoniou2016)	The first stage of the trial (the Phase II stage) involves randomization within one of two arms which simultaneously compare two experimental treatments with the standard of care (control) using an intermediate outcome measure (e.g. progression free survival). The arm within which a patient is included depends on their biomarker status, for example patients positive for biomarker 1 may be randomized in arm 1 to	

For peer review only

			Where there is more than one clinically important question to be addressed (which is commonly the case), a multi-arm trial approach can simultaneously and systematically test each of these approaches against the current standard of care (the control arm). (Kaplan2015)	either standard of care or experimental treatment 1 whilst patients positive for biomarker 2 may be randomized in arm 2 to either standard of care or experimental treatment 2. At the end of this first stage, an interim analysis is undertaken in each arm, comparing the experimental treatment with standard of care. Depending on the outcome of the interim analysis, accrual of patients either continues within an arm to the second stage of the trial or the accrual of additional patients stops within that arm. (Antoniou2016)	
		**Two-stage adaptive seamless design**	It uses the MAMS approach combining two separate studies into one single study and uses interim monitoring as well as multi-arm design features. (Antoniou2016)	the general procedure of this Phase II/III strategy is presented by Brannath et al. (2009) as follows: When half of individuals are recruited in the study, an interim analysis is performed in order to decide whether to accept or not a biomarker-defined subpopulation identified in a separate exploratory study. At this interim stage, a decision is also made about whether to continue accruing patients from the aforementioned biomarker-defined subset or from the entire study population. If the first case occurs, the treatment effect is assessed only in this biomarker subpopulation and if the second case happens, the treatment effect is tested in the entire population and biomarker-defined subgroup at the same time. In case that there is no identified biomarker-defined subpopulation from the separate exploratory study, the trial continues in the overall population using a classical group sequential design. An extension of the above approach by Brannath et al. (2009) is proposed by Jenkins et al. (2011) which can result in the rapid approval of novel treatments to the most appropriate individuals who are likely to benefit from the new drug. During the Phase II trial an interim analysis is conducted using a short-term intermediate outcome measure (i.e., survival endpoint) in order to select the population (either the entire population or the biomarker-positive patients) which will be used in the Phase III study with a long–term endpoint. Mehta et al. (2014) proposed an alternative seamless approach for subgroup selection in time-to-event-data for situations where there is no a priory assumption that a biomarker is predictive of treatment efficacy; consequently their design tests whether there is treatment effect in both biomarker-negative and biomarker-positive subpopulation separately instead of	According to Scher et al. (2011), formulas for sample size calculation/allocation are proposed in situations where the study endpoints are continuous, discrete, and contain time-to-event data supposing the availability of a well-established relationship between the study endpoints at different stages, and that the study objectives at different stages are the same. Ang et al. (2010) have stated that even in case that the trial stops early, a Phase III infrastructure should be developed. Such strategies have been proposed by Ellenberg and Eisenberger (1985) and Inoue et al. (2002) for evaluating the possibility to stop early or to continue to the confirmatory phase III repeatedly during the explanatory phase. (Antoniou2016)

				testing the null hypothesis of no treatment effect in the entire study population and in biomarker-positive subset. (Antoniou2016)	
			[…] combine the learning stage of Phase II and confirmatory stage of Phase III (Lin2015)	In the beginning of Phase II, subjects are randomized into the treatment arms of A, B, combined therapy of A and B, or control. An interim analysis is then performed to determine which active arm should be dropped. In the confirmatory stage of Phase III study, the treatment groups with only one active arm and control arm will be investigated. (Lin2015)	
			Seamless designs consolidate multiple phases into a single protocol that is designed, approved, and executed as a single trial. (Talisa2018)	After an interim analysis between the phases, which uses the shorter-term endpoint, the trial can either continue to phase III in the co-primary overall and subgroup populations, continue in the subgroup only, continue in the full population without consideration of the subgroup, or stop for futility. (Renfro2016_Clinical trial designs incorporating)	
				Initially, patients are randomized between multiple new therapies and a control. At the end of the Phase II stage, an intermediate (early) end point is employed to make a decision as to whether to continue the trial to the Phase III stage and, if so, to select the most promising experimental arms for evaluation of the definitive clinical outcome. (Freidlin2010_Biomarker-adaptive clinical trial designs)	
		**Bayesian subgroup based adaptive design (SUBA)**	[…] designs that simultaneously search for prognostic subgroups and allocate patients adaptively to the best subgroup-specific treatments throughout the course of the trial. (Xu2014)	If one treatment is inferior to all other treatments, then that treatment should be dropped from the trial. If there is only one treatment left after dropping inferior treatments, then the trial should be stopped early due to the ethical and logistics reasons. The SUBA design starts a trial with a run-in phase during which patients are equally randomized to treatments. After the initial run-in, we continuously monitor the trial until either the trial is stopped early based on a stopping rule, or the trial is stopped after reaching a prespecified maximum sample size N. (Xu2014)	

For peer review only

			SUBA applies a Bayesian random partition model to search for a suitable partition (clustering) of the patient space based on selected variables. (Simon2018)	SUBA can accommodate 3 independent variables, which are chosen a priori based on the specific project (described below). For each of the patients enrolled in phase 1, SUBA uses information on these 3 factors, their treatment assignment and their outcome. Based on the partition, SUBA calculates the posterior predictive probability that a future patient with specific variable values will respond to a particular treatment if the patient is assigned to the treatment. This treatmentspecific posterior predictive probability is then used to randomize the patient. If the posterior predictive probability is larger for one treatment, the patient will have a larger randomization probability to be assigned to that treatment. In other words, patients are assigned adaptively to treatments based on predictive response. The posterior predictive probability for each future patient is continuously updated when new outcomes are observed from previous patients. This allows the trial to continue the learning until the end, potentially providing better benefits for patients in the trial by giving them a larger chance to be randomized to more desirable treatments. (Simon2018)	
		**Group sequential design**	This strategy aims to find the most beneficial treatment for future patients based on their biomarker profiles, with a guaranteed probability of correct selection. (Antoniou2016)	According to an interim data analysis, sequential decisions about whether to continue the study or not, are taken. It is considered a simple approach where selection of cut-off points is not required before the conduct of the first interim analysis. (Antoniou2016)	
**Tandem two stage design**			It is composed of 2 optimal trials in a Phase II settings. (Antoniou2016)	In this design, a predefined biomarker is assumed. In the first stage of the trial, patients from the entire population enter the trial irrespective of their biomarker status. An interim analysis is then undertaken and if a sufficient number of events (defined in terms of clinical benefit rate or response rate) have been observed during the first stage, the study proceeds to a second stage whereby further patients are accrued from the unselected population to establish the benefit rate more precisely in unselected patients. However, if an insufficient number of events have been observed during the first stage, rather than stopping accrual for futility, a second trial commences whereby its first stage involves continued accrual of biomarker positive patients only. An interim analysis is then conducted and if a sufficient number of events have been	The sample size for this approach is calculated with the same rules as a classic two-stage or Bayesian phase II design. (Antoniou2016)

For peer review only

				occurred, this second trial continues into a second stage of biomarker positive patient accrual. Otherwise, if an insufficient number of events have occurred, the predefined biomarker is rejected. (Antoniou2016)	
**Platform design**			To study multiple-targeted therapies in the context of a single disease in a perpetual manner, with therapies allowed to enter or leave the platform on the basis of a decision algorithm (Heerspink2018_New clinical trial designs)	First, a shared master protocol is used for common elements of the multiple individual trials within the platform with relatively subtle trial design differences due to unique individual drug characteristics reflected in study-specific appendices, enabling sharing of clinical trial documents and procedures among trials. This facilitates clinically consistent trial conduct and increased efficiency. Second, the platform approach commonly involves some form of adaptive design to assign patients to the most promising drugs on the basis of new data accrued during the trial. In addition, the platform trial is not static, but it is flexible, which means that new promising drugs can enter the platform, while other drugs can be dropped due to lack of efficacy or adverse events. Declaring superiority or futility can be assessed continuously on the basis of data as they are accrued during the trial and is another adaptive design element (Heerspink2018_Trial design innovations)	
				[…] patients are assigned to a treatment arm based on concentration levels of a set of predictive markers for the available treatment options. Markers and renal function parameters are used for patient monitoring and identification of responders who remain in the assigned treatment arm, whereas nonresponses are shifted to the next-best suitable treatment based on marker profiles. (Perco2019)	
			[…] in platform trials (or ˜standing trials") patients with a specific tumor type are randomized to a common control arm or one of the several experimental arms that enter and exit the trial after interim analyses aimed to evaluate the efficacy or futility of each targeted treatment through Bayesian method. (Leonetti2019)	Platform trials are often Bayesian in nature, utilizing Bayesian decision rules based on posterior or posterior predictive probabilities to eliminate or graduate treatments within certain cohorts. (Renfro2018_Definitions and statistical properties)	

For peer review only

			[…] designs that evaluate multiple treatments simultaneously […] (Mazzarella2020)	Initially the treatments are randomized with equal weights to the patients of a stratum. As data accumulates, the randomization weights change to favor assignment of drugs with higher within-stratum response rates. The endpoint used must be observed early enough to enable adaption of randomization weights. (Simon2017_Critical review)	
			Platform trials, also referred to as multi-arm, multi-stage (MAMS) design trials, are trials that evaluate several interventions against a common control group and can be perpetual. This design has pre-specified adaptation rules to allow dropping of Ineffective intervention(s) and flexibility of adding new intervention(s) during the trial. (Park2019)	In a platform trial, the feedback loop involving collecting data, updating the Bayesian statistical model and updating RAR weights is modified to enable new arms to be added, and old arms to either be dropped or "graduate" to the next phase of testing (Talisa2018)	
			Another type of master protocol described in the literature is the platform trial (or "standing trial"), a generic term for a randomized design with a common control arm and many different experimental arms that enter and exit the trial as futility or efficacy are demonstrated, often according to Bayesian decision rules. (Renfro2017_Statistical controversies)	In both umbrella and platform trials, each arm is typically enriched with a biomarker and patients are enrolled and assigned to a cohort based on their biomarker status. Platform trials may be distinguished from umbrella studies in that they are thought to incorporate more adaptations as responses are observed, patients are algorithmically allocated to specific treatment arms according to the best match between treatment effect and their tumor type. Experimental drugs drop out for lack of efficacy or they can "graduate" for efficacy testing depending on the observed response. Randomization is adapted such that the number of patients needed to determine efficacy across biomarker groups is minimized (Cecchini2019)	
			Lastly, a platform trial may be generally defined as a type of master protocol in which sub-trials continually enter and exit, where the latter may occur due to futility or due to graduation of a marker-treatment combination to further study. (Renfro2018_Definitions and statistical properties)		

For peer review only

			A platform trial is a single histology randomized phase II clinical trial involving multiple biomarkers and multiple drugs. Rather than assuming that we know which drug is appropriate for which biomarker stratum, randomization among drugs is used in the platform trial. (Simon2017_Critical review)		
			[…] the adaptive platform trial is capable of being a platform for testing experimental treatments in a perpetual manner via a common master protocol, by dropping treatments lacking efficiency and adding new treatments going into the future. (Talisa2018)		
			Other trial designs include platform trials, which use a single analytic technique, such as NGS (next generation sequencing), to identify genomic or other biomarkers in tumors with multiple histologies; (Tsimberiou2020)		
			A parallel group design with a shared control evaluates two or more investigational treatment arms relative to a control arm in the same tumour type in a single clinical trial. (Verweij2019)		
			Platform trials randomize patients to different cohorts and take umbrella studies a step further by following algorithms to adapt and add new therapies or drop existing therapies from an ongoing study. (Cecchini2019)		
			[…] multi-arm because many treatment approaches can be tested simultaneously; multi-stage because prespecified interim analyses can be used to stop recruitment early to arms showing insufficient evidence of activity. (Gilson2017)		

For peer review only

			A platform trial is defined as a trial using a single master protocol and research infrastructure to simultaneously evaluate multiple interventions and/or disease subpopulations in multiple substudies. Platform trials gain efficiencies from shared control groups, adaptive borrowing of information from similar groups of patients, and shared infrastructure and governance. (Semler2020)<br>[…] study multiple targeted therapies in the context of a single disease in a perpetual manner, with therapies allowed to enter or leave the platform on the basis of a decision algorithm. (Alexander2019)		
	**Open adaptive platform**		The trial is "open" with respect to adding new treatments to replace ineffective treatments during the trial. (Saville2016)		
		**Randomised, embedded multifactorial adaptive platform (REMAP)**	Randomized, embedded, multifactorial adaptive platform (REMAP) trials utilize all of the features of a perpetual adaptive platform trials like I-SPY 2 or GBM-AGILE, the key distinction being that a REMAP trial is executed directly within clinical practice through the electronic medical record. (Talisa2018)		
		**Bayesian Adaptive Platform Trial**		As the trial progresses, randomization probabilities adapt on the basis of accumulating results using Bayesian estimation of the biomarker-specific probability of treatment impact on progression-free survival. Treatment arms may drop because of low probability of treatment impact on overall survival, and new arms may be added. (Alexander2019)	[…] uses biomarker subgroup-specific randomization probabilities to allow data generated during the trial to drive the biomarker specificity of arm assignments. (Alexander2019)
	**Closed platform**		The trial is a ''closed'' platform trial, meaning no additional treatments are added beyond those included at the start of the trial. (Saville2016)		
**Basket design**			Evaluates the effect of a particular targeted therapy on a particular genetic or molecular aberration across cancer organ types. Variant	Molecular profiling-based targeted therapies are prescribed to treat patients with advanced metastatic solid tumours that are usually incurable or not controlled by standard	[…] basket trials should be stratified by histology, taking into consideration the reported frequencies of the genomic event. (Garralda2019)

For peer review only - http://bmjopen.bmj.com/site/about/guidelines.xhtml

			of indication finder but the therapy is not evaluated for its off-target effects. (Berry2015)	treatments. NCI-MPACT randomly assigns patients with a mutation in a specific genetic pathway to either a targeted therapy for that pathway or a treatment not known to be pathway specific. (Gómez-López2017)	
			In this framework, patients with different tumor histologies but who harbor the same molecular aberration receive a matched targeted in the context of expansion cohorts of a Phase 1 trial or as a separate Phase 2 trial, with efficacy as the primary endpoint. (Dienstmann2015)		
			This is an innovative, histology agnostic trial design, where patients with tumours of different histologies can be enroled in the study protocol on the basis of the presence of a commonly shared molecular aberration. (Fadoukhair2016)		[…] the lower the prevalence of the biomarker, the larger the effect size needs to be for the trial to be meaningful (Janiaud2019)
			Basket trials include patients with different tumour types with a common molecular alteration who are treated with the same matched therapy (Garralda2019)	Commonly, basket trials are early stage, single-arm, phase II, proof-of-concept trials where in each basket or cohort is itself a single-arm trial studying a preliminary target-response hypothesis. Such cohorts are generally small (say, 20-30 patients) and only powered to detect strong signals of activity meant to motivate further study in a randomized context, though toxicity is often a key secondary endpoint in sub-studies where drug tolerability is not yet well understood. Each arm may further be constructed as a single-stage, two-stage, or multi-stage design, and futility-stopping rules may be incorporated. (Renfro2018_Definitions and statistical properties)	From a statistical perspective, the efficiency of basket trials comes from pulling data across all tumor subgroups to estimate the treatment effect. However, this pooled approach only works well when response to the therapy is relatively homogeneous across all tumor subgroups. Heterogeneous responses across tumor subgroups may lead to potential bias and/or inflation of the false-positive rates. A new calibrated Bayesian hierarchical model has recently been proposed to better control the type I error rate in basket trials. (Le-Rademacher2018)
			To study a single-targeted therapy in the context of multiple disease or disease subtypes (Heerspink2018_New clinical trial designs)	Patients are assigned a regimen that is expected to be active for tumors containing that alteration. Often this expectation is based on knowledge of the target of the drug and its role in the progression of the disease as well as previous approval of the drug, or a similar drug, for patients with the same genomic alteration in some specified histology. In this case, the basket trial is a phase II screening trial for off-lable use of the drug in patients with the same genomic alterations for which it was approved. (Simon2017_Critical review)	

			The distinguishable feature of basket trials is their inclusion of multiple tumor types and cancer histologies, and the term histology independentâ is often used to characterize this feature. The different tumor types can express the same mutation or different ones and are targeted by either one unique therapy or biomarker-specific therapies. (Janiaud2019)	Eligibility depends on the presence in the tumor of a specified type of genomic alteration. A few multidrug basket trials have involved randomization to a test drug that targets a mutation in the patient's tumor or to a control drug. The use of randomization in a multidrug basket trial permits the trial to test the general policy of trying to match the drug to the genomics of the tumor. (Simon2016_Genomic alteration)	• Requires strong predictive marker evidence<br>• Requires excellent assay performance<br>• Requires fast assay turn-around time (Renfro2016_Clinical trial designs incorporating)
			Basket trial design is a novel biomarker-based design that includes patients with different histologic or tumor subgroups who carry the same molecular aberrations. Each of these histologic/tumor subgroups, called a "basket", forms a substudy of the overall trial. The substudies within a basket trial can have the same type of design or different designs or a combination of both. The goal of a basket trial design is to efficiently identify effective treatment targeting a particular molecular aberration which is associated with multiple tumor types. (Le-Rademacher2018)	For each drug studied in a basket design, all of the patients generally share a common mutation, but have different primary disease sites. The standard phase II designs used for most basket clinical trials ignore this heterogeneity and pool all patients containing the same actionable mutations for analysis. (Simon2018_New designs for basket clinical trials)	From a statistical perspective, the efficiency of basket trials comes from pulling data across all tumor subgroups to estimate the treatment effect. However, this pooled approach only works well when response to the therapy is relatively homogeneous across all tumor subgroups. Heterogeneous responses across tumor subgroups may lead to potential bias and/or inflation of the false-positive rates. A new calibrated Bayesian hierarchical model has recently been proposed to better control the type I error rate in basket trials. (Le-Rademacher2018)
			Basket trials assess the effectiveness of a candidate drug based on the mechanism rather than the underlying cancer type. (Joshi2018)	In this design, individual histologic subtypes (indications) are grouped together each with its own control group. A shared control group may be used for indications with a common standard of care. Single arm designs using a concurrent registry control may be considered.<br>Concurrent registries control for disease stage migration (the process by which progressively improved sensitivity of diagnostic techniques translates over time into patients with less disease burden being assigned to a given disease stage) and for progressive improvements in outcome due to improved supportive care, but do not control for patient selection (the ability and tendency of physicians to select patients who will do well, inflating the results on non-randomized studies). The use of registry data should be pre-agreed with health authorities.<br>Each indication cohort would be sized for	• By adjusting the decision rules or sample size within each basket, investigators can limit the overall false-positive rate.<br>• […] the use of statistical modeling can enable efficacy information to be shared among the baskets, improving efficiency and thereby theoretically allowing for enrollment of fewer patients.(Tao2018)

				accelerated approval based on a predetermined surrogate endpoint (i.e. response rate, RR, or progression free survival, PFS) reasonably likely to predict clinical benefit (i.e. overall survival, OS).<br>The false positive rate for the surrogate would be pre-agreed with health authorities.<br>Effect sizes of benefit judged by hazard ratio (or by percentage improvement in median) are typically larger for surrogate endpoints compared to OS, and larger benefits can be detected with smaller sample sizes. Therefore, multiple indication cohorts can generally be pooled into a basket study of comparable size to a standard confirmatory study.<br>Tumor indications failing to meet the surrogate hurdle for accelerated approval would be "pruned"(removed from the basket). To adjust for inflation of the false positive rate of the final pooled analysis by "random high bias" due to selective pruning (please see random high bias, pruning of indications, and the false positive rate below), a prospectively designed adjustment would lower the nominal false positive rate (false positive rate before adjustment for random high bias) for the remaining indications. This adjustment amounts to a statistical penalty for using information within the study for adaptation.<br>Additional indications may be pruned based on external data such as maturing early stage data involving the definitive clinical benefit endpoint (Figure 3), or data from other agents in the class. Pruning based on external data does not inflate the false positive rate of the pooled analysis, and does not incur a statistical penalty.<br>To maintain the power of the pooled analysis after pruning, a sample size adjustment for the remaining indications may be required. (Beckman2016)	
			Basket trials usually test the effect of one drug in a single/multiple arms of cancer patients who share a specific biomarker or molecular aberration, regardless of histology or organ involvement. (Leonetti2019)		• In order for a confirmatory basket trial to meet acceptance from health authorities, it will be necessary for the false positive rate of the pooled analysis to be rigorously controlled.<br>• […] we recommend that the trial include a testing platform such as sequencing which may identify other options for ineligible patients. (Beckman2016)
			Basket trial designs offer the possibility to include multiple molecularly defined subpopulations,		Adjusted posterior probabilities were computed in accordance with the trial's reported design strategy, for which hypothesis testing assumed identical null

For peer review only

			often across histology or tumor types, but included in one cohesive design to evaluate the targeted therapy in question. (Mandrekar2015)		response rates for all organ sites. This assumption, if violated, would preclude implementation of basket trials devised to pool patients harboring common molecular tumor types arising from disparate clinical subtypes. (Hobbs2018_Statistical challenges)
			[…] trials designed to evaluate single drugs across multiple populations (Mazzarella2020)		In a basket trial, the opportunity for pooling is across histologies, and it may be appropriate if there is reasonably strong scientific rationale that the activity of the agent would be similar in the different histologies. (Yee2019)
			[…] evaluate whether a certain actionable mutations of interest (aMOI) or biomarker signature is predictive of response to a targeted drug regardless of the tumor of origin. (Moore2016)		
			Basket trials are a histologically agnostic trial design which recruit patients whose tumours contain a specific genomic aberration of interest. (O'Brien2017)		
			Basket trials refer to designs in which a targeted therapy is evaluated on multiple diseases that have common molecular alternations (Park2020)		
			[…] marker-specific but tumor agnostic and conducted in parallel without analyses across protocols (Renfro2016_Clinical trial designs incorporating)		
			A basket trial is similar to an umbrella trial in that there may be a common genetic screening platform, multiple study therapies, and multiple molecular subgroups. However, a basket trial typically enrolls multiple disease types to each of several marker-based cohorts, and these are conducted under a single protocol. (Renfro2017_Precision oncology)		
			A basket trial is a master protocol for which patient eligibility is defined by the presence of a particular biomarker or molecular alteration rather than a particular cancer type. Basket trials are predicted on the hypothesis that the molecular characterization of a particular tumor		

For peer review only

			predicts response to a matched (tar geted) treatment to a greater extent or independent of tumor histology. (Renfro2017_Statistical controversies)		
			Basket trials (also referred as pan-tumor or tissue-agnostic trials) are designed to evaluate the effect of a drug that targets a single mutation or a specific pathway in various tumor types. These trials are simple, including specific treatment arms for various tumors of origin and location "baskets" or complex, evaluating multiple drugs across selected genetic alterations in various tumor types (Said2019)		
			Basket trials are focused on the underlying target and not the disease or clinical syndrome per se. (Shah2017)		
			In contrast to umbrella and platform trials, Basket trials are not focused on patients with a single disease histology. Basket trials are focused instead on patients with a single genomic alteration or class of alterations. (Simon2017_Critical review)		
			[...] patient eligibility is based on a defined genomic alteration rather than on primary site. Basket trials are phase 2 trials. They can be nonrandomized or randomized and include a single drug or multiple individual drugs (Simon2016_Genomic alteration)		
			[...] patient eligibility is based on a defined genomic alteration rather than on primary site. (Simon2018_New designs for basket clinical trials)		
			"Basket trials" test whether a drug is effective in patients with specific genetic alterations regardless of their disease of origin. (Soldatos2019)		
			Unlike most clinical trials, which test a drug against a specific cancer type, the central organizing principle of a basket trial is themolecular		

For peer review only

			alteration. The term basket arises from each collection of patients that harbors a particular mutation. (Tao2018)		
			A basket trial is a histology-independent design where each sub-trial enrols multiple tumour types (the basket) with one common genetic mutation. (Verweij2019)		
			[…] innovative, histology-independent trial design, in which patients with cancer diagnoses of different histologies can be enrolled in the study protocol based on the presence of a specific molecular aberration. (Zardavas2015)		
			Basket or a bucket trials address a single targeted agent or subgroup across multiple histologic indications, the premise being that the fundamental classification of cancer is molecular, not histologic, and that core molecular signatures will be common across multiple histologies. (Beckman2016)		
			A basket trial is a trial for patients whose tumors have a specific molecular alteration and who are treated with an agent specifically targeted for that alteration. Basket trials are generally histology agnostic; that is, tumors of varying histologies are grouped together in a "basket" defined by a shared molecular alteration. (Yee2019)		
	**Randomised basket design**		A few multi-drug basket trials have been conducted which involve randomization to either a test drug which targets a mutation in the patient's tumor or to a control drug (Simon2018_New designs for basket clinical trials)	With randomization the trial may test the general policy of trying to match the drug to the genomics of the tumor. The null hypothesis here relates to a matching policy for a given set of drugs and genomic alterations used in the study. This policy is also determined by the type of genomic characterization performed and by the "rules" for matching drug to tumor. Rejection of the null hypothesis provides a proof of principle that matching can be useful overall but that null hypothesis is specific for the genomic alterations and the drugs on which the study is based. (Simon2018_New designs for basket)	

For peer review only

				[…] in a randomized controlled basket trial, each individual tumor indication has its own control group. A shared control group may be used for indications with a common standard of care as appropriate. (Chen2016)	
	**Non randomised basket design**				
		**Bayesian basket design**	[…] a different kind of Bayesian design for evaluating the response probabilities for the primary sites included in a basket trial of a drug. (Simon2018_New designs for basket)	At any interim analysis one can compute the posterior probability of activity (i.e. pj=phi) for each of the stratum. If that posterior probability is too small, one may close accrual to that stratum. If that posterior probability is very large, one might wish to proceed with the next stage of development of the drug in that stratum. One might wish to cap the total accrual to the trial, accepting that drug evaluation for some strata of very low prevalence may remain uncertain. (Simon2018_New designs for basket)	
			[…] flexible design that could accommodate varying hypotheses while making pre-trial choices explicit. (Alexander2016)	We generated a procedure that utilizes prior knowledge of biomarker information by quantifying the belief in the strength of the biomarker-effect linkage and combined the procedure with a Bayesian adaptive randomization algorithm. (Alexander2016)	
				In this design, a Bayesian approach is used to model the response probabilities for the various histologic strata, and two hypotheses are considered: (1) the response probabilities for a particular targeted agent are equal across the corresponding histologic strata, and (2) the activity of the drug is independent across these strata. (Ou2019)	
			[…] a design to support multiarm biomarker-driven trials that is flexible by allowing several treatments with varying biomarker hypothesis strengths in the same framework. (Trippa2017)	Bayesian basket (BB) design evaluates multiple overlapping biomarker subgroups and associated experimental therapies. It starts with explicit a priori estimates regarding the predictive utility of a biomarker for each experimental arm and then learns during the trial, thereby generating valuable information about the biomarker while providing the efficiencies of biomarker-selected clinical trials. (Trippa2017)	
		**Sequential basket trial design with Bayesian monitoring rules**		[…] the sequential design strategy uses interim analyses based on the multisource exchangeability modeling (MEM) approach to identify exchangeable metabaskets and terminate enrollment to ineffective subtypes. (Hobbs2018_Bayesian basket trial)	

		**Bayesian latent subgroup trial (BLAST) design**	The BLAST design makes the interim go/no-go treatment decision in a group sequential fashion for each cancer type based on accumulating data. (Yuan2018)	Conditional on the latent subgroup membership of the cancer type, we jointly model the binary treatment response and the longitudinal biomarker measurement that represents the biological activity of the targeted agent. (Yuan2018)	
		**Bayesian hierarchical adaptive design**	Hierarchical modeling allows information about the treatment effect in one group to be "borrowed" when estimating the treatment effect in another group. (Berry2013)	In effect, the estimate of treatment effect in each group is shrunk toward the overall mean. The amount of shrinkage depends on the results, including the relative precision of estimates in the various groups.<br>In this design, the four patient groups are considered together in a single, integrated trial, and a Bayesian hierarchical model borrows information across the groups. (Berry2013)	
**Basket of basket design**			The BoB study is testing therapies in multiple disease settings/genetic contexts, encompassed by the development of companion diagnostics based on specific biomarkers in these genetic contexts, including circulating tumour DNA (ctDNA) analysis as a way to select patients for any of the tested drugs and thus increase the efficacy of treatments. (Garralda2019)	The study consists of two parts: (a) I-Profiler will allow the molecular characterization of tumours from patients with metastatic or recurrent solid tumours using a new profiling tool and select the most suitable treatment for these patients; and (b) I-Basket is a multimodular basket trial, with different cohorts for genomically selected populations.(Garralda2019)<br>First, the patient's tumour (biopsy, plasma) is molecularly profiled by various multiplexed assays. Cancer patients with an appropriate molecular profile can then participate either in industry sponsored basket trials or in iBasket, a multi-modular investigator-initiated basket protocol. Modules can be added or dropped based on the results and may have different statistical designs (Bayesian, adaptive). Each module has individual arms with genomically selected patient populations. (Verweij2019)	
**Umbrella design**			Patients with exactly one of the targeted biomarkers are assigned to the associated sub-study evaluating an investigational therapy targeted against that aberration. For patients with more than one of the targeted biomarkers, assignment is randomized between the sub-studies they are eligible for using an algorithm that gives more weight to studies with lower prevalence biomarkers. Patients whose tumors alterations don't fall into any of the available matched drug-biomarker sub-studies are assigned to a non-match sub-study. Therefore all	The sample size for each sub-study is determined based on the biomarker prevalence, maintaining all other design parameters the same across sub-studies. (Ferrarotto2015)	1. Consistency of biomarker assay across sites is important<br>2. Planning requires wellcoordinated efforts among members of multidisciplinary team<br>3. Often needs international partnerships to make it feasible (Le-Rademacher2018)

			screened patients who satisfy the clinical eligibility criteria have a study in which to enroll. (Ferrarotto2015)		
			An umbrella trial is a master protocol for which the patient's eligibility is defined by the presence of a tumour type that is substratified according to specific molecular alterations matched to different anticancer therapies. (Garralda2019)	Within a conventionally defined disease (eg, diabetic kidney disease [DKD]), various biomarker-based subgroups are defined and different drugs are tested in these subgroups. This approach supports individualizing treatments and personalized medicine. (Heerspink2018_New clinical trial designs)	The randomization is adaptive, which means as certain subtypes respond better to a certain arm, the randomization probability for a patient with that subtype being randomized to that arm increases. In the same manner, if a certain subtype has no responses to a certain arm, the randomization probability of that arm for that subtype decreases and may even go to 0 if the arm is completely dropped for that subtype. (Moore2016)
			To study multiple targeted therapies in the context of a single disease. (Heerspink2018_New clinical trial designs)	In an umbrella trial design, patients are first screened for and assigned to a specific biomarker subgroup. Patients in each subgroup are then assigned to one of the therapies specifically targeting the biomarker they harbor. Some umbrella trials allow inclusion of a subgroup of patients with no actionable biomarker. Each of these biomarker subgroups forms a substudy of the overall trial (Le-Rademacher2018)	***Refers to both umbrella and basket design:***<br>• Careful evaluations of the pre-existing clinical evidence and underlying biologic assumptions are required to ensure that there is a biologic plausibility for the targeted interventions<br>• Accuracy of biomarker tests is important; however, because all medical tests will have some degree of inaccuracy, it is important to account for inaccuracy (ie, false-positive rates) in the trial planning stage to avoid underpowering the trial<br>• If there are multiple tumor types involved, the accuracy of biomarker tests should be similar between these tumors<br>• The biospecimen collection process should be easy, and relatively uniform high biospecimen quality and biospecimen yield must be achievable, especially for basket trials that have multiple diseases<br>• Prevalence of the biomarker(s) used should be anticipated with possible recruitment challenges<br>• The sample size calculations for umbrella

For peer review only

					trials, conversely, may be done for each of the subgroups because there are multiple targeted interventions being evaluated in umbrella trials<br>• Targeted intervention strategies rely on predictive risk factors that determine whether the patient will respond to a given intervention<br>• Use of randomization and a control group with adequate sample size can determine whether the risk factor is predictive or not<br>• If randomization is not feasible, statistical adjustments can be made. However, there are issues with making statistical adjustments with smaller data sets<br>• If there is adequate sample size, it is important to note that statistical adjustments can only account for measurable factors (Park2020)
			The umbrella design tests multiple targeted therapies in different biomarker-matched subgroups of patients, all of whom present the same tumor type or cancer histology. (Janiaud2019)	Patients are screened for a specific set of biomarkers and assigned to a biomarker-driven substudy (targeted design) if it is determined that they have one of the target biomarkers. (Mandrekar2015)	• Requires excellent assay performance<br>• Requires fast assay turn-around time<br><br>(Renfro2016_Clinical trial designs incorporating)
			Umbrella trials take patients with the same type of cancer, and assign them to treatment arms based on unique mutations (Joshi2018)	• Risk factors are used to stratify patients into multiple subgroups (patient stratification);<br>• Umbrella trials have multiple interventions, with intervention assignment being determined based on their risk factor;<br>• Similar to basket trials, intervention assignment may or may not be determined using randomization;<br>• Compared with basket trials, it may be easier to pick the choice in the control group for umbrella trials because there is one disease being studied;<br>• The existing standard of care (or placebo, if there is no established care) for the disease being studied may be used as the control for all of the subgroups (Park2020)	

For peer review only

			Umbrella trials select on the basis of a tumor type or histology […] (Lam2018_Accelerating therapeutic)	In an umbrella trial, patients with tumors from the specified cancer type are centrally screened and assigned to one of several molecularly defined subtrials where they receive (or perhaps are randomized to) a matched targeted treatment. In such trials, the relevant markers are regarded as refinements of (rather than replacements of) tumor type. (Renfro2017_Statistical controversies)	In an umbrella trial, the opportunity for pooling is across substudies defined by different biomarkers. (Yee2019)
			[…] umbrella trials evaluate multiple targeted therapies in a single-tumor type. (Lam2018_Master protocols)		• In umbrella trials, in which different experimental treatments in different biomarker subgroups within the same protocol are evaluated, an overarching statistical design that is common to all treatment arms can be deployed.<br>• […] rates of recruitment to each cohort can vary dramatically requiring interim analyses at multiple time points. (Blagden2020)
			Umbrella trials enroll patients with a single type or class of tumor. After central screening, patients are assigned to one of the many sub-trials on the basis of their molecular alteration, where they are treated (or can be treated, when randomized) with a matched targeted compound. (Leonetti2019)	In the umbrella design a separate enrichment trial is conducted for each biomarker stratum. The enrichment design for a given stratum uses as the test regimen a drug expected to be active for the alteration defining that stratum. (Simon2017_Critical review)	Thus, an umbrella trial consists of multiple substudies, each with independent subgroups of patients receiving different therapies and with the option of assuming different statistical parameters for independent designs. The substudies, however, exist under an overarching master protocol that uses a common infrastructure for screening and treatment assignment to reduce the cost and time associated with enrollment to unrelated and often sequential biomarker-informed studies. (Ou2019)
			Umbrella trials include a central infrastructure for screening and identification of patients, and focus on a single tumor type or histology with multiple subtrials, each testing a targeted therapy within a molecularly defined subset. (Mandrekar2015)	As with a basket trial, the tumor molecular screening can be performed as part of the trial or in the community. Any subtrial can be a single-arm trial designed to evaluate the efficacy of a targeted agent, or a randomized trial with a standard-treatment control arm (which could be observation). Unlike basket trials, patients without a target match in an umbrella trial can easily be put on a randomized subtrial of 2 relevant treatments for the histology. However, because patients with the designated alterations have been excluded from the nonmatch subtrial, there may be some question as to what population the results will generalize. (Yee 2019)	
			[…] trials designed to evaluate […] multiple drugs on a single population (Mazzarella2020)		
			Use of adaptive randomization and a common platform design is revolutionizing how we screen new drugs. When this strategy is applied		

For peer review only

			to one tumor type with multiple different sub studies, we are describing an umbrella trial. (Moore2016)		
			Umbrella trials, in contrast to basket trials, recruit patients with one histological diagnosis, but then allocate patients to specific arms within the trial based on the presence of specific molecular alterations in their tumours. (O'Brien2017)		
			Umbrella trials, on the other hand, evaluate multiple targeted therapies for a single disease that is stratified into subgroups by molecular alternation. (Park2019_Systematic review)		
			Umbrella trials, conversely, are prospective clinical trials that test multiple targeted interventions for a single disease based on predictive biomarkers or other predictive patient risk factors. (Park2020)		
			In an umbrella trial, a common genomic screening platform and central screening infrastructure are used to assign patients to unique marker-enriched protocols. (Renfro2017_Precision oncology)		
			[…] an umbrella trial generally restricts enrollment to a single type or class of cancers (Renfro2017_Statistical controversies)		
			An umbrella trial is another type of master protocol where patients with a common disease type (e.g., advanced non-squamous cell lung cancer) are enrolled to parallel cohorts or sub-trials that are similarly marker-driven. In this instance, the umbrella "over" the various sub-trials is the larger disease population from which the marker-based cohorts were derived. Umbrella trials may include phase II or phase II/III trials, wherein the individual marker-specific sub-trials or cohorts may be either single-arm studies of paired targeted agents, or		

For peer review only

			randomized studies comparing targeted agents versus placebo or standard of care. (Renfro2018_Definitions and statistical)		
			In an umbrella trial design, a variety of targeted treatments are tested in parallel. (Shah2017)		
			In the umbrella design a separate enrichment trial is conducted for each biomarker stratum. The enrichment design for a given stratum uses as the test regimen a drug expected to be active for the alteration defining that stratum. (Simon2017_Critical review)		
			[…] enroll many marker-defined cohorts in parallel under the "umbrella" of one disease area (Simon2010_Clinical trial designs)		
			An umbrella trial is restricted to patients with a single primary site of cancer but uses different drugs to target patients with different genomic alterations. (Simon2016_Genomic alterations)		
			Umbrella phase 3 designs consist of a combination of several enrichment designs conducted with a common genomic alteration testing infrastructure […]. (Simon2016_Genomic alterations)		
			Umbrella designs involve several molecularly targeted test drugs and a single primary site population of patients. (Simon2018_New designs for basket)		
			These protocols generally offer multiple therapeutic options matched to the patient's individual tumor genome. (Tao2018)		
			Umbrella trials involve a single histology and different treatments based on the genomic alterations in patient subgroups. (Tsimberidou2020)		
			An umbrella trial evaluates the efficacy of different targeted agents each against a different genetic mutations (sub-trials) within a single histology ("the umbrella").		

For peer review only

			(Verweij2019)		
			An umbrella trial is designed to enroll patients with a specific histology, and any of multiple potential tumor molecular alterations, who are assigned to different subtrials based on those alterations. (Yee2019)		
			Umbrella trials assign patients to one of potentially many treatment arms, based on a specific cancer type and genetic markers. (Soldatos2019)		
			Patients are screened for a panel of biochemical, genetic, and/or immunologic markers associated with their disease and, on the basis of the markers detected, assigned to a biomarker-driven treatment strategy or targeted therapy that is most likely to result in favorable outcomes. (Ou2019)		
	**Randomised umbrella design**				
	**Non randomised umbrella design**				
		**Bayesian adaptive umbrella design**			
**Umbrella-basket hybrid**					

References

1. Ahmad T, O'Connor CM. Therapeutic Implications of Biomarkers in Chronic Heart Failure. Clin Pharmacol Ther. 2013 Oct;94(4):468–79.
2. Alexander BM, Lorenzo T. Bayesian baskets: A novel approach to biomarker-based clinical trial design. J Clin Oncol. 2016;34: e14057
3. Alexander BM, Trippa L, Gaffey S, Arrillaga-Romany IC, Lee EQ, Rinne ML, et al. Individualized Screening Trial of Innovative Glioblastoma Therapy (INSIGhT): A Bayesian Adaptive Platform Trial to Develop Precision Medicines for Patients With Glioblastoma. JCO Precis Oncol. 2019 Dec;(3):1–13.
4. Antoniou M, Jorgensen AL, Kolamunnage-Dona R. Biomarker-Guided Adaptive Trial Designs in Phase II and Phase III: A Methodological Review. Soyer HP, editor. PLOS ONE. 2016 Feb 24;11(2):e0149803.
5. Antoniou M, Kolamunnage-Dona R, Jorgensen A. Biomarker-Guided Non-Adaptive Trial Designs in Phase II and Phase III: A Methodological Review. J Pers Med. 2017 Jan 25;7(1):1.
6. Barry WT, Perou CM, Marcom PK, Carey LA, Ibrahim JG. The Use of Bayesian Hierarchical Models for Adaptive Randomization in Biomarker-Driven Phase II Studies. J Biopharm Stat. 2015 Jan 2;25(1):66–88.

For peer review only

7. Beckman R, Antonijevic Z, Kalamegham R, Chen C. Adaptive Design for a Confirmatory Basket Trial in Multiple Tumor Types Based on a Putative Predictive Biomarker. Clin Pharmacol Ther. 2016 Dec;100(6):617–25.
8. Berry DA. The Brave New World of clinical cancer research: Adaptive biomarker-driven trials integrating clinical practice with clinical research. Mol Oncol. 2015 May;9(5):951–9.
9. Berry SM, Broglio KR, Groshen S, Berry DA. Bayesian hierarchical modeling of patient subpopulations: Efficient designs of Phase II oncology clinical trials. Clin Trials J Soc Clin Trials. 2013 Oct;10(5):720–34.
10. Blagden SP, Billingham L, Brown LC, Buckland SW, Cooper AM, Ellis S, et al. Effective delivery of Complex Innovative Design (CID) cancer trials—A consensus statement. Br J Cancer. 2020 Feb 18;122(4):473–82.
11. Cabarrou B, Sfumato P, Leconte E, Boher JM, Filleron T. Designing phase II clinical trials to target subgroup of interest in a heterogeneous population: A case study using an R package. Comput Biol Med. 2018 Sep;100:239–46.
12. Cecchini M, Rubin EH, Blumenthal GM, Ayalew K, Burris HA, Russell-Einhorn M, et al. Challenges with Novel Clinical Trial Designs: Master Protocols. Clin Cancer Res. 2019 Apr 1;25(7):2049–57.
13. Chen C, Li X (Nicole), Yuan S, Antonijevic Z, Kalamegham R, Beckman RA. Statistical Design and Considerations of a Phase 3 Basket Trial for Simultaneous Investigation of Multiple Tumor Types in One Study. Stat Biopharm Res. 2016 Jul 2;8(3):248–57.
14. Diao G, Dong J, Zeng D, Ke C, Rong A, Ibrahim JG. Biomarker threshold adaptive designs for survival endpoints. J Biopharm Stat. 2018 Nov 2;28(6):1038–54.
15. Dienstmann R, Rodon J, Tabernero J. Optimal design of trials to demonstrate the utility of genomically-guided therapy: Putting Precision Cancer Medicine to the test. Mol Oncol. 2015 May;9(5):940–50.
16. Doorenbos AZ, Haozous EA, Jang MK, Langford D. Sequential multiple assignment randomization trial designs for nursing research. Res Nurs Health. 2019 Dec;42(6):429–35.
17. Eng KH. Randomized reverse marker strategy design for prospective biomarker validation. Stat Med. 2014 Aug 15;33(18):3089–99.
18. Fadoukhair Z, Zardavas D, Chad MA, Goulioti T, Aftimos P, Piccart M. Evaluation of targeted therapies in advanced breast cancer: the need for large-scale molecular screening and transformative clinical trial designs. Oncogene. 2016 Apr;35(14):1743–9.
19. Ferrarotto R, Redman MW, Gandara DR, Herbst RS, Papadimitrakopoulou V. Lung-MAP-framework, overview, and design principles. Chin Clin Oncol. 2015;4(3):1–6.
20. Freidlin B, Korn EL. Biomarker-adaptive clinical trial designs. Pharmacogenomics. 2010 Dec;11(12):1679–82.
21. Freidlin B, Korn EL, Gray R. Marker Sequential Test (MaST) design. Clin Trials J Soc Clin Trials. 2014 Feb;11(1):19–27.
22. Galanis E, Wu W, Sarkaria J, Chang SM, Colman H, Sargent D, et al. Incorporation of Biomarker Assessment in Novel Clinical Trial Designs: Personalizing Brain Tumor Treatments. Curr Oncol Rep. 2011 Feb;13(1):42–9.
23. Gao Z, Roy A, Tan M. Multistage adaptive biomarker-directed targeted design for randomized clinical trials. Contemp Clin Trials. 2015 May;42:119–31.
24. Garralda E, Dienstmann R, Piris-Giménez A, Braña I, Rodon J, Tabernero J. New clinical trial designs in the era of precision medicine. Mol Oncol. 2019 Mar;13(3):549–57.
25. Gilson C, Chowdhury S, Parmar MKB, Sydes MR. Incorporating Biomarker Stratification into STAMPEDE: an Adaptive Multi-arm, Multi-stage Trial Platform. Clin Oncol. 2017 Dec;29(12):778–86.
26. Gómez-López G, Dopazo J, Cigudosa JC, Valencia A, Al-Shahrour F. Precision medicine needs pioneering clinical bioinformaticians. 2017. :1–15.
27. Heerspink HJL, List J, Perkovic V. New clinical trial designs for establishing drug efficacy and safety in a precision medicine era. Diabetes Obes Metab. 2018 Oct;20:14–8.
28. Heerspink HJL, Perkovic V. Trial Design Innovations to Accelerate Therapeutic Advances in Chronic Kidney Disease: Moving from Single Trials to an Ongoing Platform. Clin J Am Soc Nephrol. 2018 Jun 7;13(6):946–8.
29. Hobbs BP, Kane MJ, Hong DS, Landin R. Statistical challenges posed by uncontrolled master protocols: sensitivity analysis of the vemurafenib study. Ann Oncol. 2018 Dec;29(12):2296–301.
30. Hobbs BP, Landin R. Bayesian basket trial design with exchangeability monitoring: Bayesian Basket Trial Design with Exchangeability Monitoring. Stat Med. 2018 Nov 10;37(25):3557–72.
31. Hong F, Simon R. Run-In Phase III Trial Design With Pharmacodynamics Predictive Biomarkers. J Natl Cancer Inst. 2013;105(21):6.
32. Janiaud P, Serghiou S, Ioannidis JPA. New clinical trial designs in the era of precision medicine: An overview of definitions, strengths, weaknesses, and current use in oncology. Cancer Treat Rev. 2019 Feb;73:20–30.
33. Johnson DR, Galanis E. Incorporation of Prognostic and Predictive Factors Into Glioma Clinical Trials. Curr Oncol Rep. 2013 Feb;15(1):56–63.
34. Jones CL, Holmgren E. An adaptive Simon Two-Stage Design for Phase 2 studies of targeted therapies. Contemp Clin Trials. 2007 Sep;28(5):654–61.
35. Joshi YB, Light GA. Using EEG-Guided Basket and Umbrella Trials in Psychiatry: A Precision Medicine Approach for Cognitive Impairment in Schizophrenia. Front Psychiatry. 2018 Nov 19;9:554.
36. Kaplan R. The FOCUS4 design for biomarker stratified trials. Chin Clin Oncol. 2015;4(3):1–10.
37. Kesselmeier M, Scherag A. Adaptive clinical trials in sepsis research: pros and cons. Infection. 2019; 47 (Suppl 1): S1-S67.
38. Kidwell KM, Wahed AS. Weighted log-rank statistic to compare shared-path adaptive treatment strategies. Biostatistics. 2013 Apr 1;14(2):299–312.
39. Kimani PK, Todd S, Renfro LA, Stallard N. Point estimation following two-stage adaptive threshold enrichment clinical trials: Estimators for adaptive threshold enrichment clinical trials. Stat Med. 2018 Sep 30;37(22):3179–96.
40. Lam M, Loree JM, Lima AAP, Chun YS, Kopetz S. Accelerating Therapeutic Development through Innovative Trial Design in Colorectal Cancer. Curr Treat Options Oncol. 2018 Feb;19(2):11.
41. Lam VK, Papadimitrakopoulou V. Master protocols in lung cancer: experience from Lung Master Protocol. Curr Opin Oncol. 2018 Mar;30(2):92–7.
42. Le-Rademacher J, Dahlberg S, Lee JJ, Adjei AA, Mandrekar SJ. Biomarker Clinical Trials in Lung Cancer: Design, Logistics, Challenges, and Practical Considerations. J Thorac Oncol. 2018 Nov;13(11):1625–37.

For peer review only

43. Leonetti A, Boyd L, Giuliani J, Giovannetti E, Garajová I. Light and shadow on innovative clinical trial designs: reflections from the EORTC-PAMM course on 'preclinical and early-phase clinical pharmacology.' Expert Rev Clin Pharmacol. 2019 Nov 2;12(11):1033–6.
44. Lin J-A, He P. Reinventing clinical trials: a review of innovative biomarker trial designs in cancer therapies. Br Med Bull. 2015 Jun;114(1):17–27.
45. Liu S, Lee JJ. An overview of the design and conduct of the BATTLE trials. Chin Clin Oncol. 2015;4(3):13.
46. Mandrekar SJ, Dahlberg SE, Simon R. Improving Clinical Trial Efficiency: Thinking outside the Box. Am Soc Clin Oncol Educ Book. 2015 May;(35):e141–7.
47. Matsui S, Crowley J. Biomarker-Stratified Phase III Clinical Trials: Enhancement with a Subgroup-Focused Sequential Design. Clin Cancer Res. 2018 Mar 1;24(5):994–1001.
48. Mazzarella L, Morganti S, Marra A, Trapani D, Tini G, Pelicci P, et al. Master protocols in immuno-oncology: do novel drugs deserve novel designs? J Immunother Cancer. 2020 Mar;8(1):e000475.
49. Moore KN, Mannel RS. Is the NCI MATCH trial a match for gynecologic oncology? Gynecol Oncol. 2016 Jan;140(1):161–6.
50. O'Brien C, Carter L, Cook N, Dean E. Novel Early Phase Clinical Trial Design in Oncology. Pharm Med. 2017 Oct;31(5):297–307.
51. Ohwada S, Morita S. Bayesian adaptive patient enrollment restriction to identify a sensitive subpopulation using a continuous biomarker in a randomized phase 2 trial. Pharm Stat. 2016 Sep;15(5):420–9.
52. Ondra T, Dmitrienko A, Friede T, Graf A, Miller F, Stallard N, et al. Methods for identification and confirmation of targeted subgroups in clinical trials: A systematic review. J Biopharm Stat. 2016 Jan 2;26(1):99–119.
53. Ou F-S, An M-W, Ruppert AS, Mandrekar SJ. Discussion of Trial Designs for Biomarker Identification and Validation Through the Use of Case Studies. JCO Precis Oncol. 2019 Dec;(3):1–10.
54. Park JJH, Siden E, Zoratti MJ, Dron L, Harari O, Singer J, et al. Systematic review of basket trials, umbrella trials, and platform trials: a landscape analysis of master protocols. Trials. 2019 Dec;20(1):572.
55. Park JJH, Hsu G, Siden EG, Thorlund K, Mills EJ. An overview of precision oncology basket and umbrella trials for clinicians. CA Cancer J Clin. 2020 Mar;70(2):125–37.
56. Perco P, Pena M, Heerspink HJL, Mayer G. Multimarker Panels in Diabetic Kidney Disease: The Way to Improved Clinical Trial Design and Clinical Practice? Kidney Int Rep. 2019 Feb;4(2):212–21.
57. Renfro LA, Mallick H, An M-W, Sargent DJ, Mandrekar SJ. Clinical trial designs incorporating predictive biomarkers. Cancer Treat Rev. 2016 Feb;43:74–82.
58. Renfro LA, An M-W, Mandrekar SJ. Precision oncology: A new era of cancer clinical trials. Cancer Lett. 2017 Feb;387:121–6.
59. Renfro LA, Sargent DJ. Statistical controversies in clinical research: basket trials, umbrella trials, and other master protocols: a review and examples. Ann Oncol. 2017 Jan;28(1):34–43.
60. Renfro LA, Mandrekar SJ. Definitions and statistical properties of master protocols for personalized medicine in oncology. J Biopharm Stat. 2018 Mar 4;28(2):217–28.
61. Riddell CA, Zhao Y, Petkau J. An adaptive clinical trials procedure for a sensitive subgroup examined in the multiple sclerosis context. Stat Methods Med Res. 2016 Aug;25(4):1330–45.
62. Rosenblum M, Hanley DF. Adaptive Enrichment Designs for Stroke Clinical Trials. Stroke. 2017 Jul;48(7):2021–5.
63. Said R, Tsimberidou A-M. Basket Trials and the MD Anderson Precision Medicine Clinical Trials Platform: Cancer J. 2019;25(4):282–6.
64. Saville BR, Berry SM. Efficiencies of platform clinical trials: A vision of the future. Clin Trials J Soc Clin Trials. 2016 Jun;13(3):358–66.
65. Semler MW, Bernard GR, Aaron SD, Angus DC, Biros MH, Brower RG, et al. Identifying Clinical Research Priorities in Adult Pulmonary and Critical Care. NHLBI Working Group Report. Am J Respir Crit Care Med. 2020 Aug 15;202(4):511–23.
66. Shah SJ. Innovative Clinical Trial Designs for Precision Medicine in Heart Failure with Preserved Ejection Fraction. J Cardiovasc Transl Res. 2017 Jun;10(3):322–36.
67. Simon R. Clinical trial designs for evaluating the medical utility of prognostic and predictive biomarkers in oncology. Pers Med. 2010 Jan;7(1):33–47.
68. Simon R. Clinical trials for predictive medicine: new challenges and paradigms. Clin Trials J Soc Clin Trials. 2010 Oct;7(5):516–24.
69. Simon R. Genomic Alteration–Driven Clinical Trial Designs in Oncology. Ann Intern Med. 2016 Aug 16;165(4):270.
70. Simon R. Critical Review of Umbrella, Basket, and Platform Designs for Oncology Clinical Trials: Review of umbrella, basket, and platform trial designs. Clin Pharmacol Ther. 2017 Dec;102(6):934–41.
71. Simon R. New designs for basket clinical trials in oncology. J Biopharm Stat. 2018 Mar 4;28(2):245–55.
72. Simon KC, Tideman S, Hillman L, Lai R, Jathar R, Ji Y, et al. Design and implementation of pragmatic clinical trials using the electronic medical record and an adaptive design. JAMIA Open. 2018 Jul 1;1(1):99–106.
73. Soldatos, Kaduthanam, Jackson. Precision Oncology—The Quest for Evidence. J Pers Med. 2019 Sep 5;9(3):43.
74. Spencer AV, Harbron C, Mander A, Wason J, Peers I. An adaptive design for updating the threshold value of a continuous biomarker: An Adaptive Design for Updating the Threshold of a Biomarker. Stat Med. 2016 Nov 30;35(27):4909–23.
75. Tajik P, Zwinderman AH, Mol BW, Bossuyt PM. Trial Designs for Personalizing Cancer Care: A Systematic Review and Classification. Clin Cancer Res. 2013 Sep 1;19(17):4578–88.
76. Talisa VB, Yende S, Seymour CW, Angus DC. Arguing for Adaptive Clinical Trials in Sepsis. Front Immunol. 2018 Jun 28;9:1502.
77. Tao JJ, Schram AM, Hyman DM. Basket Studies: Redefining Clinical Trials in the Era of Genome-Driven Oncology. Annu Rev Med. 2018 Jan 29;69(1):319–31.
78. Trippa L, Alexander BM. Bayesian Baskets: A Novel Design for Biomarker-Based Clinical Trials. J Clin Oncol. 2017 Feb;35(6):JCO.2016.68.286.
79. Tsimberidou AM, Fountzilas E, Nikanjam M, Kurzrock R. Review of precision cancer medicine: Evolution of the treatment paradigm. Cancer Treat Rev. 2020 Jun;86:102019.
80. Uozumi R, Hamada C. Interim decision-making strategies in adaptive designs for population selection using time-to-event endpoints. J Biopharm Stat. 2017 Jan 2;27(1):84–100.
81. Verweij J, Hendriks HR, Zwierzina H, Hanauske, Wacheck V, Collignon O, et al. Innovation in oncology clinical trial design. Cancer Treat Rev. 2019 Mar;74:15–20.
82. Wang S-J, Hung HMJ, O'Neill R. Adaptive design clinical trials and trial logistics models in CNS drug development. Eur Neuropsychopharmacol. 2011 Feb;21(2):159–66.

For peer review only

83. Wang T, Wang X, Zhou H, Cai J, George SL. Auxiliary variable–enriched biomarker-stratified design. Stat Med. 2018 Dec 30;37(30):4610–35.
84. Xu Y, Trippa L, Müller P, Ji Y. Subgroup-Based Adaptive (SUBA) Designs for Multi-arm Biomarker Trials. Stat Biosci. 2016 Jun;8(1):159–80.
85. Yee LM, McShane LM, Freidlin B, Mooney MM, Korn EL. Biostatistical and Logistical Considerations in the Development of Basket and Umbrella Clinical Trials: Cancer J. 2019;25(4):254–63.
86. Yuan Y. Invited session 11 - Recent developments in umbrella, basket and platform trial designs. Clinical Trials. 2018; 15(S2);35-192
87. Zardavas D, Piccart-Gebhart M. Clinical Trials of Precision Medicine through Molecular Profiling: Focus on Breast Cancer. Am Soc Clin Oncol Educ Book. 2015 May;(35):e183–90.
88. Zhang W, Wang J, Menon S. Advancing cancer drug development through precision medicine and innovative designs. J Biopharm Stat. 2018 Mar 4;28(2):229–44.
89. Zhang Z, Chen R, Soon G, Zhang H. Treatment evaluation for a data-driven subgroup in adaptive enrichment designs of clinical trials: Treatment evaluation for a data-driven subgroup in adaptive enrichment designs of clinical trials. Stat Med. 2018 Jan 15;37(1):1–11.

For peer review only

Supplementary file VI. Examples of clinical trials

Type of trial designs	Sub-type of trial designs	Variations	Example(s)	Trial registration num.	Recruitment status as of 12 March 2021	Clinical Field	Phase	Reference
Marker stratified design			CALGB-30506	NCT00863512	Completed	Lung cancer	III	(1)
			EORTC10994 P53	NCT00017095	Completed	Breast cancer	III	(2)
			IBCSG trial IX	nf[1]	nf[1]	Breast cancer	nf[1]	(1)
			MARVEL	NCT00738881	Completed	Lung cancer	III	(1,3–6)
			MINDACT	NCT00433589	Ongoing	Breast cancer	III	(1)
			RTOG0825	NCT00884741	Completed	Glioblastoma	III	(1,7)
	Subgroup specific design	Sequential-subgroup specific design	PRIME	NCT00364013	Completed	Colorectal cancer	III	(1)
	Biomarker-positive and overall strategies	Biomarker-positive and overall strategies with parallel assessment	ARCHER	NCT01360554	Completed	Lung cancer	III	(1)
			MERiDIAN	NCT01663727	Completed	Breast cancer	III	(1)
			MONET1	NCT00460317	Completed	Lung cancer	III	(1)
			S0819	NCT00946712	Completed	Lung cancer	III	(1)
			SATURN	NCT00556712	Completed	Lung cancer	III	(1)
			ZODIAC	NCT00312377	Completed	Lung cancer	III	(1)
		Biomarker-positive and overall strategies with sequential assessment	N0147	NCT00079274	Completed	Colorectal cancer	III	(1)

For peer review only

		Marker sequential test design	ECOG E1910	NCT02003222	Ongoing	Leukemia	III	(1)
Hybrid design			TAILORx	NCT00310180	Completed	Breast cancer	III	(1,8)
Biomarker strategy design with biomarker assessment in the control arm			ERCC1	NCT00801736	Completed	Lung cancer	III	(9)
			GILT docetaxel	NCT00174629	Completed	Lung cancer	III	(1)
			LIFT	NCT02498977	Completed	Transplantation, Liver	IV	(10)
Biomarker strategy design without biomarker assessment in the control arm			GUIDE-IT	NCT01685840	Completed	Chronic Heart Failure	n/a[2]	(11)
			iPEGASUS	NCT03021525	Ongoing	Hemodynamic Instability; Cardiac Output, High; Peroperative Complication	n/a[2]	(12)
			OCTOPUS	ISRCTN81464462	Completed	Mild head injury	n/a[2]	(1)
			PUFFIN	NCT03654508	Ongoing	Asthma	n/a[2]	(13)
Modified biomarker strategy design			MINDACT	NCT00433589	Ongoing	Breast cancer	III	(8,14)
			NCI-MPACT	NCT01827384	Completed	Advanced malignant solid neoplasm	II	(5)
			SHIVA	NCT01771458	Unknown[3]	Reccurent/Metastatic Solid; Tumor Disease	II	(5,6,15)

For peer review only

Sequential Multiple Assignment Randomised Trial (SMART) design			Siyaphambili Study	NCT03500172	Completed	HIV	n/a[2]	(16)
Adaptive strategy for biomarker with measurement error			OPTIMA	ISRCTN42400492	Ongoing	Breast cancer	n/a[2]	(6)
Outcome-based adaptive randomization design			BATTLE	NCT00409968	Completed	Lung cancer	II	(5,6,17–19)
			I-SPY 2	NCT01042379	Ongoing	Breast cancer	II	(1,5,7,20–22)
			ProBio	NCT03903835	Ongoing	Prostate cancer	III	(23–25)
			SEPSIS-ACT	NCT02508649	Completed	Septic shock	II/III	(26)
Adaptive enrichment	Adaptive patient enrichment design		MISTIE	NCT01827046	Completed	Intracerebral Hemorrhage	III	(27)
			MK-0462-082 AM7	NCT01001234	Completed	Migraine	III	(28)
			THRIVE	NCT00543725	Completed	HIV	III	(29)
Adaptive parallel Simon two-stage design			-	NCT00958971	Completed	Breast cancer	II	(28)
Multi-arm multi-stage design			ATLANTIS	ISRCTN25859465	Ongoing	Bladder	II	(30)
			BIOMEDE	NCT02233049	Unknown[3]	Diffuse Intrinsic Pontine Glioma	II	(31,32)
			PanACEA MAMS	NCT01785186	Ongoing	Tuberculosis	II	(33)

For peer review only

			PLATFORM	NCT02678182	Ongoing	Gastric	II	(34)
			STAMPEDE	NCT00268476	Ongoing	Prostate cancer	II/III	(28,35,36)
		Two-stage adaptive seamless design	SEPSIS-ACT	NCT02508649	Completed	Septic shock	II/III	(26)
		Group sequential design	SHARP	NCT00105443	Completed	Liver cancer	III	(37)
Tandem two stage design			-	NCT00735917	Completed	Pancreas cancer	II	(28)
Platform design			BATTLE	NCT00409968	Completed	Lung cancer	II	(38)
			DIAN-TU	NCT01760005	Ongoing	Alzheimer's Disease	II/III	(39,40)
			EPAD	NCT02804789	Completed	Alzheimer's Disease	n/a[2]	(40)
			FOCUS4	ISRCTN90061546	Ongoing	Colorectal cancer	II/III	(41)
			FRACTION-GC	NCT2935634	Ongoing	Gastric Cancer	II	(42,43)
			FRACTION-Lung	NCT02750514	Ongoing	Lung cancer	II	(42,44)
			FRACTION-RCC	NCT2996110	Ongoing	Renal Cell Carcinoma	II	(42)
			GBM AGILE	NCT03970447	Ongoing	Glioblastoma	II/III	(45)
			I-SPY 2	NCT01042379	Ongoing	Breast cancer	II	(26)
			-	NCT03739710	Ongoing	Neoplasms	II	(46)
			ORCHARD	NCT03944772	Ongoing	Lung cancer	II	(47)
			PANGEA-IMBBP	NCT02213289	Ongoing	Adenocarcinoma	II	(48)

For peer review only

			PLATforM	NCT03484923	Ongoing	Melanoma	II	(49)
			SHIVA	NCT01771458	Unknown[3]	Reccurent/Metastatic Solid; Tumor Disease	II	(50)
			STAMPEDE	NCT00268476	Ongoing	Prostate cancer	II/III	(51,52)
		Bayesian adaptive platform trial	INSIGhT	NCT02977780	Ongoing	Glioblastoma	II	(53)
	Randomized embedded multifactorial adaptive platform (REMAP)		REMAP-CAP	NCT02735707	Ongoing	Community-acquired Pneumonia, Influenza, COVID-19	IV	(26)
			UPMC REMAP	NCT03861767	Ongoing	Aging	III	(54)
Basket design			ALCHEMIST	NCT02194738	Ongoing	Lung cancer	III	(51)
			BASKET 1	NCT00928525	Unknown[3]	Advanced Desmoid Tumor, Advanced Chondrosarcoma	II	(2)
			CAPTUR	NCT03297606	Ongoing	Lymphoma, Non-Hodgkin Multiple Myeloma Advanced Solid Tumors	II	(55)
			CLUSTER	NCT02059291	Completed	Fever	III	(40)
			CREATE	NCT01524926	Ongoing	Locally Advanced and/or Metastatic Anaplastic Large Cell Lymphoma; Locally Advanced and/or Metastatic Inflammatory Myofibroblastic Tumor; Locally Advanced	II	(56)

For peer review only

						and/or Metastatic Papillary Renal Cell Carcinoma Type; Locally Advanced and/or Metastatic Alveolar Soft Part Sarcoma; Locally Advanced and/or Metastatic Clear Cell		
			CUSTOM	NCT01306045	Ongoing	Lung cancer	II	(57)
			DART SWOG 1609	NCT02834013	Ongoing	Rare tumors	II	(58)
			DRUP	NCT02925234	Ongoing	Solid tumor, multiple myeloma or B cell non-Hodgkin lymphoma	II	(59)
			IMPACT 2	NCT02152254	Ongoing	Metastatic Malignant Neoplasm Recurrent Malignant Neoplasm	n/a[2]	(20)
			IGNYTE-ESO	NCT03967223	Ongoing	Neoplasms	II	(60)
			K-BASKET	NCT03491345 NCT03017521	Unknown[3]	Solid tumor	II	(2)
			Keynote 158	NCT02628067	Ongoing	Anal Cancer;Colorectal Cancer;Lung Cancer;Pancreas cancer;Endometrial, small intestine, cervical, vulvar, salivary gland carcinoma , mesothelioma and other advanced solid tumor	II	(61,62)

For peer review only

			MEDIOLA	NCT02734004	Ongoing	Ovarian Breast SCLC Gastric Cancers	II	(63–65)
			METADUR	NCT02811497	Ongoing	Colorectal carcinoma, ovarian and breast cancer	II	(2)
			MiMe-A	NCT03339843	Ongoing	Esophageal Adenocarcinoma, Esophagus SCC, Cholangiocarcinoma, Urothelial/Bladder Cancer, Nos Endometrial Cancer	II	(2)
			MOBILITY-001	NCT02399943	Ongoing	Colorectal cancer	II	(2)
			MOBILITY-002	NCT02428270	Ongoing	Pancreatic cancer, Adenocarcinoma	II	(2)
			MOBILITY-003	NCT02506517	Ongoing	Solid tumors	II	(2)
			MyPathway	NCT02091141	Ongoing	Neoplasms Solid Tumors; Biliary Cancer; Salivary Cancer; Bladder Cancer	II	(66)
			MoST	ACTRN12616000 908437	Ongoing	Solid tumor	II	(67,68)
			–	NCT03836352	Ongoing	Ovarian Cancer Hepatocellular Carcinoma Non-small Cell Lung Cancer Bladder Cancer Microsatellite Instability-High	II	(69)

For peer review only

			n/a	NCT02675829	Ongoing	Solid tumors	II	(70)
			NAVIGATE	NCT02576431	Ongoing	Solid Tumors Harboring NTRK Fusion	II	
			NCI CTRP	NCT02478320	Ongoing	Advanced cancers	II	(2)
			NCI-MATCH	NCT02465060	Ongoing	Advanced malignant solid neoplasm	II	(5,6,17,38,71–80)
			NCI-MPACT	NCT01827384	Ongoing	Advanced malignant solid neoplasm	II	(57,72,81,82)
			P10s Basket trial	NCT03003195	Ongoing	Neoplasms by Site Metastatic Cancer	II	(2)
			Paragon	ACTRN12610000796088 (prospectively registered)	Ongoing	Ovarian cancer	II	(2)
			SHIVA	NCT01771458	Unknown[3]	Reccurent/Metastatic Solid; Tumor Disease	II	(83)

For peer review only

			SIGNATURE	NCT01831726 NCT01885195 NCT01981187 NCT02002689 NCT02160041 NCT02186821 NCT02187783 NCT01833169	Completed	Solid tumor, hematologic malignancies	II	(2)
			STARTRK-2	NCT02568267	Ongoing	Solid tumor	II	(2)
			SUMMIT	NCT01953926	Ongoing	Solid Tumors Harboring Somatic HER2 or EGFR Exon 18 Mutations	II	(2)
			TAPUR	NCT02693535	Ongoing	Lymphoma, Non-Hodgkin Multiple Myeloma Advanced Solid Tumors	II	(20)
			TMB-H basket	UMIN000033182	Ongoing	Colorectal cancer, Gastric cancer, Esophageal cancer, Biliary tract cancer, Pancreatic cancer, and Other gastrointestinal cancer	II	(84)
			VE-BASKET	NCT01524978	Completed	Multiple Myeloma, Neoplasms	II	(2,66,85–87)
Basket of basket design			-	NCT03767075	Ongoing	Advanced Solid Tumor	II	(87–89)
Umbrella design			ADAPT	NCT01779206	Ongoing	Breast Cancer	II/III	(90–92)
			ALCHEMIST	NCT02194738 NCT02193282 NCT02201992 NCT02595944	Ongoing	Lung cancer	III	(2,5,17,38,41,73,77,93,94)

For peer review only

			BATTLE-1	NCT00411632<br>NCT00411671<br>NCT00410189<br>NCT00410059	Completed	Lung cancer	II	(2,95)
			BATTLE-2	NCT01248247	Ongoing	Lung cancer	II	(2)
			BFAST	NCT03178552	Ongoing	Lung cancer	II/III	(87)
			FOCUS4	ISRCTN90061546	Ongoing	Colorectal cancer	II/III	(2,30)
			HUDSON	NCT03334617	Ongoing	Lung cancer	II	(2)
			I-SPY 2	NCT01042379	Ongoing	Breast cancer	II	(2)
			Lung-MAP	NCT02154490<br>NCT02766335<br>NCT02785913<br>NCT02785939<br>NCT02965378<br>NCT02926638<br>NCT03373760<br>NCT03377556<br>NCT02785952	Ongoing	Lung cancer	II/III	(2,5,6,17,73,75–79,81,93,96–100)
			MiST	NCT03654833	Ongoing	Mesothelioma, Malignant	II	(101)
			MODUL	NCT02291289	Ongoing	Colorectal cancer	II	(102)
			MOSCATO	NCT01566019	Ongoing	Metastatic Solid Tumors (Any Localization)	n/a[2]	(89)
			-	NCT02276027	Completed	Lung cancer	II	(103)
			NCI-MATCH	NCT02465060	Ongoing	Advanced malignant solid neoplasm	II	(93)
			Pediatric MATCH	NCT03155620	Ongoing	Advanced Malignant Solid Neoplasm	II	(2)
			plasmaMATCH	NCT03182634	Ongoing	Breast cancer	II	(104)
			PLATO	ISRCTN88455282	Ongoing	Anal cancer	II/III	(105,106)

For peer review only

			Precision-Panc: PRIMUS	NCT04161417	Ongoing	Pancreas cancer	n/a[2]	(107)
			PRIMUS 002	ISRCTN34129115	Ongoing	Pancreas cancer	II	(108)
			SAFIR02_Lung	NCT02117167	Completed	Lung cancer	II	(56)
			SAFIR02_Breast	NCT02299999	Completed	Breast cancer	II	(56)
			SUKSES-S	NCT02688894	Ongoing	Small Cell Lung Cancers	II	(109,110)
			TRIUMPH	NCT03292250 NCT03356587	Unknown[3]	Head and neck squamous cell carcinoma	II	(2)
			TRUMP	NCT03574402	Ongoing	Lung cancer	II	(2)
			UPSTREAM	NCT03088059	Ongoing	Head and Neck Squamous Cell Carcinoma	II	(111)
			VIKTORY	NCT02299648	Ongoing	Molecular profiling	n/a[2]	(112)
			WINTHER	NCT01856296	Completed	Metastatic cancer	n/a[2]	(113)
			WSG ADAPT	NCT01781338	Ongoing	Breast cancer	II/III	(2)
		Bayesian adaptive umbrella design	National Lung Matrix Trial	NCT02664935	Ongoing	Lung cancer	II	(2,30,99)
		Randomized umbrella design	AMBITION	NCT03699449	Ongoing	Ovarian cancer	II	(114)
Umbrella-basket hybrid			MASTER KEY	UMIN000027552	Ongoing	Cancer	II	(115)
Umbrella-basket hybrid			NCI-MATCH	NCT02465060	Ongoing	Advanced malignant solid neoplasm	II	(82)

[1] Not found

[2] Not applicable is used on the Clinicaltrilas.gov website to describe trials without FDA-defined phases including trials of devices or behavioural interventions.

[3] Unknown is used to indicate a trial status that has not been verified within the past two years on the Clinical trials.gov website

For peer review only

References

1. Antoniou M, Kolamunnage-Dona R, Jorgensen A. Biomarker-Guided Non-Adaptive Trial Designs in Phase II and Phase III: A Methodological Review. J Pers Med. 2017 Jan 25;7(1):1.
2. Janiaud P, Serghiou S, Ioannidis JPA. New clinical trial designs in the era of precision medicine: An overview of definitions, strengths, weaknesses, and current use in oncology. Cancer Treat Rev. 2019 Feb;73:20–30.
3. Buch MH, Pavitt S, Parmar M, Emery P. Creative trial design in RA: optimizing patient outcomes. Nat Rev Rheumatol. 2013 Mar;9(3):183–94.
4. Ondra T, Dmitrienko A, Friede T, Graf A, Miller F, Stallard N, et al. Methods for identification and confirmation of targeted subgroups in clinical trials: A systematic review. J Biopharm Stat. 2016 Jan 2;26(1):99–119.
5. Renfro LA, An M-W, Mandrekar SJ. Precision oncology: A new era of cancer clinical trials. Cancer Lett. 2017 Feb;387:121–6.
6. Renfro LA, Mallick H, An M-W, Sargent DJ, Mandrekar SJ. Clinical trial designs incorporating predictive biomarkers. Cancer Treat Rev. 2016 Feb;43:74–82.
7. Galanis E, Wu W, Sarkaria J, Chang SM, Colman H, Sargent D, et al. Incorporation of Biomarker Assessment in Novel Clinical Trial Designs: Personalizing Brain Tumor Treatments. Curr Oncol Rep. 2011 Feb;13(1):42–9.
8. Tajik P, Zwinderman AH, Mol BW, Bossuyt PM. Trial Designs for Personalizing Cancer Care: A Systematic Review and Classification. Clin Cancer Res. 2013 Sep 1;19(17):4578–88.
9. Freidlin B, McShane LM, Korn EL. Randomized Clinical Trials With Biomarkers: Design Issues. JNCI J Natl Cancer Inst. 2010 Feb 3;102(3):152–60.
10. Clinicaltrials.gov. Liver Immunosuppression Free Trial (LIFT) [Internet]. Available from: https://clinicaltrials.gov/ct2/show/NCT02498977
11. Ahmad T, O'Connor CM. Therapeutic Implications of Biomarkers in Chronic Heart Failure. Clin Pharmacol Ther. 2013 Oct;94(4):468–79.
12. Funcke S, Saugel B, Koch C, Schulte D, Zajonz T, Sander M, et al. Individualized, perioperative, hemodynamic goal-directed therapy in major abdominal surgery (iPEGASUS trial): study protocol for a randomized controlled trial. Trials. 2018 Dec;19(1):273.
13. Vijverberg SJ, Pijnenburg MW, Hövels AM, Koppelman GH, Maitland-van der Zee A-H. The need for precision medicine clinical trials in childhood asthma: rationale and design of the PUFFIN trial. Pharmacogenomics. 2017 Mar;18(4):393–401.
14. Simon R. Clinical trial designs for evaluating the medical utility of prognostic and predictive biomarkers in oncology. Pers Med. 2010 Jan;7(1):33–47.
15. Paoletti X, Asselain B, Kamal M, Servant N, Huppé P, Bieche I, et al. Design and statistical principles of the SHIVA trial. Chin Clin Oncol. 2015;4(3):1–10.
16. Clinicaltrials.gov. HIV Treatment Retention Interventions for Women Living With HIV (Siyaphambili Study) [Internet]. Available from: https://clinicaltrials.gov/ct2/show/NCT03500172
17. Le-Rademacher J, Dahlberg S, Lee JJ, Adjei AA, Mandrekar SJ. Biomarker Clinical Trials in Lung Cancer: Design, Logistics, Challenges, and Practical Considerations. J Thorac Oncol. 2018 Nov;13(11):1625–37.

For peer review only

18. Liu S, Lee JJ. An overview of the design and conduct of the BATTLE trials. Chin Clin Oncol. 2015;4(3):13.
19. Bradbury P, Hilton J, Seymour L. Early-phase oncology clinical trial design in the era of molecularly targeted therapy: pitfalls and progress. Clin Investig. 2011 Jan;1(1):33–44.
20. Fountzilas E, Tsimberidou AM. Overview of precision oncology trials: challenges and opportunities. Expert Rev Clin Pharmacol. 2018 Aug 3;11(8):797–804.
21. Johnson DR, Galanis E. Incorporation of Prognostic and Predictive Factors Into Glioma Clinical Trials. Curr Oncol Rep. 2013 Feb;15(1):56–63.
22. Maitland ML, Schilsky RL. Clinical trials in the era of personalized oncology. CA Cancer J Clin. 2011 Nov;61(6):365–81.
23. Lindberg J, De Laere B, Crippa A, Eklund M, Grönberg H. ProBio: An outcome-adaptive, multi-arm, open-label, multiple assignment randomised controlled biomarker-driven trial in patients with metastatic castration-resistant prostate cancer. In Ann Oncol; 2019. p. v354.
24. Clinicaltrials.gov. ProBio: A Biomarker Driven Study in Patients With Metastatic Castrate Resistant Prostate Cancer (ProBio) [Internet]. Available from: https://clinicaltrials.gov/ct2/show/NCT03903835
25. Gronberg H, Eklund M, Lindberg J, Ullén A, Bjartell A, Andren O, et al. ProBio II: An adaptive and randomized multi-arm biomarker driven phase 2 study in men with castrate resistant prostate cancer (CRPC). In San Francisco, California;
26. Talisa VB, Yende S, Seymour CW, Angus DC. Arguing for Adaptive Clinical Trials in Sepsis. Front Immunol. 2018 Jun 28;9:1502.
27. Rosenblum M, Hanley DF. Adaptive Enrichment Designs for Stroke Clinical Trials. Stroke. 2017 Jul;48(7):2021–5.
28. Antoniou M, Jorgensen AL, Kolamunnage-Dona R. Biomarker-Guided Adaptive Trial Designs in Phase II and Phase III: A Methodological Review. PLOS ONE. 2016 Feb 24;11(2):e0149803.
29. Zhang W, Wang J, Menon S. Advancing cancer drug development through precision medicine and innovative designs. J Biopharm Stat. 2018 Mar 4;28(2):229–44.
30. Antoniou M, Kolamunnage-Dona R, Wason J, Bathia R, Billingham C, Bliss JM, et al. Biomarker-guided trials: Challenges in practice. Contemp Clin Trials Commun. 2019 Dec;16:100493.
31. Grill J, Teuff GL, Nysom K, Blomgren K, Hargrave D, McCowage G, et al. PDCT-01. Biological medicine for diffuse intrinsic pontine gliomas eradication (BIOMEDE): Results of the three-arm biomarker-driven randomized trial in the first 230 patients from Europe and Australia. In Neuro-Oncology; p. vi183.
32. Debily M-A, Kergrohen T, Varlet P, Le Teuff G, Nysom K, Blomgren K, et al. PDTM-36. Whole exome sequencing (WES) of DIPG patients from the BIOMEDE trial reveals new prognostic subgroups with specific oncogenis programmes. In Neuro-Oncology; 2019. p. vi195.
33. PanACEA consortium, Phillips P, Hoelscher M, Bratton D, Rehal S, Heinrich N, et al. Modifying the multi-arm multi-stage (MAMS) design for use in a phase II tuberculosis trial in sub-Saharan Africa with a time-to-event primary outcome. Trials. 2013 Nov;14(S1):P26, 1745-6215-14-S1-P26.
34. Cafferkey C, Chau I, Thistlethwaite F, Petty RD, Starling N, WatkinsSheela Rao D, et al. PLATFORM: Planning treatment of oesophago-gastric (OG) cancer randomised maintenance therapy trial. In San Francisco, California; 2016.
35. Kaplan R. The FOCUS4 design for biomarker stratified trials. Chin Clin Oncol. 2015;4(3):1–10.

For peer review only - http://bmjopen.bmj.com/site/about/guidelines.xhtml

36. Ocana A, Amir E, Vera-Badillo F, Seruga B, Tannock IF. Phase III Trials of Targeted Anticancer Therapies: Redesigning the Concept. Clin Cancer Res. 2013 Sep 15;19(18):4931–40.
37. Cheng A-L. Combining Adaptive Design and Omics for Future HCC Trials. In 2015.
38. Simon R. Critical Review of Umbrella, Basket, and Platform Designs for Oncology Clinical Trials: Review of umbrella, basket, and platform trial designs. Clin Pharmacol Ther. 2017 Dec;102(6):934–41.
39. Bateman RJ, Benzinger TL, Berry S, Clifford DB, Duggan C, Fagan AM, et al. The DIAN-TU Next Generation Alzheimer's prevention trial: Adaptive design and disease progression model. Alzheimers Dement. 2017 Jan;13(1):8–19.
40. Parke T. D2.1. Report on Terminology, References, and Scenarios for Platform Trials and Master Protocols [Internet]. Berry Consultants; 2020 Jun. Available from: https://eu-pearl.eu/wp-content/uploads/2020/06/EU-PEARL_D2.1_Report-on-Terminology-and-Scenarios-for-Platform-Trials-and-Masterprotocols.pdf
41. Renfro LA, Mandrekar SJ. Definitions and statistical properties of master protocols for personalized medicine in oncology. J Biopharm Stat. 2018 Mar 4;28(2):217–28.
42. Simonsen KL, Fracasso PM, Bernstein SH, Wind-Rotolo M, Gupta M, Comprelli A, et al. The Fast Real-time Assessment of Combination Therapies in Immuno-ONcology (FRACTION) program: innovative, high-throughput clinical screening of immunotherapies. Eur J Cancer. 2018 Nov;103:259–66.
43. Aanur P, Gutierrez M, Kelly RJ, Ajani JA, Ku GY, Denlinger CS, et al. FRACTION (Fast Real-time Assessment of Combination Therapies in Immuno-Oncology)-gastric cancer (GC): A randomized, open-label, adaptive, phase 2 study of nivolumab in combination with other immuno-oncology (IO) agents in patients with advanced GC. In 2017.
44. Fracasso PM, Freeman DJ, Simonsen K, Shen Y, Gupta M, Comprelli A, et al. A phase 2, fast real-time assessment of combination therapies in immuno-oncology trial in patients with advanced non-small cell lung cancer (FRACTION-lung). Ann Oncol. 2016 Oct;27:vi451.
45. Alexander BM, Ba S, Berger MS, Berry DA, Cavenee WK, Chang SM, et al. Adaptive Global Innovative Learning Environment for Glioblastoma: GBM AGILE. Clin Cancer Res. 2018 Feb 15;24(4):737–43.
46. Spigel D, Garassino M, Besse B, Sacher A, Barve M, Cousin S, et al. P1.01-110 Novel Regimens Versus Standard-of-Care in NSCLC: A Phase II, Randomized, Open-Label, Platform Trial Using a Master Protocol. In 2019.
47. Yu H, Goldberg S, Le X, Piotrowska Z, Smith P, Mensi I, et al. P2.01-22 ORCHARD: A Phase II Platform Study in Patients with Advanced NSCLC Who Have Progressed on First-Line Osimertinib Therapy. J Thorac Oncol. 2019 Oct;14(10):S647.
48. Joshi SS, Maron SB, Lomnicki S, Polite BN, Sharma M, Ibe J, et al. Personalized antibodies for gastroesophageal adenocarcinoma (PANGEA): A phase II precision medicine trial (NCT02213289). In San Francisco, California; 2018.
49. Weber J, Long GV, Haanen JB, Arance A, Dummer R, Nathan P, et al. A randomized, open-label, phase II open platform study evaluating the efficacy and safety of novel spartalizumab (PDR001) combinations in previously treated unresectable or metastatic melanoma (PLATForM). In 2018.
50. Leonetti A, Boyd L, Giuliani J, Giovannetti E, Garajová I. Light and shadow on innovative clinical trial designs: reflections from the EORTC-PAMM course on 'preclinical and early-phase clinical pharmacology.' Expert Rev Clin Pharmacol. 2019 Nov 2;12(11):1033–6.
51. Park JJH, Siden E, Zoratti MJ, Dron L, Harari O, Singer J, et al. Systematic review of basket trials, umbrella trials, and platform trials: a

For peer review only

landscape analysis of master protocols. Trials. 2019 Dec;20(1):572.
52. Gilson C, Chowdhury S, Parmar MKB, Sydes MR. Incorporating Biomarker Stratification into STAMPEDE: an Adaptive Multi-arm, Multi-stage Trial Platform. Clin Oncol. 2017 Dec;29(12):778–86.
53. Alexander BM, Trippa L, Gaffey S, Arrillaga-Romany IC, Lee EQ, Rinne ML, et al. Individualized Screening Trial of Innovative Glioblastoma Therapy (INSIGhT): A Bayesian Adaptive Platform Trial to Develop Precision Medicines for Patients With Glioblastoma. JCO Precis Oncol. 2019 Dec;(3):1–13.
54. Semler MW, Bernard GR, Aaron SD, Angus DC, Biros MH, Brower RG, et al. Identifying Clinical Research Priorities in Adult Pulmonary and Critical Care. NHLBI Working Group Report. Am J Respir Crit Care Med. 2020 Aug 15;202(4):511–23.
55. Skamene T, Siu LL, Renouf DJ, Laskin JJ, Bedard PL, Jones SJM, et al. Canadian profiling and targeted agent utilization trial (CAPTUR/PM.1): A phase II basket precision medicine trial. In 2018.
56. Hernandez-Martinez J-M, Sánchez-Reyes R, De la Garza-Salazar JG, Arrieta O. Onco-omics Approaches and Applications in Clinical Trials for Cancer Patients. In: Translational Research and Onco-Omics Applications in the Era of Cancer Personal Genomics. Springer; 2019. p. 79–90.
57. Lopez-Chavez A, Thomas A, Rajan A, Raffeld M, Morrow B, Kelly R, et al. Molecular Profiling and Targeted Therapy for Advanced Thoracic Malignancies: A Biomarker-Derived, Multiarm, Multihistology Phase II Basket Trial. J Clin Oncol. 2015 Mar 20;33(9):1000–7.
58. Patel SP, Othus M, Chae YK, Giles FJ, Hansel DE, Singh PP, et al. A Phase II Basket Trial of Dual Anti–CTLA-4 and Anti–PD-1 Blockade in Rare Tumors (DART SWOG 1609) in Patients with Nonpancreatic Neuroendocrine Tumors. Clin Cancer Res. 2020 May 15;26(10):2290–6.
59. Timmers L, van Waalwijk van Doorn S, Pisters A, van Saase L, Voest E. PPM1 A RISK SHARING MODEL FOR BIOMARKER-DRIVEN TREATMENT OF RARE SUBGROUPS OF CANCER PATIENTS. Value Health. 2019 Nov;22:S837.
60. D'Angelo S, Blay J, Chow W, Demetri G, Thistlethwaite F, Wagner M. Autologous T cells with NY-ESO-1-specific T-cell receptor (GSK3377794) in HLA-A*02+previously-treated and -untreated advanced metastatic/unresectable synovial sarcoma: A master protocol study design. In Journal for Immunotherapy of Cancer; 2019. p. 282.
61. Cecchini M, Rubin EH, Blumenthal GM, Ayalew K, Burris HA, Russell-Einhorn M, et al. Challenges with Novel Clinical Trial Designs: Master Protocols. Clin Cancer Res. 2019 Apr 1;25(7):2049–57.
62. Marabelle A, Le DT, Ascierto PA, Di Giacomo AM, De Jesus-Acosta A, Delord J-P, et al. Efficacy of Pembrolizumab in Patients With Noncolorectal High Microsatellite Instability/Mismatch Repair–Deficient Cancer: Results From the Phase II KEYNOTE-158 Study. J Clin Oncol. 2020 Jan 1;38(1):1–10.
63. Bang Y-J, Kaufman B, Geva R, Stemmer SM, Hong S-H, Lee J-S, et al. An open-label, phase II basket study of olaparib and durvalumab (MEDIOLA): Results in patients with relapsed gastric cancer. In 2019.
64. Domchek SM, Postel-Vinay S, Im S-A, Hee Park Y, Delord J-P, Italiano A, et al. An open-label, phase II basket study of olaparib and durvalumab (MEDIOLA): Updated results in patients with germline BRCA-mutated (gBRCAm) metastatic breast cancer (MBC). In San Antonio, Texas; 2018.
65. Krebs M, Ross K, Kim S, De Jonge M, Barlesi F, Postel-Vinay S, et al. P1.15-004 An Open-Label, Multitumor Phase II Basket Study of

For peer review only - http://bmjopen.bmj.com/site/about/guidelines.xhtml

Olaparib and Durvalumab (MEDIOLA): Results in Patients with Relapsed SCLC. J Thorac Oncol. 2017 Nov;12(11):S2044–5.
66. Hyman DM, Taylor BS, Baselga J. Implementing Genome-Driven Oncology. Cell. 2017 Feb;168(4):584–99.
67. Thavaneswaran S, Sebastian L, Ballinger M, Best M, Hess D, Lee CK, et al. Cancer Molecular Screening and Therapeutics (MoST): a framework for multiple, parallel signal-seeking studies of targeted therapies for rare and neglected cancers. Med J Aust. 2018 Oct;209(8):354–5.
68. Thavaneswaran S, Sebastian L, Ballinger M, Cowley M, Grady J, Joshua A, et al. The Cancer Molecular Screening and Therapeutics Program (MoST) – A molecular screening platform with multiple, parallel, signal-seeking therapeutic substudies. In Annals of Oncology; 2018. p. viii133–48.
69. Conter HJ, MacDonald LD, Fiset S, Bramhecha YM, Chaney M, Rosu GN. Safety and efficacy results of the combination of DPX-Survivac, pembrolizumab and intermittent low dose cyclophosphamide (CPA) in subjects with advanced and metastatic solid tumours: Preliminary results from the hepatocellular carcinoma (HCC), NSCLC, bladder cancer, & MSI-H cohorts. Ann Oncol. 2019 Oct;30:v494.
70. Li B, Zauderer M, Chaft J, Drilon A, Eng J, Sima C. Ado-trastuzumab emtansine for HER2 amplified or HER2 overexpressed cancers: A phase II "basket" trial. In Cancer Res; 2015.
71. Barroilhet L, Matulonis U. The NCI-MATCH trial and precision medicine in gynecologic cancers. Gynecol Oncol. 2018 Mar;148(3):585–90.
72. Do K, Coyne GO, Chen AP. An overview of the NCI precision medicine trials—NCI MATCH and MPACT. Chin Clin Oncol. 2015;4(3):8.
73. Mandrekar SJ, Sargent DJ. All-Comers versus Enrichment Design Strategy in Phase II Trials. J Thorac Oncol. 2011 Apr;6(4):658–60.
74. Mazzarella L, Morganti S, Marra A, Trapani D, Tini G, Pelicci P, et al. Master protocols in immuno-oncology: do novel drugs deserve novel designs? J Immunother Cancer. 2020 Mar;8(1):e000475.
75. Moore A, Jones R. Supporting and enhancing peer review in the *BJGP*. Br J Gen Pract. 2014 Jul;64(624):e459–61.
76. O'Brien C, Carter L, Cook N, Dean E. Novel Early Phase Clinical Trial Design in Oncology. Pharm Med. 2017 Oct;31(5):297–307.
77. Renfro LA, Sargent DJ. Statistical controversies in clinical research: basket trials, umbrella trials, and other master protocols: a review and examples. Ann Oncol. 2017 Jan;28(1):34–43.
78. Simon R. Genomic Alteration–Driven Clinical Trial Designs in Oncology. Ann Intern Med. 2016 Aug 16;165(4):270.
79. Soldatos, Kaduthanam, Jackson. Precision Oncology—The Quest for Evidence. J Pers Med. 2019 Sep 5;9(3):43.
80. Zardavas D, Piccart-Gebhart M. Clinical Trials of Precision Medicine through Molecular Profiling: Focus on Breast Cancer. Am Soc Clin Oncol Educ Book. 2015 May;(35):e183–90.
81. Simon R. New designs for basket clinical trials in oncology. J Biopharm Stat. 2018 Mar 4;28(2):245–55.
82. Coyne GO, Takebe N, Chen AP. Defining precision: The precision medicine initiative trials NCI-MPACT and NCI-MATCH. Curr Probl Cancer. 2017 May;41(3):182–93.
83. Tao JJ, Schram AM, Hyman DM. Basket Studies: Redefining Clinical Trials in the Era of Genome-Driven Oncology. Annu Rev Med. 2018 Jan 29;69(1):319–31.
84. Nakamura Y, Komatsu Y, Kato K, Shinozaki E, Bando H, Kato T, et al. bTMB-High Basket trial: A multicenter phase II trial of nivolumab monotherapy in patients with advanced gastrointestinal cancers with high blood tumor mutational burden (bTMB). In 2019.

For peer review only - http://bmjopen.bmj.com/site/about/guidelines.xhtml

85. Dienstmann R, Rodon J, Tabernero J. Optimal design of trials to demonstrate the utility of genomically-guided therapy: Putting Precision Cancer Medicine to the test. Mol Oncol. 2015 May;9(5):940–50.
86. Kummar S, Chen A, Lih J, Williams M, Rubinstein L, Conley B, et al. SP007 Molecular profiling based assignment of cancer therapy (MPACT). Eur J Cancer. 2013 Nov;49:S3.
87. Verweij J, Hendriks HR, Zwierzina H, Hanauske, Wacheck V, Collignon O, et al. Innovation in oncology clinical trial design. Cancer Treat Rev. 2019 Mar;74:15–20.
88. Brana I, Massard C, Baird RD, Opdam F, Schlenk RF, De Petris L, et al. Basket of baskets (BoB): A modular, open label, phase II, multicenter study to evaluate targeted agents in molecularly selected populations with advanced solid tumors. In 2019.
89. Garralda E, Dienstmann R, Piris-Giménez A, Braña I, Rodon J, Tabernero J. New clinical trial designs in the era of precision medicine. Mol Oncol. 2019 Mar;13(3):549–57.
90. Hofmann D, Nitz U, Gluz O, Kates RE, Schinkoethe T, Staib P, et al. WSG ADAPT – adjuvant dynamic marker-adjusted personalized therapy trial optimizing risk assessment and therapy response prediction in early breast cancer: study protocol for a prospective, multi-center, controlled, non-blinded, randomized, investigator initiated phase II/III trial. Trials. 2013;14(1):261.
91. Nitz U, Gluz O, von Schumann R, Hofmann D, Kates RE, Kuemmel S, et al. ADAPT - Adjuvant Dynamic marker-Adjusted Personalized Therapy trial optimizing risk assessment and therapy response prediction in early breast cancer. In 2015.
92. Cochrane Library. Trial for the optimisation of risk assessment and therapy success predicition in patients with early breast cancer by the use of biomarkers in advance to therapy decission-making to personalize therapies [Internet]. Available from: https://www.cochranelibrary.com/central/doi/10.1002/central/CN-01873376/full
93. Abrams J, Conley B, Mooney M, Zwiebel J, Chen A, Welch JJ, et al. National Cancer Institute's Precision Medicine Initiatives for the New National Clinical Trials Network. Am Soc Clin Oncol Educ Book. 2014 May;(34):71–6.
94. Yee LM, McShane LM, Freidlin B, Mooney MM, Korn EL. Biostatistical and Logistical Considerations in the Development of Basket and Umbrella Clinical Trials: Cancer J. 2019;25(4):254–63.
95. Heckman-Stoddard BM, Smith JJ. Precision Medicine Clinical Trials: Defining New Treatment Strategies. Semin Oncol Nurs. 2014 May;30(2):109–16.
96. Ferrarotto R, Redman MW, Gandara DR, Herbst RS, Papadimitrakopoulou V. Lung-MAP-framework, overview, and design principles. Chin Clin Oncol. 2015;4(3):1–6.
97. Gandara DR, Hammerman PS, Sos ML, Lara PN, Hirsch FR. Squamous Cell Lung Cancer: From Tumor Genomics to Cancer Therapeutics. Clin Cancer Res. 2015 May 15;21(10):2236–43.
98. Lam VK, Papadimitrakopoulou V. Master protocols in lung cancer: experience from Lung Master Protocol. Curr Opin Oncol. 2018 Mar;30(2):92–7.
99. Hollingsworth SJ. Precision medicine in oncology drug development: a pharma perspective. Drug Discov Today. 2015 Dec;20(12):1455–63.
100. Redman MW, Allegra CJ. The Master Protocol Concept. Semin Oncol. 2015 Oct;42(5):724–30.
101. Fennell D, Hudka M, Darlison L, Lord K, Bzura A, Dzialo J, et al. P2.06-02 Mesothelioma Stratified Therapy (MiST): A Phase IIA

Umbrella Trial for Accelerating the Development of Precision Medicines. J Thorac Oncol. 2019 Oct;14(10):S755–6.
102. Schmoll H-J, Arnold D, de Gramont A, Ducreux M, Grothey A, O'Dwyer PJ, et al. MODUL—a multicenter randomized clinical trial of biomarker-driven maintenance therapy following first-line standard induction treatment of metastatic colorectal cancer: an adaptable signal-seeking approach. J Cancer Res Clin Oncol. 2018 Jun;144(6):1197–204.
103. Zhou Q, Zhang X-C, Tu H-Y, Gan B, Wang B-C, Xu C-R, et al. Biomarker-integrated study of single agent targeting molecular alterations of PI3KCA, MET, ALK, ROS1, KRAS, NRAS or BRAF in advanced NSCLC: Phase 2 umbrella trial in China (CTONG1505). In 2018.
104. Park JJH, Hsu G, Siden EG, Thorlund K, Mills EJ. An overview of precision oncology basket and umbrella trials for clinicians. CA Cancer J Clin. 2020 Mar;70(2):125–37.
105. Bell S, Copel J, Smith A. The pros and cons of an "umbrella" trial design for a rare disease from a trial management and data management perspective. In Liverpool, United Kingdom; 2017.
106. Sebag-Montefiore D, Adams R, Bell S, Berkman L, Gilbert DC, Glynne-Jones R, et al. The Development of an Umbrella Trial (PLATO) to Address Radiation Therapy Dose Questions in the Locoregional Management of Squamous Cell Carcinoma of the Anus. Int J Radiat Oncol. 2016 Oct;96(2):E164–5.
107. ISRCTN Registry. Precision Panc: Advancing personalised medicine treatment strategies for pancreatic cancer [Internet]. Available from: https://www.isrctn.com/ISRCTN14879538
108. Grose DB, McKay CJ, Cooke S, Graham JS, Duthie F, Jamieson N, et al. PRIMUS-002: A multicentre, open-label, phase II study examining FOLFOX and nab-paclitaxel (FA) and nab-paclitaxel and gemcitabine (AG) as neoadjuvant therapy for (borderline) resectable pancreatic cancer (PC), focusing on biomarker and liquid biopsy development. In Chicago;
109. Park S. P2.12-05 SUKSES (Small Cell Lung Cancer Umbrella Korea Studies): A Phase II Biomarker-Driven Umbrella Study in Relapsed or Refractory SCLC. In p. 1.
110. Park S, Shim J, Jung HA, Sun J-M, Lee S-H, Park W-Y, et al. Biomarker driven phase II umbrella trial study of AZD1775, AZD2014, AZD2811 monotherapy in relapsed small cell lung cancer. In 2019.
111. Galot R, Le Tourneau C, Saada-Bouzid E, Daste A, Even C, Debruyne PR, et al. A phase II study of monalizumab in patients with recurrent/metastatic (RM) squamous cell carcinoma of the head and neck (SCCHN): Results of the I1 cohort of the EORTC-HNCG-1559 trial (UPSTREAM). Ann Oncol. 2019 Oct;30:v449–50.
112. Lee J, Kim ST, Kim K, Lee H, Kozarewa I, Mortimer PG, et al. Tumor genomic profiling guides metastatic gastric cancer patients to targeted treatment: The VIKTORY Umbrella Trial. Cancer Discov. 2019 Jul 17;CD-19-0442.
113. De Mattos-Arruda L, Rodon J. Pilot Studies for Personalized Cancer Medicine: Focusing on the Patient for Treatment Selection. The Oncologist. 2013 Nov;18(11):1180–8.
114. Lee J-Y, Yi JY, Kim H-S, Lim J, Kim S, Nam BH, et al. An umbrella study of biomarker-driven targeted therapy in patients with platinum-resistant recurrent ovarian cancer: a Korean Gynecologic Oncology Group study (KGOG 3045), AMBITION. Jpn J Clin Oncol. 2019 Aug 1;49(8):789–92.
115. Noda S, Yonemori K, Shirakawa N, Okuma HS, Shimizu T, Hirakawa A, et al. MASTER KEY project: A basket/umbrella trial for rare cancers in Japan. In 2019.

For peer review only

For peer review only

Supplementary file VII. Trials evaluating personalised versus no personalised medicine

Type of trial designs	Example(s)	Trial registration num.	Recruitment status as of 12 March 2021	Clinical Field	Phase	References
Adaptive strategy designs for biomarkers with measurement error	OPTIMA	ISRCTN42400492	Ongoing	Breast Cancer	n/a[1]	(1)
Basket design	NCI-MPACT	NCT01827384	Completed	Advanced malignant solid neoplasm	II	(2–4)
	SHIVA	NCT01771458	Unknown*	Reccurent/Metastatic Solid; Tumor Disease	II	(5)
	IMPACT II	NCT02152254	Completed	Reccurent/Metastatic Solid; Tumor Disease	II	(6)
Biomarker strategy design with biomarker assessment in the control arm	ERCC1	NCT00801736	Completed	Lung cancer	III	(7)
	GILT docetaxel	NCT00174629	Completed	Lung cancer	III	(8)
	LIFT	NCT02498977	Completed	Transplantation, Liver	IV	(9)
Biomarker-strategy design without biomarker assessment in the control arm	GUIDE-IT	NCT01685840	Completed	Chronic Heart Failure	n/a[1]	(10)
	iPEGASUS	NCT03021525	Ongoing	Hemodynamic Instability, Cardiac Output (High), Peroperative Complication	n/a[1]	(11)
	OCTOPUS	ISRCTN81464462	Completed	Mild head injury	n/a[1]	(8)
	PUFFIN	NCT03654508	Ongoing	Asthma	n/a[1]	(12)
Modified biomarker	SHIVA	NCT01771458	Unknown*	Reccurent/Metastatic Solid; Tumor Disease	II	(1,13–15)

For peer review only

strategy design	NCI-MPACT	NCT01827384	Completed	Advanced malignant solid neoplasm	II	(15)
Outcome-based adaptive randomization design	ProBio	NCT03903835	Ongoing	Prostate cancer	III	(16)
Platform	SHIVA	NCT01771458	Unknown*	Reccurent/Metastatic Solid; Tumor Disease	II	(17)
Sequential Multiple Assignment Randomized Trial (SMART)	Siyaphambili Study	NCT03500172	Ongoing	HIV	n/a[1]	(18)
Umbrella	UPSTREAM	NCT03088059	Ongoing	Head and Neck Squamous Cell Carcinoma	II	(19)
	SAFIR02_Braest	NCT02299999	Completed	Breast Cancer	II	(20)
	SAFIR02_Lung	NCT02117167	Completed	Lung cancer	II	(17)

[1]Not applicable is used on the Clinicaltrilas.gov website to describe trials without FDA-defined phases including trials of devices or behavioural interventions.

References

1. Renfro LA, Mallick H, An M-W, Sargent DJ, Mandrekar SJ. Clinical trial designs incorporating predictive biomarkers. Cancer Treat Rev. 2016 Feb;43:74–82.
2. Coyne GO, Takebe N, Chen AP. Defining precision: The precision medicine initiative trials NCI-MPACT and NCI-MATCH. Curr Probl Cancer. 2017 May;41(3):182–93.
3. Lopez-Chavez A, Thomas A, Rajan A, Raffeld M, Morrow B, Kelly R, et al. Molecular Profiling and Targeted Therapy for Advanced Thoracic Malignancies: A Biomarker-Derived, Multiarm, Multihistology Phase II Basket Trial. J Clin Oncol. 2015 Mar 20;33(9):1000–7.
4. Simon R. New designs for basket clinical trials in oncology. J Biopharm Stat. 2018 Mar 4;28(2):245–55.
5. Tao JJ, Schram AM, Hyman DM. Basket Studies: Redefining Clinical Trials in the Era of Genome-Driven Oncology. Annu Rev Med. 2018 Jan 29;69(1):319–31.
6. Fountzilas E, Tsimberidou AM. Overview of precision oncology trials: challenges and opportunities. Expert Rev Clin Pharmacol. 2018 Aug 3;11(8):797–804.
7. Freidlin B, McShane LM, Korn EL. Randomized Clinical Trials With Biomarkers: Design Issues. JNCI J Natl Cancer Inst. 2010 Feb 3;102(3):152–60.
8. Antoniou M, Kolamunnage-Dona R, Jorgensen A. Biomarker-Guided Non-Adaptive Trial Designs in Phase II and Phase III: A Methodological Review. J Pers Med. 2017 Jan 25;7(1):1.
9. Clinicaltrials.gov. Liver Immunosuppression Free Trial (LIFT) [Internet]. Available from: https://clinicaltrials.gov/ct2/show/NCT02498977
10. Ahmad T, O'Connor CM. Therapeutic Implications of Biomarkers in Chronic Heart Failure. Clin Pharmacol Ther. 2013 Oct;94(4):468–79.
11. Funcke S, Saugel B, Koch C, Schulte D, Zajonz T, Sander M, et al. Individualized, perioperative, hemodynamic goal-directed therapy in major abdominal surgery (iPEGASUS trial): study protocol for a randomized controlled trial. Trials. 2018 Dec;19(1):273.
12. Vijverberg SJ, Pijnenburg MW, Hövels AM, Koppelman GH, Maitland-van der Zee A-H. The need for precision medicine clinical trials in childhood asthma: rationale and design of the PUFFIN trial. Pharmacogenomics. 2017 Mar;18(4):393–401.

13. Dienstmann R, Rodon J, Tabernero J. Optimal design of trials to demonstrate the utility of genomically-guided therapy: Putting Precision Cancer Medicine to the test. Mol Oncol. 2015 May;9(5):940–50.
14. Paoletti X, Asselain B, Kamal M, Servant N, Huppé P, Bieche I, et al. Design and statistical principles of the SHIVA trial. Chin Clin Oncol. 2015;4(3):1–10.
15. Renfro LA, An M-W, Mandrekar SJ. Precision oncology: A new era of cancer clinical trials. Cancer Lett. 2017 Feb;387:121–6.
16. Lindberg J, De Laere B, Crippa A, Eklund M, Grönberg H. ProBio: An outcome-adaptive, multi-arm, open-label, multiple assignment randomised controlled biomarker-driven trial in patients with metastatic castration-resistant prostate cancer. In Barcelona, Spain;
17. Leonetti A, Boyd L, Giuliani J, Giovannetti E, Garajová I. Light and shadow on innovative clinical trial designs: reflections from the EORTC-PAMM course on 'preclinical and early-phase clinical pharmacology.' Expert Rev Clin Pharmacol. 2019 Nov 2;12(11):1033–6.
18. Clinicaltrials.gov. HIV Treatment Retention Interventions for Women Living With HIV (Siyaphambili Study) [Internet]. Available from: https://clinicaltrials.gov/ct2/show/NCT03500172
19. Galot R, Le Tourneau C, Saada-Bouzid E, Daste A, Even C, Debruyne PR, et al. A phase II study of monalizumab in patients with recurrent/metastatic (RM) squamous cell carcinoma of the head and neck (SCCHN): Results of the I1 cohort of the EORTC-HNCG-1559 trial (UPSTREAM). Ann Oncol. 2019 Oct;30:v449–50.
20. Hernandez-Martinez J-M, Sánchez-Reyes R, De la Garza-Salazar JG, Arrieta O. Onco-omics Approaches and Applications in Clinical Trials for Cancer Patients. In: Translational Research and Onco-Omics Applications in the Era of Cancer Personal Genomics. Springer; 2019. p. 79–90.

For peer review only

Preferred Reporting Items for Systematic reviews and Meta-Analyses extension for Scoping Reviews (PRISMA-ScR) Checklist

SECTION	ITEM	PRISMA-ScR CHECKLIST ITEM	REPORTED ON PAGE #
**TITLE**			
Title	1	Identify the report as a scoping review.	1
**ABSTRACT**			
Structured summary	2	Provide a structured summary that includes (as applicable): background, objectives, eligibility criteria, sources of evidence, charting methods, results, and conclusions that relate to the review questions and objectives.	2
**INTRODUCTION**			
Rationale	3	Describe the rationale for the review in the context of what is already known. Explain why the review questions/objectives lend themselves to a scoping review approach.	3
Objectives	4	Provide an explicit statement of the questions and objectives being addressed with reference to their key elements (e.g., population or participants, concepts, and context) or other relevant key elements used to conceptualize the review questions and/or objectives.	3
**METHODS**			
Protocol and registration	5	Indicate whether a review protocol exists; state if and where it can be accessed (e.g., a Web address); and if available, provide registration information, including the registration number.	4
Eligibility criteria	6	Specify characteristics of the sources of evidence used as eligibility criteria (e.g., years considered, language, and publication status), and provide a rationale.	4
Information sources*	7	Describe all information sources in the search (e.g., databases with dates of coverage and contact with authors to identify additional sources), as well as the date the most recent search was executed.	4
Search	8	Present the full electronic search strategy for at least 1 database, including any limits used, such that it could be repeated.	Supplementary file 1
Selection of sources of evidence†	9	State the process for selecting sources of evidence (i.e., screening and eligibility) included in the scoping review.	4
Data charting process‡	10	Describe the methods of charting data from the included sources of evidence (e.g., calibrated forms or forms that have been tested by the team before their use, and whether data charting was done independently or in duplicate) and any processes for obtaining and confirming data from investigators.	5
Data items	11	List and define all variables for which data were sought and any assumptions and simplifications made.	5
Critical appraisal of individual sources of evidence§	12	If done, provide a rationale for conducting a critical appraisal of included sources of evidence; describe the methods used and how this information was used in any data synthesis (if appropriate).	na
Synthesis of results	13	Describe the methods of handling and summarizing the data that were charted.	5

For peer review only - http://bmjopen.bmj.com/site/about/guidelines.xhtml

SECTION	ITEM	PRISMA-ScR CHECKLIST ITEM	REPORTED ON PAGE #
**RESULTS**			
Selection of sources of evidence	14	Give numbers of sources of evidence screened, assessed for eligibility, and included in the review, with reasons for exclusions at each stage, ideally using a flow diagram.	Figure 1
Characteristics of sources of evidence	15	For each source of evidence, present characteristics for which data were charted and provide the citations.	6 - Suppl. file 3
Critical appraisal within sources of evidence	16	If done, present data on critical appraisal of included sources of evidence (see item 12).	na
Results of individual sources of evidence	17	For each included source of evidence, present the relevant data that were charted that relate to the review questions and objectives.	Supplementary files 4-5-6
Synthesis of results	18	Summarize and/or present the charting results as they relate to the review questions and objectives.	5-6-7-8-9
**DISCUSSION**			
Summary of evidence	19	Summarize the main results (including an overview of concepts, themes, and types of evidence available), link to the review questions and objectives, and consider the relevance to key groups.	9
Limitations	20	Discuss the limitations of the scoping review process.	10
Conclusions	21	Provide a general interpretation of the results with respect to the review questions and objectives, as well as potential implications and/or next steps.	10
**FUNDING**			
Funding	22	Describe sources of funding for the included sources of evidence, as well as sources of funding for the scoping review. Describe the role of the funders of the scoping review.	14

JBI = Joanna Briggs Institute; PRISMA-ScR = Preferred Reporting Items for Systematic reviews and Meta-Analyses extension for Scoping Reviews.
* Where *sources of evidence* (see second footnote) are compiled from, such as bibliographic databases, social media platforms, and Web sites.
† A more inclusive/heterogeneous term used to account for the different types of evidence or data sources (e.g., quantitative and/or qualitative research, expert opinion, and policy documents) that may be eligible in a scoping review as opposed to only studies. This is not to be confused with *information sources* (see first footnote).
‡ The frameworks by Arksey and O'Malley (6) and Levac and colleagues (7) and the JBI guidance (4, 5) refer to the process of data extraction in a scoping review as data charting.
§ The process of systematically examining research evidence to assess its validity, results, and relevance before using it to inform a decision. This term is used for items 12 and 19 instead of "risk of bias" (which is more applicable to systematic reviews of interventions) to include and acknowledge the various sources of evidence that may be used in a scoping review (e.g., quantitative and/or qualitative research, expert opinion, and policy document).

From: Tricco AC, Lillie E, Zarin W, O'Brien KK, Colquhoun H, Levac D, et al. PRISMA Extension for Scoping Reviews (PRISMAScR): Checklist and Explanation. Ann Intern Med. 2018;169:467–473. doi: 10.7326/M18-0850.

For peer review only - http://bmjopen.bmj.com/site/about/guidelines.xhtml
